# Remote 1,4‐Carbon‐to‐Carbon Boryl Migration: From a Mechanistic Challenge to a Valuable Synthetic Application of Bicycles

**DOI:** 10.1002/advs.202309779

**Published:** 2024-02-15

**Authors:** Paula Dominguez‐Molano, Albert Solé‐Daura, Jorge J. Carbó, Elena Fernández

**Affiliations:** ^1^ Departament de Química Física i Inorgànica Universitat Rovira i Virgili Tarragona 43007 Spain

**Keywords:** bicycles, boryl migration, copper, mechanism, Simmons‐smith

## Abstract

The present paper reports a remote carbon‐to‐carbon boryl migration via an intramolecular 1,4‐B/Cu shift, which establishes an in situ stereospecific electrophilic trap on the alkene moiety. The synthetic application is developed to prepare functionalized cyclopentenes by means of a palladium‐catalyzed regioselective intramolecular coupling that completes a strategic cyclopropanation and generates valuable structural bicyclic systems. The mechanism is characterized by DFT (density functional theory) calculations which showed that the 1,4‐migration proceeds through an intramolecular, nucleophilic attack of the copper‐alkyl moiety on the boron atom bonded to the C(sp^2^), leading to a 5‐membered boracycle structure. The computation of the 1,3‐ and 1,4‐B/Cu shifts is also compared as is the impact of the endo‐ or exocyclic alkene on the reaction kinetics.

## Graphical abstract

The intramolecular 1,4‐B/Cu shift justifies the observed remote carbon‐to‐carbon boryl migration, which makes stereoselective electrophilic trapping on the alkene moiety possible. Subsequent palladium‐catalyzed regioselective intramolecular coupling enables functionalized cyclopentenes to be prepared that complete strategic cyclopropanation and deliver structurally valuable bicyclic systems. DFT mechanistic studies show that remote boryl migration occurs efficiently through 5‐membered boracycle intermediates.

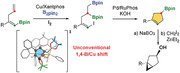

## 1 Introduction

**Scheme 1:**
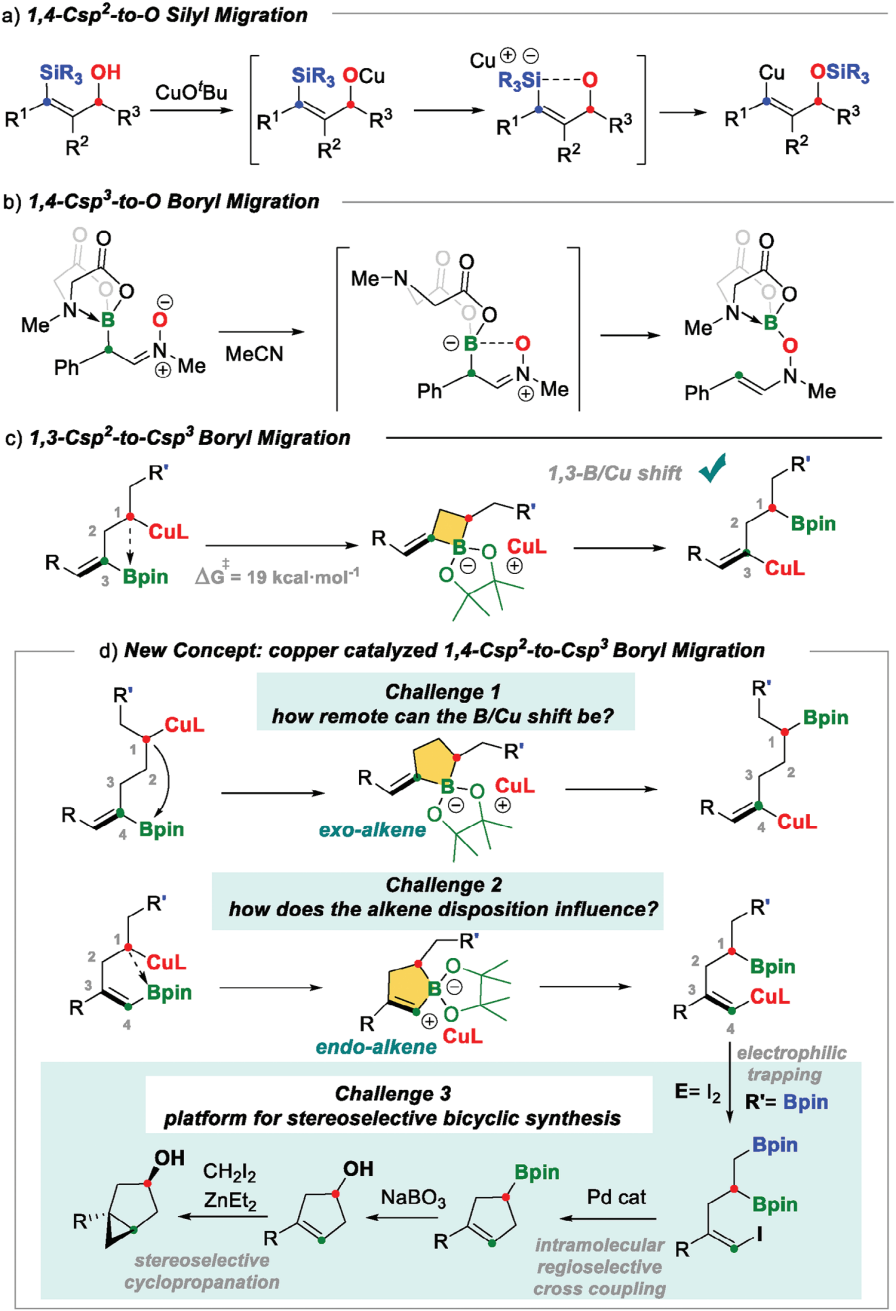
1,4‐Si or 1,4‐B migration sequences.

The 1,4‐migration orchestrated by transition metals has provided an unexpected way of translocating a variety of atoms along molecular structures. The main complexes involved in 1,4‐metal rearrangements are Rh and Pd although other metals such as Ni, Pt, Co, and Fe also showed some ability to engage in remote atom migration.^[^ ^1^, ^2^ ^]^ Cu complexes are less conventionally used to relocate atoms and in some cases, the assistance of heteroatoms has been required.^[^ ^3^, ^4^, ^5^, ^6^, ^7^, ^8^, ^9^, ^10^, ^11^, ^12^, ^13^ ^]^ Copper (I) has been used for the 1,4‐Csp^2^‐to‐O silyl migration via hypercoordinated cyclic silicates in (*Z*)‐*γ*‐trimethylsilyl allylic alcohol to promote the Brook‐type rearrangement (**Scheme** **1**a).^[^ ^14^ ^]^ Hypercoordinated cyclic boronic esters have also been postulated as the key intermediates in the 1,4‐boryl migration of boryl nitrone, which forms a five‐membered zwitterionic intermediate that evolves to O‐boryl hydroxylamine (Scheme 1b).^[^ ^15^ ^]^ Oxygen assistance seems to be the key factor in the success of 1,4‐silyl or 1,4‐boryl migration, via five‐membered ring intermediates, with hypercoordinated Si and B atoms, respectively.

Our aim in this paper is to discuss a new concept, based on a 1,4‐boryl copper shift without the assistance of a heteroatom, to envisage a remote 1,4‐carbon‐to‐carbon boryl migration initiated by the nucleophilic attack by the copper‐alkyl moiety Cu‐C(sp^3^) on the empty p orbital of the boron atom bonded to the C(sp^2^). This leads to a hyper‐coordinated boracycle structure responsible for the remote Cu/B shift. Our hypothesis is founded on previously identified 1,3‐B/Cu shifts between C(sp^2^)‐Bpin and C(sp^3^)‐Cu fragments, which proceed through four‐membered boracycle intermediates with an exo‐alkene moiety (Scheme 1c).^[^ ^16^ ^]^ Now we explore how remote the B/Cu shift can be, in an attempt to achieve the 1,4‐B/Cu rearrangement involving five‐membered boracycle intermediates (Scheme 1d). Using density functional theory (DFT) studies, we elucidate the reaction mechanism and assess how the endo‐ or exo‐alkene disposition affects the reactivity. We expect the endocyclic alkene to be advantageous for stereospecific iodination followed by Pd‐catalyzed regioselective cross‐coupling to construct cyclopentene scaffolds, which can be transformed into bicycles via stereoselective Simmons‐Smith cyclopropanation (Scheme 1d).

## 2 Results and Discussion

**Scheme 2:**
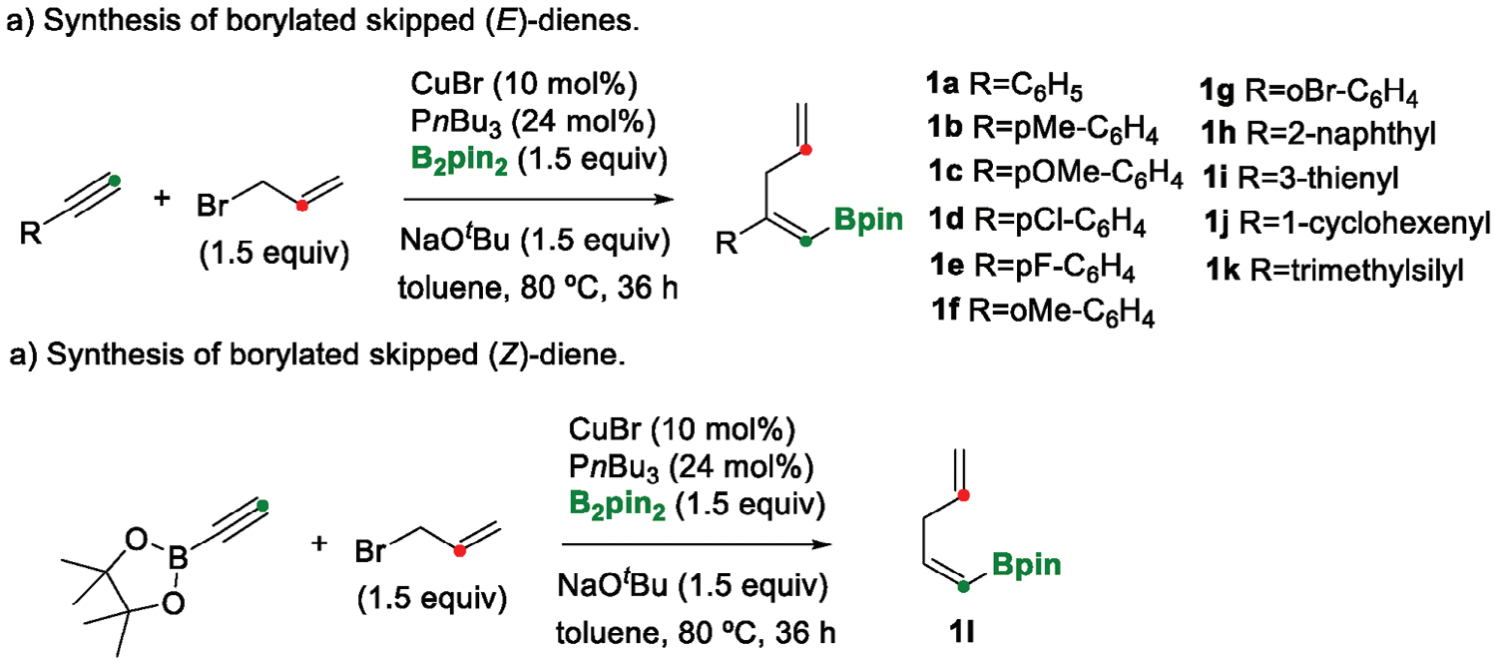
Synthesis of borylated skipped (*E*)‐dienes. Reaction conditions: alkyne (2 mmol), allyl bromide (3 mmol), CuBr (0.2 mmol), NaO*^t^* Bu (3 mmol), B_2_pin_2_ (3 mmol), and P*_n_* Bu_3_ (0.48 mmol) were stirred in toluene (10 mL) under an argon atmosphere at 80 °C for 36 h.

To study the 1,4‐B/Cu shift, we first prepared (*E*)‐trisubstituted vinyl pinacol boronic esters with a 1,4‐diene skeleton to be used as model substrates. To this end, we followed an approach by Zhao and Wang^[^ ^17^ ^]^ based on the efficient copper‐catalyzed regioselective formal allylboration of terminal alkynes with B_2_pin_2_ (bis(pinacolato)diboron). We reproduced the synthesis for substrates 1a‐1i, and we prepared compounds 1j and 1k for the first time here, to show that the reaction was compatible with alkenyl and silyl groups (**Scheme** **2**a). Surprisingly when 2‐ethynyl‐4,4,5,5‐tetramethyl‐1,3,2‐dioxaborolane reacted with CuBr/P*_n_* Bu_3_ toward the formal allylboration, we observed that the reaction generated product 1l, from which the original Bpin (pinacolboryl) moiety had been removed, suggesting that the synthesis followed a protodeborylation pathway (Scheme 2b).

**Scheme 3:**
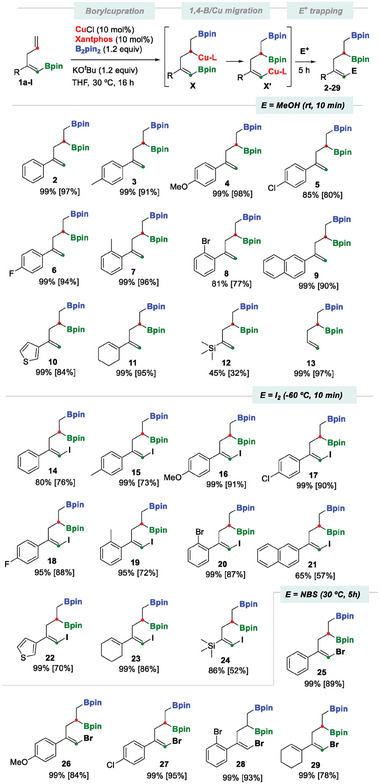
One pot transformation of borylated skipped (*E*)‐dienes via borylcupration/1,4‐B/Cu shift/electrophilic trapping. Reaction conditions for borylcupration: dienes 1a‐l (0.2 mmol), B_2_pin_2_ (0.24 mmol), CuCl (0.01 mmol), Xantphos (0.01 mmol) NaO*^t^* Bu (0.24 mmol) were stirred in THF (0.5 mL) under an argon atmosphere at 30 °C for 16 h. Protonation with 1 mL MeOH for 10 min. Iodination with I_2_ (0.4 mmol in 2 ml THF) at −60 °C for 10 min. Bromination with NBS (0.4 mmol in 2 mL THF) at 30 °C for 5 h. The NMR yield was calculated with naphthalene as the internal standard. Isolated yield in brackets.

We then went on to work on the borylcupration of borylated skipped (*E*)‐dienes, using the efficient catalytic system CuCl/Xantphos for regioselective borylcupration.^[^ ^18^, ^19^, ^20^ ^]^ The conversion of model substrate 1a into diborated alkene 2 was quantitatively performed with 10 mol% of CuCl/Xantphos, in the presence of 1.2 equiv. of B_2_pin_2_ and KO*^t^* Bu as a base, at 30 °C, in tetrahydrofurane (THF) (**Scheme** **3**). Product 2 was formed by Cu‐catalyzed regioselective borylcupration on the terminal alkene of 1a, leading to the organocopper species X. By means of a subsequent straightforward intramolecular 1,4‐B/Cu shift, this evolved to alkenyl copper intermediate X’, which was eventually trapped with a proton. This reaction proved to be general for substrates with electron‐withdrawing and electron‐donating aryl substituents, in both para and ortho positions (Scheme 3, products 3–8). The reaction showed noteworthy functional group tolerance with 2‐naphthyl and 3‐thienyl groups (Scheme 3, products 9–10). The introduction of a cyclohexenyl group into substrate 1j addressed the chemoselective issue during the initial borylcupration. However, only the terminal alkene was activated, which gave access to the desired diborated product 11. The compatibility of the trimethylsilyl group along the Cu‐catalyzed 1,4‐boryl migration played an important role in generating product 12, since no 1,3‐C(sp^2^)‐to‐C(sp^3^) silyl migration was observed in agreement with the higher hypercoordination ability of the boryl group versus the silyl group. Eventually, the non‐substituted substrate (*Z*)−4,4,5,5‐tetramethyl‐2‐(penta‐1,4‐dien‐1‐yl)−1,3,2‐dioxaborolane (1l) was efficiently transformed into diborated product 13 in high yields (Scheme 3). We also demonstrated that the use of [Cu(MeCN)_4_]PF_6_, instead of CuCl, led to the formation of the diborated products 2–13 in similar high yields.

Having evaluated the viability of selective borylcupration – 1,4‐B/Cu shift – protonation in a single operation, we then went on to explore the complementary I_2_‐iodonolysis^[^ ^21^ ^]^ of the alkenyl copper (I) intermediates X’ generated after borylcupration‐1/4‐B,Cu shift, which provided the key vinylic iodide products 14–24 in high yields (Scheme 3). Alternatively, the brominated trisubstituted alkenes 25–29 could also be efficiently generated by stereospecific electrophilic trapping by the addition of NBS (N‐bromosuccinimide) (Scheme 3).

**Figure 1:**
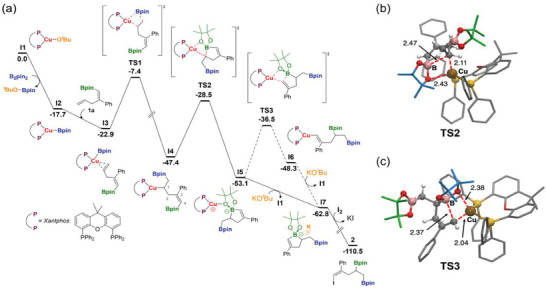
Free‐energy profile (kcal mol^−1^) for the borylcupration of skipped (*E*)‐diene 1a with B_2_pin_2_ catalyzed by Cu‐Xantphos complex in the presence of KO*^t^* Bu base, followed by I_2_‐iodonolysis a). 3D structures for transition states TS2 b) and TS3 c), where hydrogen atoms of the ligand substituents are omitted for clarity. Selected distances are given in Å.

To understand the mechanism of the 1,4‐B/Cu shift observed during the borylcupration of skipped (*E*)‐dienes, we conducted DFT calculations and compared them with our previous investigations on the 1,3‐B/Cu migration of the Bpin moiety from C(sp^2^) to C(sp^3^).^[^ ^16^ ^]^ **Figure** **1**a depicts the calculated free‐energy profile for the borylcupration mechanism of substrate 1a with B_2_pin_2_ catalyzed by the Cu(I)‐Xantphos (Xantphos = 4,5‐bis(diphenylphosphino)−9,9‐dimethylxanthene) complex in the presence of KO*^t^* Bu, followed by sequential electrophilic trapping with I_2_. The first part of the mechanism consists of 1) the in situ generation of the active catalyst Xantphos‐Cu(O*^t^* Bu) complex I1, 2) the σ‐bond metathesis with B_2_pin_2_ to form the Xantphos‐Cu‐boryl species I2, 3) the coordination of substrate 1a to the Cu complex through the terminal double bond to yield adduct I3, and 4) the regioselective borylcupration of the terminal double bond to yield the alkyl‐Cu(I) complex I4, whereby the Bpin moiety is regioselectively bonded to the terminal carbon. All these steps are well established and have been computationally studied in detail for related processes and substrates.^[^ ^16^, ^20^ ^]^ Here we observe the same main features: 1) the stepwise process to yield the alkyl‐Cu(I) complex goes downhill in energy, and 2) the computed free‐energy barrier for the borylcupration step has a moderate height of 15.5 kcal mol^−1^ (see Figure 1a). Subsequently, the alkyl‐copper(I) intermediate I4 can undergo the unprecedented 1,4‐B/Cu shift from remote C(sp^2^)‐Bpin to C(sp^3^)‐Cu position along the alkyl fragment.

The 1,4‐migration occurs in two steps (Figure 1a). In the first, the Cu‐alkyl moiety performs a nucleophilic attack on the boron atom of the C(sp^2^)‐Bpin fragment. This attack proceeds through a 5‐membered ring transition state (TS2 see 3D structure in Figure 1b), overcoming a moderate free‐energy barrier of 18.9 kcal mol^−1^. In the resulting intermediate I5, the 5‐membered boracycle substrate is bonded to copper through a pinacol oxygen atom.

From I5, the reaction may proceed via two possible pathways: *i*) the migration of the Bpin moiety to C(sp^3^) along with the boracycle ring opening by cleavage of the B‐C(sp^2^) bond to give the corresponding Cu‐alkenyl complex I6, which can transmetallate with the KO*^t^* Bu base (dashed lines in Figure 1a); and *ii*) direct transmetallation of the KO*^t^* Bu base complex to the Cu‐boracyclic complex I5. In both pathways, the Cu‐O*^t^* Bu catalyst complex is recovered, and the resulting product is a five‐membered boracycle stabilized by a K^+^ counterion. Pathway *i* involves a free‐energy barrier of 16.6 kcal mol^−1^ (see Figure 1c for the corresponding transition state 3D‐structure TS3) and the transformation of intermediate I5 into I6 is energetically disfavoured by ≈5 kcal mol^−1^. On these grounds, and due to the large excess of KO^t^Bu in the reaction mixture, we suggest that the direct transmetallation pathway *ii* is more likely to form intermediate I7. Overall, the formation of boracycle species I7 from the Cu‐alkyl intermediate I4 is exergonic by 15.4 kcal mol^−1^, and the rate‐determining step of the whole mechanism is the first step of the 1,4‐B/Cu shift (I4 → TS2). Finally, the 1,4 migration is completed by the electrophilic trapping of boracycle I7 with I_2_ giving vinylic iodide product 14. This latter step was computed to be highly exergonic and provided the thermodynamic driving force for the reaction (see Figure 1a).

**Scheme 4:**
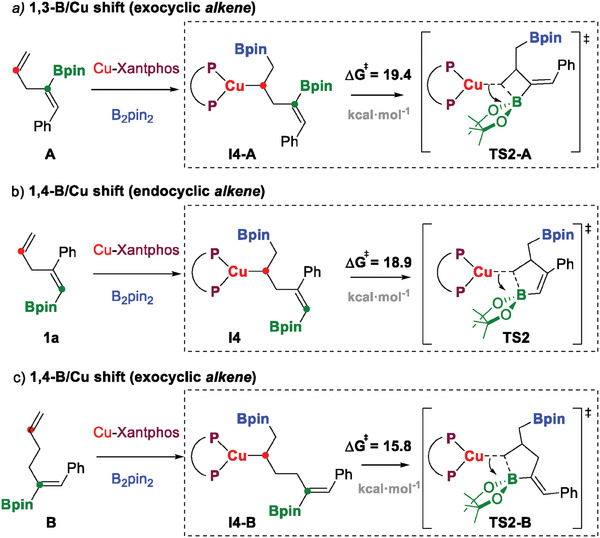
Free‐energy barriers (kcal mol^−1^) and transition state representations of the nucleophilic attack of the Cu‐alkyl moiety on the Bpin‐C(sp^2^) for different types of boryl migrations: a) 1,3‐B/Cu shift with an exocyclic alkene moiety on substrate A¸ b) 1,4‐B/Cu shift with an endocyclic alkene moiety on substrate 1a¸ and c) 1,4‐B/Cu shift with an exocyclic alkene moiety on substrate B.

To evaluate the impact of the alkene structure on the energy values of B/Cu shifts, in **Scheme** **4** we compare the free‐energy barriers, and the key transition state structures for three distinct Cu‐catalyzed, intramolecular boryl migrations on different substrates: a) the previously reported^[^ ^16^ ^]^ 1,3‐B/Cu shift on A¸ involving four‐membered boracyclic structures with an exocyclic alkene moiety; b) the 1,4‐B/Cu shift on 1a discussed here, involving five‐membered boracyclic structures with an endocyclic alkene; and c) the putative 1,4‐B/Cu shift on B, also involving five‐membered boracyclic structures, yet exhibiting an exocyclic alkene. The computed free‐energy barriers for the key Cu‐alkyl nucleophilic attack on the Bpin moiety bonded to the C(sp^2^) follow the order: A (1,3‐B/Cu *exo*) > 1a (1,4‐B/Cu‐*endo*) > B (1,4‐B/Cu‐*exo*), with values of 19.4, 18.9, and 15.8 kcal mol^−1^, respectively. Moving from the borylcupration of A to 1a, two opposite effects come into play that result in similar barriers.

On one hand, the 5‐membered ring transition state structure of the 1,4‐B/Cu shift in 1a is less strained than the 4‐membered ring of the 1,3‐B/Cu shift in A. Accordingly, the formation of the 5‐membered boracycle intermediate I5 from Cu‐alkyl intermediate I4 for 1a is thermodynamically favored by 5.7 kcal mol^−1^ (Figure 1a), while for A, the analogous process to form a 4‐membered boracycle is disfavored in terms of free‐energy by 10.4 kcal mol^−1^.^[^ ^16^ ^]^ On the other hand, the transition state for boryl migration in 1a involves an endocyclic alkene moiety, which destabilizes the structure as compared with the exocyclic placement of the alkene moiety during the 1,3‐boryl migration observed for substrate A (Scheme 4). We also evaluated the steric impact of borylated substrate substituents on the metal center at transition states TS2‐A and TS2 using the distance‐weighted volume (*V_W_*) parameter,^[^ ^22^ ^]^ but no differences were appreciated (see Supporting Information). Thus, when we moved to virtual substrate B, the transition state for the 1,4‐B/Cu shift involved a 5‐membered ring structure with the alkene moiety in an exocyclic position, which significantly decreased the free‐energy barrier (3.1 kcal mol^−1^) with respect to that computed for the endocyclic 1,4‐B/Cu shift of 1a.

**Scheme 5:**
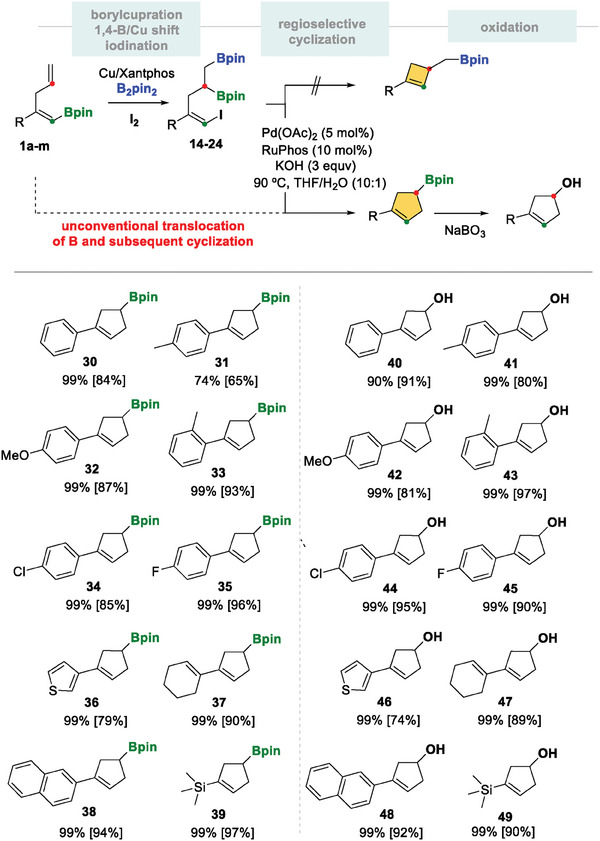
Regioselective intramolecular cross‐coupling of diborylated (*E*)‐iodo substituted alkene and the subsequent oxidation step. Reaction conditions: alkenes 14–24 (0.2 mmol), Pd(OAc)_2_ (5 mol%), RuPhos (10 mol%) KOH (0.6 mmol) were stirred in THF/H_2_O (2/0.2 mL) under an argon atmosphere at 90 °C for 16 h. *Oxidation* with NaBO_3_·H_2_O (0.3 mmol) in 2 ml THF and H_2_O (2 mL) for 16 h. NMR yield was calculated with naphthalene as the internal standard. Isolated yield in brackets.

The synthetic application of novel diborated iodoalkenyl products 14–24 was developed to prepare functionalized borylated cyclopentenes via a palladium‐catalyzed regioselective intramolecular cross‐coupling sequence. When compound 14 reacted with Pd(OAc)_2_/Ruphos,^[^ ^23^ ^]^ (Ruphos = dicuclohexyl(2′,6′‐diisopropoxy‐[1,1′‐biphenyl]−2‐yl)phosphine) a regioselective cyclization with the terminal Bpin moiety took place and exclusively generated the cyclopentene structure 30, retaining the original Bpin moiety from substrate 1a (**Scheme** **5**). This sequence shows us that the global reaction involves an unconventional remote translocation of B along boracycle intermediates that are intramolecularly cycled into valuable functionalized cyclopentene.^[^ ^24^ ^]^

The reaction is compatible with electron‐donating and electron‐withdrawing substituents in the *para* and *ortho* position of the aryl group (Scheme 5, products 31–35) as well as 3‐thienyl, cyclohexenyl and 2‐naphthyl groups (Scheme 5, products 36–38). Interestingly, the trimethylsilyl substituted (*E*)‐iodinated alkene 24 was efficiently transformed into the corresponding borylated/silylated cyclopentene 39 in an isolated yield of 97% (Scheme 5). The oxidation of the C‐B bond to borylated functionalized cyclopentenes provided facile access to the aryl‐ or vinyl cyclopentenyl alcohols 40–49, which are otherwise difficult to synthesize from Cu‐catalyzed arylation of cyclopentenes with diaryliodonium salts (Scheme 5).^[^ ^25^ ^]^ These 3‐arylcyclopent‐3‐en‐1‐ol products play a major role in the synthesis of a prostaglandin endoperoxide model system.^[^ ^26^ ^]^

**Scheme 6:**
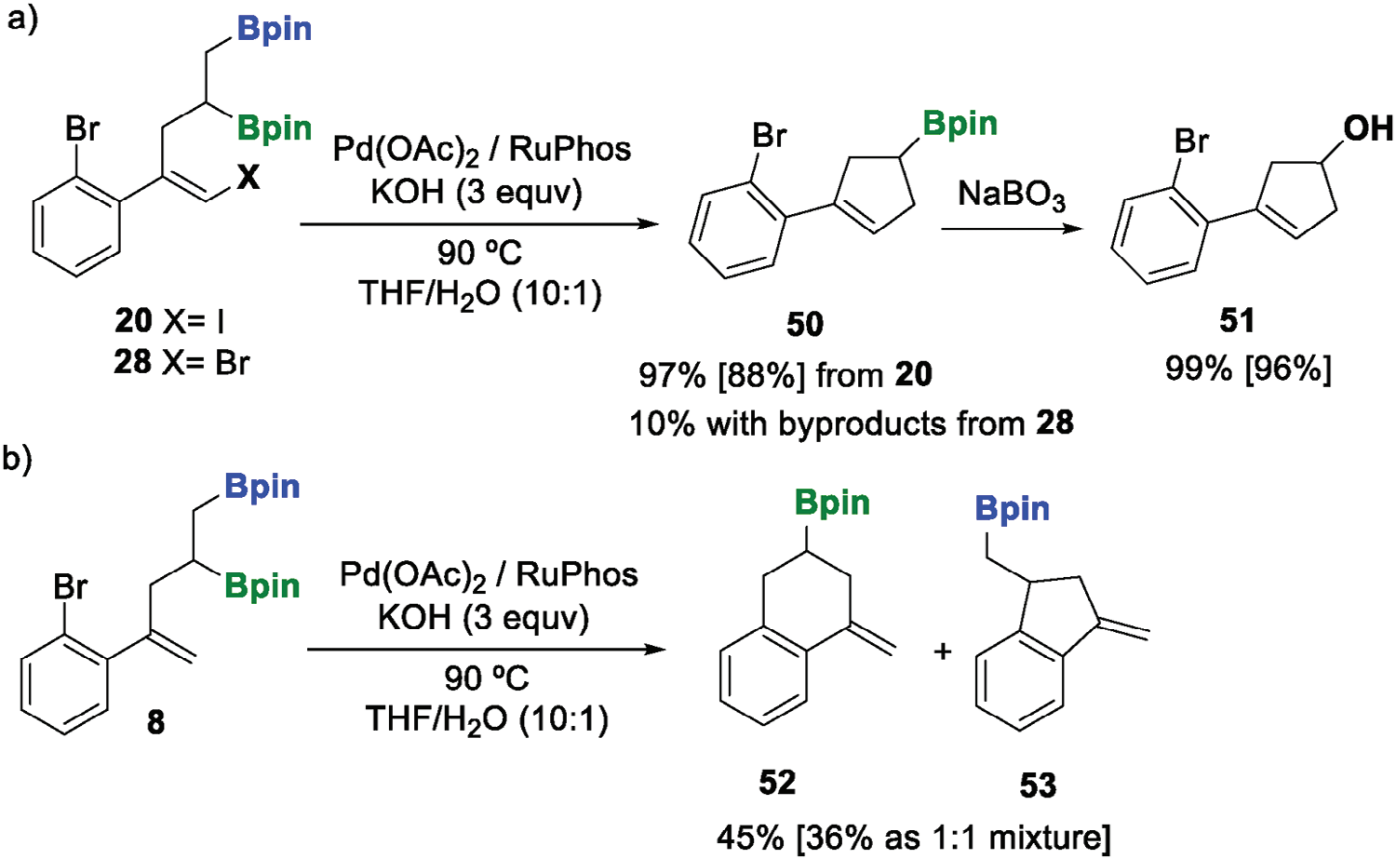
Comparative regioselective intramolecular competitive cross‐coupling reactions. Reaction conditions: alkenes 20, 28, or 8 (0.2 mmol), Pd(OAc)_2_ (5 mol%), RuPhos (10 mol%) KOH (0.6 mmol) were stirred in THF/H_2_O (2/0.2 mL) under an argon atmosphere at 90 °C for 16 h. Oxidation with NaBO_3_·H_2_O (0.3 mmol) in 2 mL THF and H_2_O (2 mL) for 16 h. NMR yield was calculated with naphthalene as the internal standard. Isolated yield in brackets.

We complemented this regioselective intramolecular cross‐coupling approach by exploring the reaction of diborylated (*E*)‐iodo substituted alkene 20, since four plausible cyclic compounds could have been formed. Gratifyingly, the reaction outcome proved to be regioselective since cyclopentene structure 50 was exclusively observed and isolated. The Bpin moiety originally in substrate 1 g was retained and the *ortho*‐Br‐C_6_H_4_ bond unaltered (**Scheme** **6**). The oxidation of product 50 gave the valuable functionalized cyclopentenol 51 in high yield (Scheme 6). As expected, the oxidative addition of C(sp^2^)‐I to the Pd complex is preferred to the oxidative addition of C(sp^2^)‐Br. However, to find more information about the exclusive formation of cyclopentene 50, we conducted the intramolecular cross‐coupling reaction with the analogous compound 28, which contains two C(sp^2^)‐Br bonds. Surprisingly the expected cyclopentene 50 was only detected in a yield of ≈10% together with other byproducts (Scheme 6). This confirms that the presence of C(sp^2^)‐I is crucial for this competitive intramolecular coupling. In addition, when product eight was submitted to the intramolecular cross‐coupling reaction conditions, an equative mixture of substituted tetrahydro naphthalene 52 and dihydro indene 53 was isolated in a 1:1 ratio (Scheme 6).

**Scheme 7:**
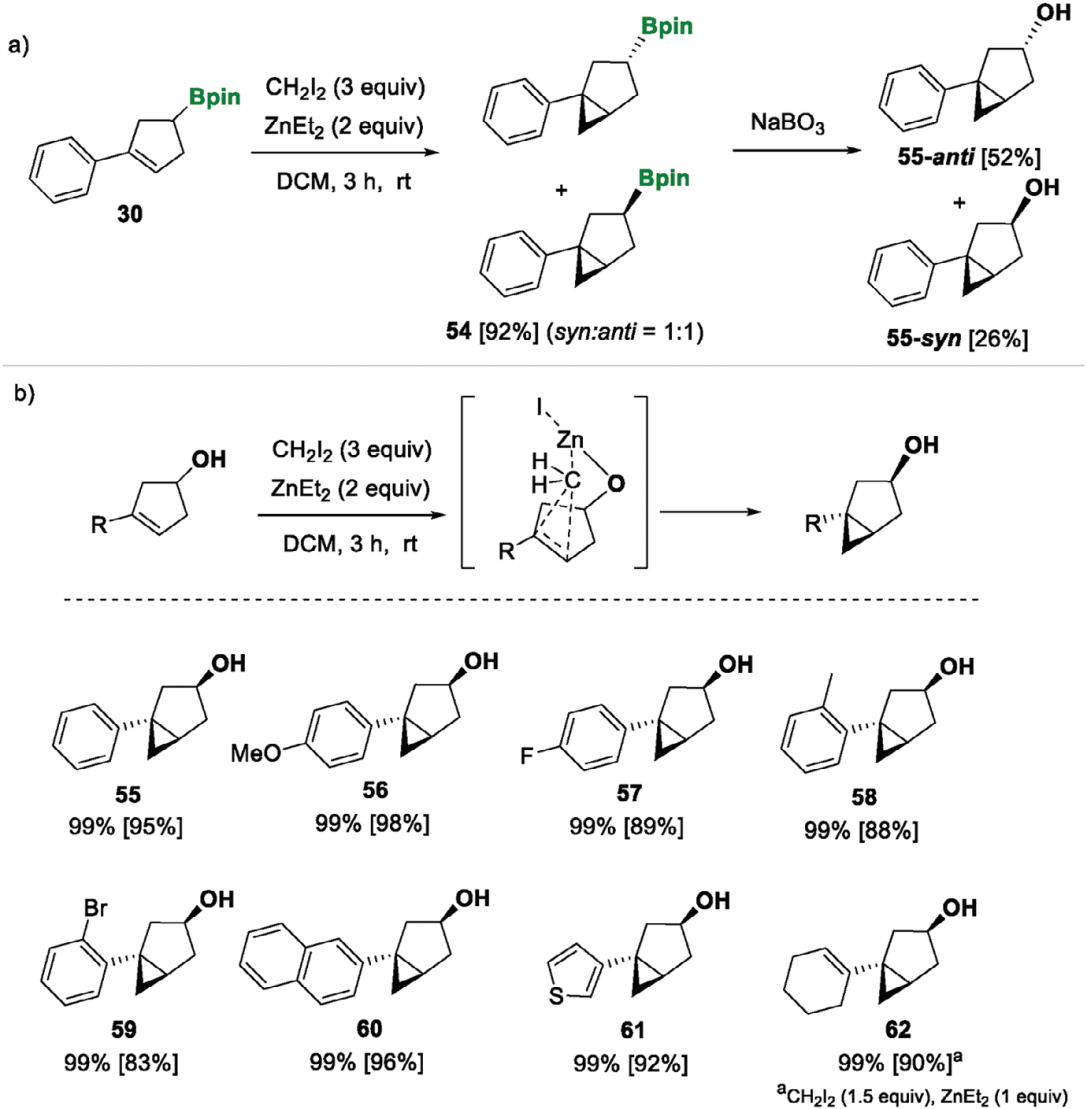
Stereoselective synthesis of bicycles via Simmons‐Smith cyclopropanation. Reaction conditions: cyclopentenes (0.2 mmol), ZnEt_2_ (0.4 mmol), CH_2_I_2_ (0.6 mmol) were stirred in DCM (2 mL) under an argon atmosphere for 3 h. NMR yield was calculated with naphthalene as the internal standard. Isolated yield in brackets.

Subsequently, we conducted the Simmons‐Smith cyclopropanation of the borylated cyclopentene 30, in order to find a convenient platform for constructing bicyclic systems, considered to be analogues of thujone^[^ ^27^ ^]^ (inhibitors of the γ‐aminobutyric acid A receptor). Under Furukawa conditions,^[^ ^28^ ^]^ the borylated bicycle 54 was afforded in quantitative yield as a 1:1 mixture of both diastereoisomers. The oxidation of 54 with NaBO_3_ proceeded smoothly, giving access to the corresponding alcohols 55‐syn and 55‐anti, which were isolated separately in an equative ratio (**Scheme** **7**a). However, when the substituted cyclopentenyl alcohol 40 reacted with diiodomethane and the zinc complex, the cyclopropanation proceeded diastereomerically, since zinc coordinates with the alcohol substituent and directs the cyclopropanation *cis* to the hydroxyl group. Following this synthetic strategy, compound 55 was isolated in a yield of 95% (Scheme 7b). The methodology was extended to include aryl groups with electron‐donating and electron‐withdrawing substituents in the *para* and *ortho* position (Scheme 7b, products 56–59) as well as 2‐naphthyl and 3‐thienyl groups (Scheme 5, products 60–61). Interestingly, when the substituted cyclohexenyl cyclopentenyl alcohol 47 reacted with CH_2_I_2_ (3 equiv) and ZnEt_2_ (2 equiv), both alkene groups led to the corresponding cyclopropanation, giving mixtures of the two diastereoisomers. However, when CH_2_I_2_ (1.5 equiv) and ZnEt_2_ (1 equiv) were used, compound 47 was converted into product 62 where only the double bond from the cyclopentenyl alcohol fragment was cyclopropanated, in a stereoselective manner, as expected (Scheme 7b).

## 3 Conclusion

In conclusion, we have developed a method for 1,4‐boryl copper shift without the assistance of a heteroatom via a remote 1,4‐carbon‐to‐carbon boryl migration. The borylcupration of borylated skipped (*E*)‐dienes, with CuCl/Xantphos, produced diborated terminal compounds and, to our surprise, that involved the Bpin migration from C_1_(sp^2^) to C_4_(sp^3^). This unprecedented remote carbon‐to‐carbon boryl migration takes place with stereospecificity around the alkene, contributing to the subsequent stereoselective electrophilic trapping by in situ addition of H^+^, I_2,_ or NBS. DFT calculations showed that the 1,4‐B/Cu shift occurs via nucleophilic attack of the copper‐alkyl moiety on the boron atom bonded to the C(sp^2^), leading to a 5‐membered boracycle structure. Then, the boracycle can be trapped electrophilically, completing the 1,4‐migration. The rate determining step corresponds to the intramolecular nucleophilic attack with a moderate, computed free‐energy barrier of 18.9 kcal mol^−1^, which is close to that computed for the 1,3‐B/Cu shift with an exo‐alkene moiety. This is the consequence of two opposite effects: 1) the 5‐membered ring transition state structure of the 1,4‐B/Cu shift is less strained than that of the 1,3‐B/Cu; and 2) the endo‐alkene configuration in the 1,4‐B/Cu shift destabilizes the cyclic structure. Thus, we predicted that the 1,4‐B/Cu shift involving an exo‐alkene moiety, instead of an endo‐alkene, would be even more favorable kinetically. Eventually, the synthetic application of the novel diborylated (*E*)‐iodo substituted alkene was developed to prepare functionalized cyclopentenes via a palladium‐catalyzed regioselective intramolecular coupling that was complemented by a strategic cyclopropanation to deliver stereoselective structural valuable bicyclic systems, via the OH‐directed Simmons‐Smith reaction.

## 4 Experimental Section

### General Procedure for the Synthesis of Borylated Dienes 1a‐1l

To a mixture of NaO*^t^* Bu (290 mg, 3 mmol), CuBr (30 mg, 0.2 mmol), and B_2_pin_2_ (762 mg, 3 mmol) in toluene (10 mL), added P*_n_* Bu_3_ (97 mg, 0.48 mmol), phenylacetylene (204 mg, 2 mmol), and allyl bromide (365 mg, 3 mmol) successively.^[^ ^17a^ ^]^ The resultant mixture was stirred at 80 °C for 36 h. The reaction was quenched with ethyl acetate and water and then extracted with ethyl acetate (3 × 10 mL). The combined organic layers were dried with anhydrous Na_2_SO_4_. After filtration, the filtrate was concentrated in a vacuum and the residue was purified by silica gel column chromatography (PE/EA = 40/1) to give the desired products.

### General Procedure for Cu‐Catalyzed Borylcupration/1,4‐B/Cu Migration/Protonation

CuCl (0.98 mg, 10 mol%, 0.01 mmol), bis(pinacolato)diboron (60.9 mg, 1.2 equiv, 0.24 mmol), and Xantphos (138.8 mg, 10 mol%, 0.01 mmol) were placed in an oven‐dried reaction vial. The vial was sealed with a screw cap containing a Teflon‐coated rubber septum. The vial was connected to a vacuum/nitrogen manifold through a needle, evacuated, and backfilled with nitrogen and THF (0.24 mL, 1 M). KO*^t^* Bu (26.9 mg, 1.2 equiv, 0.24 mmol) in THF (0.24 mL, 1 M) was added to the vial through the rubber septum. Then, the borylated (*E*) skipped dienes (1 equiv, 0.2 mmol) in THF (0.2 mL, 1 M) were added dropwise at 30 °C for 16 h. After the reaction was complete, 1 mL MeOH was added and stirred for 10 min and the reaction mixture was filtered over Celite. The organic extracts were then concentrated under vacuum and the NMR yield was calculated through comparison to an internal standard (naphthalene). The crude residue was purified by silica gel flash chromatography to obtain the desired product.

### General Procedure for Cu‐Catalyzed Borylcupration/1,4‐B/Cu Migration Followed by the Electrophilic Trapping with I_2_ or NBS

CuCl (0.98 mg, 10 mol%, 0.01 mmol), bis(pinacolato)diboron (60.9 mg, 1.2 equiv, 0.24 mmol), and Xantphos (138.8 mg, 10 mol%, 0.01 mmol) were placed in an oven‐dried reaction vial. The vial was sealed with a screw cap containing a Teflon‐coated rubber septum. The vial was connected to a vacuum/nitrogen manifold through a needle, evacuated, and backfilled with nitrogen and THF (0.24 mL, 1 m). KO*^t^* Bu (26.9 mg, 1.2 equiv, 0.24 mmol) in THF (0.24 mL, 1 m) was added to the vial through the rubber septum. Next, a solution of borylated (Z) skipped dienes (1 equivalent, 0.2 mmol) in 1 M THF (0.2 mL) was slowly added at 30 °C and stirred for 16 h. Afterward, the iodine (101.5 mg, 2 equiv, 0.4 mmol) was dissolved in 2 mL of THF in a second oven‐dried flask. The reaction mixture was kept at −60 °C while the iodine solution (0.4 mmol of iodine in 2 mL of THF) passed through a Teflon tubing. The cooling bath was removed, and the temperature was allowed to rise to room temperature. After 10 min at room temperature, a mixture of 10 mL of saturated aqueous ammonium chloride and 1 mL of saturated sodium bisulfite was added with vigorous stirring. The mixture was filtered through Celite by suction, and the funnel contents were washed with Et_2_O (3 × 15 mL). The inorganic layer was washed twice with pentane. The organic extracts were dried over anhydrous magnesium sulfate, filtered, and then concentrated under a vacuum. The NMR yield was calculated through comparison to an internal standard (naphthalene). The crude residue was purified by silica gel flash chromatography to obtain the desired product.

### General Procedure for Intramolecular Regioselective Suzuki‐Miyaura Cross‐Coupling

In a flamed Schlenk‐tube equipped with a magnetic stir bar, Pd(OAc)_2_ (2.2 mg, 5 mol%), RuPhos (9.3, 10 mol%), and the substrate (0.2 mmol) were added in THF (2 mL). Under an argon atmosphere, KOH (33.6 mg, 0.6 mmol, 3 equiv) and deoxygenated water (0.2 mL) were added. The reaction mixture was stirred at 90 °C for 16 h. After that, the mixture was filtered over Celite and concentrated under vacuum, and the NMR yield was calculated through comparison to an internal standard (naphthalene). The crude residue was purified by silica gel flash chromatography to obtain the desired product.

### General Procedure for Oxidation Reaction

In an open‐air flask, charged with a magnetic stir bar, added the corresponding cross‐coupling product (0.1 mmol, 1 equiv), NaBO_3_·H_2_O (0.3 mmol, 3 equiv), THF (2 mL), and distilled water (2 mL). The reaction was closed with a septum with a needle to avoid overpressure and was stirred for 16 h at room temperature. After this period of time, the mixture was extracted with Et_2_O (3 × 15 mL), the organic layer was dried with anhydrous magnesium sulfate, filtered over Celite, and the solvents were evaporated. The resulting crude was purified by silica gel chromatography to obtain the corresponding product.

### General Procedure for Simmons–Smith Cyclopropanation Reaction

To a solution of the oxidated product (0.2 mmol, 1.0 equiv) in anhydrous DCM (2.0 mL, 0.1 m) was added ZnEt_2_ (1.0 m in hexane, 0.4 mL, 0.4 mmol, 2.0 equiv) at 0 °C. After stirring for 10 min, CH_2_I_2_ (161 mg, 0.6 mmol, 3.0 equiv) was added. The mixture was stirred at room temperature and a white precipitate was gradually generated. After 3 h, the mixture was quenched with saturated aqueous NH_4_Cl (8 mL) and extracted with EtOAc (3 × 10 mL). The combined organic layers were dried with Na_2_SO_4_, filtered over Celite, and concentrated in vacuo. The residue was purified by flash column chromatography on silica gel (eluting with petroleum ether/ethyl acetate = 20/1) to give the corresponding product.

### Computational Details

Geometry optimizations, transition state searches, and energy evaluations were performed with Gaussian 16 package.^[^ ^29^ ^]^ The quantum mechanics calculations were performed within the framework of Density Functional Theory (DFT)^[^ ^30^ ^]^ by using the ωB97X‐D functional.^[^ ^31^ ^]^ For Cu, P, K, and I, effective core potentials (ECPs) with double‐ζ valence basis set (LANL2DZ) were employed,^[^ ^32^ ^]^ supplemented with polarized shells with the following exponents: Cu (f = 3.525), P (d = 0.387), K (d = 1.000) and I (d = 0.289).^[^ ^33^ ^]^ For all other atoms, the 6–31G(d) basis set was used.^[^ ^34^ ^]^ Solvent effects of THF were included using the implicit solvation model SMD.^[^ ^35^ ^]^ Free energies were computed at a concentration of 1 M  and a temperature of 298.15 K.

To quantify the steric hindrance of substrate substituents, the distance‐weighed volume parameter (*V_W_*),^[^ ^22^ ^]^ which measures the steric bulkiness of the molecular environment and its impact on the copper center was used. The descriptor quantifies the bulk produced by substrate substituents considering three parameters: 1) The number of atoms, excluding those forming the cyclic structures, 2) the size of the atom (*r* = van der Waals radii in Å), and 3) the distance (d) from the atom to the copper center (in Å). The factor *r^3^* was divided by d for each atom and the sum was extended to all the atoms in the given fragment, as given by the following equation:

(1) $$V_{W}=\sum_{i=1}^{N}\frac{r^{3}}{d_{i}}$$

In this case, the Cu center was placed at the origin. Then, the number of atoms on the substituents of substrates A and 1a, excluding those on the ring skeleton, and the distance from those atoms to the Cu center were considered. The analysed structures, TS2‐A and TS2, have the same value of *V_W_*. This indicates that the ligand‐substrate interactions have a minor effect on the free‐energy barrier differences, and they can be attributed to ring strain with two opposite effects.

## Conflict of Interest

The authors declare no conflict of interest.

## Supporting information

Supporting Information

## Data Availability

The data that support the findings of this study are available on request from the corresponding author. The data are not publicly available due to privacy or ethical restrictions.
