# Supplementary material for: Remote 1,4‐Carbon‐to‐Carbon Boryl Migration: From a Mechanistic Challenge to a Valuable Synthetic Application of Bicycles
Source: Adv Sci (Weinh). 2024 Feb 15;11(16):2309779. doi: 10.1002/advs.202309779 (PMC11040385; doi:10.1002/advs.202309779)
Supplement: Supplementary file 1 — Supporting Information [file ADVS-11-2309779-s001.pdf]

ADVANCED SCIENCE
Open Access

# Supporting Information

for *Adv. Sci.*, DOI 10.1002/advs.202309779

Remote 1,4-Carbon-to-Carbon Boryl Migration: From a Mechanistic Challenge to a Valuable Synthetic Application of Bicycles

*Paula Dominguez-Molano, Albert Solé-Daura, Jorge J. Carbó* and Elena Fernández**

# Remote 1,4-Carbon-to-Carbon Boryl Migration: from a Mechanistic Challenge to a Valuable Synthetic Application of Bicycles

Paula Dominguez-Molano, Albert Solé-Daura, Jorge J. Carbó,* Elena Fernández.*

Dept. Química Física i Inorgànica, University Rovira i Virgili, 43007 Tarragona, Spain.

## Contents:

- General Information S2
- General procedure for the synthesis of borylated dienes **1a-1l** S2
- General procedure for Cu-catalyzed borylcupration/1,4-B/Cu migration/ Protonation S3
- General procedure for Cu-catalyzed borylcupration/1,4-B/Cu migration followed by electrophilic trapping with $I_2$ or NBS S3
- General procedure for intramolecular regioselective Suzuki-Miyaura cross-coupling S4
- General procedure for oxidation reaction S4
- General procedure for Simmons–Smith cyclopropanation reaction S4
- Characterization data for borylated dienes S5
- Characterization data for products isolated from Cu-catalyzed borylcupration /1,4-B/Cu migration/protonation (or iodination or bromination) S6
- Characterization data for cross-coupling products and corresponding alcohols after oxidation S17
- Characterization data for bicycles S24
- $^{1}H$, $^{13}C$ and $^{11}B$ Spectra of borylated dienes S28
- $^{1}H$, $^{13}C$, $^{11}B$ Spectra for products isolated from Cu-catalyzed borylcupration /1,4-B/Cu migration/ protonation (or iodination or bromination) S33
- $^{1}H$, $^{13}C$, $^{11}B$ Spectra for cross-coupling products and corresponding alcohols S77
- $^{1}H$, $^{13}C$ Spectra for bicycle products S107
- Computational Details S118
- References S151

## -General Information

*Solvents and reagents*: Solvents and reagents were obtained from commercial suppliers and dried and/or purified (if needed) by standard procedures. Diboron reagents were purchased from Ally Chem and used without further purification. All reactions were conducted in an oven and flame-dried glassware under an inert atmosphere of argon, using Schlenk-type techniques. *Flash chromatography* was performed on standard silica gel (Merck Kieselgel 60 F254 400-630 mesh). *Thin layer chromatography* was performed on Merck Kieselgel 60 F254 which was developed using standard visualizing agents: UV fluorescence (254 and 366 nm) or potassium permanganate/Δ. *NMR spectra* were recorded at a Varian Goku 400 or a Varian Mercury 400 spectrometer. $^{1}$H NMR and $^{13}$C{$^{1}$H} NMR chemical shifts (δ) are reported in ppm with the solvent resonance as the internal standard ($CHCl_3$: 7.26 ppm ($^{1}$H)) and ($CDCl_3$: 77.16 ppm ($^{13}$C). $^{11}$B{$^{1}$H} NMR chemical shifts (δ) are reported in ppm relative to $(CH_3)_2O{\cdots}BF_3$. Data are reported as follows: chemical shift, multiplicity (s = singlet, d = doublet, t = triplet, q = quartet, hept = septuplet, br = broad, m = multiplet), coupling constants (Hz), and integration. *High-resolution mass spectra (HRMS)* were recorded using a 6210 Time of Flight (TOF) mass spectrometer from Agilent Technologies (Waldbronn, Germany) with an ESI interface and it was performed at the Servei de Recursos Científics i Tècnics (Universitat Rovira i Virgili, Tarragona) or using a BIOTOF II Time of Flight (TOF) mass spectrometer from Bruker with an APCI interface or EI interface and it was performed at the Unidade de Espectrometría de Masas e Proteómica (Universidade de Santiago de Compostela, Santiago de Compostela). GC-MS analyses were performed on an HP6890 gas chromatograph, and an Agilent Technologies 5973 Mass selective detector (Waldbronn, Germany) equipped with an achiral capillary column HP-5 (30m, 0.25mm i. d., 0.25μm thickness) using He as the carrier gas.

## - General procedure for the synthesis of borylated dienes 1a-1l [1]

To a mixture of NaO$^{t}$Bu (290 mg, 3 mmol), CuBr (30 mg, 0.2 mmol), and $B_2pin_2$ (762 mg, 3 mmol) in toluene (10 ml) were added $P_nBu_3$ (97 mg, 0.48 mmol), phenylacetylene (204 mg, 2 mmol), and allyl bromide (365 mg, 3 mmol) successively. The resultant mixture was stirred at 80 °C for 36 hrs. The reaction was quenched with ethyl acetate and water, and then extracted with ethyl acetate (3 x 10 mL). The combined organic layers were dried with anhydrous $Na_2SO_4$. After filtration, the filtrate was concentrated in vacuum and the residue was purified by silica gel column chromatography (PE/EA = 40/1) to give the desired products.

## -General procedure for Cu-catalyzed borylcupration/1,4-B/Cu migration/protonation[2]

CuCl (0.98 mg, 10 mol%, 0.01 mmol), bis(pinacolato)diboron (60.9 mg, 1.2 equiv, 0.24 mmol) and Xantphos (138.8 mg, 10 mol%, 0.01 mmol) were placed in an oven-dried reaction vial. The vial was sealed with a screw cap containing a Teflon-coated rubber septum. The vial was connected to a vacuum/nitrogen manifold through a needle, evacuated, and backfilled with nitrogen and THF (0.24 ml, 1 M). KO$^t$Bu (26.9 mg, 1.2 equiv, 0.24 mmol) in THF (0.24 ml, 1 M) were added in the vial through the rubber septum. Then, the borylated (*E*) skipped dienes (1 equiv, 0.2 mmol) in THF (0.2 ml, 1 M) were added dropwise at 30 °C for 16 hours. After the reaction was complete, 1 mL MeOH was added and stirred for 10 min and the reaction mixture was filtered over Celite. The organic extracts were then concentrated under vacuum and the NMR yield was calculated through comparison to an internal standard (naphthalene). The crude residue was purified by silica gel flash chromatography to obtain the desired product.

## -General procedure for Cu-catalyzed borylcupration/1,4-B/Cu migration followed by the electrophilic trapping with $I_2$ or NBS

CuCl (0.98 mg, 10 mol%, 0.01 mmol), bis(pinacolato)diboron (60.9 mg, 1.2 equiv, 0.24 mmol) and Xantphos (138.8 mg, 10 mol%, 0.01 mmol) were placed in an oven-dried reaction vial. The vial was sealed with a screw cap containing a Teflon-coated rubber septum. The vial was connected to a vacuum/nitrogen manifold through a needle, evacuated, and backfilled with nitrogen and THF (0.24 ml, 1 M). KO$^t$Bu (26.9 mg, 1.2 equiv, 0.24 mmol) in THF (0.24 ml, 1 M) was added in the vial through the rubber septum. Next, a solution of borylated (Z) skipped dienes (1 equivalent, 0.2 mmol) in 1 M THF (0.2 ml) was slowly added at 30 °C and stirred for 16 hours.

Afterward, the iodine (101.5 mg, 2 equiv, 0.4 mmol) was dissolved in 2 mL of THF in a second oven-dried flask. The reaction mixture was kept at -60 °C while the iodine solution (0.4 mmol of iodine in 2 mL of THF) passed through a Teflon tubing. The cooling bath was removed, and the temperature was allowed to rise to room temperature. After 10 minutes at room temperature, a mixture of 10 mL of saturated aqueous ammonium chloride and 1 mL of saturated sodium bisulfite is added with vigorous stirring. The mixture was filtered through Celite by suction, and the funnel contents were washed with $Et_2O$ (3 x 15 mL). The inorganic layer is washed twice with pentane. The organic extracts were dried over anhydrous magnesium sulfate and then concentrated under a vacuum. The NMR yield was calculated through comparison to an internal standard (naphthalene). The crude residue was purified by silica gel flash chromatography to obtain the desired product.

## -General procedure for intramolecular regioselective Suzuki-Miyaura cross-coupling

In a flamed Schlenk-tube equipped with a magnetic stir bar, $Pd(OAc)_2$ (2.2 mg, 5 mol%), RuPhos (9.3, 10 mol%), and the substrate (0.2 mmol) were added in THF (2 mL). Under an argon atmosphere, KOH (33.6 mg, 0.6 mmol, 3 equiv) and deoxygenated water (0.2 mL) were added. The reaction mixture was stirred at 90 °C for 16 h. After that, the mixture was filtered through Celite and concentrated under vacuum, and the NMR yield was calculated through comparison to an internal standard (naphthalene). The crude residue was purified by silica gel flash chromatography to obtain the desired product.

## -General procedure for oxidation reaction

In an open-air flask, charged with a magnetic stir bar, were added the corresponding cross-coupling product (0.1 mmol, 1 equiv), $NaBO_3 \cdot H_2O$ (0.3 mmol, 3 equiv), THF (2 mL) and distilled water (2 mL). The reaction was closed with a septum with a needle to avoid over pressure and was stirred for 16 h at room temperature. After this period of time, the mixture was extracted with $Et_2O$ (3 x 15 mL), the organic layer was dried with anhydrous magnesium sulfate, filtered through Celite and the solvents were evaporated. The resulting crude was purified by silica gel chromatography to obtain the corresponding product.

## - General procedure for Simmons–Smith cyclopropanation reaction[3]

To a solution of the oxidated product (0.2 mmol, 1.0 equiv) in anhydrous DCM (2.0 mL, 0.1 M) was added $ZnEt_2$ (1.0 M in hexane, 0.4 mL, 0.4 mmol, 2.0 equiv) at 0 °C. After stirring for 10 min, $CH_2I_2$ (161 mg, 0.6 mmol, 3.0 equiv) was added. The mixture was stirred at room temperature and a white precipitate was gradually generated. After 3 hours, the mixture was quenched with saturated aqueous $NH_4Cl$ (8 mL) and extracted with EtOAc (3 x 10 mL). The combined organic layers were dried with $Na_2SO_4$, filtered through Celite and concentrated in vacuo. The residue was purified by flash column chromatography on silica gel (eluting with petroleum ether/ethyl acetate = 20/1) to give the corresponding product.

## - Characterization data for borylated dienes

Characterization of compounds **1a**-**1i** was in agreement with reported literature.[1]

**(*E*)-2-(2-(cyclohex-1-en-1-yl)penta-1,4-dien-1-yl)-4,4,5,5-tetramethyl-1,3,2-dioxaborolane (1j)**

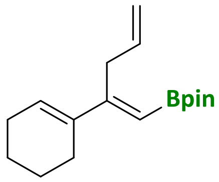


The product was purified by flash chromatography using as eluent a mixture of petroleum ether/diethyl ether (40:1). The product was isolated as a colorless oil (50 mg, 90%, >50:1).

$^{1}$H NMR (CDCl$_3$, 400 MHz) δ 6.12 (bs, 1H), 5.84 (dt, J = 16.7, 10.0 Hz, 1H), 5.39 (s, 1H), 5.06 (dd, J = 17.1, 1.8 Hz, 1H), 4.92 (dd, J = 10.1, 1.6 Hz, 1H), 3.43 (dt, J = 6.5, 1.6 Hz, 2H), 2.17 (bs, 4H), 1.64 (m 2H), 1.55 (m 2H), 1.26 (s, 12H).

$^{13}$C NMR (CDCl$_3$, 100 MHz) δ 160.2, 138.6, 137.3, 128.0, 114.5, 82.7, 35.8, 26.2, 26.0, 24.8, 23.0, 22.0.

$^{11}$B NMR (CDCl$_3$, 128.3 MHz) δ 30.1.

HRMS-(ESI+) for $C_{17}H_{28}BO_2$ $[M+H]^+$: calculated: 275.2020; found: 275.2019.

**(*E*)-trimethyl(1-(4,4,5,5-tetramethyl-1,3,2-dioxaborolan-2-yl)penta-1,4-dien-2-yl)silane (1k)**

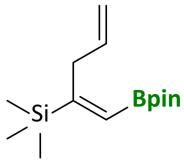


The product was purified by flash chromatography using as eluent a mixture of petroleum ether/diethyl ether (40:1). The product was isolated as a colorless oil (30 mg, 55%, >50:1).

$^{1}$H NMR (CDCl$_3$, 400 MHz) δ 5.92 (s, 1H), 5.77 (dt, J = 16.8, 10.0 Hz, 1H), 5.02 (dd, J = 17.1, 1.8 Hz, 1H), 4.97 – 4.89 (m, 1H), 3.29 (dq, J = 6.7, 1.3 Hz, 2H), 1.26 (s, 12H), 0.07 (s, 9H).

$^{13}$C NMR (CDCl$_3$, 100 MHz) δ 169.6, 139.6, 116.0, 84.1, 40.3, 26.1, -0.0.

$^{11}$B NMR (CDCl$_3$, 128.3 MHz) δ 28.8.

HRMS-(ESI+) for $C_{14}H_{28}BO_2Si$ $[M+H]^+$: calculated: 267.1946; found: 267.1940.

**(Z)-4,4,5,5-tetramethyl-2-(penta-1,4-dien-1-yl)-1,3,2-dioxaborolane (1l)**

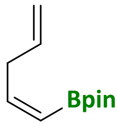


The product was purified by flash chromatography using as eluent a mixture of petroleum ether/diethyl ether (40:1). The product was isolated as a colorless oil (38 mg, 98%, >50:1).

$^{1}$H NMR (CDCl$_3$, 400 MHz) δ 6.42 (m, 1H), 5.83 (dt, *J* = 16.7, 10.1 Hz, 1H), 5.40 (dt, *J* = 13.4, 1.4 Hz, 1H), 5.05 (dd, *J* = 17.2, 1.7 Hz, 1H), 4.98 (dd, *J* = 10.1, 1.6 Hz, 1H), 3.16 (dt, *J* = 6.5, 1.5 Hz, 2H), 1.26 (s, 12H).

$^{13}$C NMR (CDCl$_3$, 100 MHz) δ 151.6, 136.9, 115.0, 82.9, 36.6, 24.8.

$^{11}$B NMR (CDCl$_3$, 128.3 MHz) δ 29.6.

HRMS-(ESI+) for $C_{11}H_{20}BO_2$ $[M+H]^+$: calculated: 195.1557; found: 195.1559.

## - Characterization data for products isolated from Cu-catalyzed borylcupration/1,4-B/Cu migration/protonation (or iodination or bromination)

**2,2'-(4-phenylpent-4-ene-1,2-diyl)bis(4,4,5,5-tetramethyl-1,3,2-dioxaborolane) (2)**

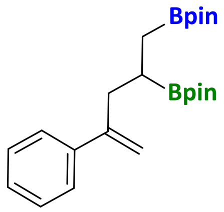


The product was purified by flash chromatography using as eluent a mixture of petroleum ether/diethyl ether (100:5). The product was isolated as a colorless oil (77 mg, 97%).

$^{1}$H NMR (CDCl$_3$, 400 MHz) δ 7.43 – 7.35 (m, 2H), 7.32 – 7.09 (m, 3H), 5.22 (d, J = 1.7 Hz, 1H), 5.04 (d, J = 1.4 Hz, 1H), 2.71 (ddd, J = 14.5, 7.6, 1.2 Hz, 1H), 2.46 (ddd, J = 14.4, 8.1, 1.1 Hz, 1H), 1.19 (s, 6H), 1.19 (s, 6H), 1.18 (s, 6H), 1.18 (s, 6H), 1.18 (m, 1H), 0.82 (m, 2H).

$^{13}$C NMR (CDCl$_3$, 100 MHz) δ 148.2, 141.5, 128.0, 127.0, 126.4, 113.0, 82.8, 82.8, 38.8, 24.8, 24.8, 24.8, 24.7.

$^{11}$B NMR (CDCl$_3$, 128.3 MHz) δ 33.5.

HRMS-(ESI+) for $C_{23}H_{37}B_2O_4$ [M+H]$^+$: calculated: 399.2872; found: 399.2891.

**2,2'-(4-(p-tolyl)pent-4-ene-1,2-diyl)bis(4,4,5,5-tetramethyl-1,3,2-dioxaborolane) (3)**

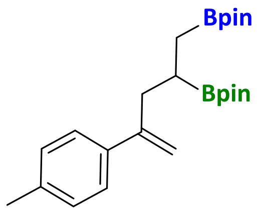


The product was purified by flash chromatography using as eluent a mixture of petroleum ether/diethyl ether (100:5). The product was isolated as a colorless oil (75 mg, 91%).

$^{1}$H NMR (CDCl$_3$, 400 MHz) δ 7.32 (d, J = 8.2 Hz, 2H), 7.13 – 7.06 (m, 2H), 5.22 (d, J = 1.7 Hz, 1H), 5.01 (d, J = 1.5 Hz, 1H), 2.71 (ddd, J = 14.5, 7.6, 1.2 Hz, 1H), 2.51 – 2.41 (m, 1H), 2.32 (s, 3H), 1.22 (s, 6H), 1.22 (s, 6H), 1.21 (s, 6H), 1.21 (s, 6H), 1.21 (m, 1H), 0.84 (m, 2H).

$^{13}$C NMR (CDCl$_3$, 100 MHz) δ 148.0, 138.5, 136.7, 128.8, 126.2, 112.2, 82.8, 82.8, 38.8, 24.8, 24.8, 24.8, 24.7, 21.0.

$^{11}$B NMR (CDCl$_3$, 128.3 MHz) δ 33.3.

HRMS-(ESI+) for $C_{24}H_{39}B_2O_4$ [M+H]$^+$: calculated: 413.3036; found: 413.3038.

**2,2'-(4-(4-methoxyphenyl)pent-4-ene-1,2-diyl)bis(4,4,5,5-tetramethyl-1,3,2-dioxaborolane) (4)**

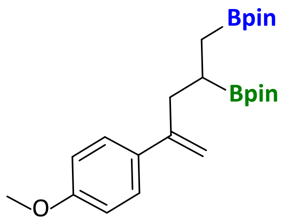


The product was purified by flash chromatography using as eluent a mixture of petroleum ether/diethyl ether (100:5). The product was isolated as a colorless oil (84 mg, 98%).

$^{1}$H NMR (CDCl$_3$, 400 MHz) δ 7.40 – 7.32 (m, 2H), 6.86 – 6.77 (m, 2H), 5.18 (d, J = 1.7 Hz, 1H), 4.98 (s, 1H), 3.79 (s, 3H), 2.70 (ddd, J = 14.4, 7.6, 1.2 Hz, 1H), 2.44 (ddd, J = 14.4, 8.1, 1.0 Hz, 1H), 1.21 (m, 1H), 1.21 (s, 6H), 1.21 (s, 6H), 1.20 (s, 12H), 0.84 (m, 2H).

$^{13}C$ NMR ($CDCl_3$, 100 MHz) δ 158.8, 147.4, 133.9, 127.4, 113.4, 111.5, 82.8, 82.8, 55.2, 38.9, 24.8, 24.8, 24.8, 24.7, 17.0 (C–B), 13.0 (C–B).

$^{11}B$ NMR ($CDCl_3$, 128.3 MHz) δ 34.1.

HRMS-(ESI+) for $C_{24}H_{39}B_2O_5$ $[M+H]^+$: calculated: 429.2984; found: 429.2978.

**2,2'-(4-(4-chlorophenyl)pent-4-ene-1,2-diyl)bis(4,4,5,5-tetramethyl-1,3,2-dioxaborolane) (5)**

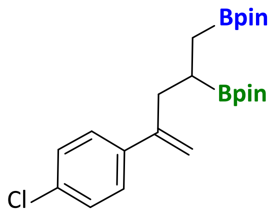


The product was purified by flash chromatography using as eluent a mixture of petroleum ether/diethyl ether (100:5). The product was isolated as a colorless oil (69 mg, 80%).

$^{1}H$ NMR ($CDCl_3$, 400 MHz) δ 7.38 – 7.31 (m, 2H), 7.22 – 7.10 (m, 2H), 5.23 (d, J = 1.5 Hz, 1H), 5.07 (d, J = 1.6 Hz, 1H), 2.68 (ddd, J = 14.4, 7.7, 1.2 Hz, 1H), 2.51 – 2.40 (m, 1H), 1.22 (s, 12H), 1.20 (s, 12H), 1.20 (m, 1H), 0.86 – 0.77 (m, 2H).

$^{13}C$ NMR ($CDCl_3$, 100 MHz) δ 147.2, 139.9, 132.8, 128.2, 127.7, 113.5, 82.9, 82.9, 38.7, 24.8, 24.8, 24.8, 24.7.

$^{11}B$ NMR ($CDCl_3$, 128.3 MHz) δ 34.3.

HRMS-(ESI+) for $C_{23}H_{36}B_2ClO_4$ $[M+H]^+$: calculated: 433.2483; found: 433.2504.

**2,2'-(4-(4-fluorophenyl)pent-4-ene-1,2-diyl)bis(4,4,5,5-tetramethyl-1,3,2-dioxaborolane) (6)**

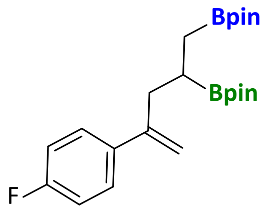


The product was purified by flash chromatography using as eluent a mixture of petroleum ether/diethyl ether (100:5). The product was isolated as a colorless oil (78 mg, 94%).

$^{1}H$ NMR ($CDCl_3$, 400 MHz) δ 7.43 – 7.32 (m, 2H), 6.97 (d, J = 8.8 Hz, 1H), 6.95 (d, J = 8.7 Hz, 1H), 5.18 (d, J = 1.6 Hz, 1H), 5.04 (d, J = 1.5 Hz, 1H), 2.69 (ddd, J = 14.4, 7.7, 1.2 Hz, 1H), 2.45 (ddd, J = 14.4, 8.0, 1.1 Hz, 1H), 1.21 (s, 12H), 1.20 (s, 12H), 1.20 (m, 1H), 0.83 (m, 2H).

$^{13}C$ NMR ($CDCl_3$, 100 MHz) δ 162.1 (d, $^{1}J_{C-F}$ = 245.5 Hz), 147.2, 137.5, 137.4, 127.9 (d, $^{3}J_{C-F}$ = 7.9 Hz), 114.8 (d, $^{2}J_{C-F}$ = 21.1 Hz), 113.0, 112.9, 82.9, 82.8, 38.9, 24.8, 24.8, 24.8, 24.7, 16.9(C-B), 11.9(C-B).

$^{11}B$ NMR ($CDCl_3$, 128.3 MHz) δ 33.5.

$^{19}F$ NMR ($CDCl_3$, 377 MHz) δ -116.05.

HRMS-(ESI+) for $C_{23}H_{36}B_2FO_4$ $[M+H]^+$: calculated: 417.2778; found: 417.2787.

**2,2'-(4-(o-tolyl)pent-4-ene-1,2-diyl)bis(4,4,5,5-tetramethyl-1,3,2-dioxaborolane) (7)**

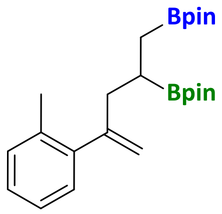


The product was purified by flash chromatography using as eluent a mixture of petroleum ether/diethyl ether (100:5). The product was isolated as a colorless oil (79 mg, 96%).

$^{1}$H NMR ($CDCl_3$, 400 MHz) δ 7.18 – 7.06 (m, 4H), 5.17 (d, J = 1.6 Hz, 1H), 4.85 (d, J = 2.1 Hz, 1H), 2.59 – 2.49 (m, 1H), 2.34 (m, 1H), 2.30 (s, 3H), 1.31 – 1.26 (m, 1H), 1.22 (s, 12H), 1.21 (s, 12H), 0.93 – 0.89 (m, 2H).

$^{13}$C NMR ($CDCl_3$, 100 MHz) δ 149.7, 143.4, 134.9, 129.9, 128.5, 126.5, 125.2, 114.0, 82.8, 82.8, 41.1, 24.8, 24.8.

$^{11}$B NMR ($CDCl_3$, 128.3 MHz) δ 33.6.

HRMS-(ESI+) for $C_{24}H_{39}B_2O_4$ $[M+H]^+$: calculated: 413.3036; found: 413.3029.

**2,2'-(4-(2-bromophenyl)pent-4-ene-1,2-diyl)bis(4,4,5,5-tetramethyl-1,3,2-dioxaborolane) (8)**

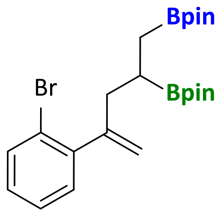


The product was purified by flash chromatography using as eluent a mixture of petroleum ether/diethyl ether (100:5). The product was isolated as a colorless oil (73 mg, 77%).

$^{1}$H NMR ($CDCl_3$, 400 MHz) δ 7.57 (dd, J = 8.0, 1.3 Hz, 1H), 7.30 – 7.22 (m, 2H), 7.18 – 7.09 (m, 1H), 5.29 (d, J = 1.6 Hz, 1H), 5.01 (d, J = 1.8 Hz, 1H), 2.74 (ddd, J = 14.8, 8.0, 1.1 Hz, 1H), 2.47 (ddd, J = 14.8, 8.0, 1.1 Hz, 1H), 1.28 (s, 12H), 1.28 (m, 1H), 1.28 (s, 12H), 1.03 – 0.92 (m, 2H).

$^{13}$C NMR ($CDCl_3$, 100 MHz) δ 149.4, 144.2, 132.5, 130.5, 128.1, 126.9, 122.0, 115.6, 82.9, 82.8, 40.3, 24.8, 24.8, 24.8, 24.7, 16.9(C-B), 11.7(C-B).

$^{11}$B NMR ($CDCl_3$, 128.3 MHz) δ 33.6.

HRMS-(ESI+) for $C_{23}H_{36}B_2BrO_4$ $[M+H]^+$: calculated: 477.1978; found: 477.1973.

**2,2'-(4-(naphthalen-2-yl)pent-4-ene-1,2-diyl)bis(4,4,5,5-tetramethyl-1,3,2-dioxaborolane) (9)**

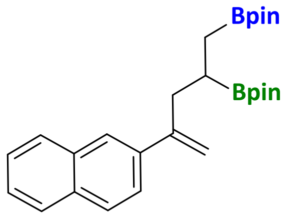


The product was purified by flash chromatography using as eluent a mixture of petroleum ether/diethyl ether (100:5). The product was isolated as a colorless oil (81 mg, 90%).

$^{1}$H NMR ($CDCl_3$, 400 MHz) δ 7.87 – 7.73 (m, 4H), 7.59 (dd, J = 8.6, 1.8 Hz, 1H), 7.47 – 7.38 (m, 2H), 5.39 (d, J = 1.6 Hz, 1H), 5.17 (d, J = 1.4 Hz, 1H), 2.85 (ddd, J = 14.5, 7.7, 1.2 Hz, 1H), 2.60 (ddd, J = 14.4, 8.1, 1.1 Hz, 1H), 1.22 (s, 6H), 1.21 (s, 6H), 1.20 (s, 6H), 1.20 (s, 6H), 1.20 (m, 1H), 0.89 (m, 2H).

$^{13}C$ NMR ($CDCl_3$, 100 MHz) δ 148.1, 138.8, 133.4, 132.7, 128.2, 127.5, 127.4, 125.8, 125.5, 125.1, 124.9, 113.6, 82.9, 82.8, 38.8, 24.8, 24.8, 24.8, 24.7.

$^{11}B$ NMR ($CDCl_3$, 128.3 MHz) δ 34.7.

HRMS-(ESI+) for $C_{27}H_{39}B_2O_4$ $[M+H]^+$: calculated: 449.3032; found: 449.3029.

**2,2'-(4-(thiophen-3-yl)pent-4-ene-1,2-diyl)bis(4,4,5,5-tetramethyl-1,3,2-dioxaborolane) (10)**

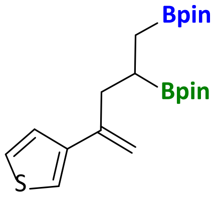


The product was purified by flash chromatography using as eluent a mixture of petroleum ether/diethyl ether (100:5). The product was isolated as a colorless oil (68 mg, 84%).

$^{1}H$ NMR ($CDCl_3$, 400 MHz) δ 7.32 – 7.25 (m, 1H), 7.22 (qd, J = 5.0, 2.1 Hz, 2H), 5.33 (d, J = 1.5 Hz, 1H), 5.01 (d, J = 1.3 Hz, 1H), 2.67 (ddd, J = 14.1, 7.7, 1.2 Hz, 1H), 2.40 (ddd, J = 14.2, 7.9, 1.1 Hz, 1H), 1.47 – 1.37 (m, 1H), 1.22 (s, 12H), 1.21 (s, 12H), 0.96 – 0.79 (m, 2H).

$^{13}C$ NMR ($CDCl_3$, 100 MHz) δ 142.6, 142.5, 126.0, 124.9, 120.6, 111.6, 82.9, 39.0, 24.8, 24.8, 24.7.

$^{11}B$ NMR ($CDCl_3$, 128.3 MHz) δ 34.1.

HRMS-(ESI+) for $C_{23}H_{37}B_2O_4$ $[M+H]^+$: calculated: 399.2878; found: 399.2889.

**2,2'-(4-(cyclohex-1-en-1-yl)pent-4-ene-1,2-diyl)bis(4,4,5,5-tetramethyl-1,3,2-dioxaborolane) (11)**

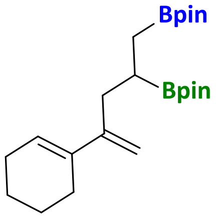


The product was purified by flash chromatography using as eluent a mixture of petroleum ether/diethyl ether (100:5). The product was isolated as a colorless oil (76 mg, 95%).

$^{1}H$ NMR ($CDCl_3$, 400 MHz) δ 5.92 (m, 1H), 4.92 (d, J = 1.8 Hz, 1H), 4.79 (d, J = 1.7 Hz, 1H), 2.45 (ddd, J = 14.0, 8.0, 1.0 Hz, 1H), 2.24 – 2.15 (m, 1H), 2.13 – 2.07 (m, 4H), 1.70 – 1.58 (m, 2H), 1.54 (m, 2H), 1.38 – 1.28 (m, 1H), 1.22 (s, 12H), 1.21 (s, 12H), 0.85 – 0.77 (m, 2H).

$^{13}C$ NMR ($CDCl_3$, 100 MHz) δ 148.1, 135.5, 124.3, 109.4, 82.8, 82.7, 37.4, 26.0, 25.8, 24.9, 24.8, 24.8, 24.7, 22.9, 22.2.

$^{11}B$ NMR ($CDCl_3$, 128.3 MHz) δ 33.6.

HRMS-(ESI+) for $C_{23}H_{41}B_2O_4$ $[M+H]^+$: calculated: 403.3191; found: 403.3205.

**(4,5-bis(4,4,5,5-tetramethyl-1,3,2-dioxaborolan-2-yl)pent-1-en-2-yl)trimethylsilane (12)**

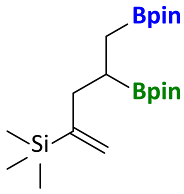


The product was purified by flash chromatography using as eluent a mixture of petroleum ether/diethyl ether (100:5). The product was isolated as a colorless oil (25 mg, 32%).

$^{1}$H NMR ($CDCl_3$, 400 MHz) δ 5.56 (dd, J = 3.1, 1.6 Hz, 1H), 5.30 (dd, J = 3.0, 1.1 Hz, 1H), 2.35 (ddd, J = 14.5, 7.8, 1.4 Hz, 1H), 2.08 (ddd, J = 14.6, 8.0, 1.3 Hz, 1H), 1.38 – 1.28 (m, 1H), 1.21 (s, 12H), 1.20 (s, 12H), 0.88 – 0.75 (m, 2H), 0.07 (s, 9H).

$^{13}$C NMR ($CDCl_3$, 100 MHz) δ 153.2, 125.6, 84.2, 84.1, 40.9, 32.3, 26.2, 26.2, 26.1, -0.00.

$^{11}$B NMR ($CDCl_3$, 128.3 MHz) δ 33.7.

HRMS-(ESI+) for $C_{20}H_{41}B_2O_4Si$ $[M+H]^+$: calculated:395.2798; found: 395.2808.

**2,2'-(pent-4-ene-1,2-diyl)bis(4,4,5,5-tetramethyl-1,3,2-dioxaborolane) (13)**

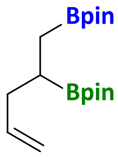


The product was purified by flash chromatography using as eluent a mixture of petroleum ether/diethyl ether (100:5). The product was isolated as a colorless oil (62 mg, 97%).

$^{1}$H NMR ($CDCl_3$, 400 MHz) δ 5.77 (ddt, J = 17.1, 10.1, 7.0 Hz, 1H), 4.96 (dd, J = 17.1, 2.5 Hz, 1H), 4.90 (dd, J = 10.1, 2.3 Hz, 1H), 2.20 (m, 1H), 2.12 – 2.01 (m, 1H), 1.21 (s, 24H), 1.21 (m, 1H), 0.90 – 0.75 (m, 2H).

$^{13}$C NMR ($CDCl_3$, 100 MHz) δ 138.7, 114.9, 82.9, 82.8, 37.8, 24.8, 24.8, 24.7, 24.7, 19.5(C-B), 12.9(C-B).

$^{11}$B NMR ($CDCl_3$, 128.3 MHz) δ 33.8.

HRMS-(ESI+) for $C_{17}H_{33}B_2O_4$ $[M+H]^+$: calculated: 323.2559; found: 323.2563.

**(*E*)-2,2'-(5-iodo-4-phenylpent-4-ene-1,2-diyl)bis(4,4,5,5-tetramethyl-1,3,2-dioxaborolane) (14)**

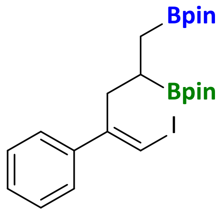


The product was purified by flash chromatography using as eluent a mixture of petroleum ether/diethyl ether (100:5). The product was isolated as a colorless oil (80 mg, 76%).

$^{1}$H NMR ($CDCl_3$, 400 MHz) δ 7.31 – 7.20 (m, 5H), 6.34 (s, 1H), 2.86 (dd, J = 13.8, 7.0 Hz, 1H), 2.71 (dd, J = 13.9, 8.6 Hz, 1H), 1.18 (s, 12H), 1.18 (m, 1H), 1.15 (s, 6H), 1.15 (s, 6H), 0.83 (m, 2H).

$^{13}$C NMR ($CDCl_3$, 100 MHz) δ 152.0, 141.0, 128.3, 126.8, 113.0, 82.9, 82.8, 79.6, 40.1, 24.9, 24.8, 24.8, 24.7.

$^{11}$B NMR ($CDCl_3$, 128.3 MHz) δ 34.6.

HRMS-(ESI+) for $C_{23}H_{36}B_2IO_4$ $[M+H]^+$: calculated: 525.1839; found: 525.1850.

**(*E*)-2,2'-(5-iodo-4-(p-tolyl)pent-4-ene-1,2-diyl)bis(4,4,5,5-tetramethyl-1,3,2-dioxaborolane) (15)**

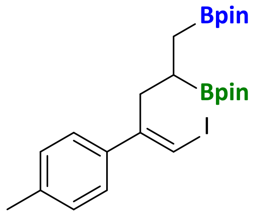


The product was purified by flash chromatography using as eluent a mixture of petroleum ether/diethyl ether (100:5). The product was isolated as a colorless oil (79 mg, 73%).

$^{1}$H NMR ($CDCl_3$, 400 MHz) δ 7.21 – 7.15 (m, 2H), 7.12 – 7.05 (m, 2H), 6.32 (s, 1H), 2.86 (dd, J = 13.8, 6.9 Hz, 1H), 2.71 (dd, J = 13.8, 8.7 Hz, 1H), 2.31 (s, 3H), 1.20 (s, 12H), 1.18 (s, 12H), 1.18 (m, 1H), 0.84 (m, 2H).

$^{13}$C NMR ($CDCl_3$, 100 MHz) δ 151.8, 138.1, 137.3, 129.0, 126.7, 82.9, 82.8, 78.8, 40.0, 24.9, 24.9, 24.8, 24.7, 21.1.

$^{11}$B NMR ($CDCl_3$, 128.3 MHz) δ 33.4.

HRMS-(ESI+) for $C_{24}H_{38}B_2IO_4$ $[M+H]^+$: calculated: 539.1995; found: 539.2004.

**(*E*)-2,2'-(5-iodo-4-(4-methoxyphenyl)pent-4-ene-1,2-diyl)bis(4,4,5,5-tetramethyl-1,3,2-dioxaborolane) (16)**

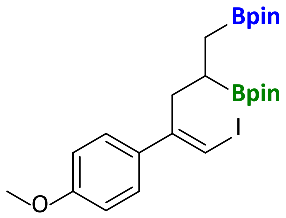


The product was purified by flash chromatography using as eluent a mixture of petroleum ether/diethyl ether (100:5). The product was isolated as a colorless oil (101 mg, 91%).

$^{1}$H NMR ($CDCl_3$, 400 MHz) δ 7.23 (d, J = 8.8 Hz, 2H), 6.81 (d, J = 8.8 Hz, 2H), 6.28 (s, 1H), 3.79 (s, 3H), 2.85 (dd, J = 13.8, 7.0 Hz, 1H), 2.70 (dd, J = 13.8, 8.8 Hz, 1H), 1.20 (s, 6H), 1.20 (s, 6H), 1.18 (s, 12H), 1.18 (m, 1H), 0.84 (m, 2H).

$^{13}$C NMR ($CDCl_3$, 100 MHz) δ 159.1, 151.3, 133.5, 127.9, 113.7, 82.9, 82.8, 78.0, 55.2, 40.0, 24.9, 24.9, 24.8, 24.7.

$^{11}$B NMR ($CDCl_3$, 128.3 MHz) δ 33.1.

HRMS-(ESI+) for $C_{24}H_{38}B_2IO_5$ $[M+H]^+$: calculated: 555.1952; found: 555.1945.

**(*E*)-2,2'-(4-(4-chlorophenyl)-5-iodopent-4-ene-1,2-diyl)bis(4,4,5,5-tetramethyl-1,3,2-dioxaborolane) (17)**

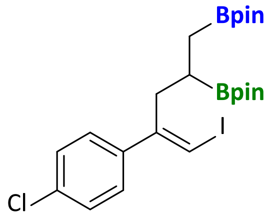


The product was purified by flash chromatography using as eluent a mixture of petroleum ether/diethyl ether (100:5). The product was isolated as a colorless oil (101 mg, 90%).

$^{1}$H NMR ($CDCl_3$, 400 MHz) δ 7.25 – 7.15 (m, 4H), 6.34 (s, 1H), 2.81 (dd, J = 13.9, 7.2 Hz, 1H), 2.66 (dd, J = 13.9, 8.6 Hz, 1H), 1.17 (m, 1H), 1.17 (s, 6H), 1.16 (s, 6H), 1.14 (s, 12H), 0.82 – 0.76 (m, 2H).

$^{13}C$ NMR ($CDCl_3$, 100 MHz) δ 150.9, 139.4, 133.4, 128.4, 128.1, 126.9, 83.0, 82.9, 80.1, 40.1, 24.9, 24.9, 24.8, 24.7.

$^{11}B$ NMR ($CDCl_3$, 128.3 MHz) δ 34.8.

HRMS-(ESI+) for $C_{23}H_{34}B_2ClIO_4$ $[M+H]^+$: calculated: 559.1452; found: 559.1449.

**(*E*)-2,2'-(4-(4-fluorophenyl)-5-iodopent-4-ene-1,2-diyl)bis(4,4,5,5-tetramethyl-1,3,2-dioxaborolane) (18)**

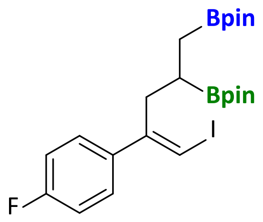


The product was purified by flash chromatography using as eluent a mixture of petroleum ether/diethyl ether (100:5). The product was isolated as a colorless oil (95 mg, 88%).

$^{1}H$ NMR ($CDCl_3$, 400 MHz) δ 7.43 (dd, J = 8.9, 5.4 Hz, 2H), 6.98 (d, J = 8.8 Hz, 1H), 6.96 (d, J = 8.8 Hz, 1H), 5.58 (s, 1H), 2.89 – 2.81 (m, 2H), 1.56 – 1.41 (m, 1H), 1.29 (s, 12H), 1.22 (s, 12H), 0.85 – 0.74 (m, 2H).

$^{13}C$ NMR ($CDCl_3$, 100 MHz) δ 162.5 (d, $^{1}J_{C-F}$ = 246.6 Hz), 161.9, 128.0 (d, $^{3}J_{C-F}$ = 8.0 Hz), 114.8 (d, $^{2}J_{C-F}$ = 21.2 Hz), 82.8, 82.8, 36.0, 24.8, 24.8.

$^{11}B$ NMR ($CDCl_3$, 128.3 MHz) δ 33.9.

$^{19}F$ NMR ($CDCl_3$, 377 MHz) δ -114.88.

HRMS-(ESI+) for $C_{23}H_{35}B_2FIO_4$ $[M+H]^+$: calculated: 543.1745; found: 543.1749.

**(*E*)-2,2'-(5-iodo-4-(o-tolyl)pent-4-ene-1,2-diyl)bis(4,4,5,5-tetramethyl-1,3,2-dioxaborolane) (19)**

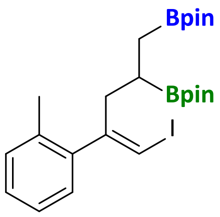


The product was purified by flash chromatography using as eluent a mixture of petroleum ether/diethyl ether (100:5). The product was isolated as a colorless oil (78 mg, 72%).

$^{1}H$ NMR ($CDCl_3$, 400 MHz) δ 7.19 – 7.03 (m, 4H), 6.08 (s, 1H), 2.72 (dd, *J* = 13.7, 6.3 Hz, 1H), 2.61 (dd, *J* = 13.7, 8.7 Hz, 1H), 2.25 (s, 3H), 1.20 (m, 1H), 1.20 (s, 6H), 1.20 (s, 6H), 1.18 (s, 6H), 1.17 (s, 6H), 0.96 – 0.84 (m, 2H).

$^{13}C$ NMR ($CDCl_3$, 100 MHz) δ 152.5, 141.1, 135.1, 130.1, 128.9, 127.2, 125.4, 83.0, 82.8, 80.1, 41.3, 24.9, 24.8, 24.8, 24.7, 19.8, 15.7(C-B), 12.1(C-B).

$^{11}B$ NMR ($CDCl_3$, 128.3 MHz) δ 33.8.

HRMS-(ESI+) for $C_{24}H_{38}B_2IO_4$ $[M+H]^+$: calculated: 539.1992; found: 539.1995.

**(*E*)-2,2'-(4-(2-bromophenyl)-5-iodopent-4-ene-1,2-diyl)bis(4,4,5,5-tetramethyl-1,3,2-dioxaborolane) (20)**

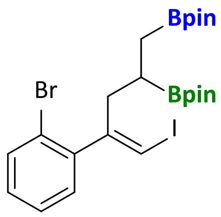


The product was purified by flash chromatography using as eluent a mixture of petroleum ether/diethyl ether (100:5). The product was isolated as a colorless oil (105 mg, 87%).

$^1$H NMR (CDCl$_3$, 400 MHz) δ 7.59 (d, J = 8.0 Hz, 1H), 7.40 – 7.22 (m, 2H), 7.20 (d, J = 2.0 Hz, 1H), 6.31 (s, 1H), 2.90 (dd, J = 13.8, 6.3 Hz, 1H), 2.75 (dd, J = 13.9, 9.0 Hz, 1H), 1.27 (s, 24H), 1.04 (m, 1H), 1.04 – 0.93 (m, 2H).

$^{13}$C NMR (CDCl$_3$, 100 MHz) δ 151.8, 142.0, 132.8, 130.7, 128.7, 127.0, 122.1, 83.0, 82.8, 40.7, 24.9, 24.9, 24.7, 24.7.

$^{11}$B NMR (CDCl$_3$, 128.3 MHz) δ 33.5.

HRMS-(ESI+) for $C_{23}H_{35}B_2BrIO_4$ [M+H]$^+$: calculated: 603.0944; found: 603.0965.

**(*E*)-2,2'-(5-iodo-4-(naphthalen-2-yl)pent-4-ene-1,2-diyl)bis(4,4,5,5-tetramethyl-1,3,2-dioxaborolane) (21)**

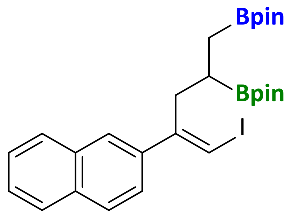


The product was purified by flash chromatography using as eluent a mixture of petroleum ether/diethyl ether (100:5). The product was isolated as a colorless oil (65 mg, 57%).

$^1$H NMR (CDCl$_3$, 400 MHz) δ 7.83 – 7.73 (m, 4H), 7.50 – 7.39 (m, 3H), 6.51 (s, 1H), 2.99 (dd, J = 13.9, 7.0 Hz, 1H), 2.84 (dd, J = 13.9, 8.7 Hz, 1H), 1.30 – 1.24 (m, 1H), 1.20 (s, 6H), 1.18 (s, 6H), 1.15 (s, 6H), 1.14 (s, 6H), 0.89 (d, J = 7.6 Hz, 2H).

$^{13}$C NMR (CDCl$_3$, 100 MHz) δ 152.0, 138.4, 133.3, 132.8, 128.1, 127.8, 127.5, 126.1, 125.8, 125.8, 125.1, 82.9, 82.8, 80.1, 40.1, 24.9, 24.8, 24.7.

$^{11}$B NMR (CDCl$_3$, 128.3 MHz) δ 33.6.

HRMS-(ESI+) for $C_{27}H_{38}B_2IO_4$ [M+H]$^+$: calculated: 575.1995; found: 575.1989.

**(*E*)-2,2'-(5-iodo-4-(thiophen-3-yl)pent-4-ene-1,2-diyl)bis(4,4,5,5-tetramethyl-1,3,2-dioxaborolane) (22)**

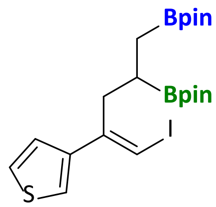


The product was purified by flash chromatography using as eluent a mixture of petroleum ether/diethyl ether (100:5). The product was isolated as a colorless oil (74 mg, 70%).

$^1$H NMR (CDCl$_3$, 400 MHz) δ 7.30 (m, 1H), 7.23 (m, 1H), 7.12 (dd, J = 5.0, 1.4 Hz, 1H), 6.56 (s, 1H), 2.72 – 2.60 (m, 2H), 1.39 (m, 1H), 1.23 (s, 12H), 1.22 (s, 6H), 1.21 (s, 6H), 0.90 – 0.85 (m, 2H).

$^{13}C$ NMR ($CDCl_3$, 100 MHz) δ 146.0, 141.3, 126.0, 125.3, 121.3, 82.9, 79.0, 40.0, 24.9, 24.9, 24.7, 24.7.

$^{11}B$ NMR ($CDCl_3$, 128.3 MHz) δ 33.1.

HRMS-(ESI+) for $C_{21}H_{33}B_2INaO_4S$ $[M+Na]^+$: calculated: 553.1228; found: 553.1236.

**(*E*)-2,2'-(4-(cyclohex-1-en-1-yl)-5-iodopent-4-ene-1,2-diyl)bis(4,4,5,5-tetramethyl-1,3,2-dioxaborolane) (23)**

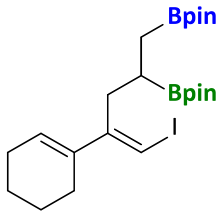


The product was purified by flash chromatography using as eluent a mixture of petroleum ether/diethyl ether (100:5). The product was isolated as a colorless oil (91 mg, 86%).

$^1H$ NMR ($CDCl_3$, 400 MHz) δ 6.17 (s, 1H), 5.83 (m, 1H), 2.58 (dd, J = 13.8, 6.6 Hz, 1H), 2.40 (dd, J = 13.8, 9.1 Hz, 1H), 2.07 (m, 4H), 1.69 – 1.60 (m, 2H), 1.58 – 1.47 (m, 2H), 1.24 (s, 6H), 1.22 (s, 18H), 1.22 (m, 1H), 0.88 – 0.80 (m, 2H).

$^{13}C$ NMR ($CDCl_3$, 100 MHz) δ 152.9, 136.7, 125.9, 82.9, 82.8, 77.5, 37.6, 26.8, 25.7, 24.9, 24.9, 24.8, 24.7, 22.8, 21.9.

$^{11}B$ NMR ($CDCl_3$, 128.3 MHz) δ 33.8.

HRMS-(ESI+) for $C_{23}H_{40}B_2IO_4$ $[M+H]^+$: calculated: 529.2160; found: 529.2169.

**(*E*)-(1-iodo-4,5-bis(4,4,5,5-tetramethyl-1,3,2-dioxaborolan-2-yl)pent-1-en-2-yl)trimethylsilane (24)**

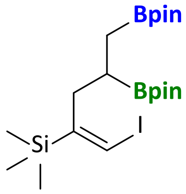


The product was purified by flash chromatography using as eluent a mixture of petroleum ether/diethyl ether (100:5). The product was isolated as a colorless oil (54 mg, 52%).

$^1H$ NMR ($CDCl_3$, 400 MHz) δ 6.55 (s, 1H), 2.51 – 2.42 (m, 1H), 2.27 (ddd, J = 13.4, 10.4, 0.7 Hz, 1H), 1.36 (m, 1H), 1.24 (s, 6H), 1.22 (s, 6H), 1.22 (s, 6H), 1.21 (s, 6H), 0.85 – 0.75 (m, 2H), 0.14 (s, 9H).

$^{13}C$ NMR ($CDCl_3$, 100 MHz) δ 155.1, 93.4, 82.9, 82.8, 42.6, 24.9, 24.9, 24.7, 24.7, -0.5.

$^{11}B$ NMR ($CDCl_3$, 128.3 MHz) δ 33.6.

HRMS-(ESI+) for $C_{20}H_{40}B_2IO_4Si$ $[M+H]^+$: calculated: 521.1926; found: 521.1928.

**(*E*)-2,2'-(5-bromo-4-phenylpent-4-ene-1,2-diyl)bis(4,4,5,5-tetramethyl-1,3,2-dioxaborolane) (25)**

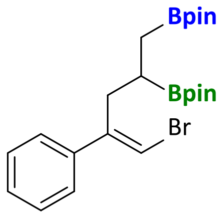


The product was purified by flash chromatography using as eluent a mixture of petroleum ether/diethyl ether (100:5). The product was isolated as a colorless oil (85 mg, 89%).

$^{1}$H NMR ($CDCl_3$, 400 MHz) δ 7.33 – 7.18 (m, 5H), 6.28 (s, 1H), 2.78 (m, 2H), 1.18 (s, 6H), 1.17 (m, 1H), 1.17 (s, 6H), 1.15 (s, 6H), 1.14 (s, 6H), 0.82 (m, 2H).

$^{13}$C NMR ($CDCl_3$, 100 MHz) δ 146.4, 140.3, 128.3, 127.5, 126.9, 105.7, 82.9, 82.8, 35.8, 24.9, 24.8, 24.8, 24.7.

$^{11}$B NMR ($CDCl_3$, 128.3 MHz) δ 33.3.

HRMS-(ESI+) for $C_{23}H_{36}B_2BrO_4$ $[M+H]^+$: calculated: 477.1978; found: 477.1972.

**(*E*)-2,2'-(5-bromo-4-(4-methoxyphenyl)pent-4-ene-1,2-diyl)bis(4,4,5,5-tetramethyl-1,3,2-dioxaborolane) (26)**

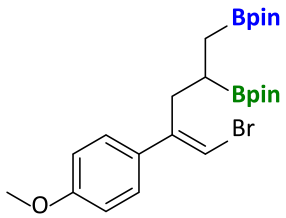


The product was purified by flash chromatography using as eluent a mixture of petroleum ether/diethyl ether (100:5). The product was isolated as a colorless oil (85 mg, 84%).

$^{1}$H NMR ($CDCl_3$, 400 MHz) δ 7.16 (d, J = 8.8 Hz, 2H), 6.76 (d, J = 8.8 Hz, 2H), 6.17 (s, 1H), 3.73 (s, 3H), 2.71 (m, 2H), 1.14 (m, 1H), 1.14 (s, 6H), 1.13 (s, 6H), 1.11 (s, 6H), 1.11 (s, 6H), 0.75 (m, 2H).

$^{13}$C NMR ($CDCl_3$, 100 MHz) δ 159.2, 145.7, 132.7, 128.0, 113.8, 104.3, 82.9, 82.8, 55.2, 35.8, 24.9, 24.9, 24.8, 24.7.

$^{11}$B NMR ($CDCl_3$, 128.3 MHz) δ 35.1.

HRMS-(ESI+) for $C_{24}H_{38}B_2BrO_5$ $[M+H]^+$: calculated: 507.2092; found: 507.2083.

**(*E*)-2,2'-(5-bromo-4-(4-chlorophenyl)pent-4-ene-1,2-diyl)bis(4,4,5,5-tetramethyl-1,3,2-dioxaborolane) (27)**

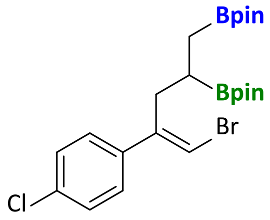


The product was purified by flash chromatography using as eluent a mixture of petroleum ether/diethyl ether (100:5). The product was isolated as a colorless oil (92 mg, 95%).

$^{1}$H NMR ($CDCl_3$, 400 MHz) δ 7.36 – 7.24 (m, 4H), 6.35 (s, 1H), 2.91 – 2.73 (m, 2H), 1.25 (m, 1H), 1.25 (s, 6H), 1.24 (s, 6H), 1.22 (s, 6H), 1.22 (s, 6H), 0.99 – 0.79 (m, 2H).

$^{13}C$ NMR ($CDCl_3$, 100 MHz) δ 145.4, 138.7, 133.4, 128.5, 128.2, 106.6, 83.0, 82.9, 35.8, 24.9, 24.8, 24.8, 24.7.

$^{11}B$ NMR ($CDCl_3$, 128.3 MHz) δ 33.3.

HRMS-(ESI+) for $C_{23}H_{35}B_2BrClO_4$ $[M+H]^+$: calculated: 511.1596; found: 511.1588.

**(*E*)-2,2'-(5-bromo-4-(2-bromophenyl)pent-4-ene-1,2-diyl)bis(4,4,5,5-tetramethyl-1,3,2-dioxaborolane) (28)**

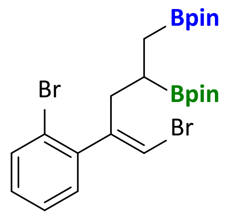


The product was purified by flash chromatography using as eluent a mixture of petroleum ether/diethyl ether (100:5). The product was isolated as a colorless oil (103 mg, 93%).

$^1H$ NMR ($CDCl_3$, 400 MHz) δ 7.52 (d, J = 7.9 Hz, 1H), 7.24 – 7.17 (m, 2H), 7.11 (ddd, J = 7.9, 7.0, 2.1 Hz, 1H), 6.16 (s, 1H), 2.76 (m, 2H), 1.20 (s, 24H), 1.16 – 1.06 (m, 1H), 0.97 – 0.89 (m, 2H).

$^{13}C$ NMR ($CDCl_3$, 100 MHz) δ 146.2, 141.3, 132.8, 131.0, 128.9, 127.3, 122.6, 108.1, 83.0, 82.8, 36.6, 24.9, 24.8, 24.8, 24.7.

$^{11}B$ NMR ($CDCl_3$, 128.3 MHz) δ 33.9.

HRMS-(ESI+) for $C_{23}H_{35}B_2Br_2O_4$ $[M+H]^+$: calculated: 555.1083; found: 555.1105.

**(*E*)-2,2'-(5-bromo-4-(cyclohex-1-en-1-yl)pent-4-ene-1,2-diyl)bis(4,4,5,5-tetramethyl-1,3,2-dioxaborolane) (29)**

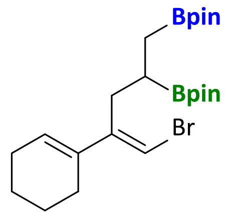


The product was purified by flash chromatography using as eluent a mixture of petroleum ether/diethyl ether (100:5). The product was isolated as a colorless oil (75 mg, 78%).

$^1H$ NMR ($CDCl_3$, 400 MHz) δ 6.16 (s, 1H), 5.85 – 5.80 (m, 1H), 2.55 (dd, J = 13.7, 6.6 Hz, 1H), 2.45 (dd, J = 13.7, 9.2 Hz, 1H), 2.06 (m, 4H), 1.70 – 1.59 (m, 2H), 1.55 (m, 2H), 1.23 (m, 1H), 1.23 (s, 6H), 1.22 (s, 18H), 0.84 (m, 2H).

$^{13}C$ NMR ($CDCl_3$, 100 MHz) δ 147.1, 135.7, 125.7, 103.5, 103.5, 82.9, 82.8, 33.1, 26.5, 25.7, 24.6, 24.9, 24.8, 24.7, 22.7, 22.0.

$^{11}B$ NMR ($CDCl_3$, 128.3 MHz) δ 34.3.

HRMS-(ESI+) for $C_{23}H_{39}B_2BrNaO_4$ $[M+Na]^+$: calculated: 503.2115; found: 503.2110.

## - Characterization data for cross-coupling products and corresponding alcohols after oxidation

**4,4,5,5-tetramethyl-2-(3-phenylcyclopent-3-en-1-yl)-1,3,2-dioxaborolane (30)**

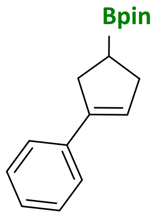


The product was purified by flash chromatography using as eluent a mixture of petroleum ether/diethyl ether (100:5). The product was isolated as a colorless oil (45 mg, 84%).

$^{1}$H NMR ($CDCl_3$, 400 MHz) δ 7.46 – 7.40 (m, 2H), 7.30 (dd, J = 7.8, 6.9 Hz, 2H), 7.23 – 7.16 (m, 1H), 6.17 (m, 1H), 2.89 (m, 1H), 2.83 – 2.65 (m, 2H), 2.58 (m, 1H), 1.83 (m, 1H), 1.27 (s, 12H).

$^{13}$C NMR ($CDCl_3$, 100 MHz) δ 142.4, 136.7, 128.2, 126.7, 126.1, 125.6, 83.1, 35.7, 35.6, 24.7.

$^{11}$B NMR ($CDCl_3$, 128.3 MHz) δ 35.4.

HRMS-(ESI+) for $C_{17}H_{24}BO_2$ $[M+H]^+$: calculated: 271.1864; found: 271.1860.

**4,4,5,5-tetramethyl-2-(3-(p-tolyl)cyclopent-3-en-1-yl)-1,3,2-dioxaborolane (31)**

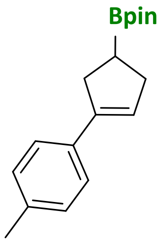


The product was purified by flash chromatography using as eluent a mixture of petroleum ether/diethyl ether (100:5). The product was isolated as a colorless oil (37 mg, 65%).

$^{1}$H NMR ($CDCl_3$, 400 MHz) δ 7.36 – 7.30 (m, 2H), 7.14 – 7.07 (m, 2H), 6.10 (m, 1H), 2.87 (m, 1H), 2.81 – 2.63 (m, 2H), 2.57 (m, 1H), 2.33 (s, 3H), 1.82 (m, 1H), 1.27 (s, 12H).

$^{13}$C NMR ($CDCl_3$, 100 MHz) δ 142.2, 136.4, 133.9, 128.9, 125.5, 125.0, 83.1, 35.6, 24.8, 24.7, 21.1.

$^{11}$B NMR ($CDCl_3$, 128.3 MHz) δ 35.5.

HRMS-(ESI+) for $C_{18}H_{26}BO_2$ $[M+H]^+$: calculated: 285.2026; found: 285.2018.

**2-(3-(4-methoxyphenyl)cyclopent-3-en-1-yl)-4,4,5,5-tetramethyl-1,3,2-dioxaborolane (32)**

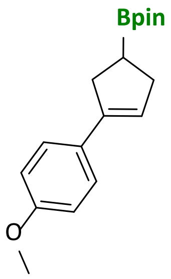


The product was purified by flash chromatography using as eluent a mixture of petroleum ether/diethyl ether (100:5). The product was isolated as a colorless oil (52 mg, 87%).

$^{1}$H NMR ($CDCl_3$, 400 MHz) δ 7.37 (d, J = 8.8 Hz, 2H), 6.88 – 6.80 (m, 2H), 6.02 (m, 1H), 3.80 (s, 3H), 2.92 – 2.81 (m, 1H), 2.77 – 2.62 (m, 2H), 2.62 – 2.49 (m, 1H), 1.89 – 1.75 (m, 1H), 1.27 (s, 12H).

$^{13}$C NMR ($CDCl_3$, 100 MHz) δ 158.5, 141.7, 129.6, 126.7, 123.9, 113.6, 83.1, 55.2, 35.7, 35.6, 24.7.

$^{11}$B NMR ($CDCl_3$, 128.3 MHz) δ 34.5.

HRMS-(ESI+) for $C_{18}H_{26}BO_3$ $[M+H]^+$: calculated: 301.1965; found: 301.1970.

**4,4,5,5-tetramethyl-2-(3-(o-tolyl)cyclopent-3-en-1-yl)-1,3,2-dioxaborolane (33)**

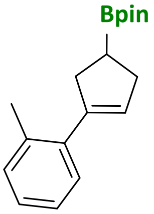


The product was purified by flash chromatography using as eluent a mixture of petroleum ether/diethyl ether (100:5). The product was isolated as a colorless oil (53 mg, 93%).

$^{1}$H NMR ($CDCl_3$, 400 MHz) δ 7.25 – 7.05 (m, 4H), 5.75 (m, 1H), 2.84 – 2.73 (m, 2H), 2.77 – 2.63 (m, 1H), 2.58 (m, 1H), 2.36 (s, 3H), 1.87 – 1.74 (m, 1H), 1.27 (s, 12H).

$^{13}$C NMR ($CDCl_3$, 100 MHz) δ 143.2, 138.1, 135.5, 130.3, 129.3, 128.0, 126.4, 125.4, 83.1, 38.9, 35.9, 24.7, 21.2.

$^{11}$B NMR ($CDCl_3$, 128.3 MHz) δ 34.7.

HRMS-(ESI+) for $C_{18}H_{26}BO_2$ $[M+H]^+$: calculated: 285.2026; found: 285.2031.

**2-(3-(4-chlorophenyl)cyclopent-3-en-1-yl)-4,4,5,5-tetramethyl-1,3,2-dioxaborolane (34)**

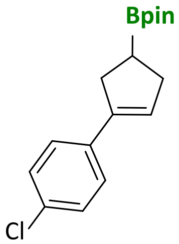


The product was purified by flash chromatography using as eluent a mixture of petroleum ether/diethyl ether (100:5). The product was isolated as a colorless oil (52 mg, 85%).

$^{1}$H NMR ($CDCl_3$, 400 MHz) δ 7.35 (d, J = 8.5 Hz, 2H), 7.30 – 7.20 (m, 2H), 6.18 – 6.12 (m, 1H), 2.84 (m, 1H), 2.78 – 2.63 (m, 2H), 2.57 (m, 1H), 1.89 – 1.75 (m, 1H), 1.26 (s, 12H).

$^{13}$C NMR ($CDCl_3$, 100 MHz) δ 141.3, 135.2, 132.3, 128.3, 126.8, 126.8, 83.2, 35.7, 35.6, 24.7, 24.7.

$^{11}$B NMR ($CDCl_3$, 128.3 MHz) δ 34.5.

HRMS-(ESI+) for $C_{17}H_{23}BClO_2$ $[M+H]^+$: calculated: 305.1480; found: 305.1489.

**2-(3-(4-fluorophenyl)cyclopent-3-en-1-yl)-4,4,5,5-tetramethyl-1,3,2-dioxaborolane (35)**

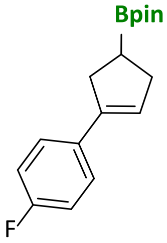


The product was purified by flash chromatography using as eluent a mixture of petroleum ether/diethyl ether (100:5). The product was isolated as a colorless oil (55 mg, 96%).

$^{1}$H NMR ($CDCl_3$, 400 MHz) δ 7.45 – 7.34 (m, 2H), 7.03 – 6.90 (m, 2H), 6.12 – 6.06 (m, 1H), 2.90 – 2.79 (m, 1H), 2.79 – 2.64 (m, 2H), 2.57 (m, 1H), 1.90 – 1.76 (m, 1H), 1.27 (s, 12H).

$^{13}$C NMR ($CDCl_3$, 100 MHz) δ161.8 (d, $^{1}J_{C-F}$ = 245.6 Hz), 141.3, 132.9, 132.9, 127.1 (d, $^{3}J_{C-F}$ = 7.9 Hz), 125.7, 125.7, 115.0 (d, $^{2}J_{C-F}$ = 21.4 Hz), 83.2, 35.8, 35.7, 24.7, 24.7.

$^{11}$B NMR ($CDCl_3$, 128.3 MHz) δ 35.3.

$^{19}$F NMR ($CDCl_3$, 377 MHz) δ -115.86.

HRMS-(ESI+) for $C_{17}H_{23}BFO_2$ $[M+H]^+$: calculated: 289.1775; found: 289.1778.

**4,4,5,5-tetramethyl-2-(3-(thiophen-3-yl)cyclopent-3-en-1-yl)-1,3,2-dioxaborolane (36)**

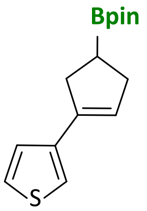


The product was purified by flash chromatography using as eluent a mixture of petroleum ether/diethyl ether (100:5). The product was isolated as a colorless oil (44 mg, 79%).

$^{1}$H NMR ($CDCl_3$, 400 MHz) δ 7.24 (d, J = 2.1 Hz, 2H), 7.07 (t, J = 2.1 Hz, 1H), 6.03 – 5.97 (m, 1H), 2.91 – 2.80 (m, 1H), 2.80 – 2.62 (m, 2H), 2.62 – 2.49 (m, 1H), 1.88 – 1.74 (m, 1H), 1.27 (s, 12H).

$^{13}$C NMR ($CDCl_3$, 100 MHz) δ 138.9, 137.9, 125.8, 125.5, 125.3, 119.8, 83.1, 36.1, 35.5, 24.8, 24.7.

$^{11}$B NMR ($CDCl_3$, 128.3 MHz) δ 34.7.

HRMS-(ESI+) for $C_{15}H_{22}BO_2S$ $[M+H]^+$: calculated: 277.1422; found: 277.1434.

**2-(3-(cyclohex-1-en-1-yl)cyclopent-3-en-1-yl)-4,4,5,5-tetramethyl-1,3,2-dioxaborolane (37)**

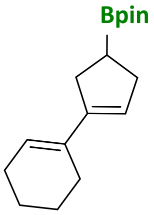


The product was purified by flash chromatography using as eluent a mixture of petroleum ether/diethyl ether (100:5). The product was isolated as a colorless oil (49 mg, 90%).

$^{1}$H NMR ($CDCl_3$, 400 MHz) δ 5.70 (m, 1H), 5.64 – 5.60 (m, 1H), 2.72 – 2.61 (m, 1H), 2.60 – 2.40 (m, 4H), 2.21 (m, 2H), 2.13 (m, 2H), 1.70 – 1.63 (m, 2H), 1.60 – 1.52 (m, 2H), 1.25 (s, 12H).

$^{13}$C NMR ($CDCl_3$, 100 MHz) δ 144.4, 133.5, 124.5, 123.5, 83.0, 35.3, 34.5, 26.2, 25.7, 24.7, 22.8, 22.4.

$^{11}$B NMR ($CDCl_3$, 128.3 MHz) δ 35.0.

HRMS-(ESI+) for $C_{17}H_{28}BO_2$ $[M+H]^+$: calculated: 275.2177; found: 275.2175.

**4,4,5,5-tetramethyl-2-(3-(naphthalen-2-yl)cyclopent-3-en-1-yl)-1,3,2-dioxaborolane (38)**

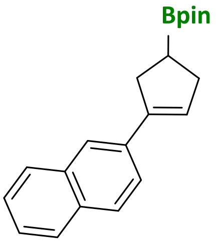


The product was purified by flash chromatography using as eluent a mixture of petroleum ether/diethyl ether (100:5). The product was isolated as a colorless oil (60 mg, 94%).

$^{1}$H NMR ($CDCl_3$, 400 MHz) δ 7.83 – 7.66 (m, 5H), 7.50 – 7.37 (m, 2H), 6.35 – 6.29 (m, 1H), 3.03 (m, 1H), 2.91 (m, 1H), 2.77 (m, 1H), 2.64 (m, 1H), 1.96 – 1.85 (m, 1H), 1.29 (s, 12H).

$^{13}$C NMR ($CDCl_3$, 100 MHz) δ 142.4, 134.0, 133.6, 132.5, 128.0, 127.6, 127.5, 126.9, 126.0, 125.4, 124.3, 124.1, 83.2, 35.8, 35.6, 24.8, 24.8.

$^{11}$B NMR ($CDCl_3$, 128.3 MHz) δ 34.7.

HRMS-(ESI+) for $C_{21}H_{26}BO_2$ $[M+H]^+$: calculated: 321.2020; found: 321.2031.

**Trimethyl(4-(4,4,5,5-tetramethyl-1,3,2-dioxaborolan-2-yl)cyclopent-1-en-1-yl)silane (39)**

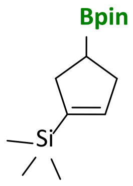

The product was purified by flash chromatography using as eluent a mixture of petroleum ether/diethyl ether (100:5). The product was isolated as a colorless oil (52 mg, 97%).

$^{1}$H NMR ($CDCl_3$, 400 MHz) δ 5.99 – 5.92 (m, 1H), 2.65 – 2.49 (m, 2H), 2.40 – 2.25 (m, 2H), 1.60 – 1.52 (m, 1H), 1.25 (s, 12H), 0.06 (s, 9H).

$^{13}$C NMR ($CDCl_3$, 100 MHz) δ 146.1, 141.9, 84.6, 40.0, 39.0, 26.3, -0.00.

$^{11}$B NMR ($CDCl_3$, 128.3 MHz) δ 34.6.

HRMS-(ESI+) for $C_{14}H_{28}BO_2Si$ $[M+H]^+$: calculated: 267.1952; found: 267.1964.

**2-(3-(2-bromophenyl)cyclopent-3-en-1-yl)-4,4,5,5-tetramethyl-1,3,2-dioxaborolane (50)**

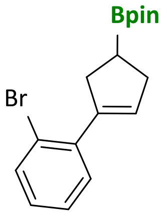

The product was purified by flash chromatography using as eluent a mixture of petroleum ether/diethyl ether (100:5). The product was isolated as a colorless oil (61 mg, 88%).

$^{1}$H NMR ($CDCl_3$, 400 MHz) δ 7.55 (d, J = 8.0 Hz, 1H), 7.25 – 7.20 (m, 2H), 7.06 (dd, J = 8.0, 5.3 Hz, 1H), 5.97 (m, 1H), 2.89 (m, 1H), 2.84 – 2.74 (m, 1H), 2.69 (m, 1H), 2.57 (m, 1H), 1.91 – 1.76 (m, 1H), 1.27 (s, 12H).

$^{13}$C NMR ($CDCl_3$, 100 MHz) δ 142.8, 139.5, 133.1, 131.2, 129.9, 127.9, 126.9, 125.6, 122.2, 83.1, 38.4, 35.7, 24.7.

$^{11}$B NMR ($CDCl_3$, 128.3 MHz) δ 34.9.

HRMS-(ESI+) for $C_{17}H_{23}BBrO_2$ $[M+H]^+$: calculated: 349.0963; found 349.0967.

**4,4,5,5-tetramethyl-2-(4-methylene-1,2,3,4-tetrahydronaphthalen-2-yl)-1,3,2-dioxaborolane and 4,4,5,5-tetramethyl-2-((3-methylene-2,3-dihydro-1H-inden-1-yl)methyl)-1,3,2-dioxaborolane (52-53)**

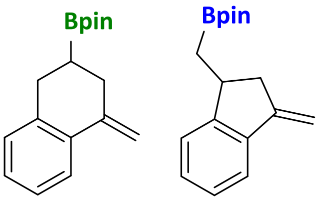

The products were purified by flash chromatography using as eluent a mixture of petroleum ether/diethyl ether (100:5). The products were isolated as a mixture of a colorless oil (20 mg, 36%, 1:1 ratio).

**Product 52:** $^{1}$H NMR ($CDCl_3$, 400 MHz) δ 7.65 – 7.60 (m, 1H), 7.48 – 7.43 (m, 1H), 7.25 – 7.16 (m, 2H), 5.41 (t, J = 2.5 Hz, 1H), 5.01 (t, J = 2.1 Hz, 1H), 3.39 (tt, J = 9.9, 5.3 Hz, 1H), 2.51 – 2.35 (m, 2H), 1.35 (dd, J = 15.6, 5.0 Hz, 1H), 1.26 (s, 6H), 1.26 (s, 6H), 1.01 (dd, J = 15.6, 9.7 Hz, 1H).

**Product 53:** $^{1}$H NMR ($CDCl_3$, 400 MHz) δ 7.31 – 7.27 (m, 1H), 7.18 – 7.08 (m, 3H), 5.45 (m, 1H), 4.95 (m, 1H), 3.06 (ddt, J = 16.3, 8.1, 2.1 Hz, 1H), 2.95 – 2.77 (m, 2H), 2.73 – 2.65 (m, 1H), 1.53 – 1.43 (m, 1H), 1.25 (s, 12H).

$^{13}$C NMR ($CDCl_3$, 100 MHz) δ 151.7, 150.2, 149.4, 145.1, 143.5, 140.4, 137.7, 137.6, 136.1, 134.6, 128.9, 128.3, 127.4, 126.4, 126.1, 125.7, 124.6, 124.2, 124.2, 122.6, 120.3, 118.7, 107.5, 102.3, 83.2, 83.1, 44.6, 40.9, 38.1, 34.3, 31.7, 24.9, 24.9, 24.7, 24.7, 24.7, 12.8.

$^{11}$B NMR ($CDCl_3$, 128.3 MHz) δ 34.1.

HRMS-(ESI+) for $C_{17}H_{24}BO_2$ $[M+H]^+$: calculated: 271.1864; found: 271.1869.

**3-Phenylcyclopent-3-en-1-ol (40)**

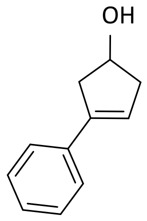


The product was purified by flash chromatography using as eluent a mixture of petroleum ether/diethyl ether (100:5). The product was isolated as a colorless oil (29 mg, 91%).

$^{1}$H NMR ($CDCl_3$, 400 MHz) δ 7.39 – 7.33 (m, 2H), 7.32 – 7.21 (m, 2H), 7.20 – 7.08 (m, 2H), 6.05 (dt, *J* = 3.9, 2.0 Hz, 1H), 4.57 (tt, *J* = 6.3, 2.4 Hz, 1H), 2.96 (ddq, *J* = 16.5, 6.1, 1.9 Hz, 1H), 2.81 (ddq, *J* = 17.9, 6.1, 1.9 Hz, 1H), 2.63 (ddq, *J* = 16.5, 2.8, 1.4 Hz, 1H), 2.44 (dtt, *J* = 17.9, 2.8, 1.5 Hz, 1H), 1.83 (s, 1H).

$^{13}$C NMR ($CDCl_3$, 100 MHz) δ 140.0, 136.1, 128.3, 127.2, 125.6, 122.9, 71.8, 43.4, 43.3.

**3-(p-Tolyl)cyclopent-3-en-1-ol (41)**

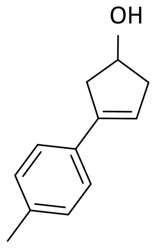


The product was purified by flash chromatography using as eluent a mixture of petroleum ether/diethyl ether (100:5). The product was isolated as a colorless oil (28 mg, 80%).

$^{1}$H NMR ($CDCl_3$, 400 MHz) δ 7.36 – 7.30 (m, 2H), 7.13 (d, J = 7.9 Hz, 2H), 6.07 (bs, 1H), 4.65 (m, 1H), 3.03 (dd, J = 16.5, 6.1 Hz, 1H), 2.88 (dd, J = 17.9, 6.2 Hz, 1H), 2.69 (dd, J = 16.5, 2.6 Hz, 1H), 2.51 (dd, J = 17.8, 2.3 Hz, 1H), 2.34 (s, 3H).

$^{13}$C NMR ($CDCl_3$, 100 MHz) δ 139.9, 137.0, 133.3, 129.0, 125.5, 121.8, 71.9, 43.4, 43.4, 21.1.

**3-(4-Methoxyphenyl)cyclopent-3-en-1-ol (42)**

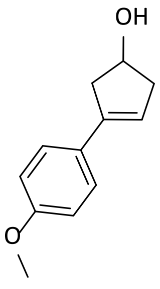


The product was purified by flash chromatography using as eluent a mixture of petroleum ether/diethyl ether (100:5). The product was isolated as a colorless oil (31 mg, 81%).

$^{1}$H NMR ($CDCl_3$, 400 MHz) δ 7.40 – 7.34 (m, 2H), 6.89 – 6.82 (m, 2H), 5.99 (bs, 1H), 4.64 (m, 1H), 3.81 (s, 3H), 3.07 – 2.95 (m, 1H), 2.88 (dd, J = 17.6, 6.1 Hz, 1H), 2.68 (dd, J = 16.4, 2.6 Hz, 1H), 2.55 – 2.46 (m, 1H).

$^{13}C$ NMR ($CDCl_3$, 100 MHz) δ 158.9, 39.4, 129.0, 126.7, 120.6, 113.7, 71.9, 55.2, 43.4, 43.4.

**3-(o-Tolyl)cyclopent-3-en-1-ol (43)**

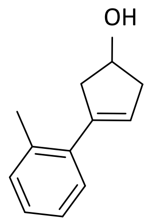

The product was purified by flash chromatography using as eluent a mixture of petroleum ether/diethyl ether (100:5). The product was isolated as a colorless oil (34 mg, 97%).

$^{1}H$ NMR ($CDCl_3$, 400 MHz) δ 7.22 – 7.10 (m, 4H), 5.74 (bs, 1H), 4.63 (m, 1H), 3.03 (dd, *J* = 16.5, 6.0 Hz, 1H), 2.89 (dd, *J* = 17.4, 6.0 Hz, 1H), 2.66 (dd, *J* = 16.6, 2.9 Hz, 1H), 2.53 (dd, *J* = 17.4, 2.7 Hz, 1H), 2.37 (s, 3H).

$^{13}C$ NMR ($CDCl_3$, 100 MHz) δ 140.9, 137.3, 135.6, 130.5, 128.1, 126.9, 126.2, 125.6, 125.5, 71.9, 46.5, 43.5, 21.2.

**3-(4-Chlorophenyl)cyclopent-3-en-1-ol (44)**

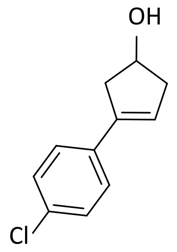

The product was purified by flash chromatography using as eluent a mixture of petroleum ether/diethyl ether (100:5). The product was isolated as a colorless oil (37 mg, 95%).

$^{1}H$ NMR ($CDCl_3$, 400 MHz) δ 7.28 (d, J = 8.6 Hz, 2H), 7.24 – 7.12 (m, 2H), 6.05 (bs, 1H), 4.59 (m, 1H), 2.94 (dd, J = 16.5, 6.1 Hz, 1H), 2.82 (dd, J = 18.0, 6.2 Hz, 1H), 2.60 (dd, J = 16.5, 2.9 Hz, 1H), 2.45 (dd, J = 18.0, 2.7 Hz, 1H).

$^{13}C$ NMR ($CDCl_3$, 100 MHz) δ 139.0, 134.6, 132.8, 128.5, 126.8, 123.6, 71.7, 43.5, 43.3.

**3-(4-Fluorophenyl)cyclopent-3-en-1-ol (45)**

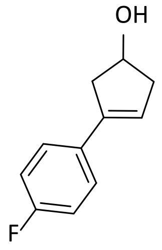

The product was purified by flash chromatography using as eluent a mixture of petroleum ether/diethyl ether (100:5). The product was isolated as a colorless oil (32 mg, 90%).

$^{1}H$ NMR ($CDCl_3$, 400 MHz) δ 7.43 – 7.34 (m, 2H), 7.05 – 6.95 (m, 2H), 6.04 (bs, 1H), 4.65 (m, 1H), 3.00 (dd, J = 16.4, 6.1 Hz, 1H), 2.93 – 2.83 (m, 1H), 2.67 (ddd, J = 16.4, 2.7 Hz, 1H), 2.60 – 2.46 (m, 1H), 1.83 (bs, 1H).

$^{13}C$ NMR ($CDCl_3$, 100 MHz) δ 162.0 (d, $^{1}J_{C-F}$ = 246.5 Hz), 138.9, 132.3, 132.3, 127.1 (d, $^{3}J_{C-F}$ = 7.9 Hz), 122.5, 115.2 (d, $^{2}J_{C-F}$ = 21.4 Hz), 71.7, 43.4, 43.4.

$^{19}F$ NMR ($CDCl_3$, 377 MHz) δ -114.99.

**3-(Thiophen-3-yl)cyclopent-3-en-1-ol (46)**

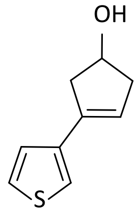


The product was purified by flash chromatography using as eluent a mixture of petroleum ether/diethyl ether (100:5). The product was isolated as a colorless oil (25 mg, 74%).

$^{1}$H NMR ($CDCl_3$, 400 MHz) δ 7.28 (dd, J = 5.0, 2.7 Hz, 1H), 7.25 (d, J = 1.5 Hz, 1H), 7.10 (dt, J = 2.1, 1.0 Hz, 1H), 5.95 (bs, 1H), 4.66 – 4.61 (m, 1H), 3.01 (dd, J = 16.4, 6.3 Hz, 1H), 2.95 – 2.81 (m, 1H), 2.67 (dd, J = 16.4, 2.7 Hz, 1H), 2.50 (dd, J = 17.9, 2.6 Hz, 1H).

$^{13}$C NMR ($CDCl_3$, 100 MHz) δ 138.4, 135.5, 125.7, 125.5, 122.2, 120.4, 71.8, 43.8, 43.3.

**3-(Cyclohex-1-en-1-yl)cyclopent-3-en-1-ol (47)**

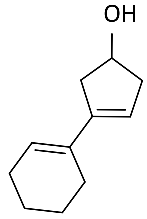


The product was purified by flash chromatography using as eluent a mixture of petroleum ether/diethyl ether (100:5). The product was isolated as a colorless oil (29 mg, 89%).

$^{1}$H NMR ($CDCl_3$, 400 MHz) δ 5.74 – 5.69 (m, 1H), 5.59 – 5.55 (m, 1H), 4.53 (tt, J = 6.2, 2.3 Hz, 1H), 2.76 (ddt, J = 17.4, 6.4, 2.3 Hz, 2H), 2.43 (m, 2H), 2.29 – 2.18 (m, 2H), 2.16 – 2.11 (m, 2H), 1.67 (q, J = 6.4, 6.0 Hz, 2H), 1.63 – 1.50 (m, 2H).

$^{13}$C NMR ($CDCl_3$, 100 MHz) δ 142.2, 133.4, 125.3, 120.2, 71.7, 43.1, 42.3, 29.7, 25.8, 25.7, 22.7, 22.3.

**3-(Naphthalen-2-yl)cyclopent-3-en-1-ol (48)**

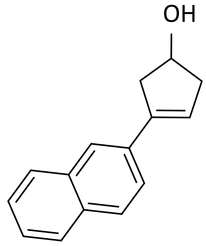


The product was purified by flash chromatography using as eluent a mixture of petroleum ether/diethyl ether (100:5). The product was isolated as a colorless oil (39 mg, 92%).

$^{1}$H NMR ($CDCl_3$, 400 MHz) δ 7.85 – 7.64 (m, 5H), 7.50 – 7.39 (m, 2H), 6.27 (dt, J = 3.9, 1.9 Hz, 1H), 4.72 (bs, 1H), 3.22 – 3.11 (m, 1H), 2.96 (dd, J = 18.0, 6.2 Hz, 1H), 2.85 (dd, J = 16.4, 2.7 Hz, 1H), 2.59 (dd, J = 18.0, 2.8 Hz, 1H).

$^{13}$C NMR ($CDCl_3$, 100 MHz) δ 140.1, 133.5, 132.7, 128.0, 127.8, 127.6, 126.2, 125.7, 124.3, 123.9, 123.7, 71.9, 43.6, 43.3.

**3-(trimethylsilyl)cyclopent-3-en-1-ol (49)**

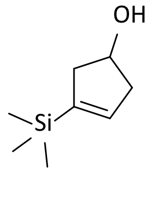


The product was purified by flash chromatography using as eluent a mixture of petroleum ether/diethyl ether (100:5). The product was isolated as a colorless oil (28 mg, 90%).

$^{1}$H NMR ($CDCl_3$, 400 MHz) δ 5.94 (bs, 1H), 4.53 (m, 1H), 2.75 – 2.60 (m, 2H), 2.46 – 2.29 (m, 2H), 0.09 (s, 9H)

$^{13}$C NMR ($CDCl_3$, 100 MHz) δ 146.1, 135.9, 72.2, 45.9, 38.7, 0.0

**3-(2-Bromophenyl)cyclopent-3-en-1-ol (51)**

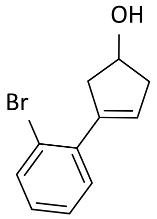


The product was purified by flash chromatography using as eluent a mixture of petroleum ether/diethyl ether (100:5). The product was isolated as a colorless oil (46 mg, 96%).

$^{1}$H NMR ($CDCl_3$, 400 MHz) δ 7.56 (dd, J = 8.0, 1.1 Hz, 1H), 7.29 – 7.21 (m, 2H), 7.11 (m, 1H), 5.91 (bs, 1H), 4.63 (m, 1H), 3.15 – 3.03 (m, 1H), 2.87 (dd, J = 17.5, 5.8 Hz, 1H), 2.75 (dd, J = 16.6, 2.7 Hz, 1H), 2.53 (dd, J = 17.5, 2.6 Hz, 1H).

$^{13}$C NMR ($CDCl_3$, 100 MHz) δ 140.8, 133.1, 130.1, 128.4, 128.1, 127.1, 125.6, 122.2, 72.3, 46.0, 43.3.

## -Characterization data for bicycles

**4,4,5,5-tetramethyl-2-((1S,3R,5S)-1-phenylbicyclo[3.1.0]hexan-3-yl)-1,3,2-dioxaborolane and 4,4,5,5-tetramethyl-2-((1S,3S,5S)-1-phenylbicyclo[3.1.0]hexan-3-yl)-1,3,2-dioxaborolane (54)**

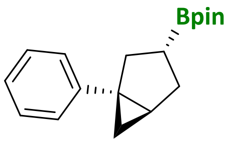


The products were purified by flash chromatography using as eluent a mixture of petroleum ether/diethyl ether (100:5). The products were isolated as a colorless oil (52 mg, 92%).

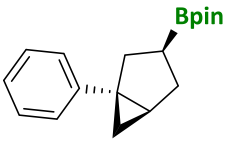


**54a**: $^{1}$H NMR ($CDCl_3$, 400 MHz) δ 7.27 – 7.23 (m, 2H), 7.18 (ddd, J = 8.3, 3.4, 1.5 Hz, 2H), 7.16 – 7.09 (m, 1H), 2.35 – 2.25 (m, 1H), 2.22 – 2.13 (m, 1H), 2.09 – 1.99 (m, 1H), 1.69 – 1.58 (m, 2H), 1.24 (s, 12H), 0.97 – 0.92 (m, 1H), 0.75 – 0.70 (m, 1H).

**54b:** $^{1}$H NMR ($CDCl_3$, 400 MHz) δ 7.27 – 7.23 (m, 2H), 7.18 (ddd, J = 8.3, 3.4, 1.5 Hz, 2H), 7.16 – 7.09 (m, 1H), 2.35 – 2.25 (m, 1H), 2.22 – 2.13 (m, 1H), 2.09 – 1.99 (m, 1H), 1.69 – 1.58 (m, 2H), 1.26 (s, 12H), 0.97 – 0.92 (m, 1H), 0.75 – 0.70 (m, 1H).

$^{13}$C NMR ($CDCl_3$, 100 MHz) δ 145.8, 145.6, 129.4, 128.7, 128.1, 128.0, 128.0, 127.4, 126.1, 125.6, 125.0, 83.2, 83.0, 34.3, 34.0, 32.6, 32.5, 30.2, 29.8, 27.2, 27.1, 24.8, 24.7, 18.4, 16.4.

$^{11}$B NMR ($CDCl_3$, 128.3 MHz) δ 35.0.

HRMS-(ESI+) for $C_{18}H_{26}BO_2$ $[M+H]^+$: calculated: 285.2026; found: 285.2031.

**(1R,3S,5R)-1-phenylbicyclo[3.1.0]hexan-3-ol (55-*syn*)**

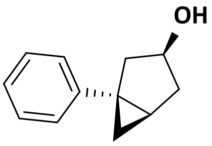


The product was purified by flash chromatography using as eluent a mixture of petroleum ether/diethyl ether (100:5). The product was isolated as a colorless oil (18 mg, 52% from **54**) (33 mg, 95% from **41**).

$^{1}$H NMR ($CDCl_3$, 400 MHz) δ 7.44 – 7.32 (m, 2H), 7.30 – 7.20 (m, 3H), 4.61 (m, 1H), 2.52 (dd, J = 13.9, 6.5 Hz, 1H), 2.47 – 2.36 (m, 1H), 2.27 (d, J = 14.0 Hz, 1H), 1.98 (d, J = 14.2 Hz, 1H), 1.80 (dt, J = 8.9, 4.7 Hz, 1H), 1.72 – 1.66 (m, 1H), 1.48 (t, J = 4.5 Hz, 1H), 1.10 – 1.01 (m, 1H).

$^{13}C$ NMR ($CDCl_3$, 100 MHz) δ 145.3, 128.2, 126.0, 125.3, 73.2, 42.7, 38.7, 32.6, 26.8, 21.9.

**(1R,3R,5R)-1-phenylbicyclo[3.1.0]hexan-3-ol (55-*anti*)**

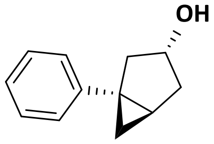

The product was purified by flash chromatography using as eluent a mixture of petroleum ether/diethyl ether (100:5). The product was isolated as a colorless oil (9 mg, 26%).

$^{1}H$ NMR ($CDCl_3$, 400 MHz) δ 7.34 – 7.25 (m, 2H), 7.16 (m, 3H), 4.16 (m, 1H), 2.58 (dd, J = 12.3, 7.0 Hz, 1H), 2.31 (dd, J = 12.4, 7.1 Hz, 1H), 2.11 – 2.00 (m, 1H), 1.94 – 1.81 (m, 1H), 1.66 (dt, J = 8.3, 4.7 Hz, 1H), 1.42 (d, J = 12.9 Hz, 1H), 1.32 – 1.20 (m, 1H), 0.82 – 0.74 (m, 1H).

$^{13}C$ NMR ($CDCl_3$, 100 MHz) δ 145.7, 128.2, 125.7, 125.4, 71.1, 41.2, 37.1, 29.7, 24.8, 19.2.

**(1R,3S,5R)-1-(4-methoxyphenyl)bicyclo[3.1.0]hexan-3-ol (56)**

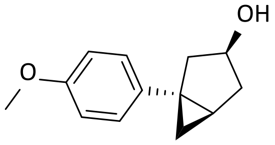

The product was purified by flash chromatography using as eluent a mixture of petroleum ether/diethyl ether (100:5). The product was isolated as a colorless oil (40 mg, 98%).

$^{1}H$ NMR ($CDCl_3$, 400 MHz) δ 7.15 – 7.07 (m, 2H), 6.87 – 6.79 (m, 2H), 4.55 – 4.48 (m, 1H), 3.79 (s, 3H), 2.43 – 2.26 (m, 2H), 2.15 (d, J = 14.0 Hz, 1H), 1.87 (d, J = 14.1 Hz, 1H), 1.67 – 1.56 (m, 1H), 1.49 (s, 1H), 1.31 (t, J = 4.4 Hz, 1H), 0.91 (m, 1H).

$^{13}C$ NMR ($CDCl_3$, 100 MHz) δ 157.5, 137.3, 127.4, 113.7, 73.4, 55.3, 43.5, 38.8, 32.2, 26.2, 21.0.

**(1R,3S,5R)-1-(4-fluorophenyl)bicyclo[3.1.0]hexan-3-ol (57)**

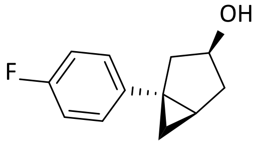

The product was purified by flash chromatography using as eluent a mixture of petroleum ether/diethyl ether (100:5). The product was isolated as a colorless oil (34 mg, 89%).

$^{1}H$ NMR ($CDCl_3$, 400 MHz) δ 7.19 – 7.07 (m, 2H), 7.00 – 6.90 (m, 2H), 4.52 (m, 1H), 2.42 – 2.28 (m, 2H), 2.16 (d, J = 13.9 Hz, 1H), 1.88 (d, J = 14.2 Hz, 1H), 1.72 – 1.61 (m, 1H), 1.51 (s, 2H), 1.36 (t, J = 4.5 Hz, 1H), 0.91 (m, 1H).

$^{13}C$ NMR ($CDCl_3$, 100 MHz) δ160.9 (d, *J* = 243.3 Hz), 140.9, 140.8, 127.7 (d, *J* = 7.8 Hz), 114.9 (d, *J* = 21.1 Hz), 73.2, 43.2, 38.7, 32.2, 26.5, 21.4.

$^{19}F$ NMR ($CDCl_3$, 377 MHz) δ -117.34.

**(1R,3S,5R)-1-(o-tolyl)bicyclo[3.1.0]hexan-3-ol (58)**

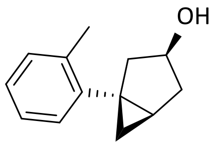


The product was purified by flash chromatography using as eluent a mixture of petroleum ether/diethyl ether (100:5). The product was isolated as a colorless oil (33 mg, 88%).

$^{1}$H NMR ($CDCl_3$, 400 MHz) δ 7.31 – 7.22 (m, 1H), 7.17 – 7.07 (m, 3H), 4.53 (m, 1H), 2.46 (m, 1H), 2.40 (s, 3H), 2.23 – 2.06 (m, 2H), 1.93 (d, J = 14.3 Hz, 1H), 1.60 (d, J = 8.8 Hz, 1H), 1.28 (d, J = 8.2 Hz, 1H), 0.87 (m, 1H).

$^{13}$C NMR ($CDCl_3$, 100 MHz) δ 142.4, 138.2, 130.1, 129.4, 126.4, 125.7, 73.8, 44.5, 39.0, 33.3, 24.9, 19.5, 18.7.

**(1R,3S,5R)-1-(2-bromophenyl)bicyclo[3.1.0]hexan-3-ol (59)**

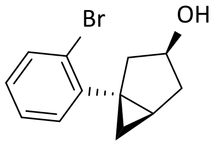


The product was purified by flash chromatography using as eluent a mixture of petroleum ether/diethyl ether (100:5). The product was isolated as a colorless oil (42 mg, 83%).

$^{1}$H NMR ($CDCl_3$, 400 MHz) δ 7.45 (d, J = 7.9 Hz, 1H), 7.28 (d, J = 7.6 Hz, 1H), 7.19 – 7.12 (m, 1H), 7.00 (d, J = 7.6 Hz, 1H), 4.51 – 4.43 (m, 1H), 2.47 (m, 1H), 2.29 (m, 1H), 1.99 (d, J = 14.1 Hz, 1H), 1.85 (d, J = 14.1 Hz, 1H), 1.54 (dd, J = 8.8, 4.5 Hz, 1H), 1.27 (m, 1H), 0.92 – 0.82 (m, 1H).

$^{13}$C NMR ($CDCl_3$, 100 MHz) δ 143.2, 132.8, 132.2, 128.2, 128.0, 127.2, 126.0, 73.7, 43.9, 39.0, 35.2, 25.9, 18.6.

**(1R,3S,5R)-1-(naphthalen-2-yl)bicyclo[3.1.0]hexan-3-ol (60)**

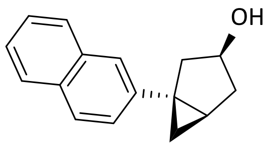


The product was purified by flash chromatography using as eluent a mixture of petroleum ether/diethyl ether (100:5). The product was isolated as a colorless oil (43 mg, 96%).

$^{1}$H NMR ($CDCl_3$, 400 MHz) δ 7.85 – 7.72 (m, 3H), 7.64 (d, J = 1.9 Hz, 1H), 7.43 (m, 2H), 7.29 – 7.22 (m, 1H), 4.62 – 4.54 (m, 1H), 2.55 (dd, J = 14.0, 6.6 Hz, 1H), 2.39 (dd, J = 14.2, 6.2 Hz, 1H), 2.29 (d, J = 14.0 Hz, 1H), 1.95 (d, J = 14.2 Hz, 1H), 1.83 (m, 1H), 1.47 (t, J = 4.6 Hz, 1H), 1.09 (m, 1H).

$^{13}$C NMR ($CDCl_3$, 100 MHz) δ 142.6, 133.4, 131.7, 127.8, 127.5, 127.3, 126.0, 125.0, 124.9, 124.3, 73.3, 42.9, 38.7, 33.0, 26.9, 21.7.

**(1R,3S,5R)-1-(thiophen-3-yl)bicyclo[3.1.0]hexan-3-ol (61)**

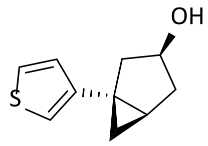


The product was purified by flash chromatography using as eluent a mixture of petroleum ether/diethyl ether (100:5). The product was isolated as a colorless oil (33 mg, 92%).

$^1$H NMR (CDCl$_3$, 400 MHz) δ 7.26 – 7.22 (m, 1H), 6.90 (dd, J = 3.0, 1.4 Hz, 1H), 6.80 (dd, J = 5.1, 1.4 Hz, 1H), 4.55 – 4.48 (m, 1H), 2.46 (dd, J = 14.0, 6.5 Hz, 1H), 2.37 – 2.25 (m, 1H), 2.14 (d, J = 14.0 Hz, 1H), 1.85 (d, J = 13.9 Hz, 1H), 1.62 (m, 1H), 1.37 (t, J = 4.6 Hz, 1H), 1.00 (m, 1H).

$^{13}$C NMR (CDCl$_3$, 100 MHz) δ 142.1, 125.8, 124.7, 118.1, 73.2, 41.9, 38.6, 31.5, 27.7, 21.4.

**(1R,3S,5R)-1-(cyclohex-1-en-1-yl)bicyclo[3.1.0]hexan-3-ol (62)**

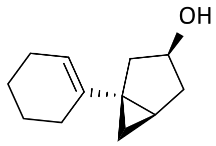


The product was purified by flash chromatography using as eluent a mixture of petroleum ether/diethyl ether (100:5). The product was isolated as a colorless oil (32 mg, 90%).

$^1$H NMR (CDCl$_3$, 400 MHz) δ 5.47 (bs, 1H), 4.40 (t, J = 6.6 Hz, 1H), 2.24 – 2.14 (m, 2H), 2.00 (bs, 2H), 1.85 – 1.72 (m, 4H), 1.57 (m, 4H), 1.37 (m, 1H), 0.93 (t, J = 4.4 Hz, 1H), 0.80 – 0.71 (m, 1H).

$^{13}$C NMR (CDCl$_3$, 100 MHz) δ 139.2, 120.0, 73.2, 41.6, 38.5, 34.6, 26.1, 25.3, 23.5, 23.0, 22.6, 17.3.

# $^{1}H$, $^{13}C$ and $^{11}B$ Spectra of borylated dienes

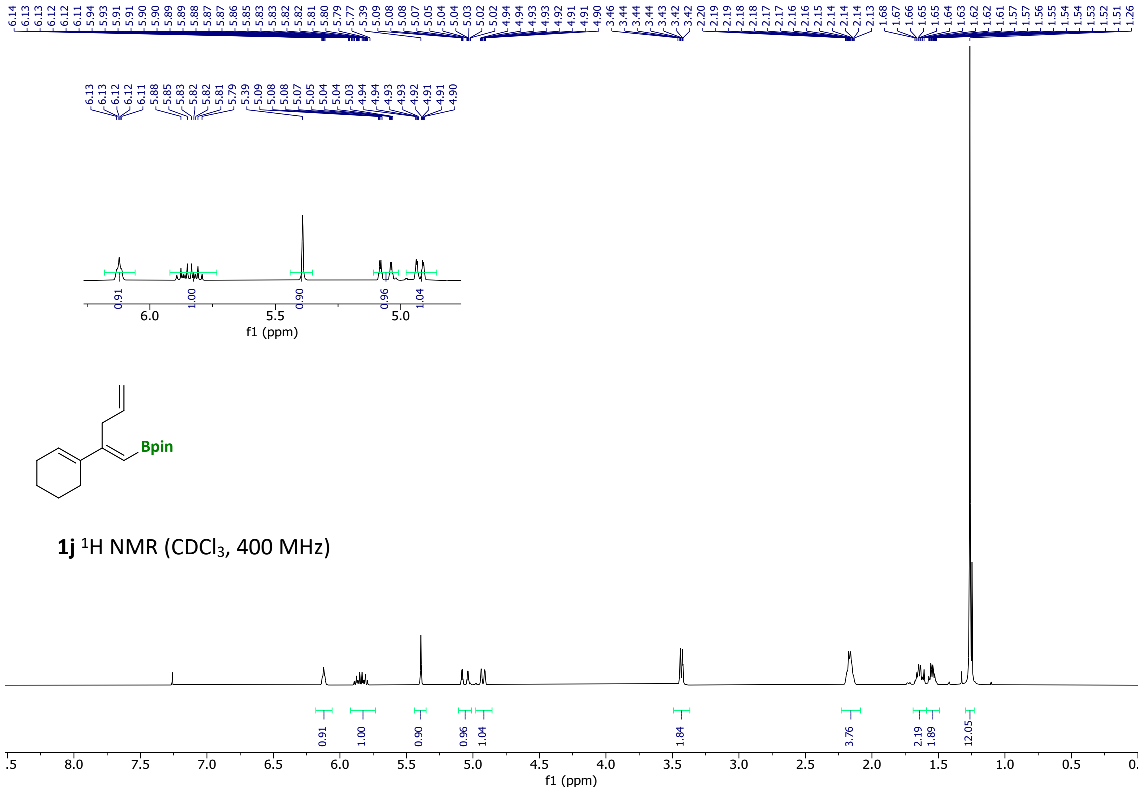


**1j** $^{1}H$ NMR ($CDCl_3$, 400 MHz)

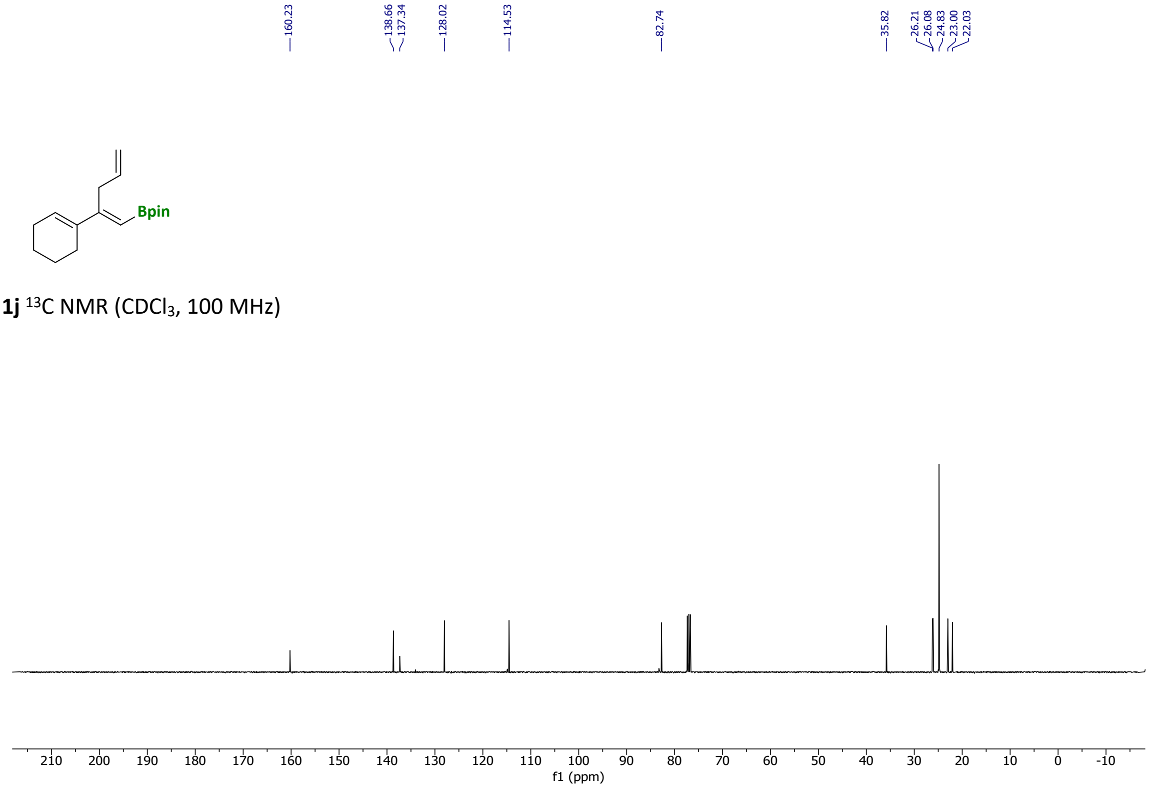


**1j** $^{13}C$ NMR ($CDCl_3$, 100 MHz)

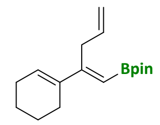

**1j** $^{11}$B NMR ($CDCl_3$, 128.3 MHz)

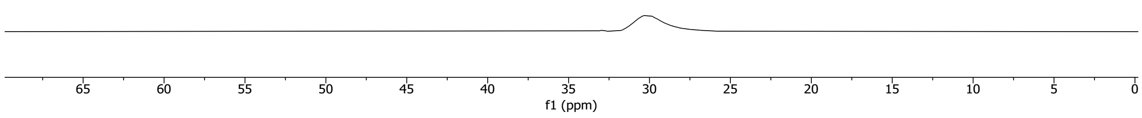


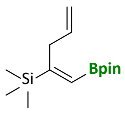

**1k** $^{1}$H NMR ($CDCl_3$, 400 MHz)

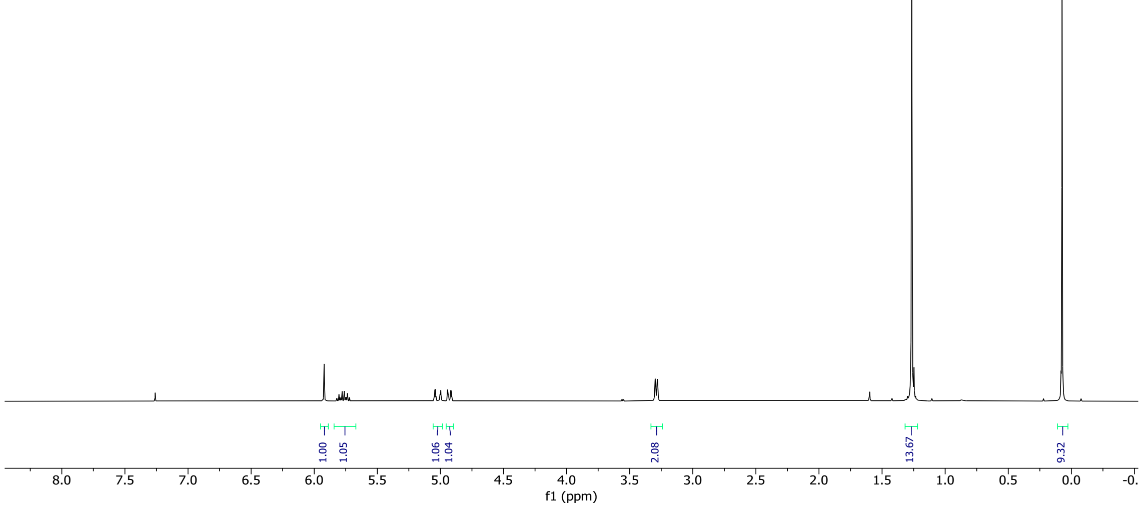

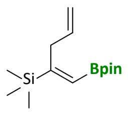

**1k** $^{13}C$ NMR ($CDCl_3$, 100 MHz)

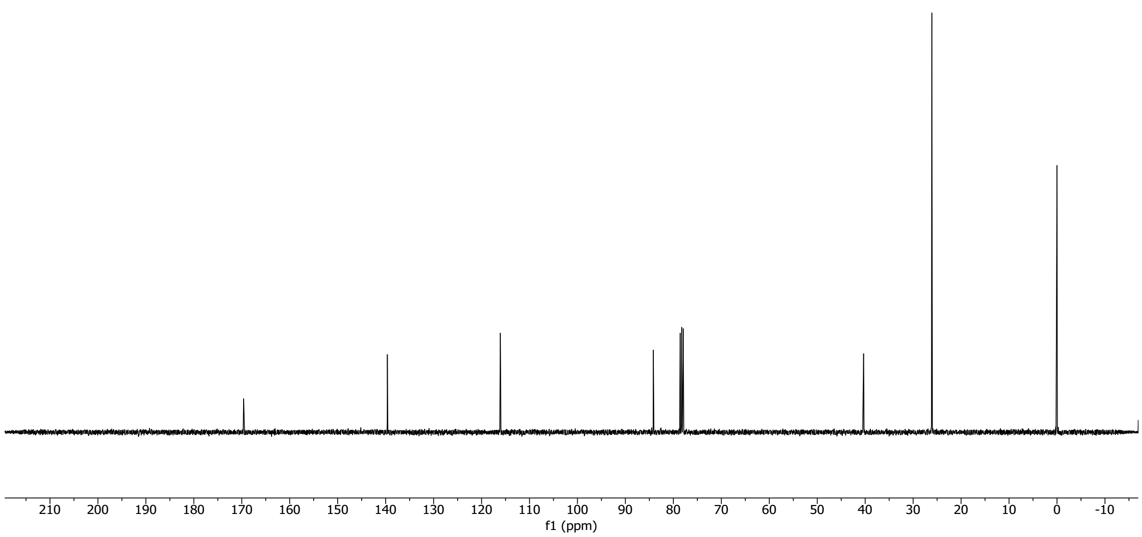


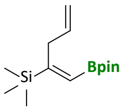

**1k** $^{11}B$ NMR ($CDCl_3$, 128.3 MHz)

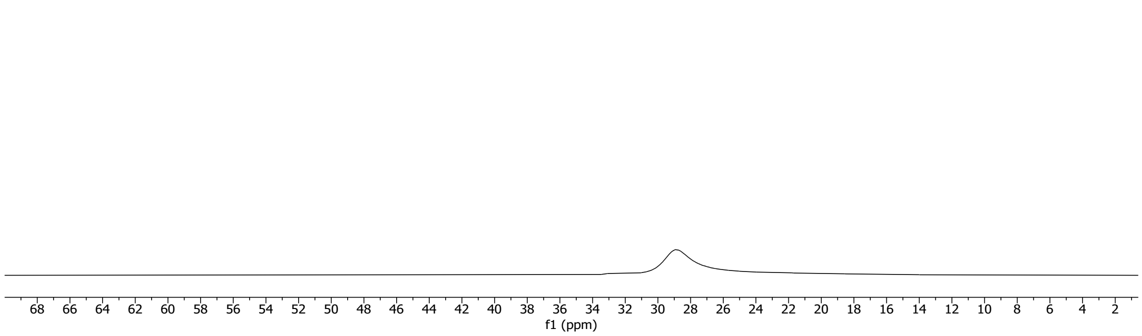

**1l** $^{1}$H NMR (CDCl$_3$, 400 MHz)

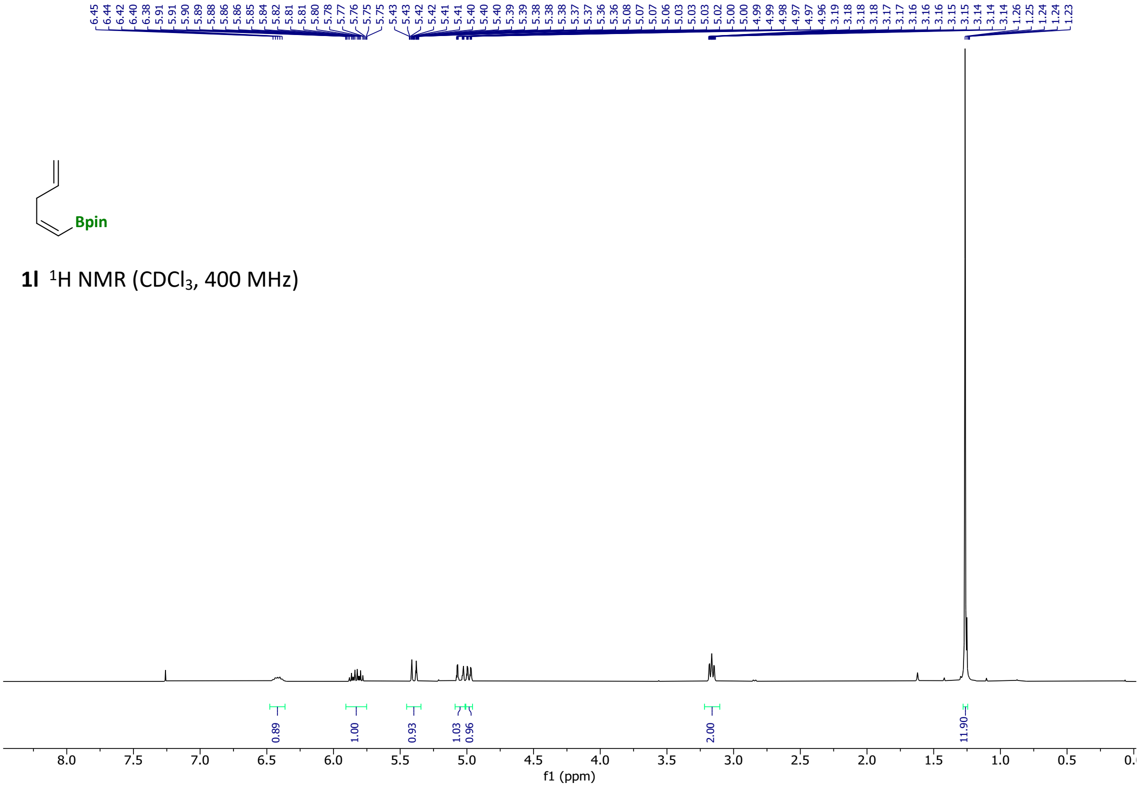


**1l** $^{13}$C NMR (CDCl$_3$, 100 MHz)

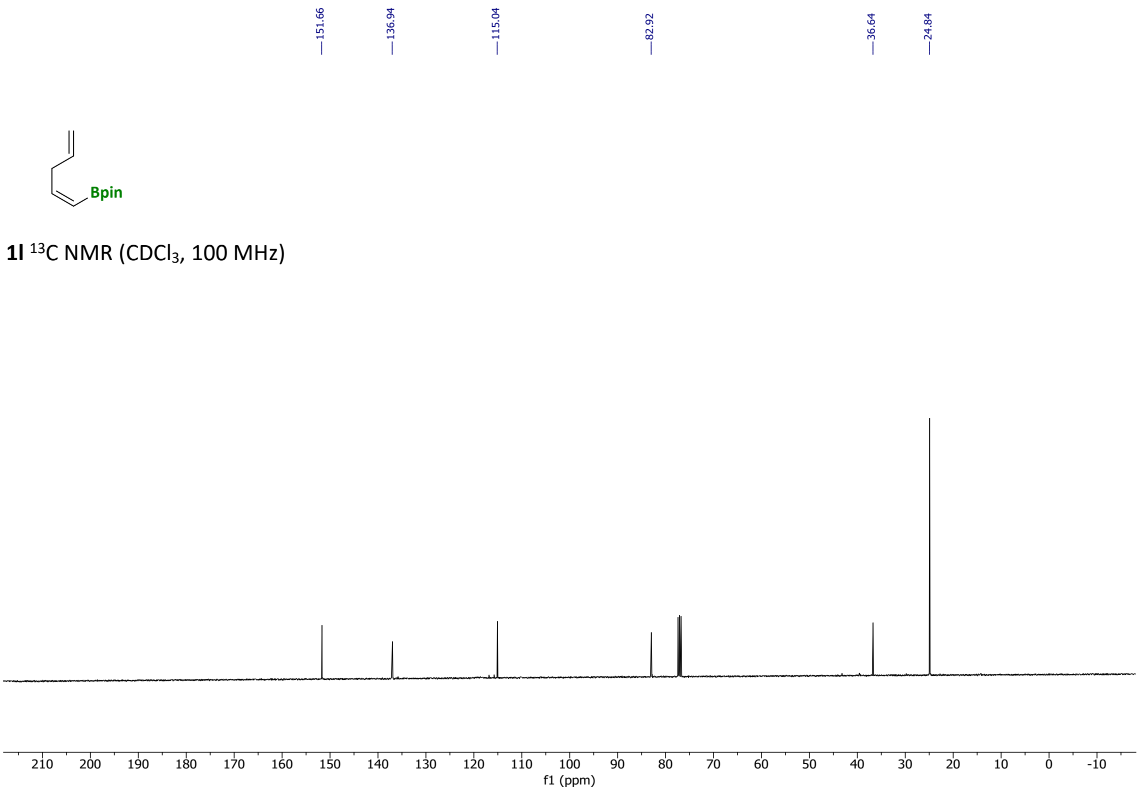

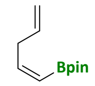


**1l** $^{11}$B NMR ($CDCl_3$, 128.3 MHz)

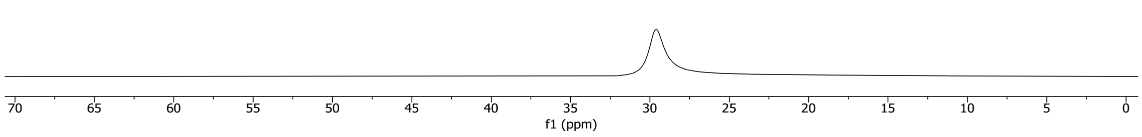

## -$^{1}$H, $^{13}$C, $^{11}$B Spectra for products isolated from Cu-catalyzed borylcupration/1,4-B/Cu migration/ protonation (or iodination or bromination)

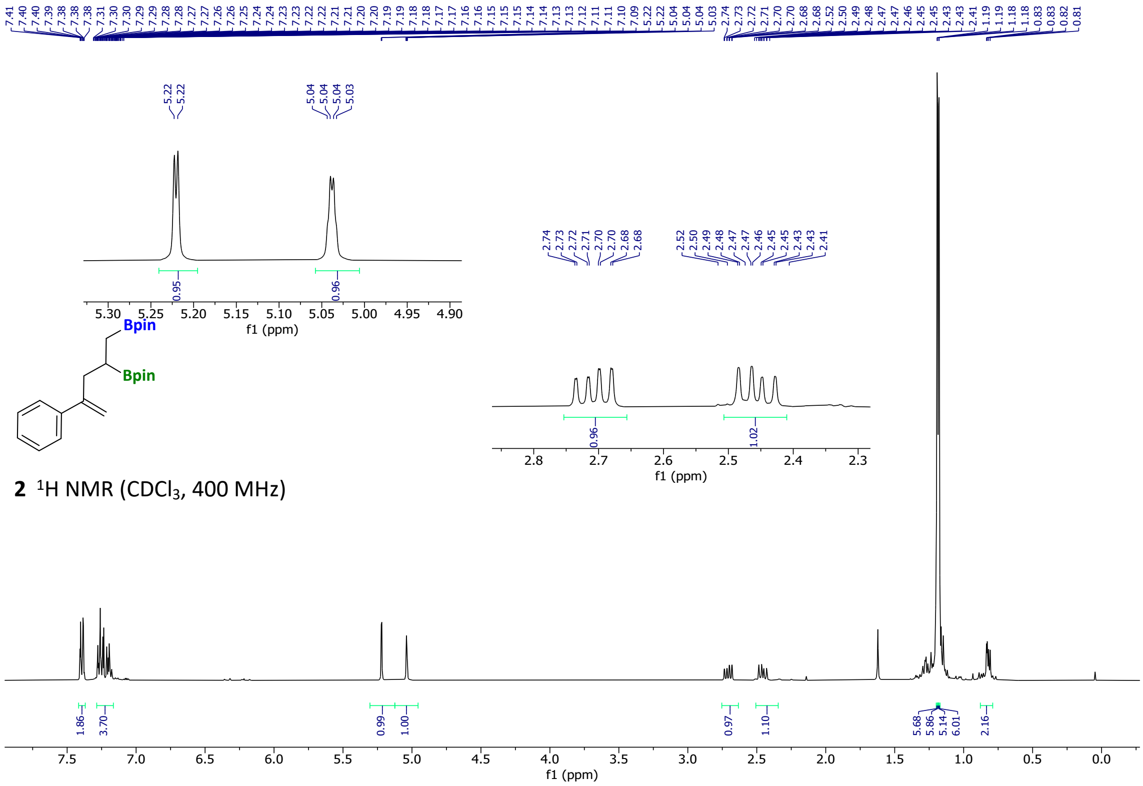


**2** $^{1}$H NMR ($CDCl_3$, 400 MHz)

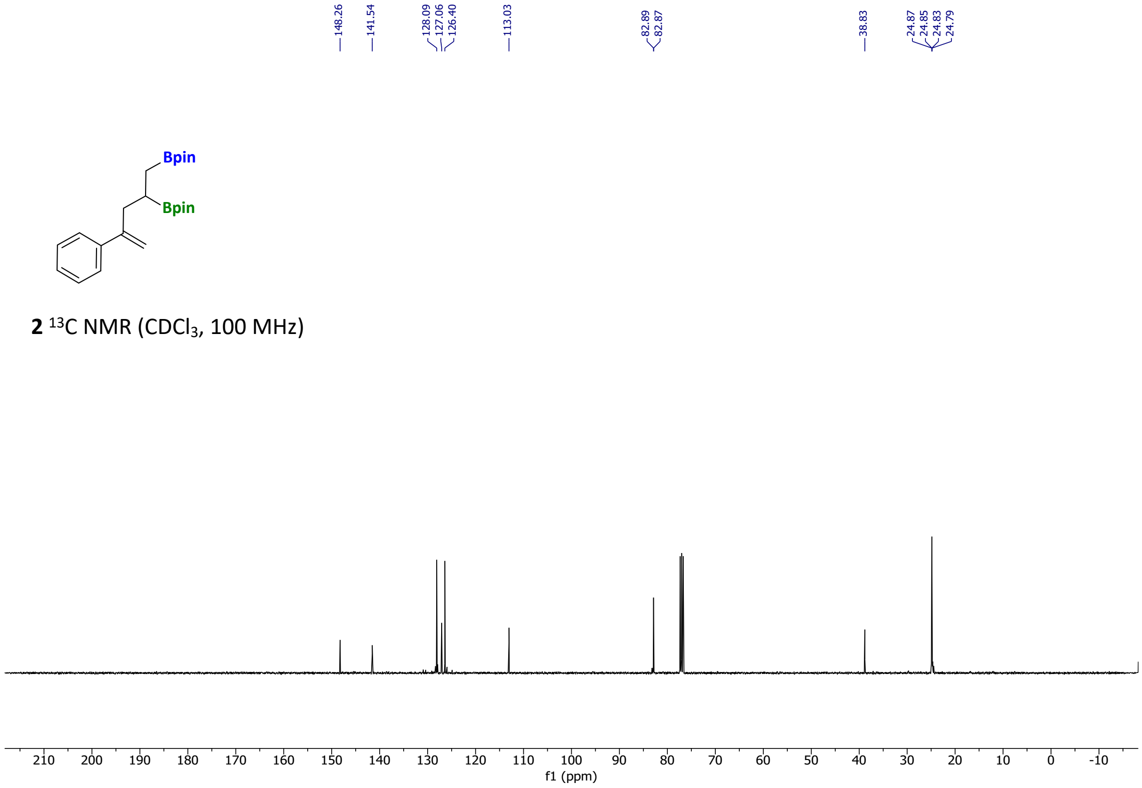


**2** $^{13}$C NMR ($CDCl_3$, 100 MHz)

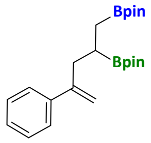

**2** $^{11}$B NMR ($CDCl_3$, 128.3 MHz)

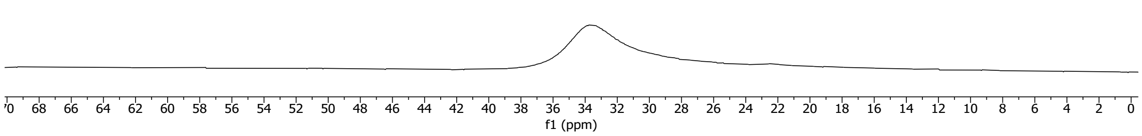


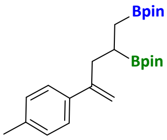

**3** $^{1}$H NMR ($CDCl_3$, 400 MHz)

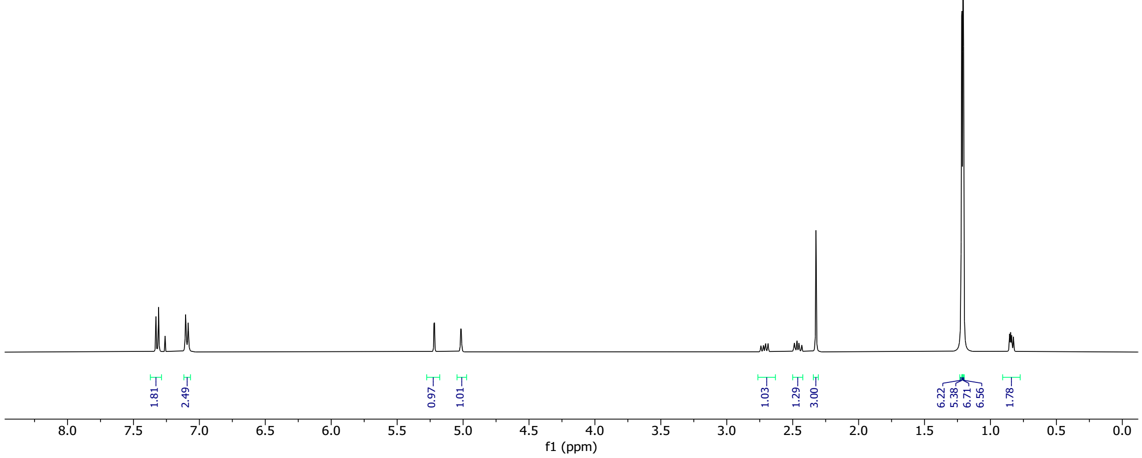

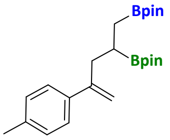


**3** $^{13}C$ NMR ($CDCl_3$, 100 MHz)

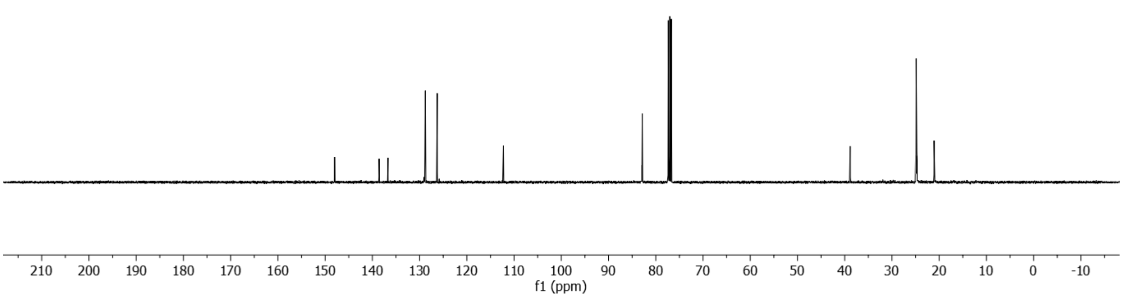


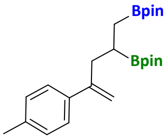


**3** $^{11}B$ NMR ($CDCl_3$, 128.3 MHz)

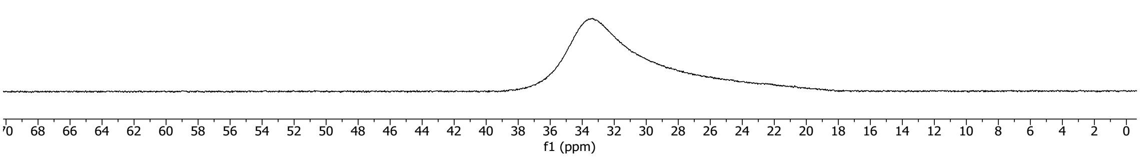

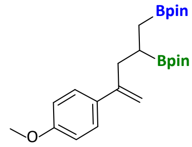

**4** $^{1}$H NMR ($CDCl_3$, 400 MHz)

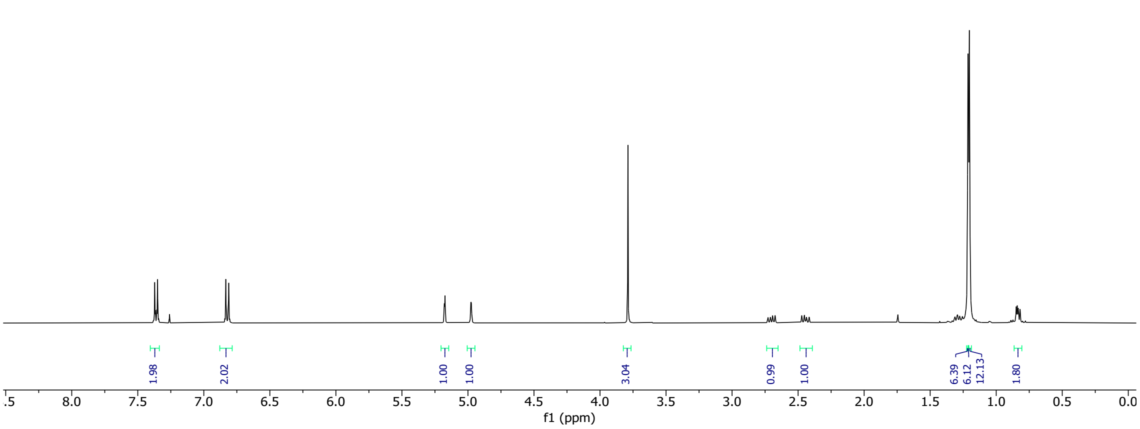


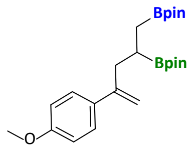

**4** $^{13}$C NMR ($CDCl_3$, 100 MHz)

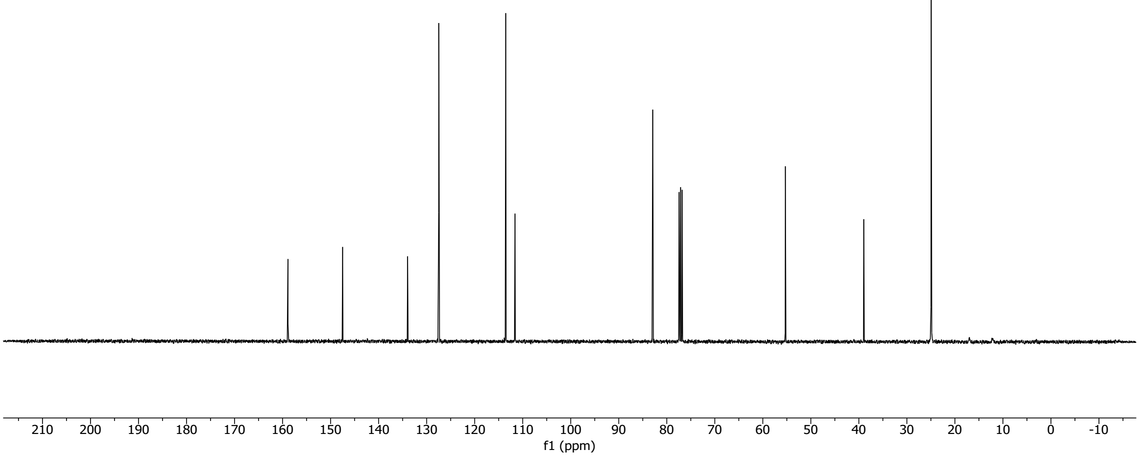

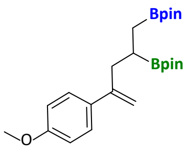

**4** $^{11}$B NMR ($CDCl_3$, 128.3 MHz)

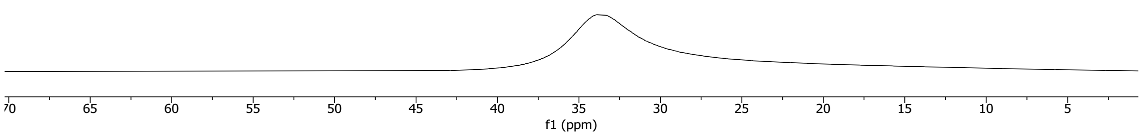


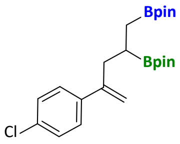

**5** $^{1}$H NMR ($CDCl_3$, 400 MHz)

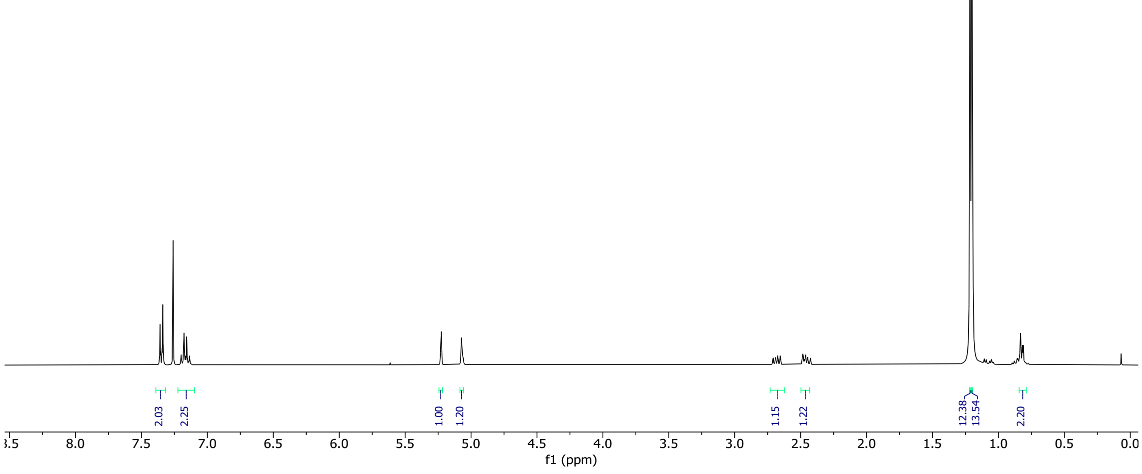

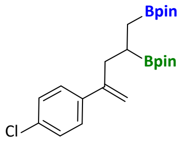


**5** $^{13}$C NMR ($CDCl_3$, 100 MHz)

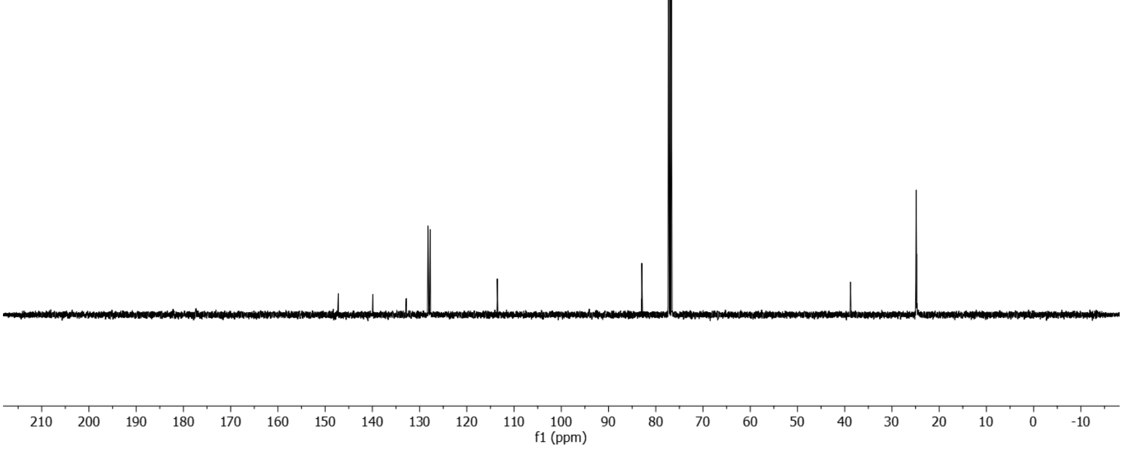


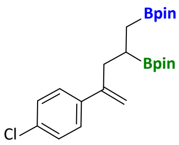


**5** $^{11}$B NMR ($CDCl_3$, 128.3 MHz)

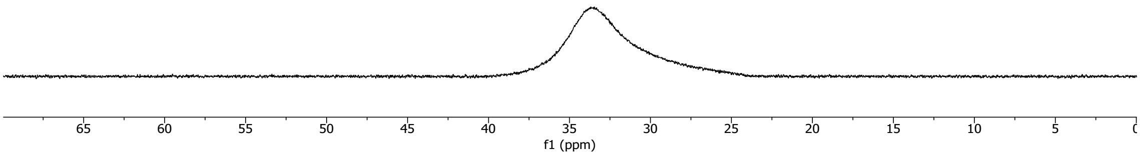

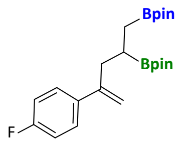


**6** $^{1}$H NMR ($CDCl_3$, 400 MHz)

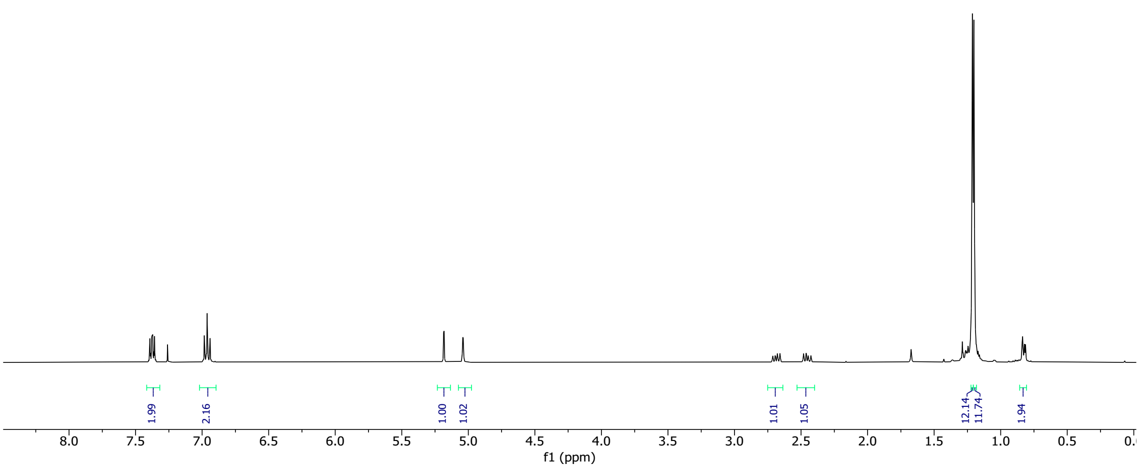


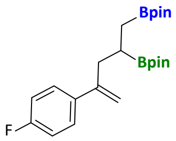


**6** $^{13}$C NMR ($CDCl_3$, 100 MHz)

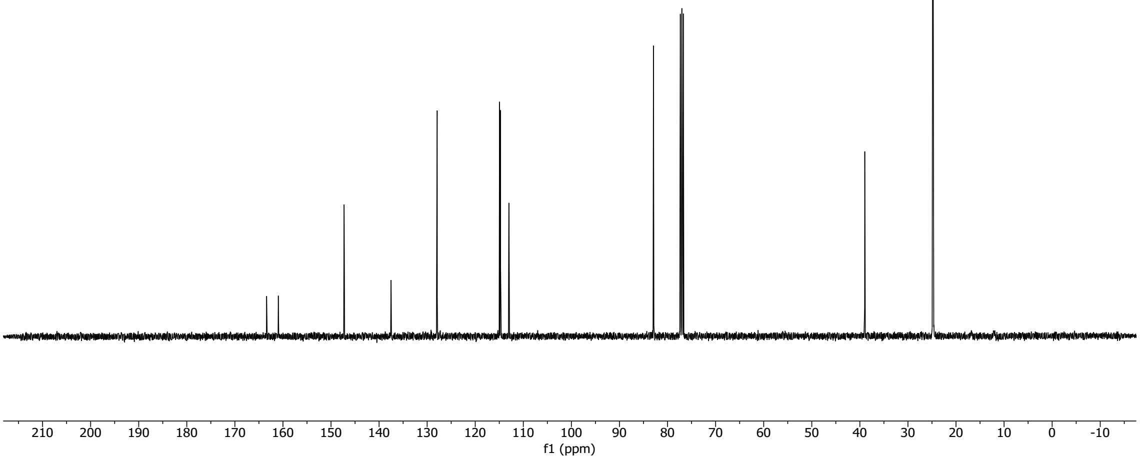

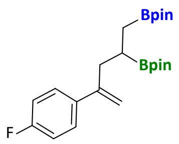


**6** $^{11}$B NMR ($CDCl_3$, 128.3 MHz)

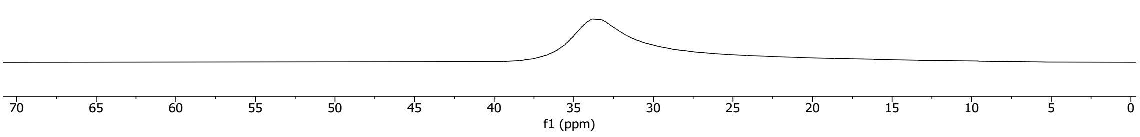


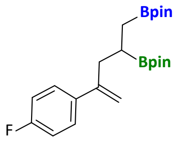


**6** $^{19}$F NMR ($CDCl_3$, 377 MHz)

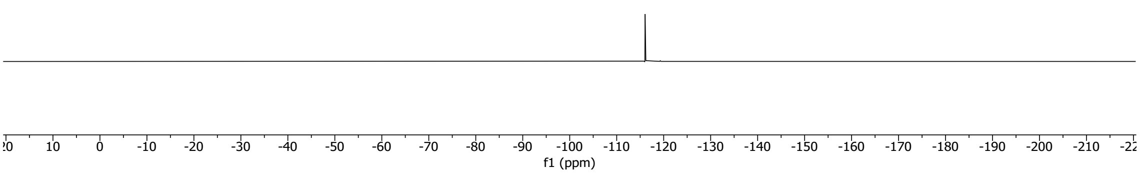

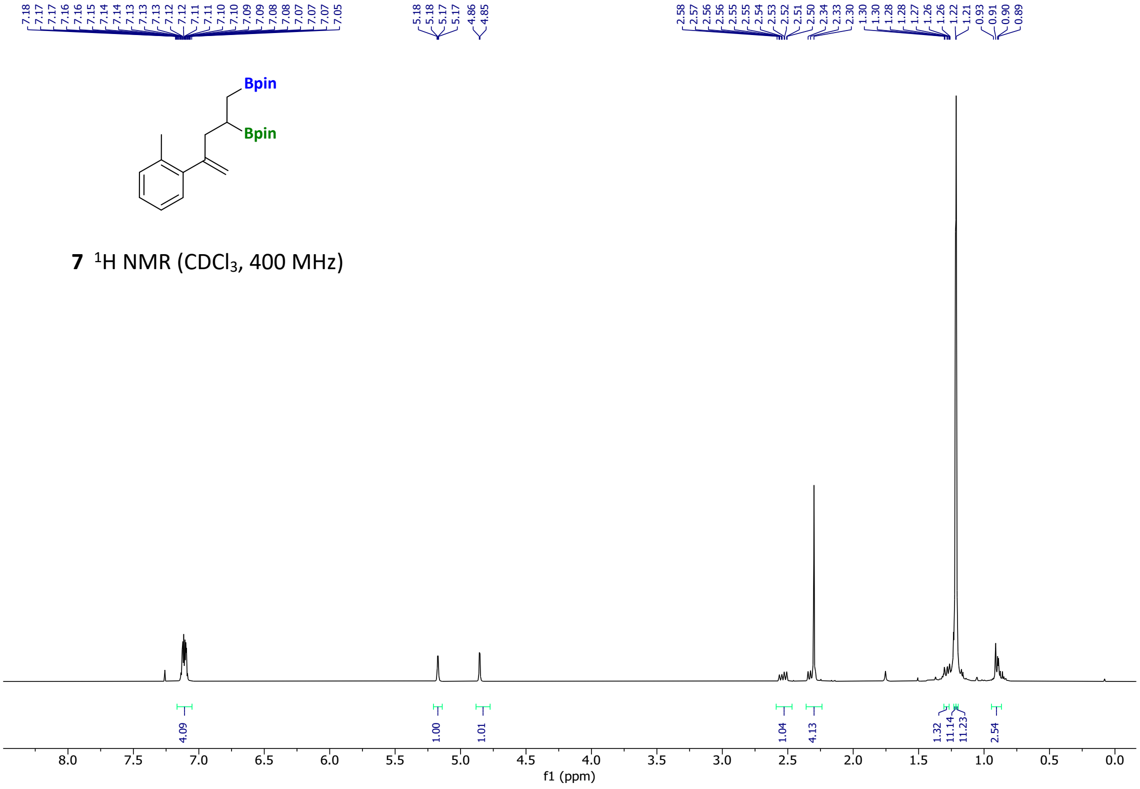
7.18 7.17 7.17 7.16 7.16 7.15 7.14 7.14 7.13 7.13 7.12 7.12 7.11 7.11 7.10 7.10 7.09 7.09 7.08 7.08 7.07 7.07 7.07 7.05
5.18 5.18 5.17 5.17 4.86 4.85
2.58 2.57 2.56 2.56 2.55 2.55 2.54 2.53 2.52 2.51 2.50 2.34 2.33 2.30 1.30 1.30 1.28 1.28 1.27 1.26 1.26 1.22 1.21 0.93 0.91 0.90 0.89
Bpin
Bpin
7 1H NMR (CDCl3, 400 MHz)
4.09
1.00
1.01
1.04
4.13
1.32
11.14
11.23
2.54
8.0 7.5 7.0 6.5 6.0 5.5 5.0 4.5 4.0 3.5 3.0 2.5 2.0 1.5 1.0 0.5 0.0
f1 (ppm)

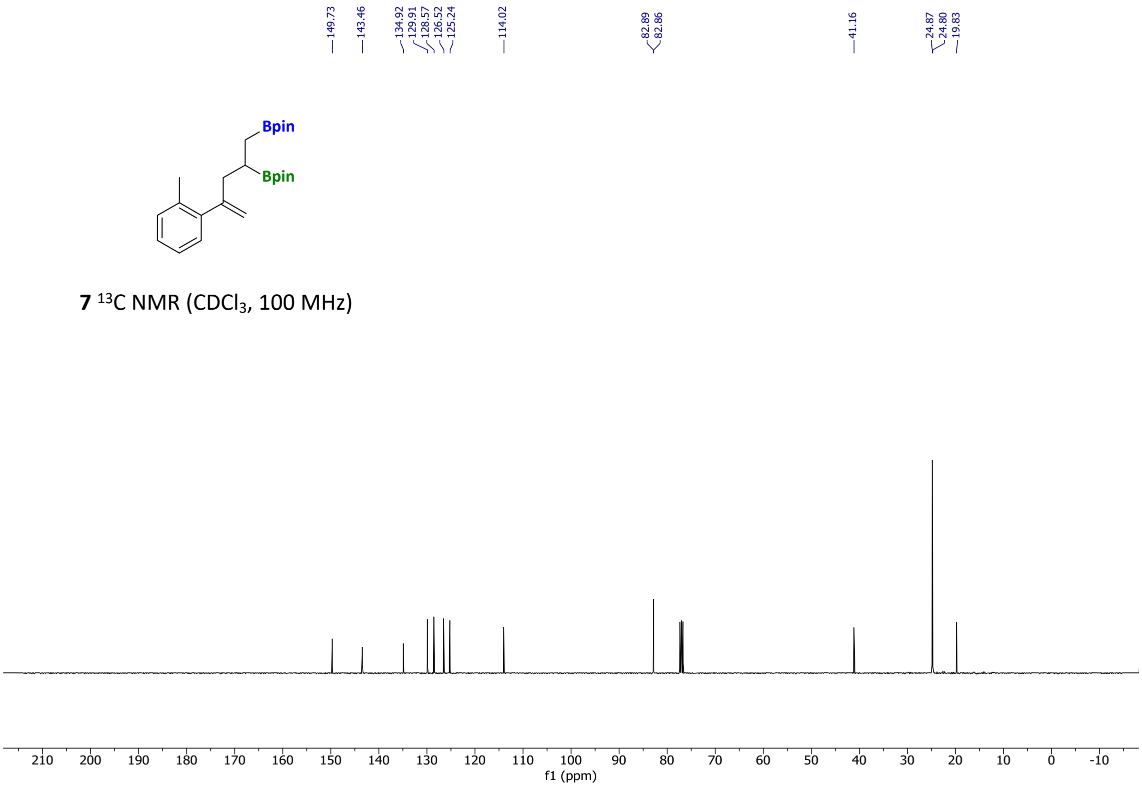
149.73
143.46
134.92 129.91 128.57 126.52 125.24
114.02
82.89 82.86
41.16
24.87 24.80 19.83
Bpin
Bpin
7 13C NMR (CDCl3, 100 MHz)
210 200 190 180 170 160 150 140 130 120 110 100 90 80 70 60 50 40 30 20 10 0 -10
f1 (ppm)

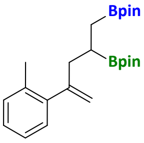

**7** $^{11}B$ NMR ($CDCl_3$, 128.3 MHz)

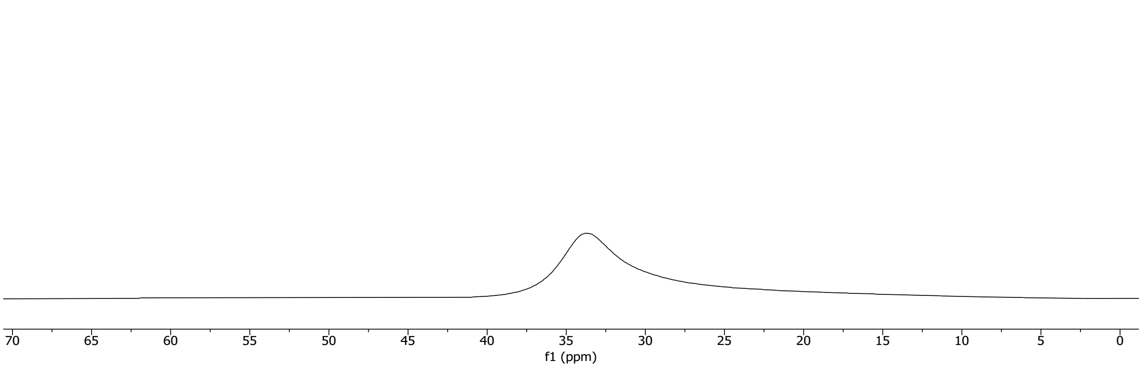

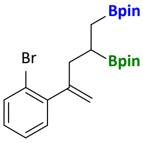

**8** $^{1}H$ NMR ($CDCl_3$, 400 MHz)

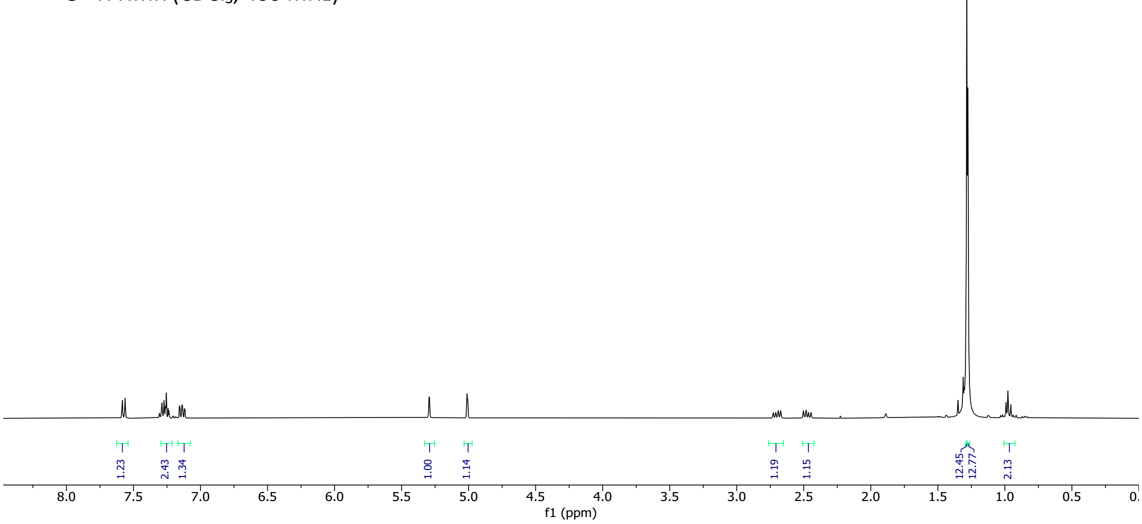

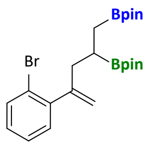


**8** $^{13}$C NMR ($CDCl_3$, 100 MHz)

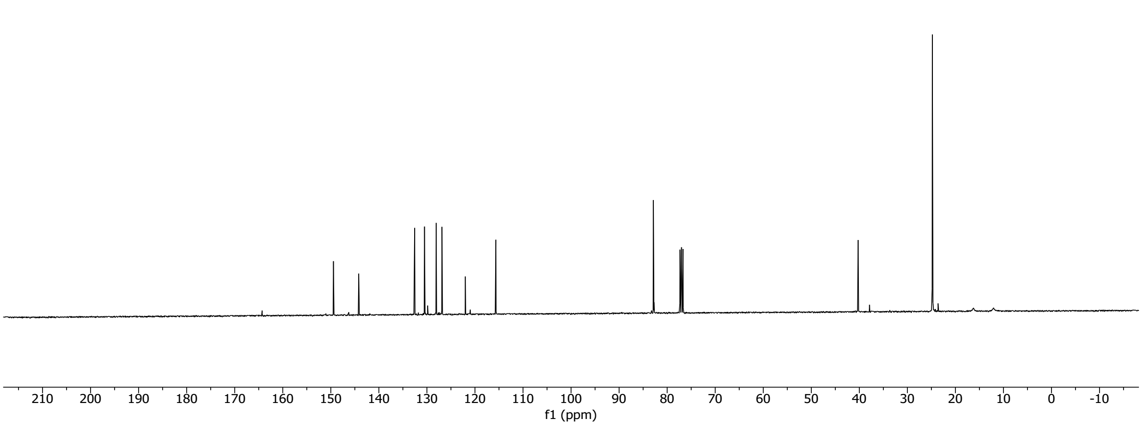


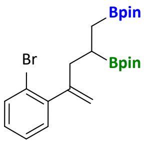


**8** $^{11}$B NMR ($CDCl_3$, 128.3 MHz)

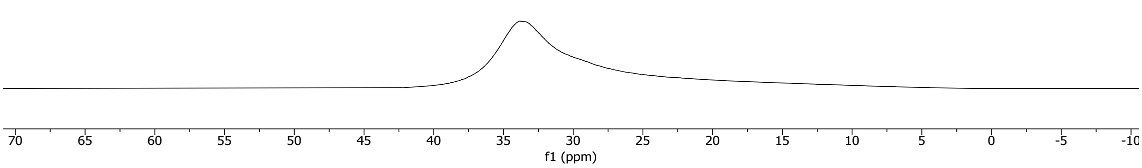

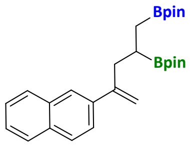


**9** $^{1}$H NMR ($CDCl_3$, 400 MHz)

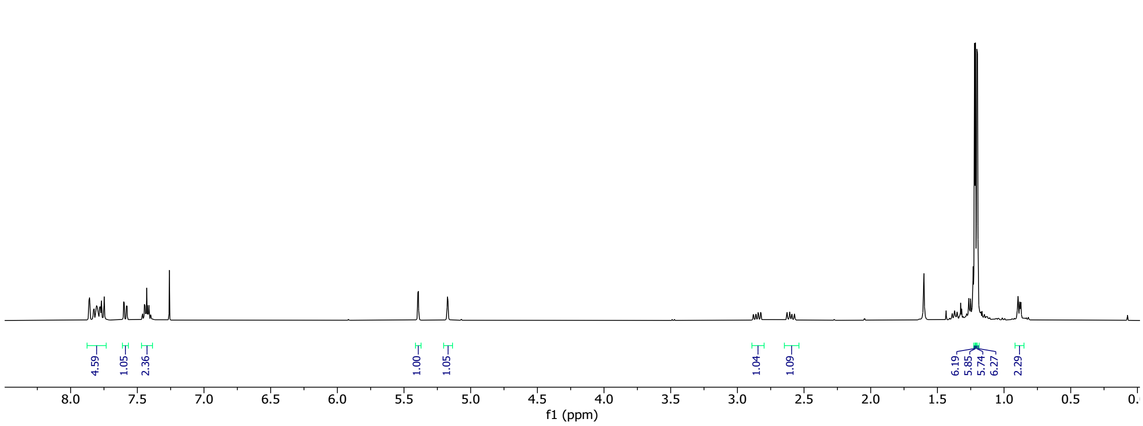


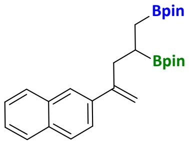


**9** $^{13}$C NMR ($CDCl_3$, 100 MHz)

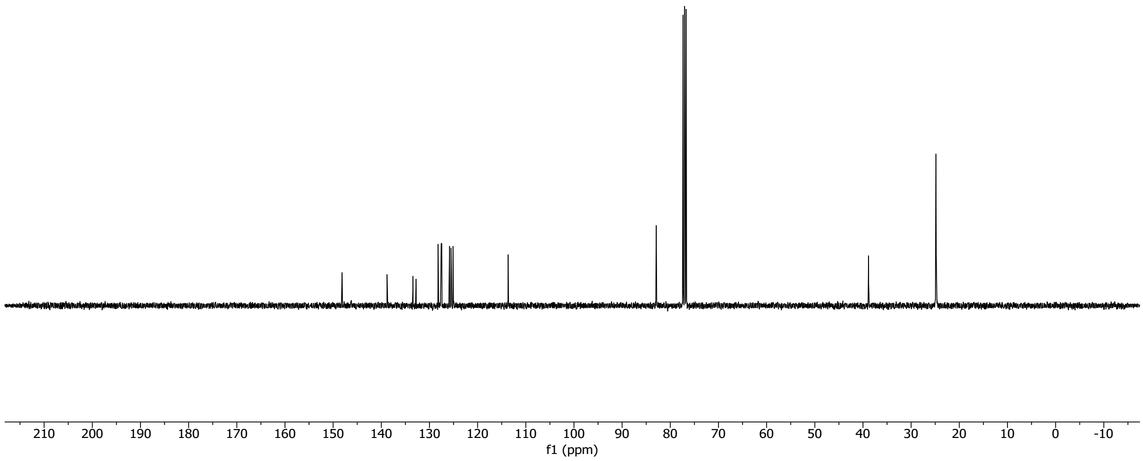

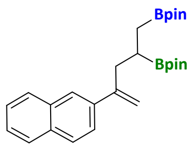

**9** $^{11}$B NMR ($CDCl_3$, 128.3 MHz)

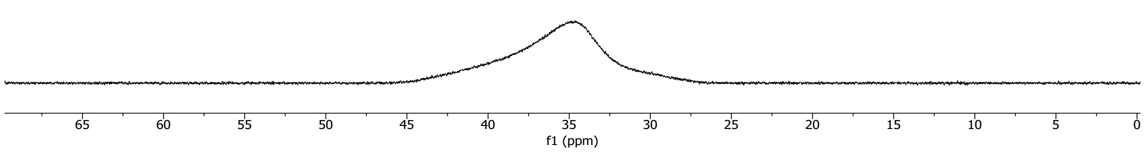


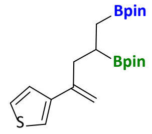

**10** $^{1}$H NMR ($CDCl_3$, 400 MHz)

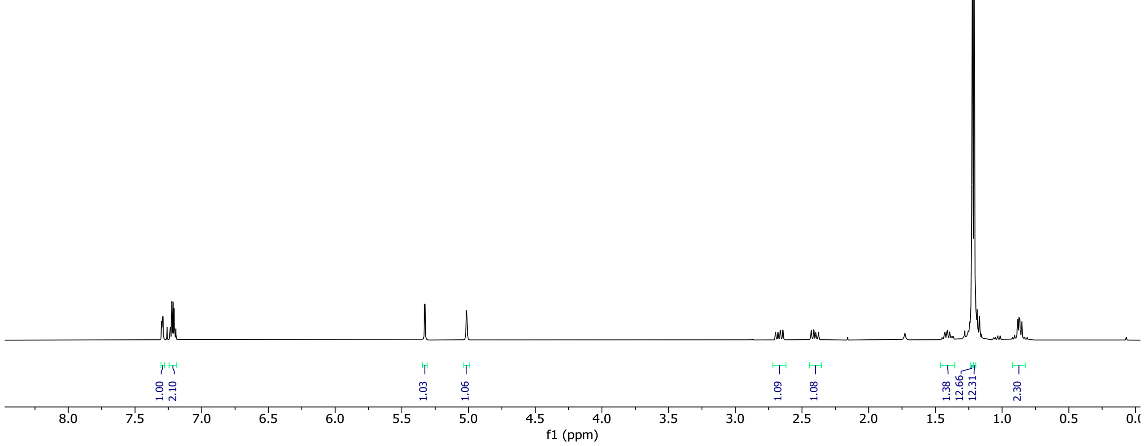

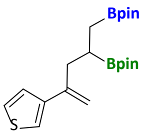


**10** $^{13}C$ NMR ($CDCl_3$, 100 MHz)

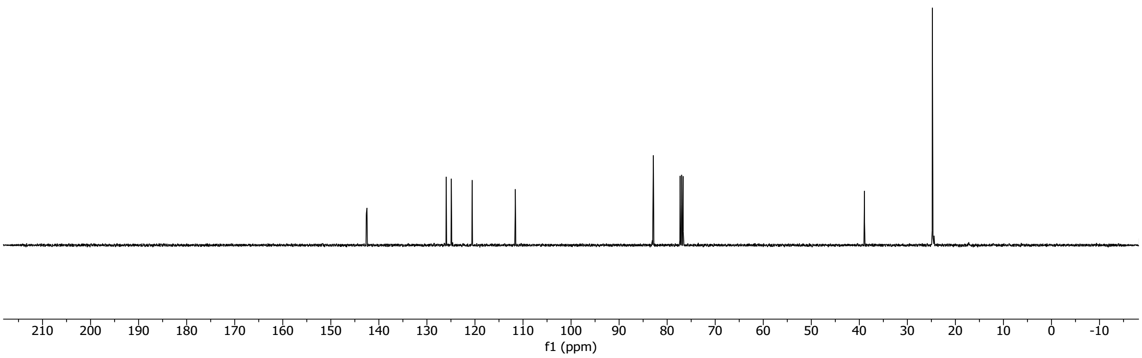


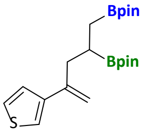


**10** $^{11}B$ NMR ($CDCl_3$, 128.3 MHz)

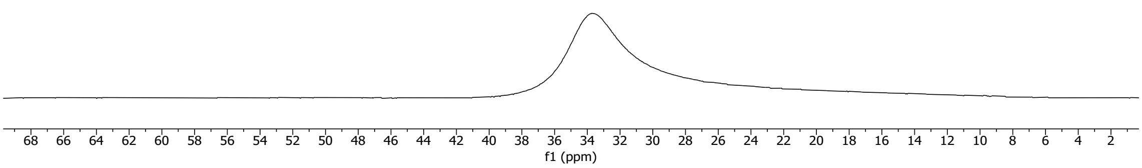

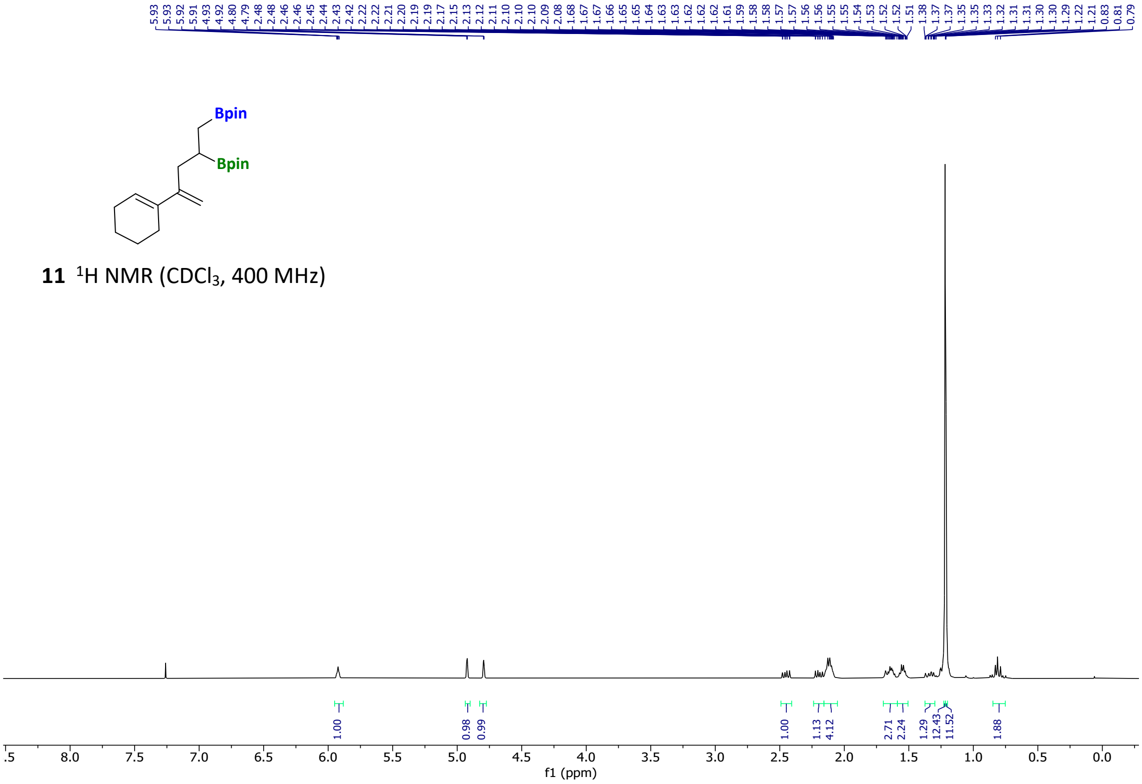


**11** $^{1}$H NMR ($CDCl_3$, 400 MHz)

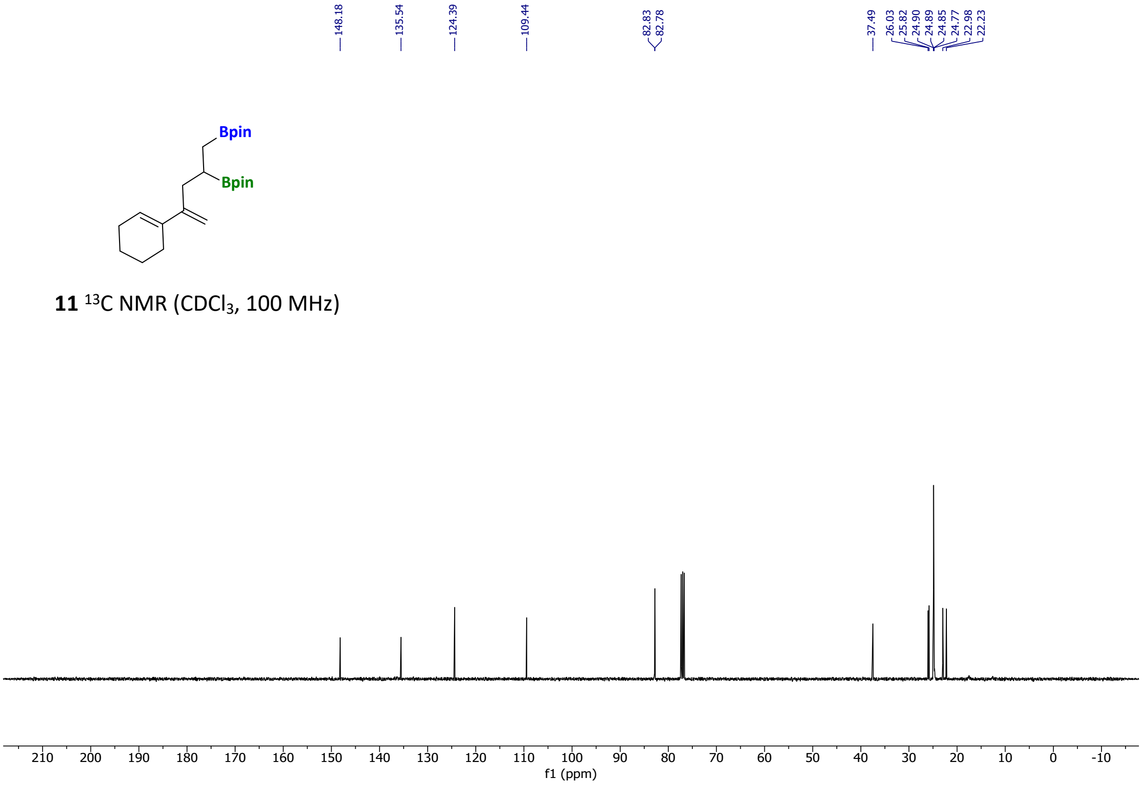


**11** $^{13}$C NMR ($CDCl_3$, 100 MHz)

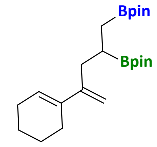


**11** $^{11}$B NMR (CDCl$_3$, 128.3 MHz)

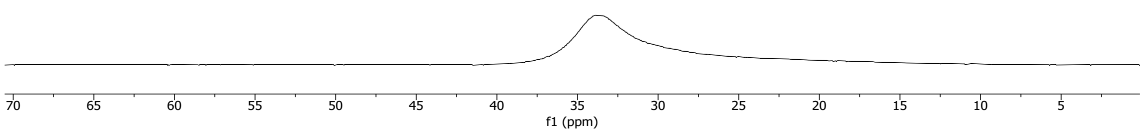


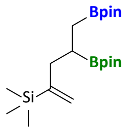


**12** $^{1}$H NMR (CDCl$_3$, 400 MHz)

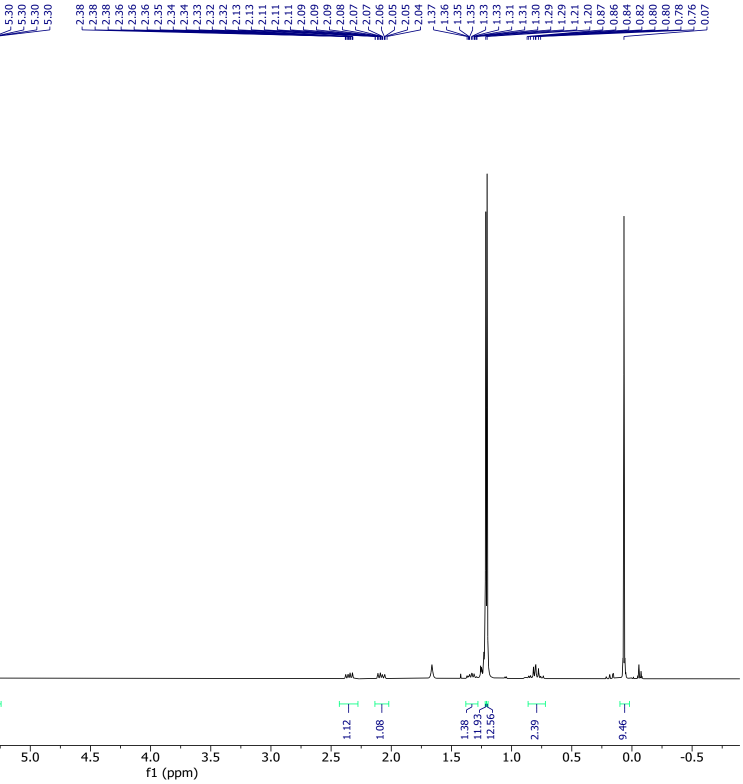

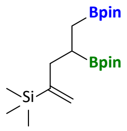


**12** $^{13}C$ NMR ($CDCl_3$, 100 MHz)

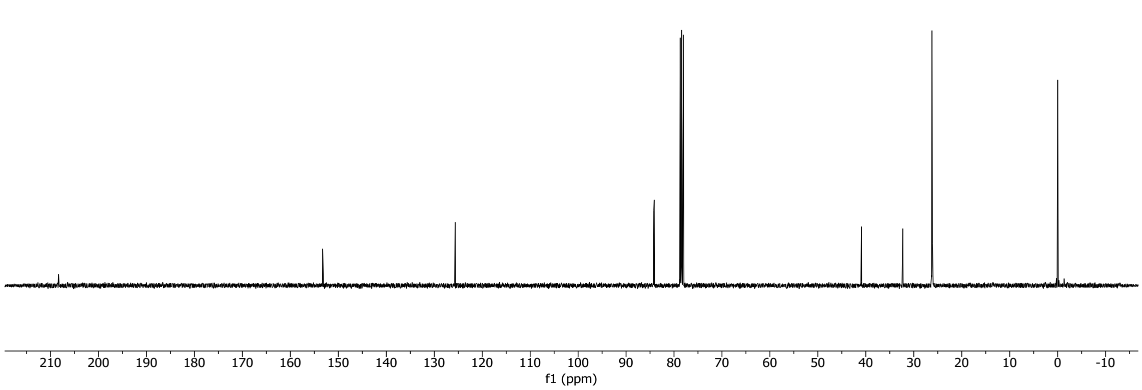


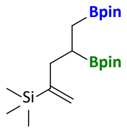


**12** $^{11}B$ NMR ($CDCl_3$, 128.3 MHz)

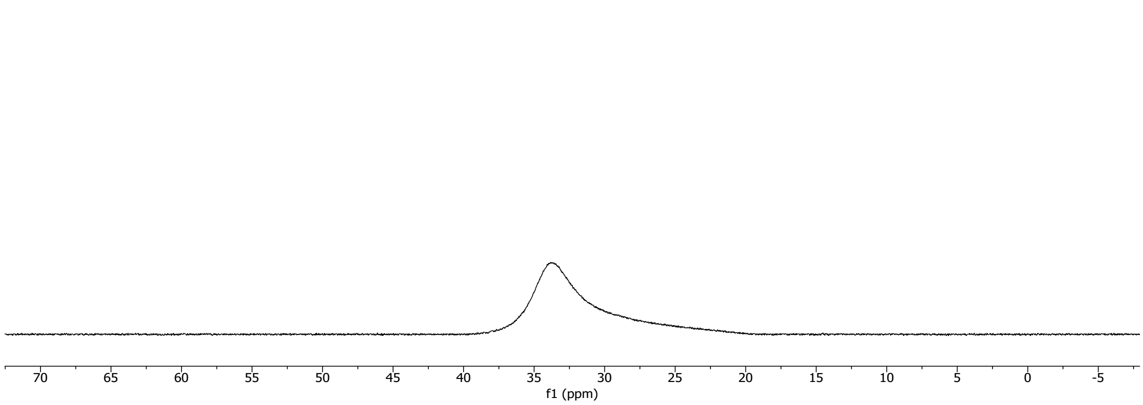

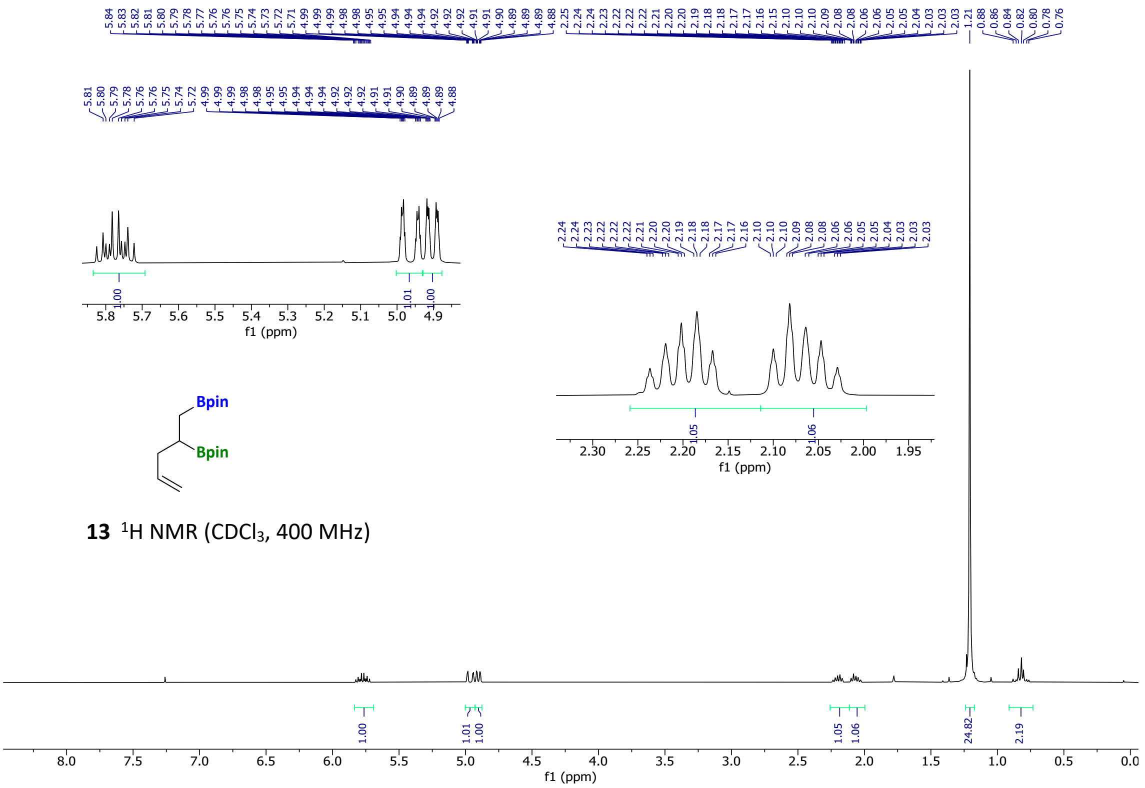


**13** ¹H NMR ($CDCl_3$, 400 MHz)

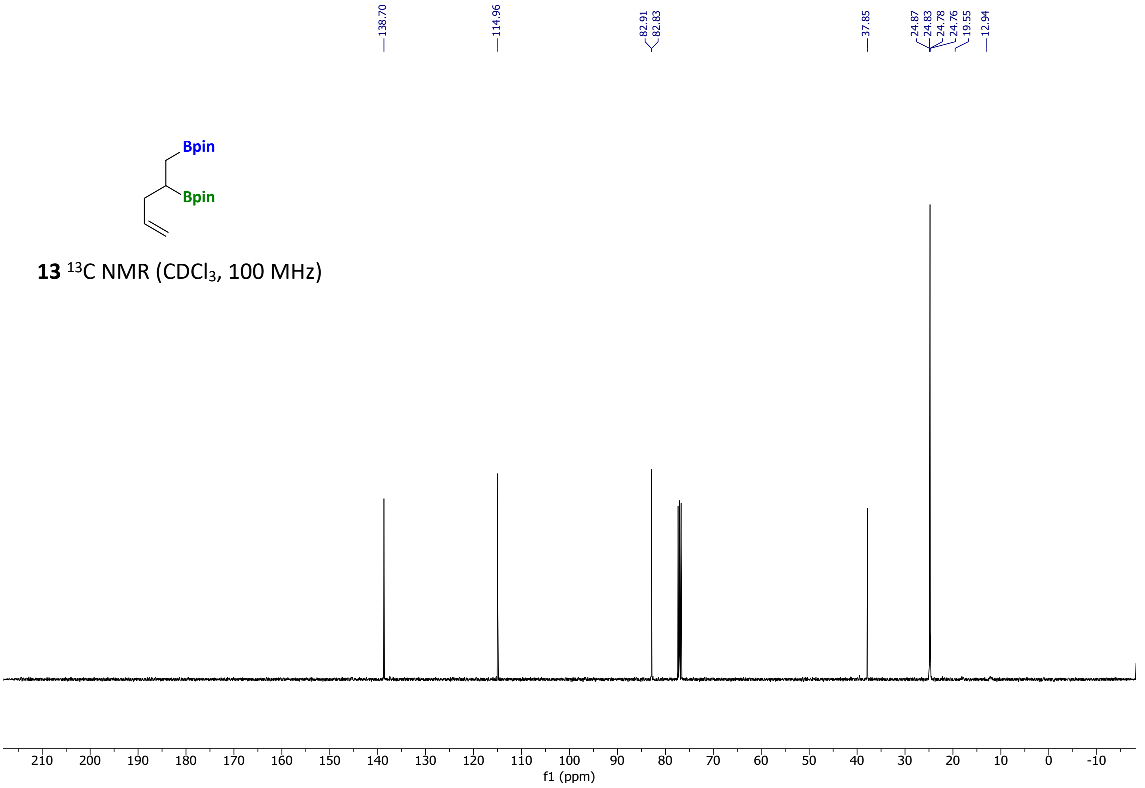


**13** ¹³C NMR ($CDCl_3$, 100 MHz)

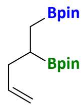


**13** $^{11}$B NMR ($CDCl_3$, 128.3 MHz)

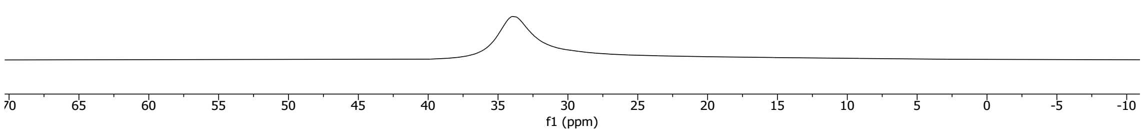

- $^{1}H$, $^{13}C$, $^{11}B$ Spectra for Cu-catalyzed borylcupration 1,4-B/Cu migration followed by electrophilic trapping

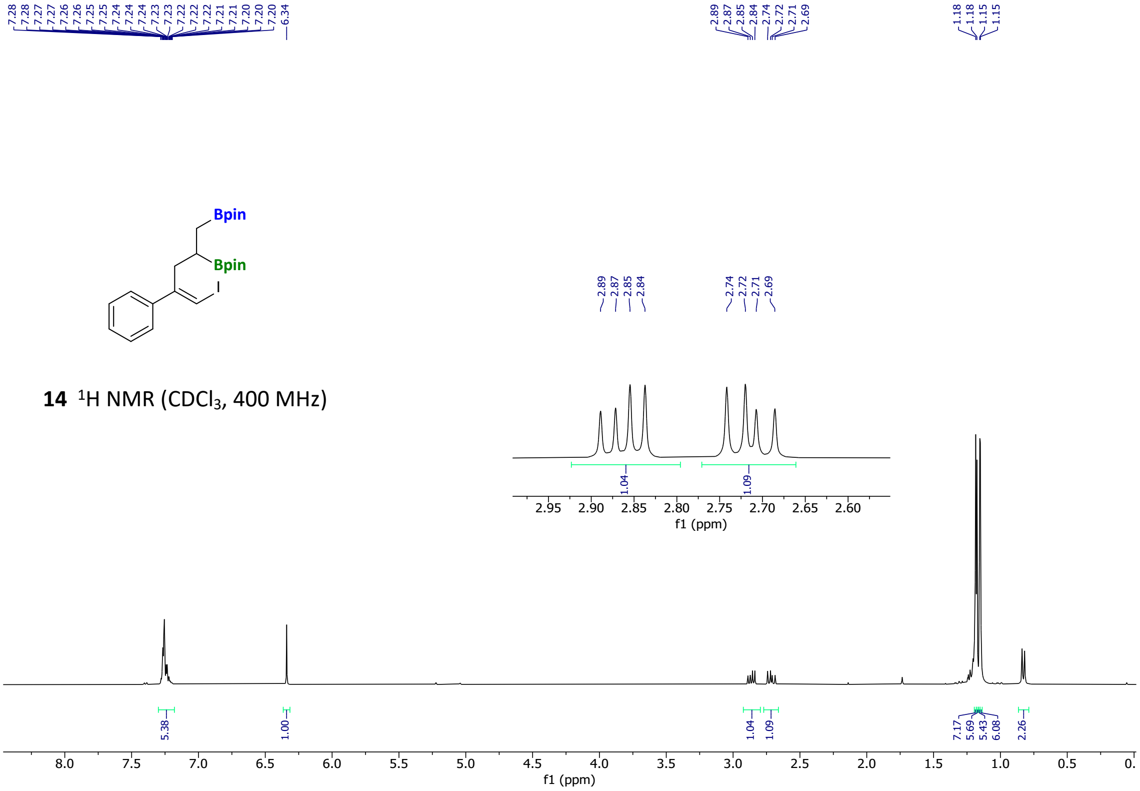

**14** $^{1}H$ NMR ($CDCl_3$, 400 MHz)

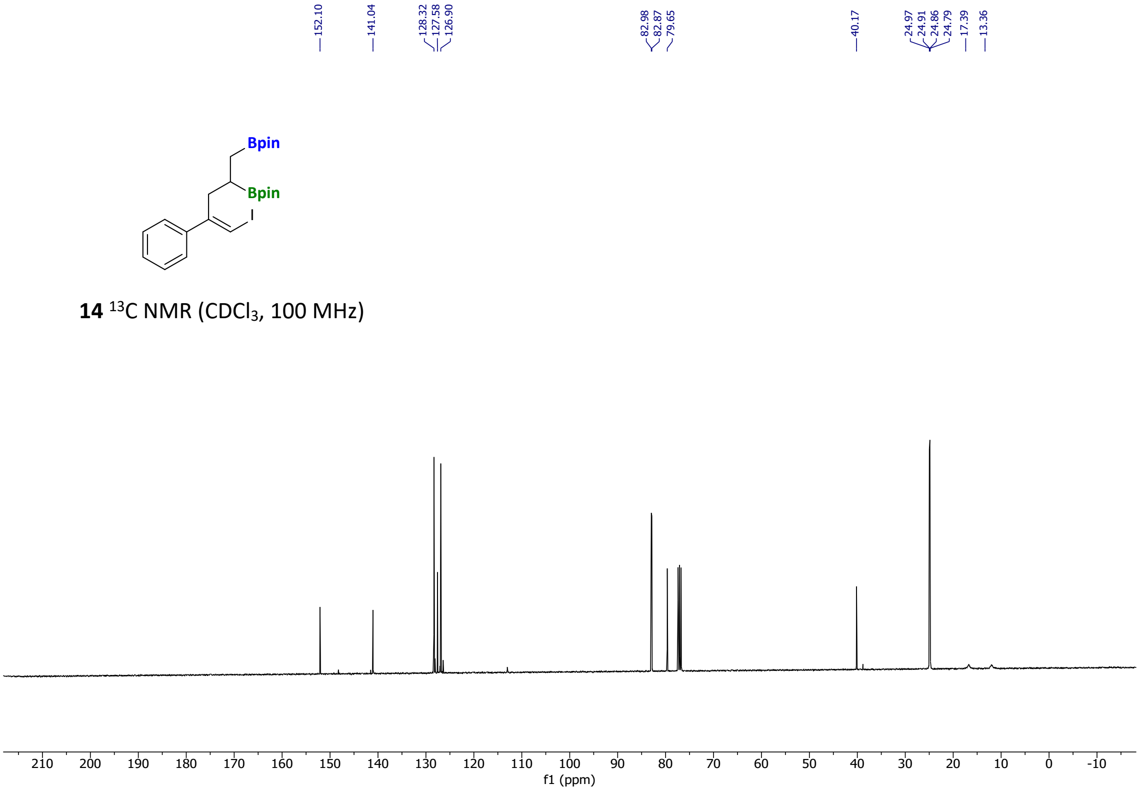

**14** $^{13}C$ NMR ($CDCl_3$, 100 MHz)

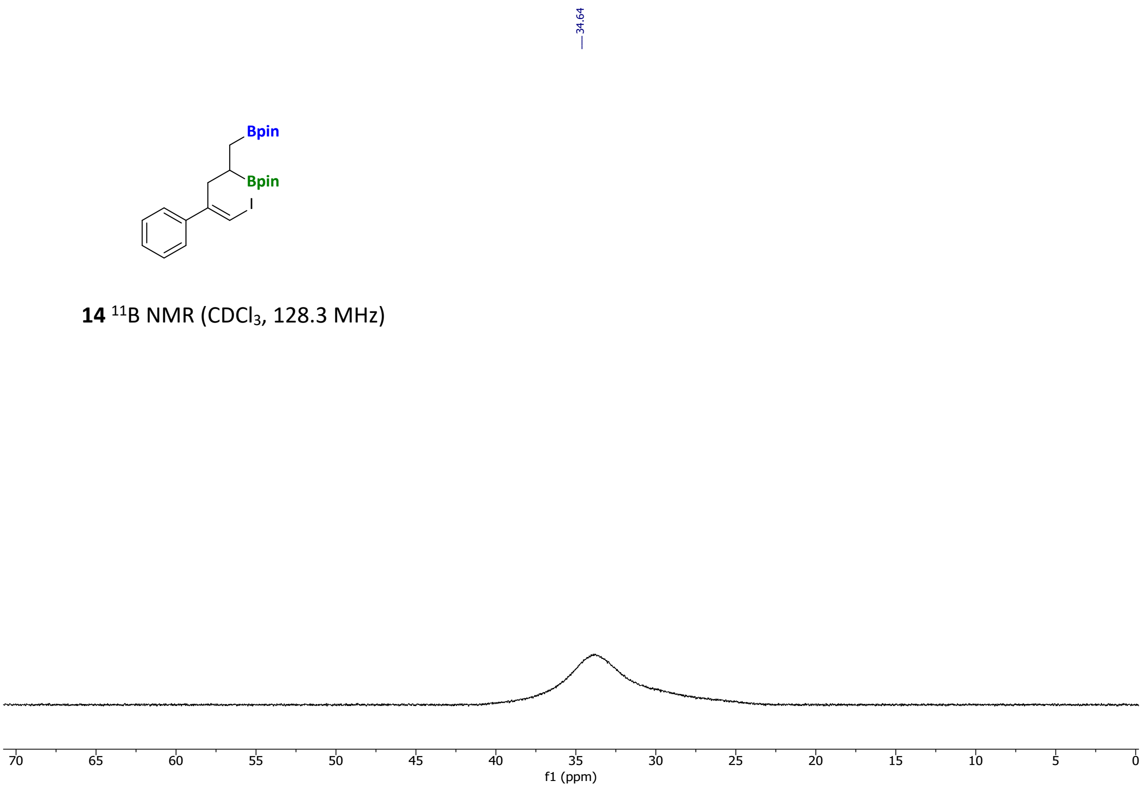


**14** $^{11}$B NMR ($CDCl_3$, 128.3 MHz)

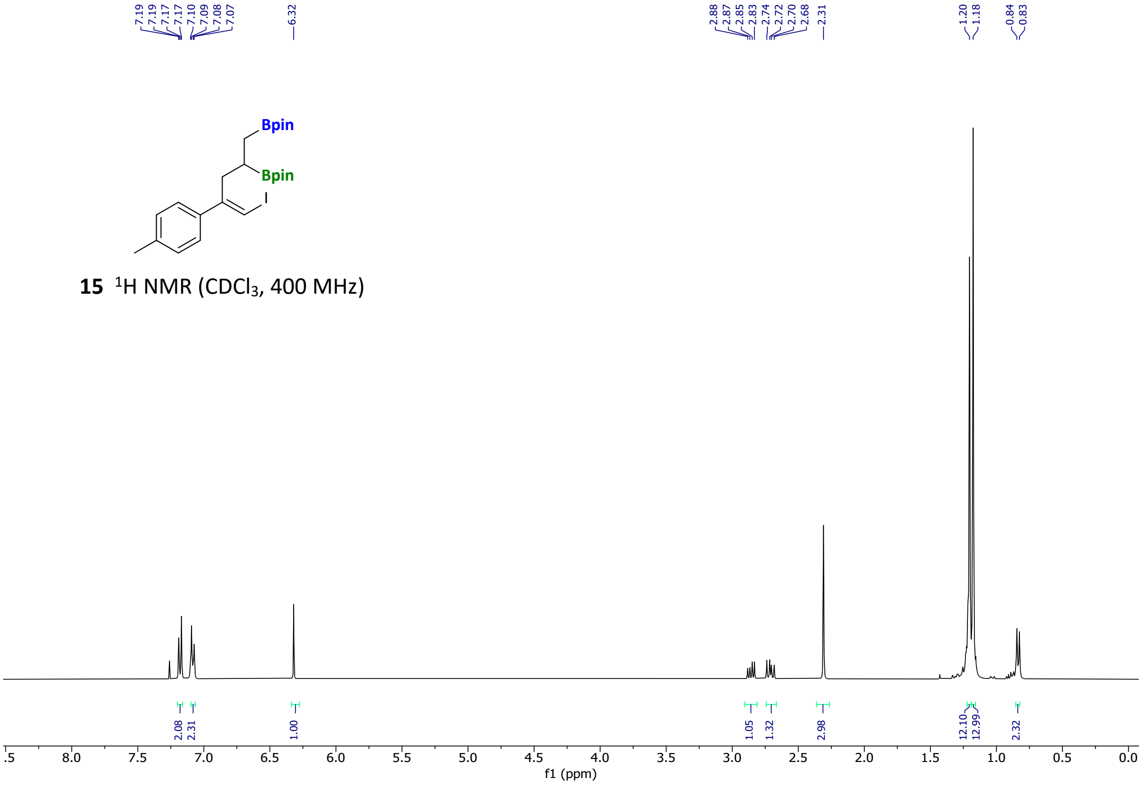


**15** $^{1}$H NMR ($CDCl_3$, 400 MHz)

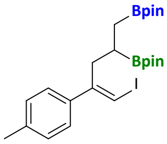


**15** $^{13}C$ NMR ($CDCl_3$, 100 MHz)

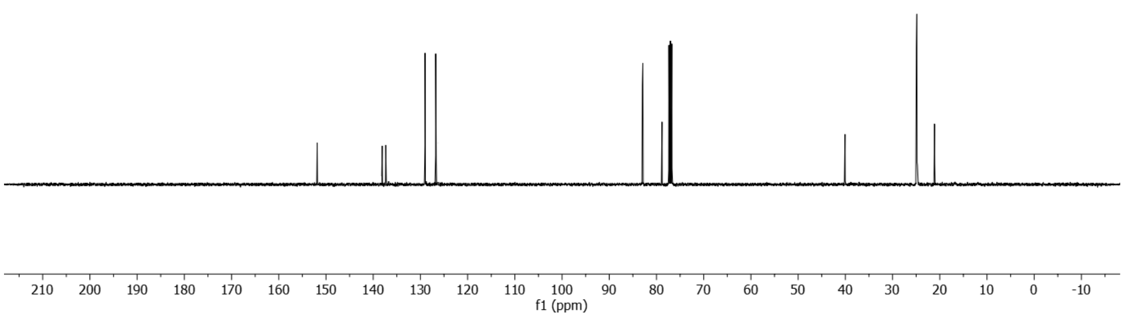


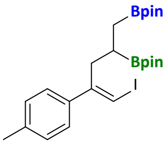


**15** $^{11}B$ NMR ($CDCl_3$, 128.3 MHz)

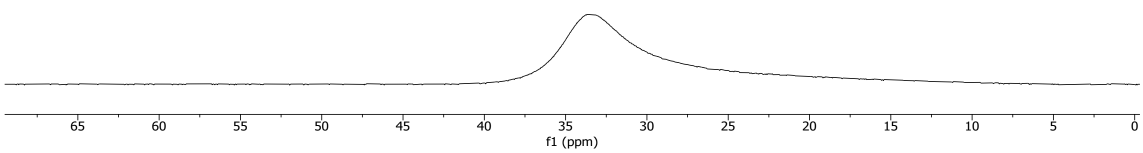

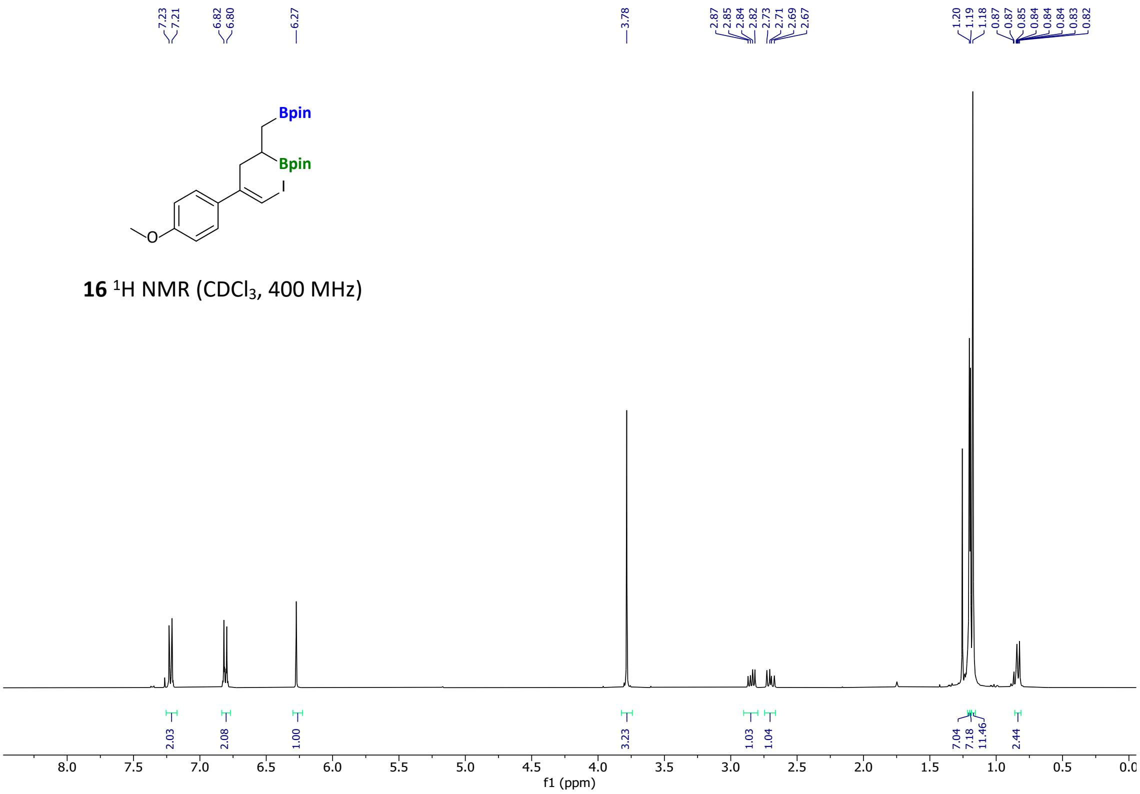


**16** $^{1}H$ NMR ($CDCl_3$, 400 MHz)

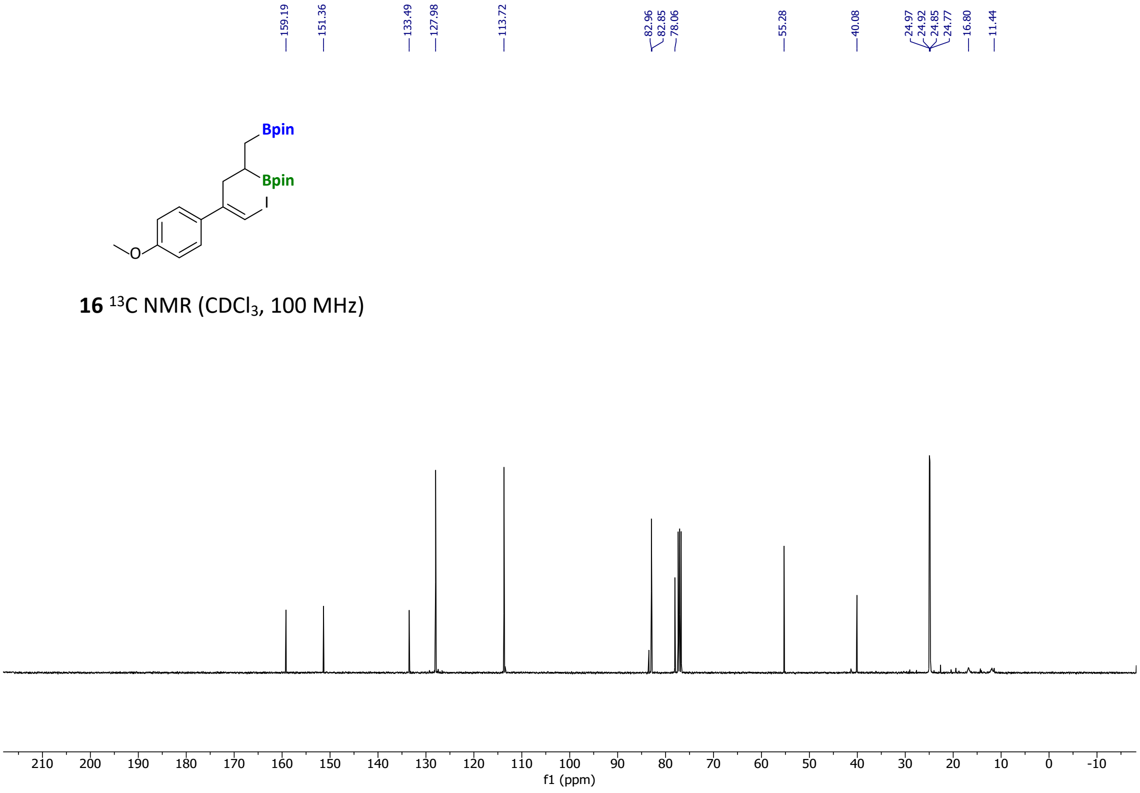


**16** $^{13}C$ NMR ($CDCl_3$, 100 MHz)

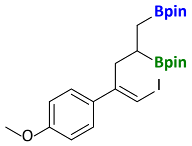

**16** $^{11}$B NMR ($CDCl_3$, 128.3 MHz)

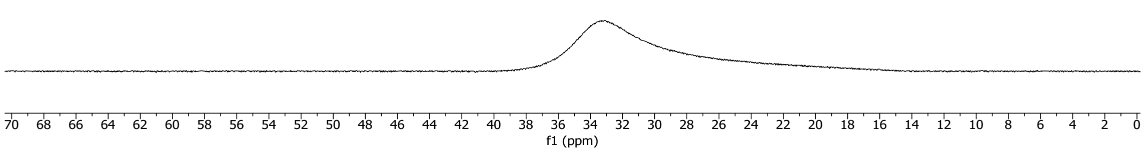


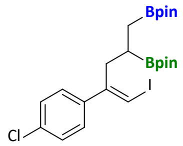

**17** $^{1}$H NMR ($CDCl_3$, 400 MHz)

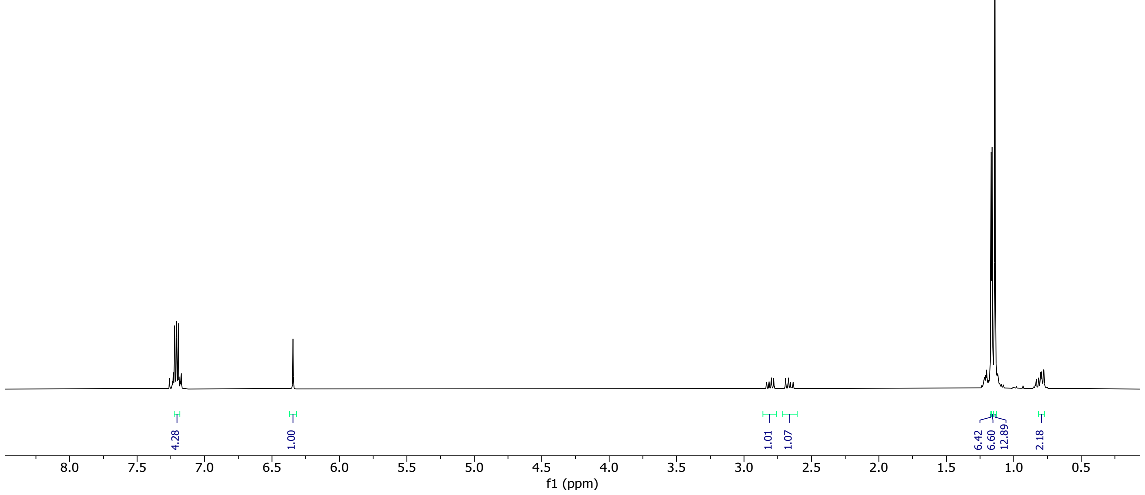

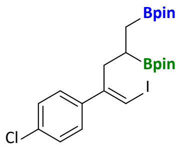


**17** $^{13}$C NMR ($CDCl_3$, 100 MHz)

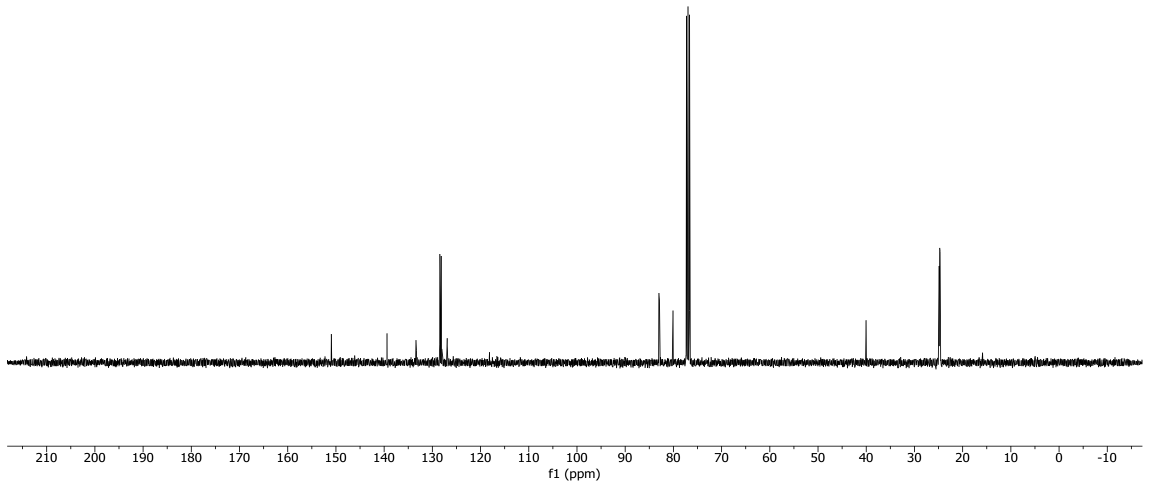


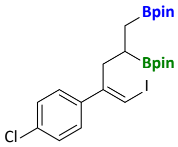


**17** $^{11}$B NMR ($CDCl_3$, 128.3 MHz)

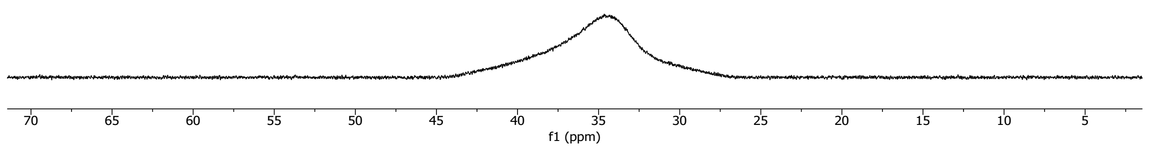

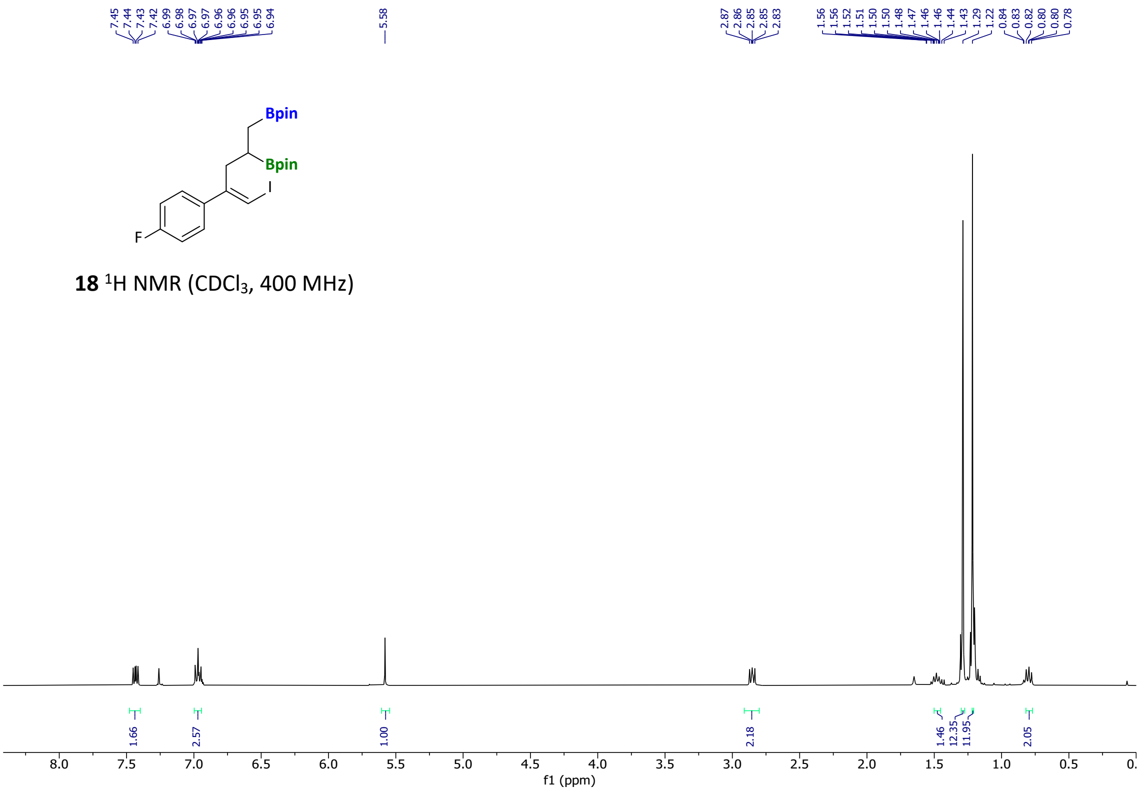


**18** $^{1}H$ NMR ($CDCl_3$, 400 MHz)

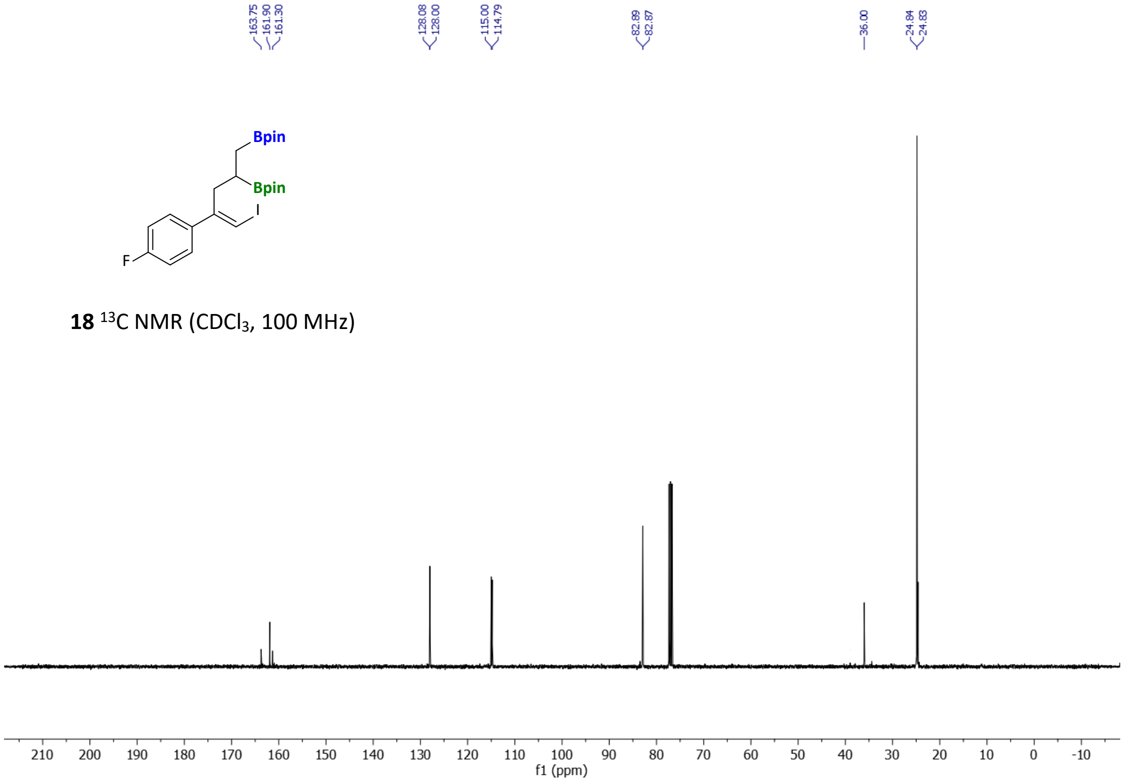


**18** $^{13}C$ NMR ($CDCl_3$, 100 MHz)

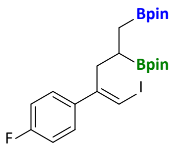


**18** $^{11}B$ NMR ($CDCl_3$, 128.3 MHz)

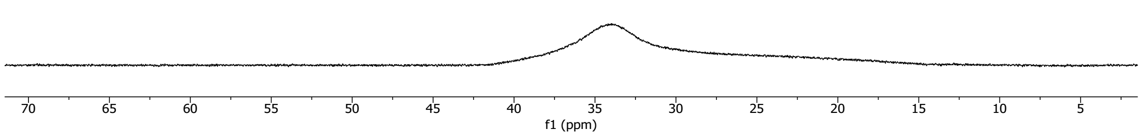


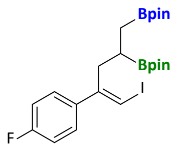


**18** $^{19}F$ NMR ($CDCl_3$, 377 MHz)

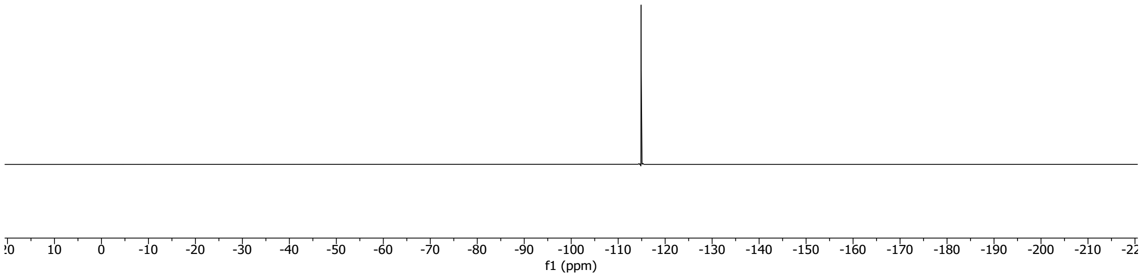

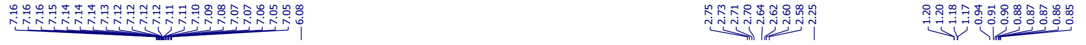

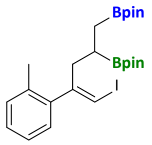

**19** $^{1}$H NMR ($CDCl_3$, 400 MHz)

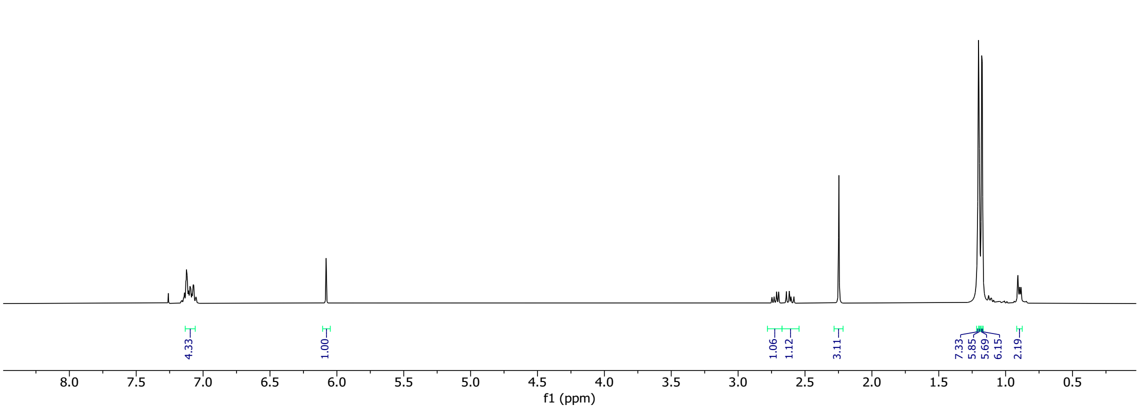


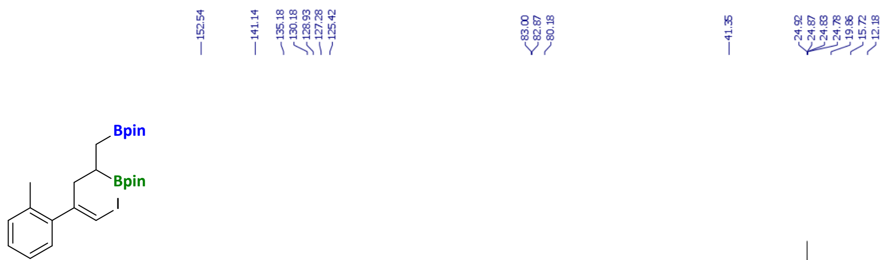

**19** $^{13}$C NMR ($CDCl_3$, 100 MHz)

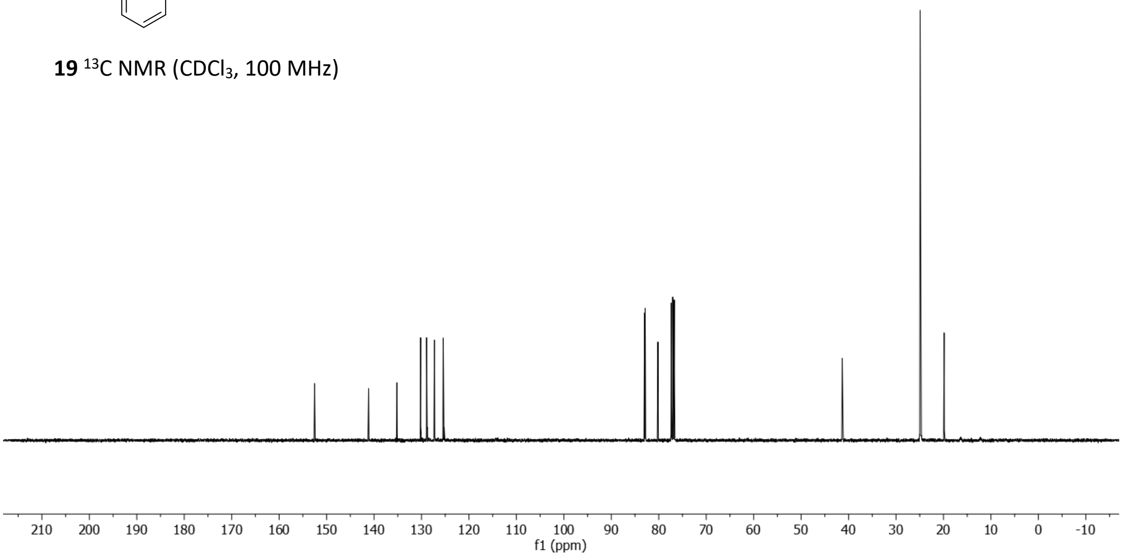

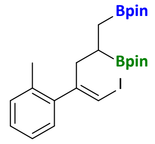


**19** $^{11}$B NMR ($CDCl_3$, 128.3 MHz)

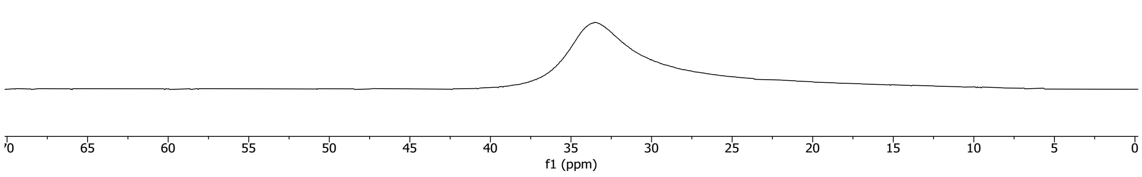


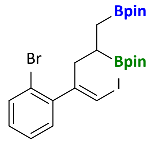


**20** $^{1}$H NMR ($CDCl_3$, 400 MHz)

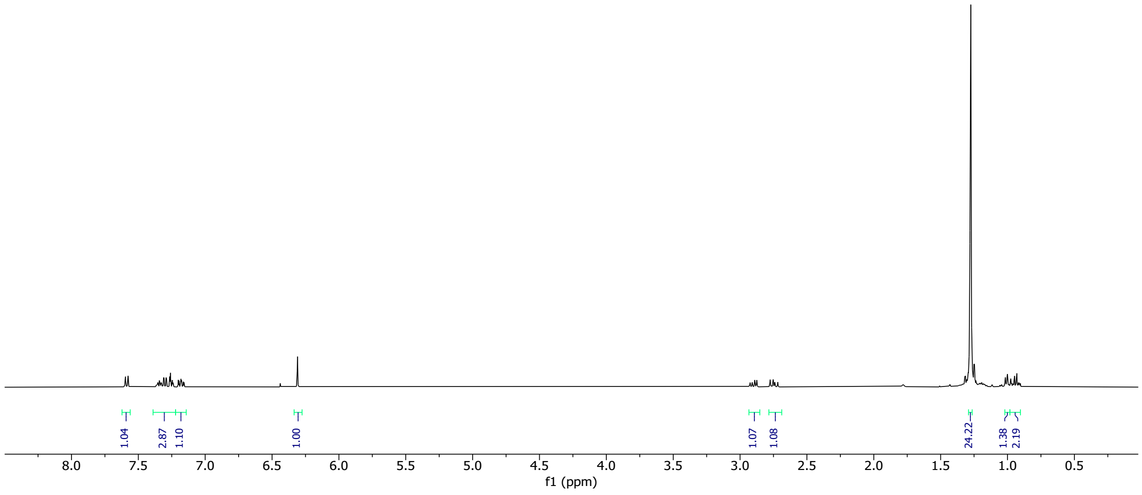

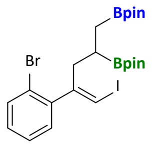


**20** $^{13}$C NMR ($CDCl_3$, 100 MHz)

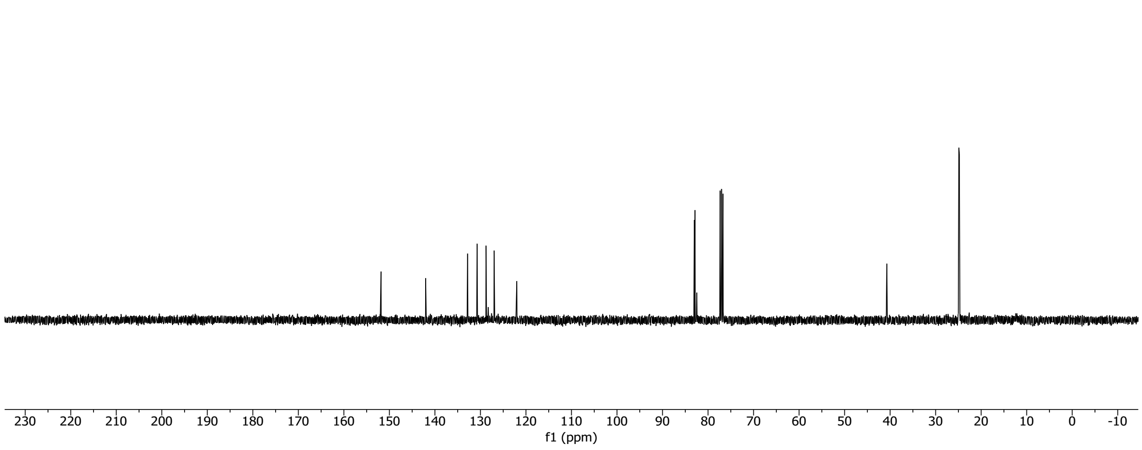


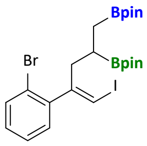


**20** $^{11}$B NMR ($CDCl_3$, 128.3 MHz)

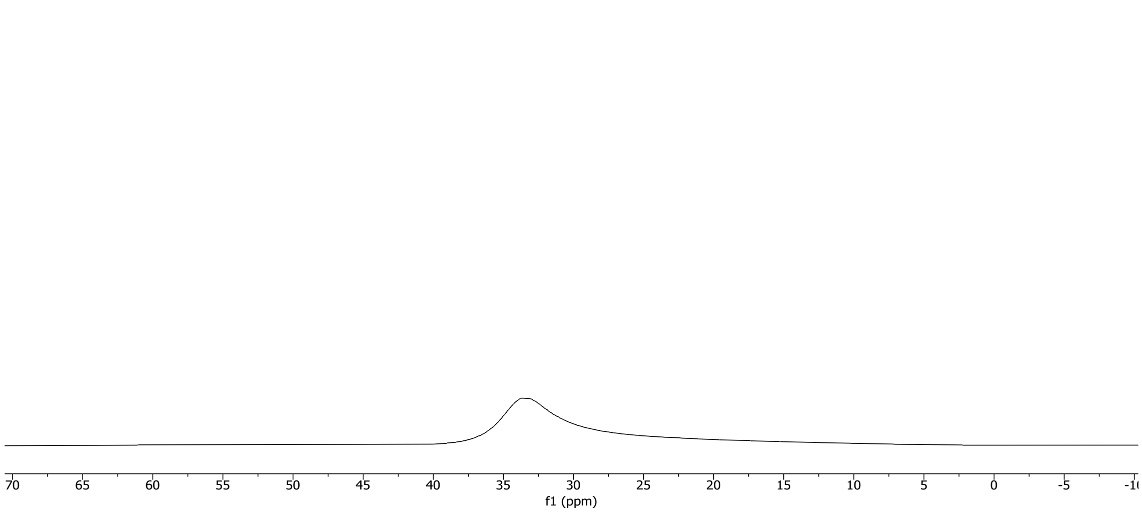

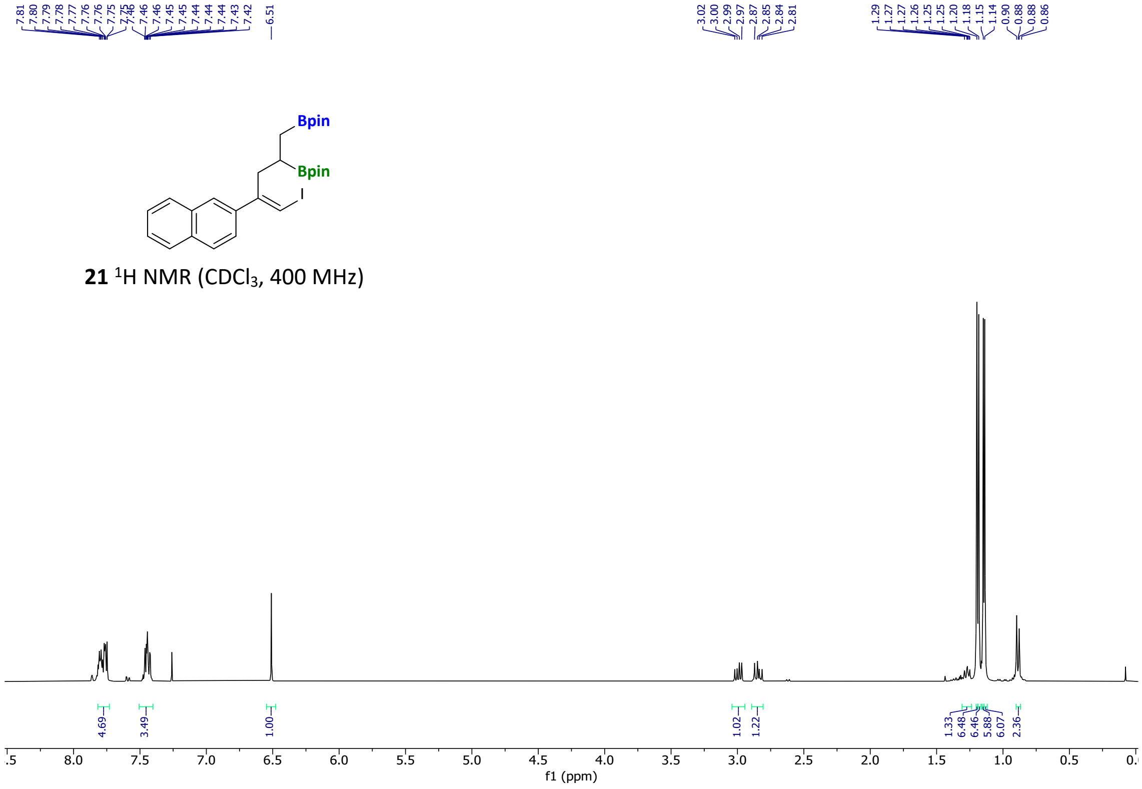


**21** $^{1}H$ NMR ($CDCl_3$, 400 MHz)

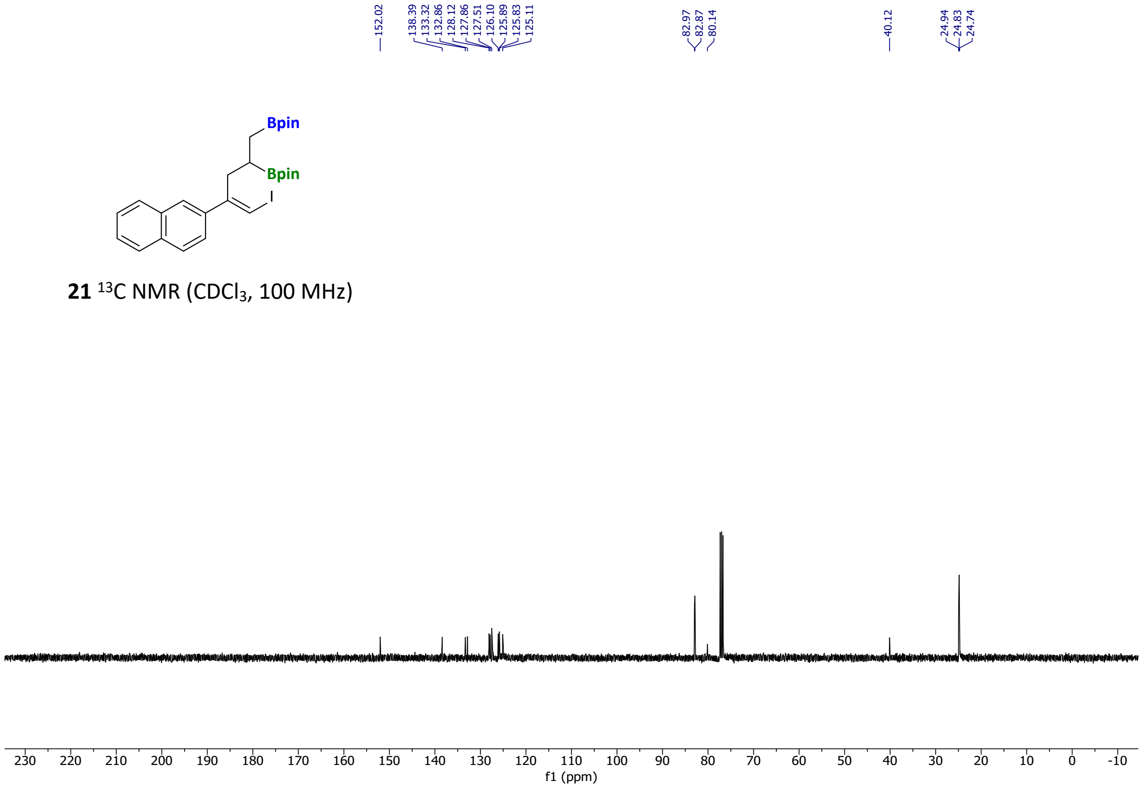


**21** $^{13}C$ NMR ($CDCl_3$, 100 MHz)

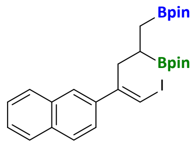


**21** $^{11}$B NMR ($CDCl_3$, 128.3 MHz)

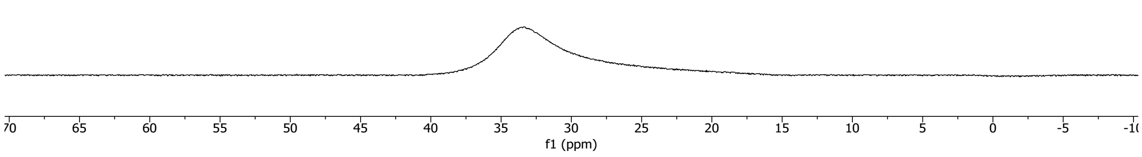


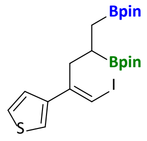


**22** $^{1}$H NMR ($CDCl_3$, 400 MHz)

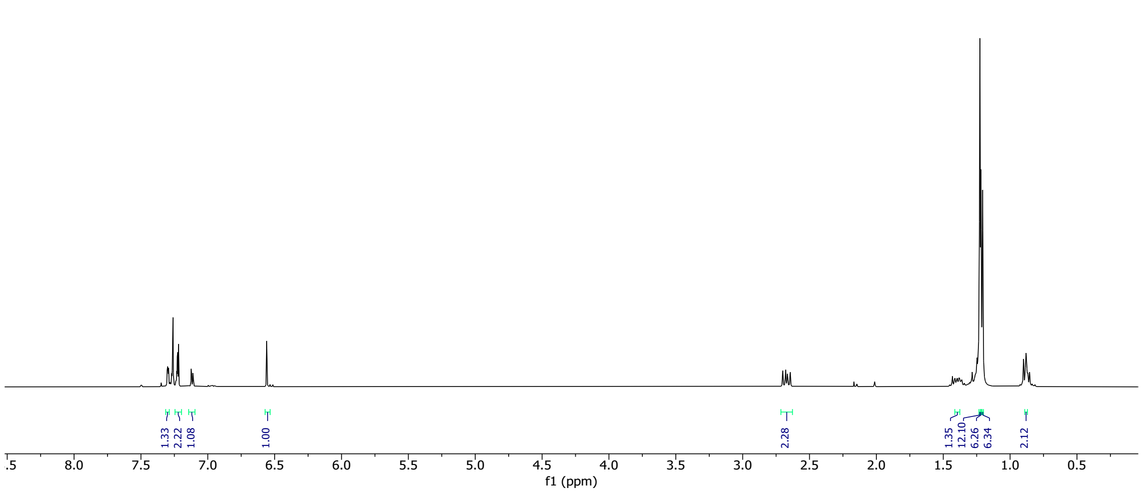

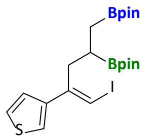


**22** $^{13}$C NMR (CDCl$_3$, 100 MHz)

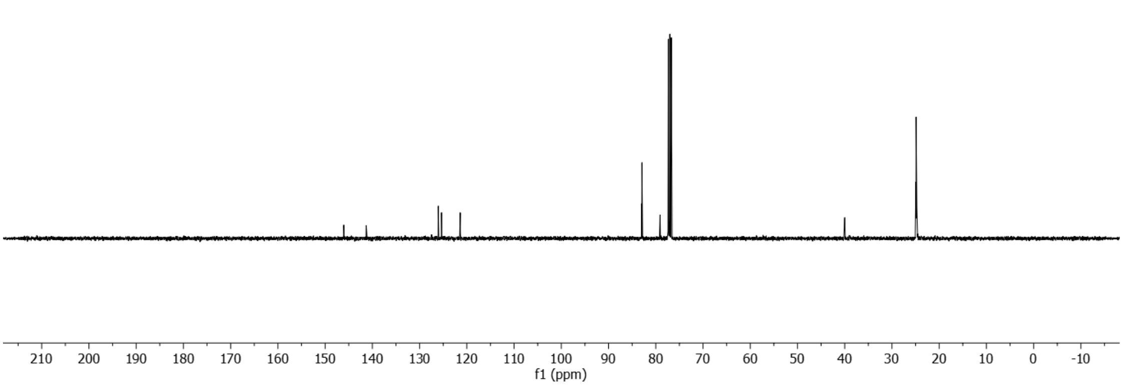


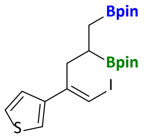


**22** $^{11}$B NMR (CDCl$_3$, 128.3 MHz)

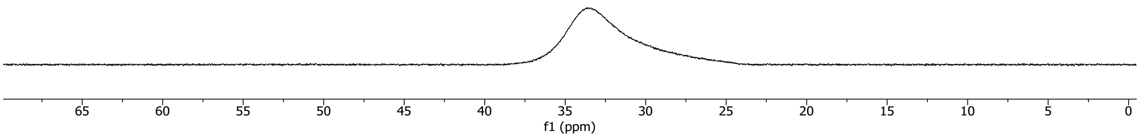

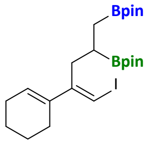


**23** $^{1}$H NMR ($CDCl_3$, 400 MHz)

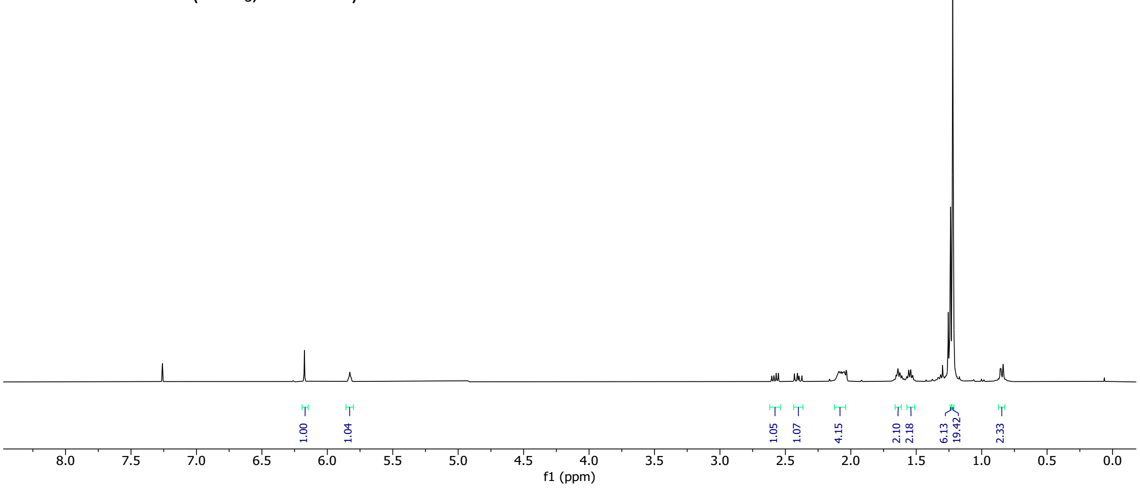


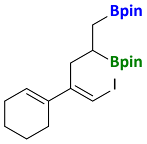


**23** $^{13}$C NMR ($CDCl_3$, 100 MHz)

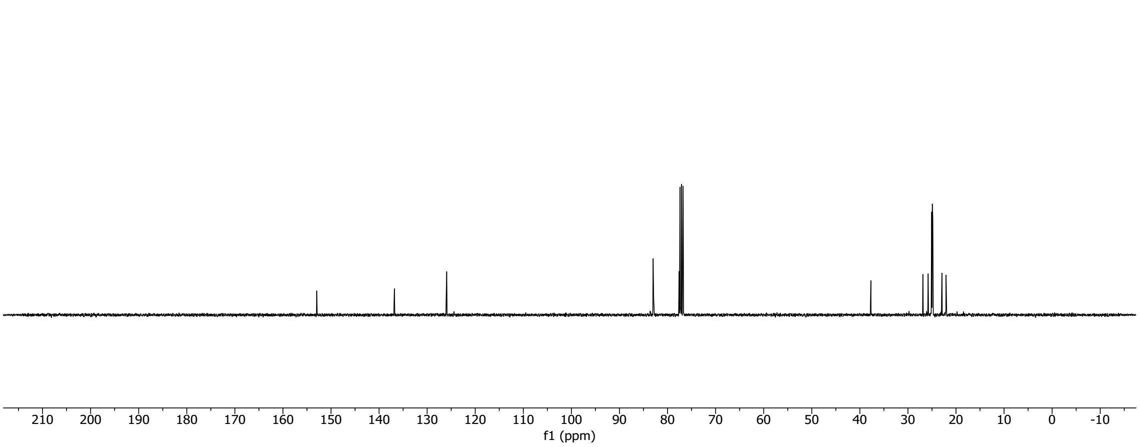

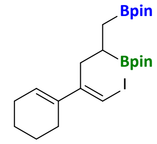


**23** $^{11}$B NMR ($CDCl_3$, 128.3 MHz)

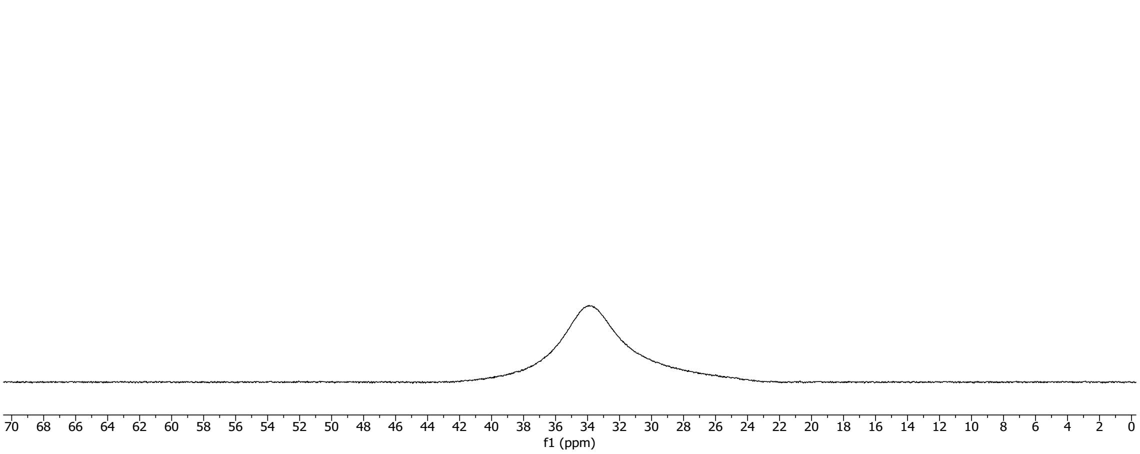


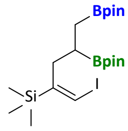


**24** $^{1}$H NMR ($CDCl_3$, 400 MHz)

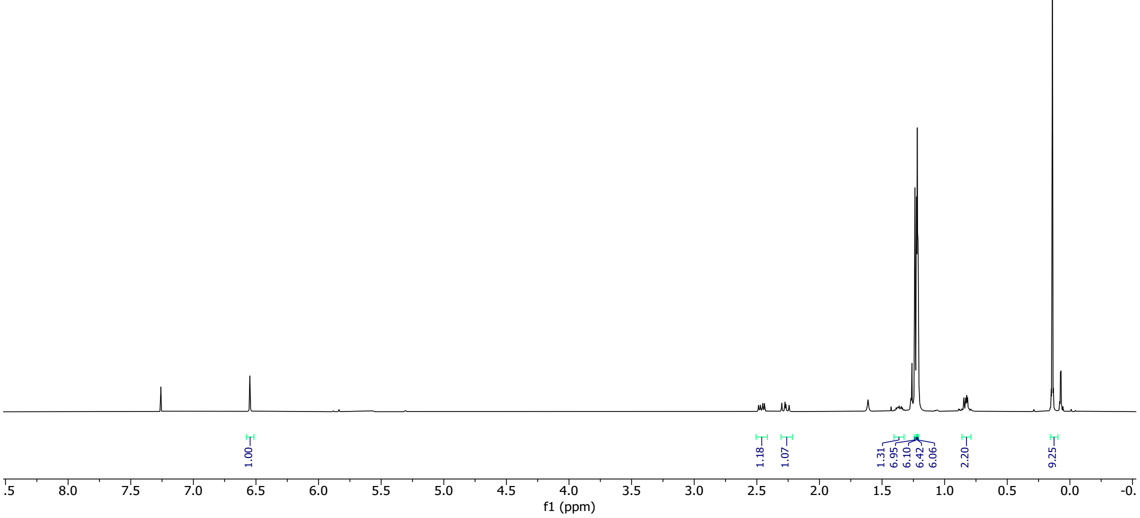

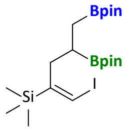

**24** $^{13}$C NMR ($CDCl_3$, 100 MHz)

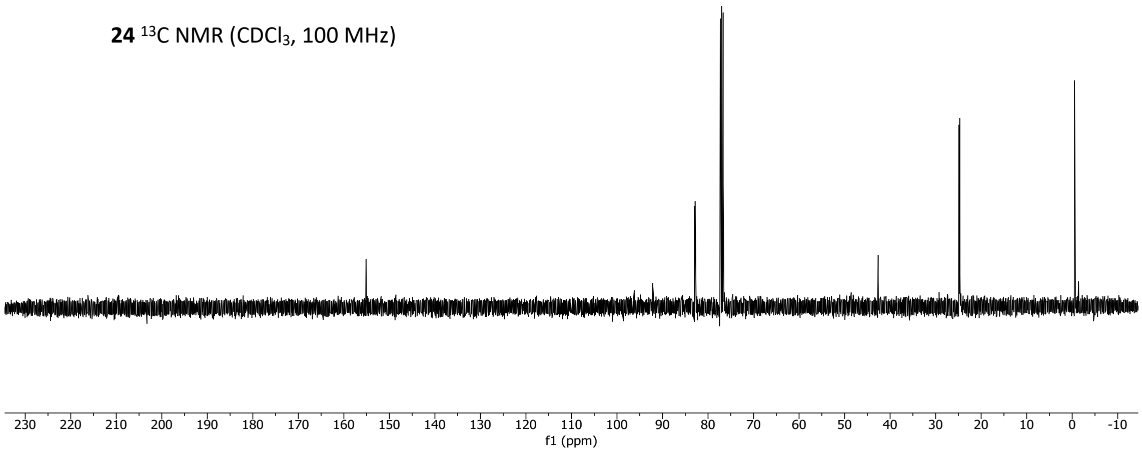


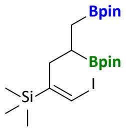

**24** $^{11}$B NMR ($CDCl_3$, 128.3 MHz)

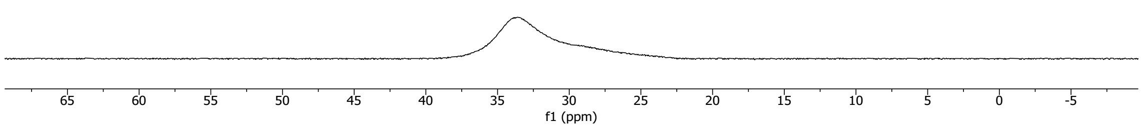

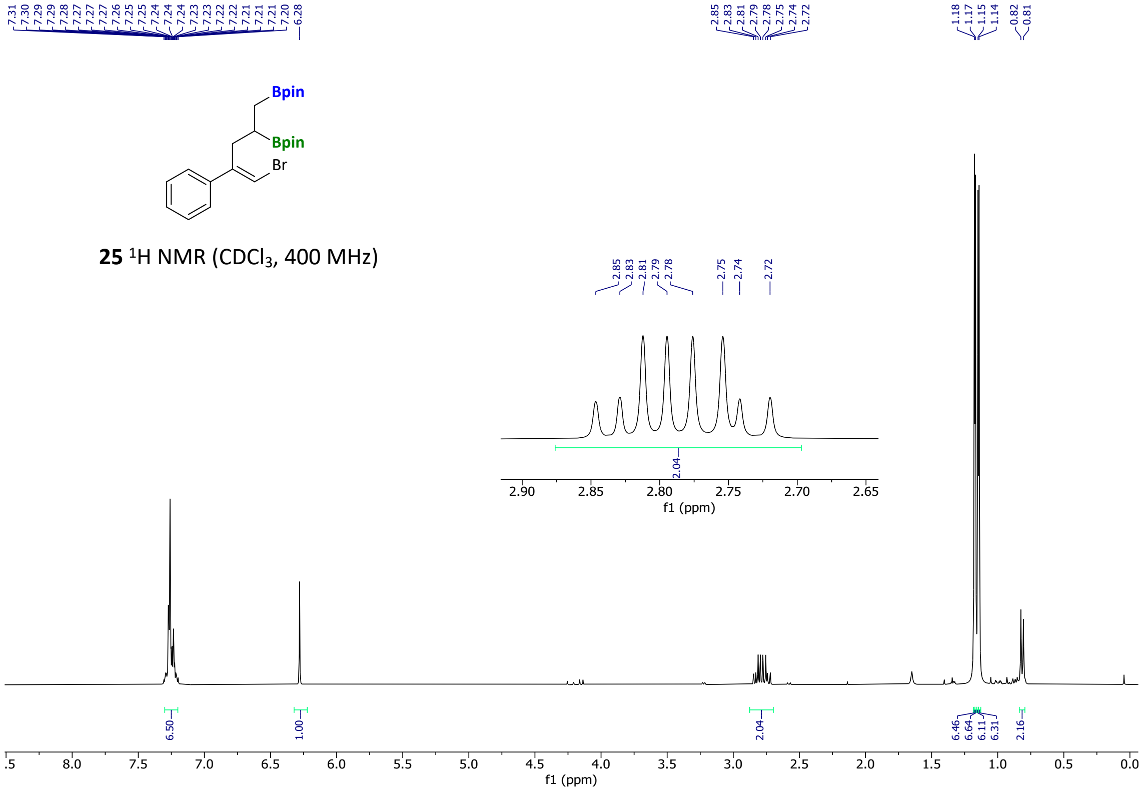


**25** $^{1}$H NMR ($CDCl_3$, 400 MHz)

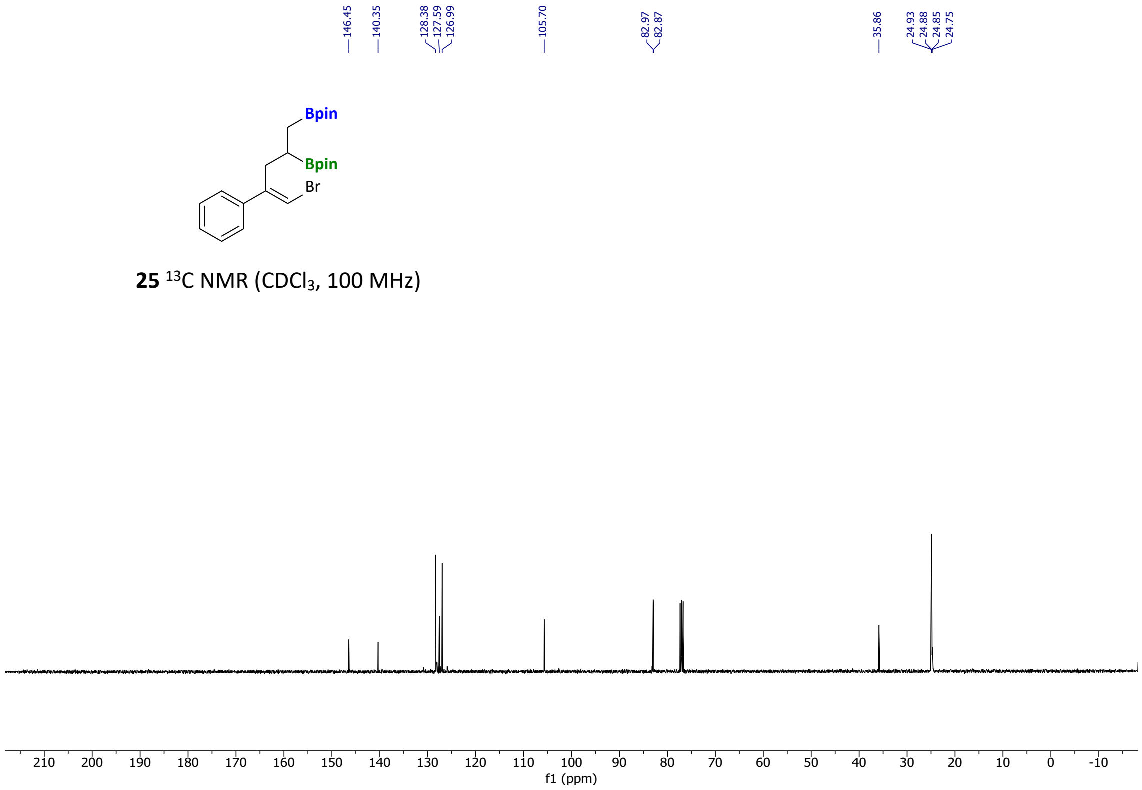


**25** $^{13}$C NMR ($CDCl_3$, 100 MHz)

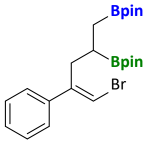


**25** $^{11}$B NMR ($CDCl_3$, 128.3 MHz)

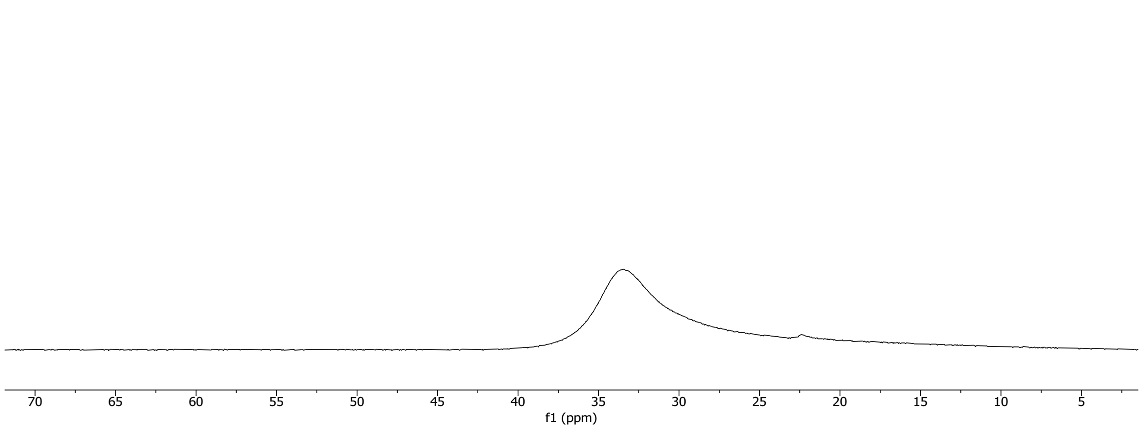




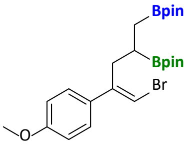


**26** $^{1}$H NMR ($CDCl_3$, 400 MHz)

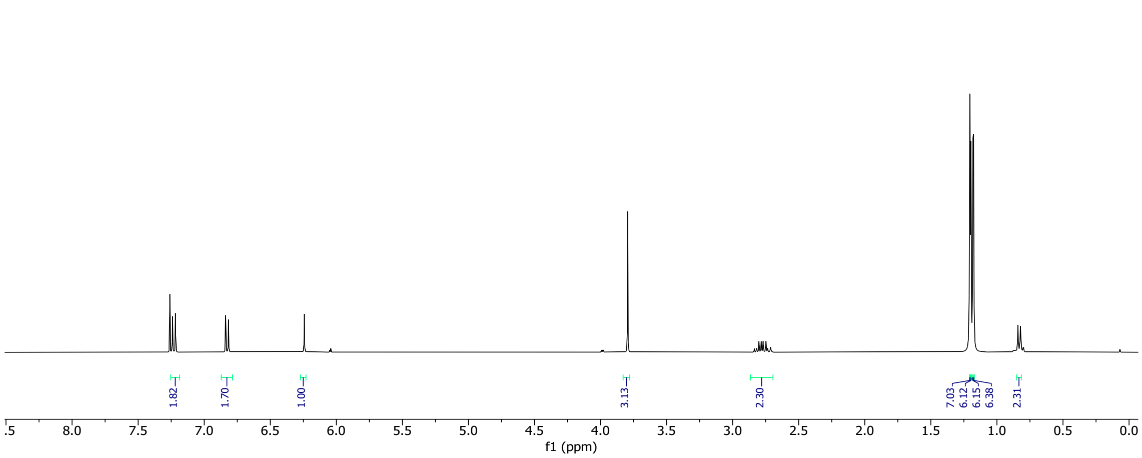

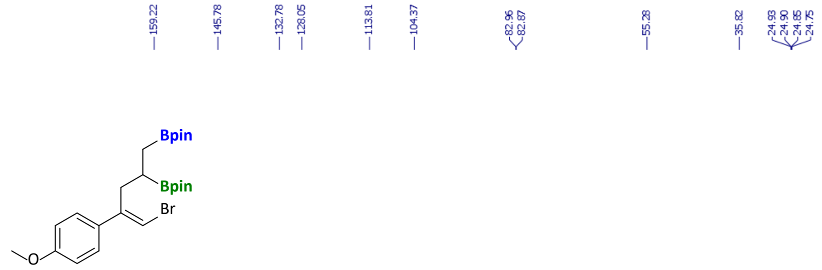


**26** $^{13}$C NMR ($CDCl_3$, 100 MHz)

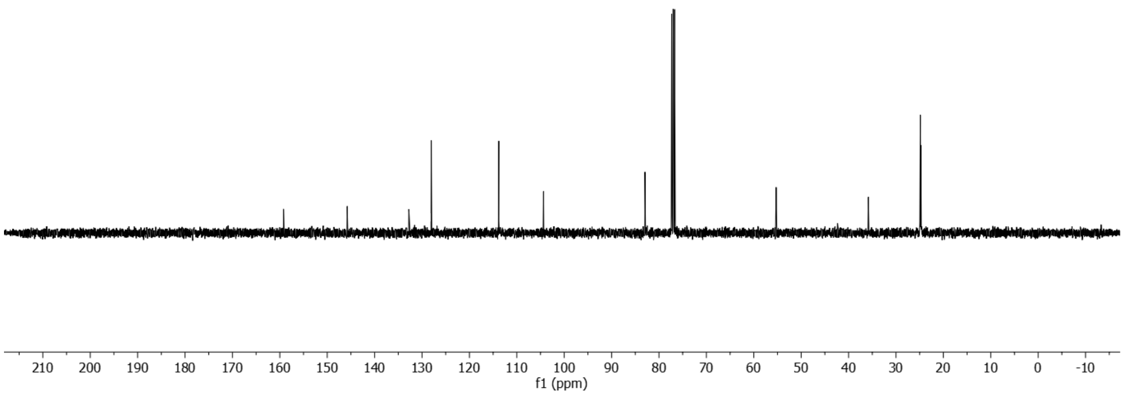


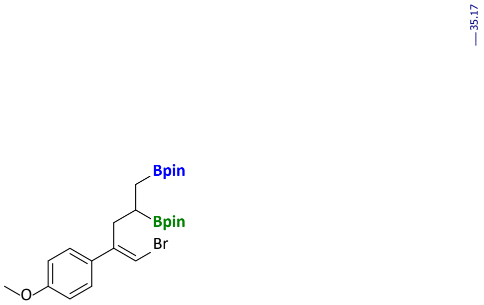


**26** $^{11}$B NMR ($CDCl_3$, 128.3 MHz)

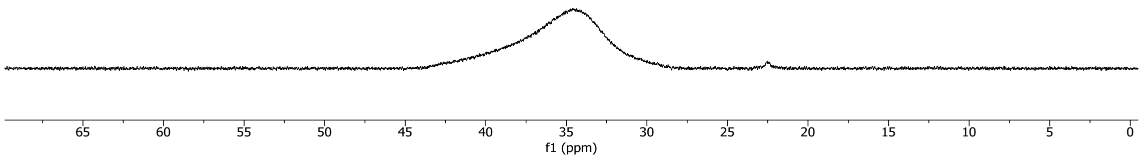

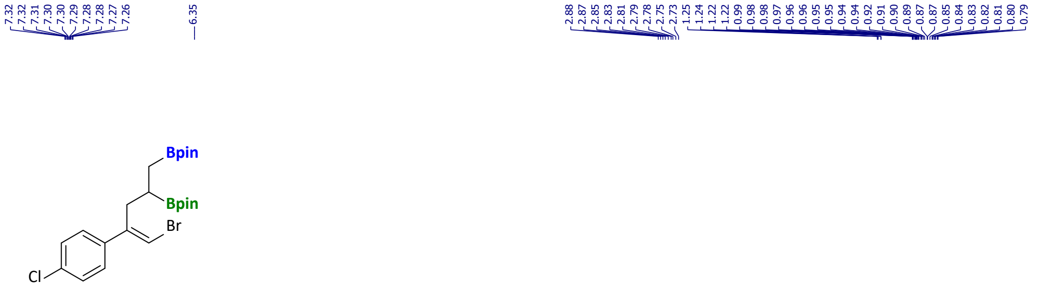


**27** $^{1}H$ NMR ($CDCl_3$, 400 MHz)

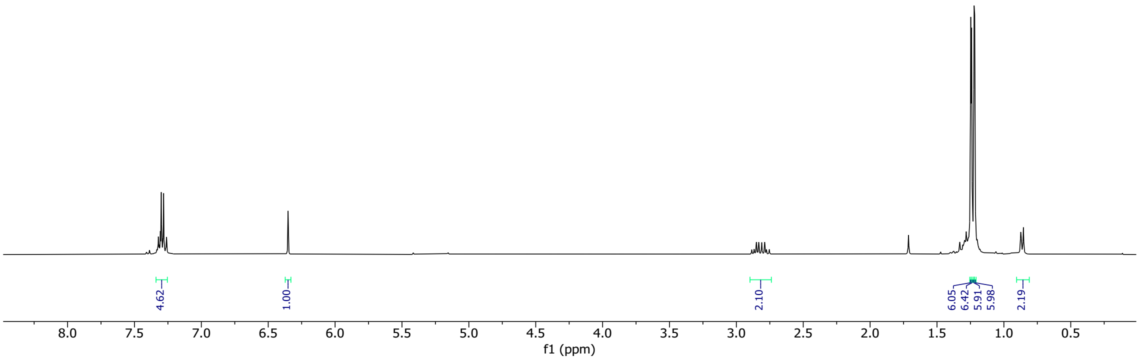


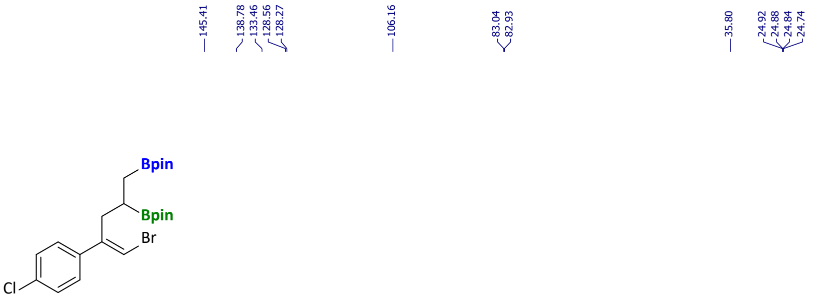


**27** $^{13}C$ NMR ($CDCl_3$, 100 MHz)

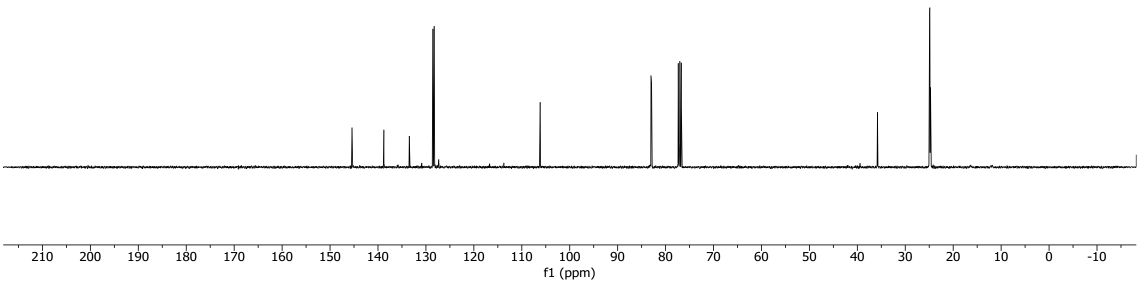

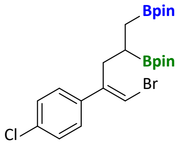


**27** $^{11}$B NMR ($CDCl_3$, 128.3 MHz)

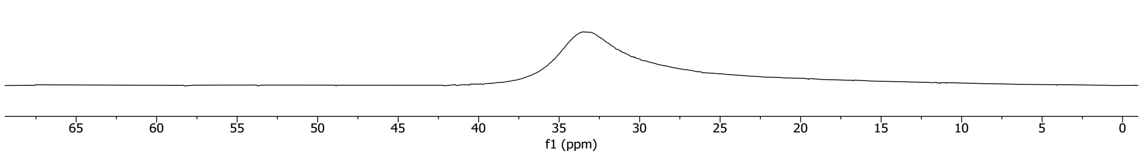


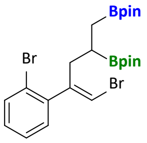


**28** $^{1}$H NMR ($CDCl_3$, 400 MHz)

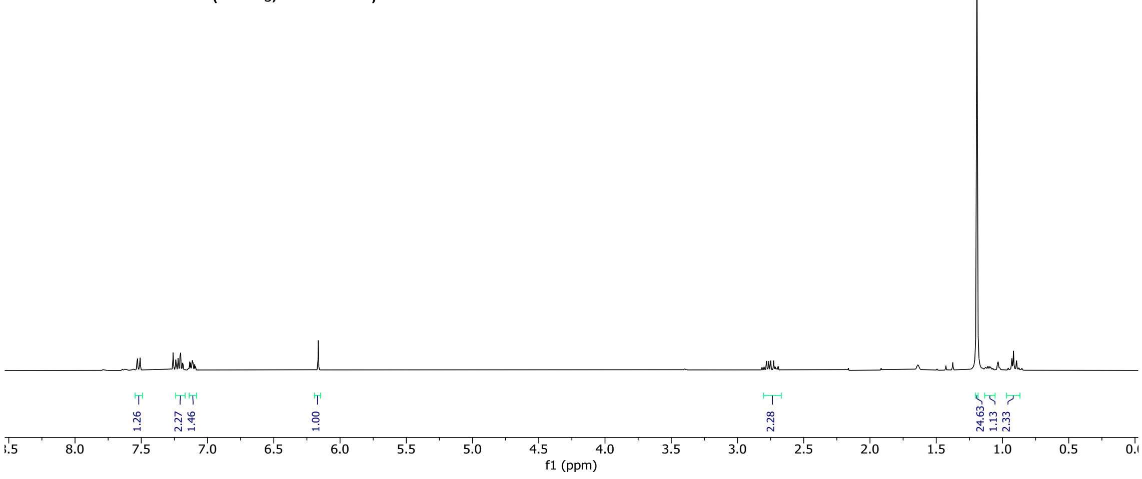

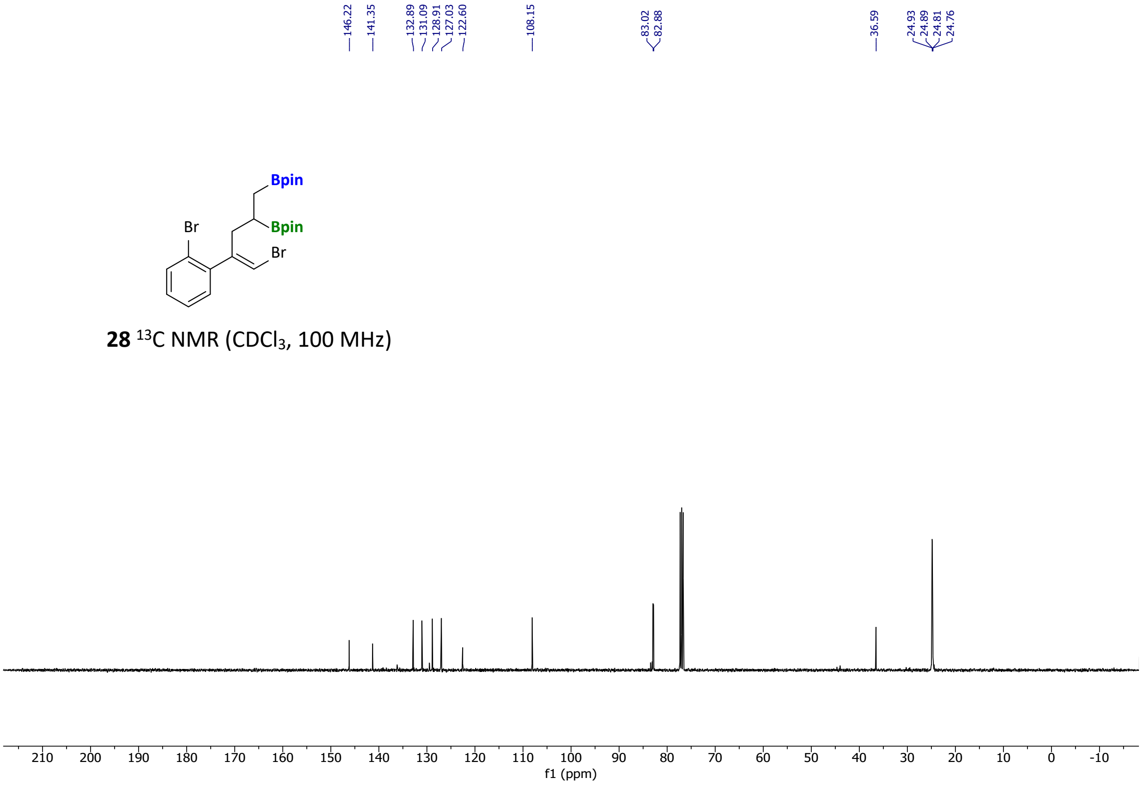


**28** $^{13}C$ NMR ($CDCl_3$, 100 MHz)

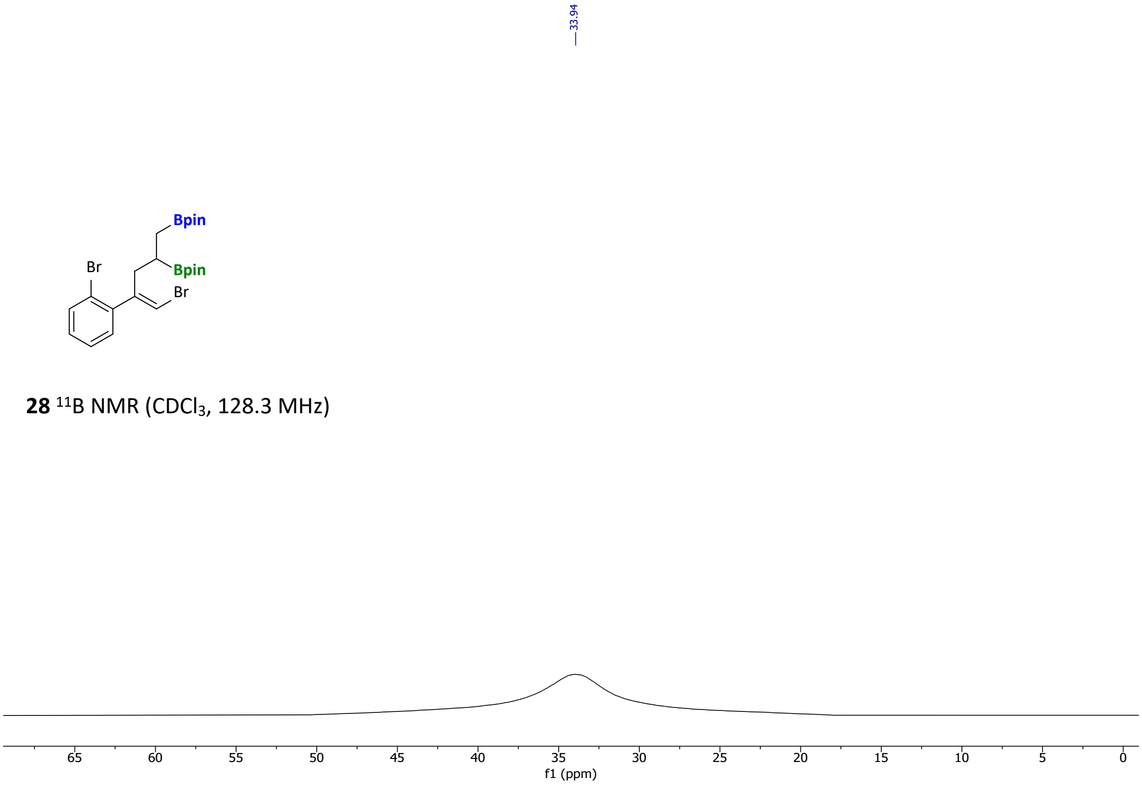


**28** $^{11}B$ NMR ($CDCl_3$, 128.3 MHz)

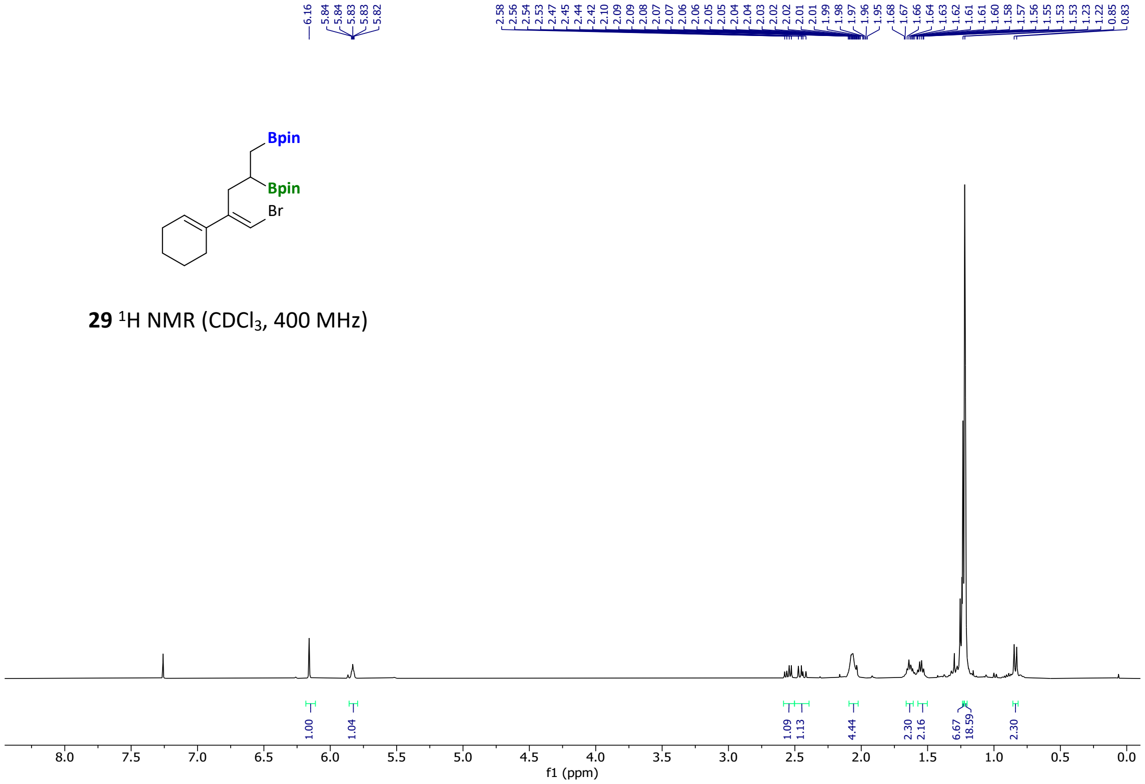

**29** $^{1}H$ NMR ($CDCl_3$, 400 MHz)

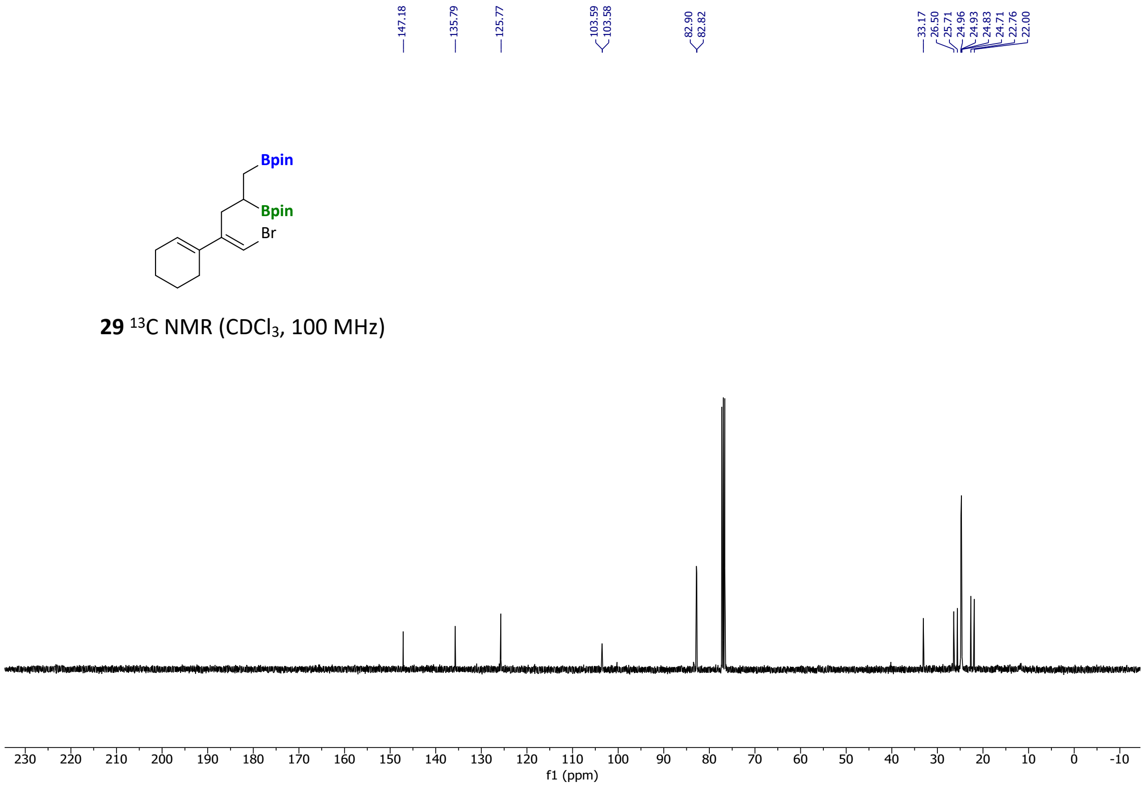

**29** $^{13}C$ NMR ($CDCl_3$, 100 MHz)

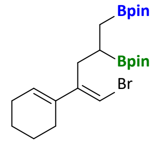


**29** $^{11}B$ NMR ($CDCl_3$, 128.3 MHz)

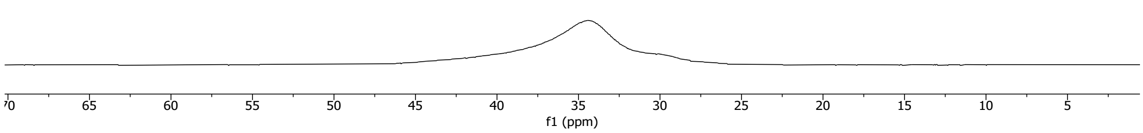

## - $^{1}$H, $^{13}$C, $^{11}$B Spectra for cross-coupling products and corresponding alcohols

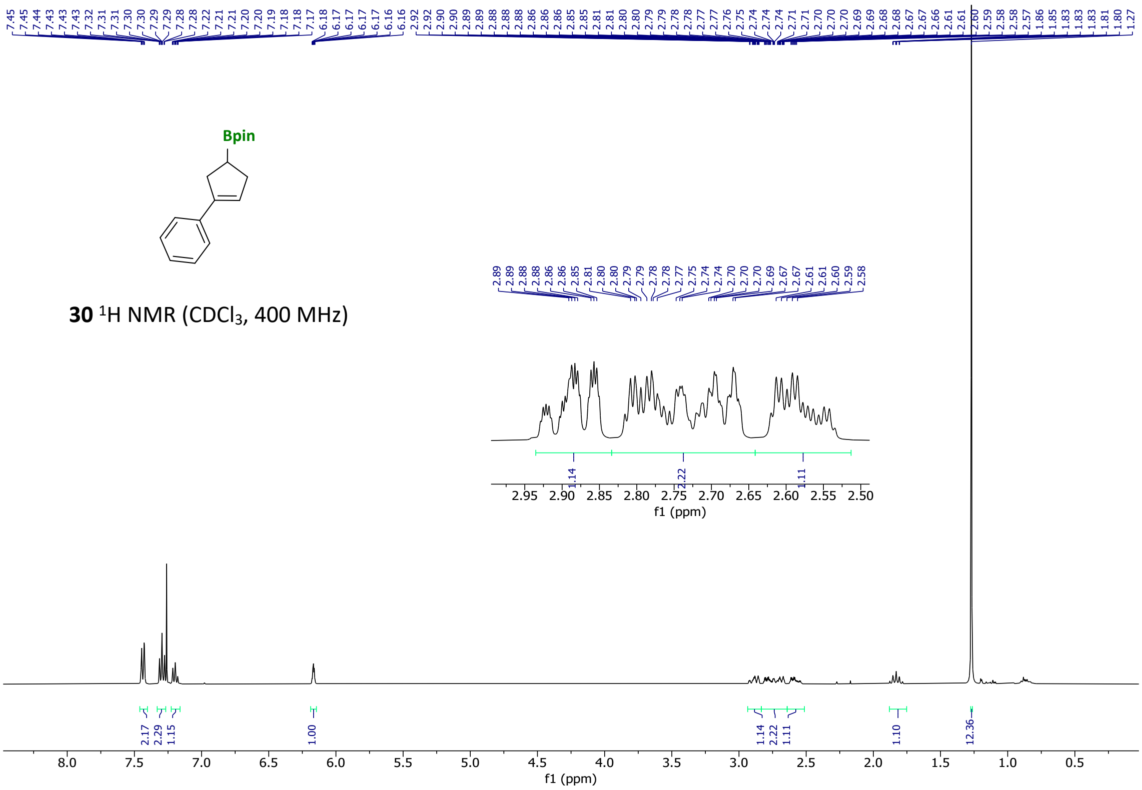


**30** $^{1}$H NMR ($CDCl_3$, 400 MHz)

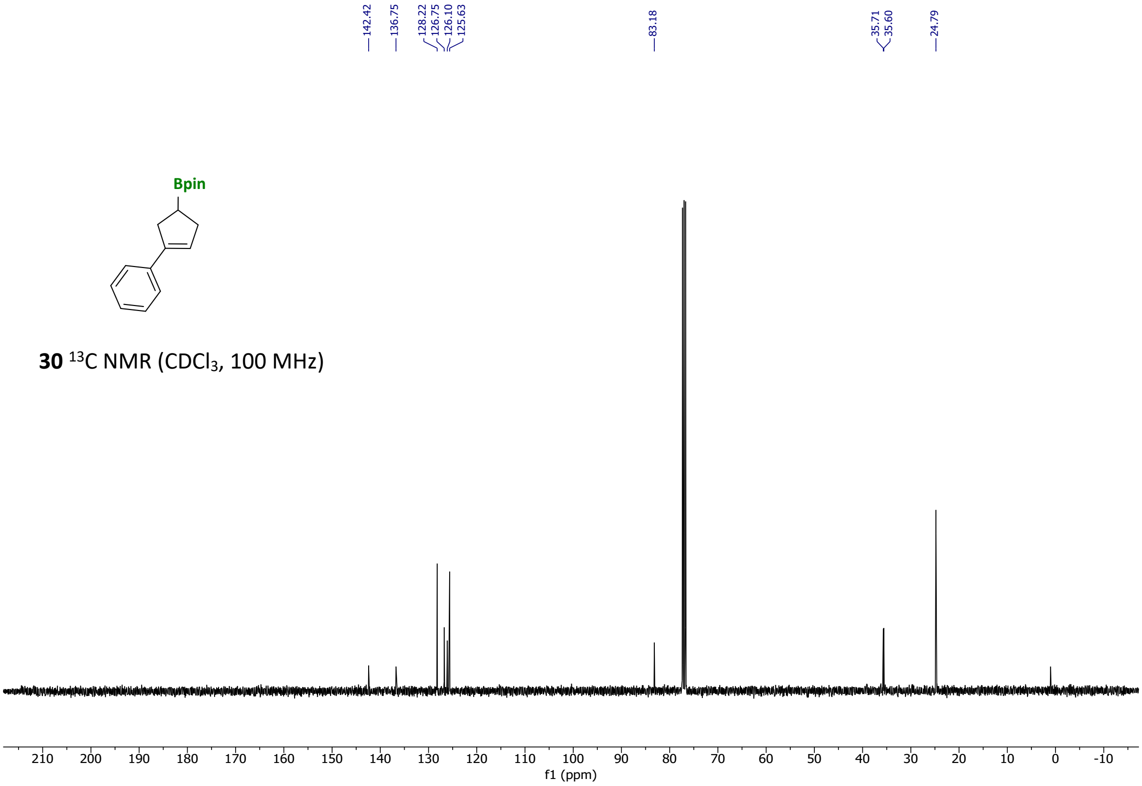


**30** $^{13}$C NMR ($CDCl_3$, 100 MHz)

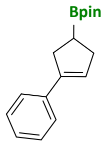


**30** $^{11}$B NMR ($CDCl_3$, 128.3 MHz)

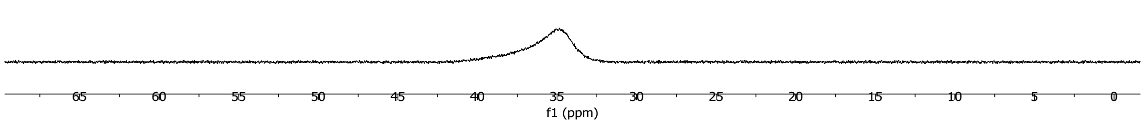


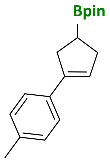


**31** $^{1}$H NMR ($CDCl_3$, 400 MHz)

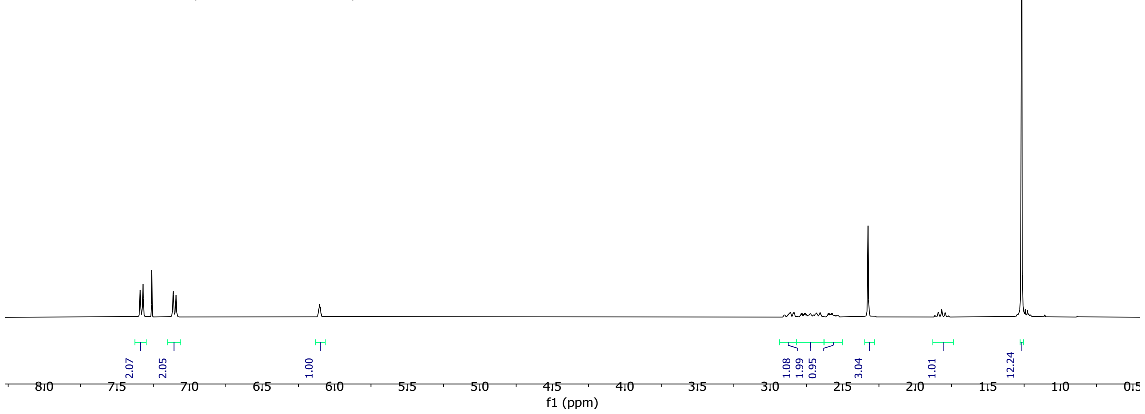

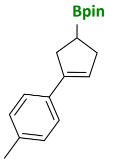

**31** $^{13}$C NMR (CDCl$_3$, 100 MHz)

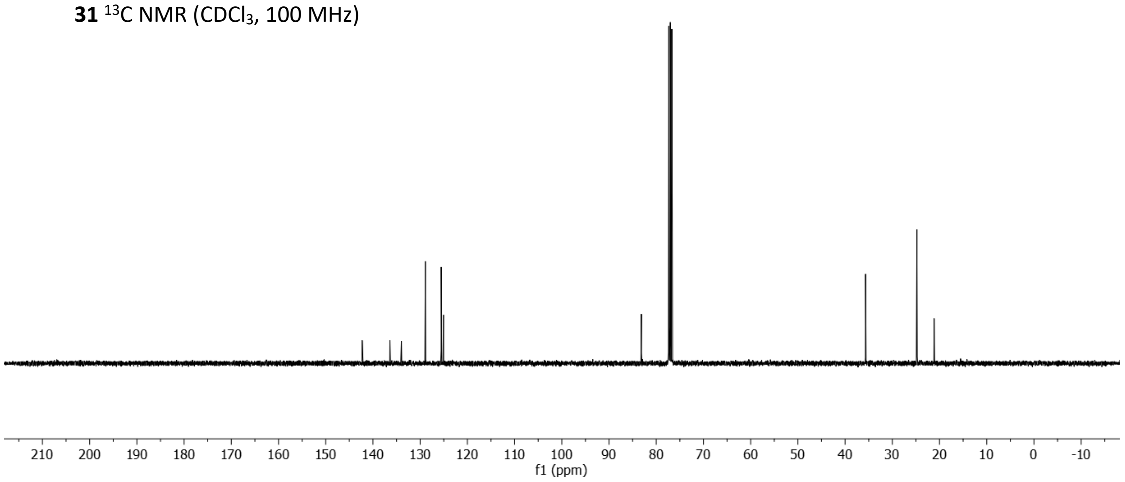


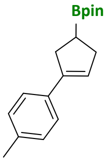

**31** $^{11}$B NMR (CDCl$_3$, 128.3 MHz)

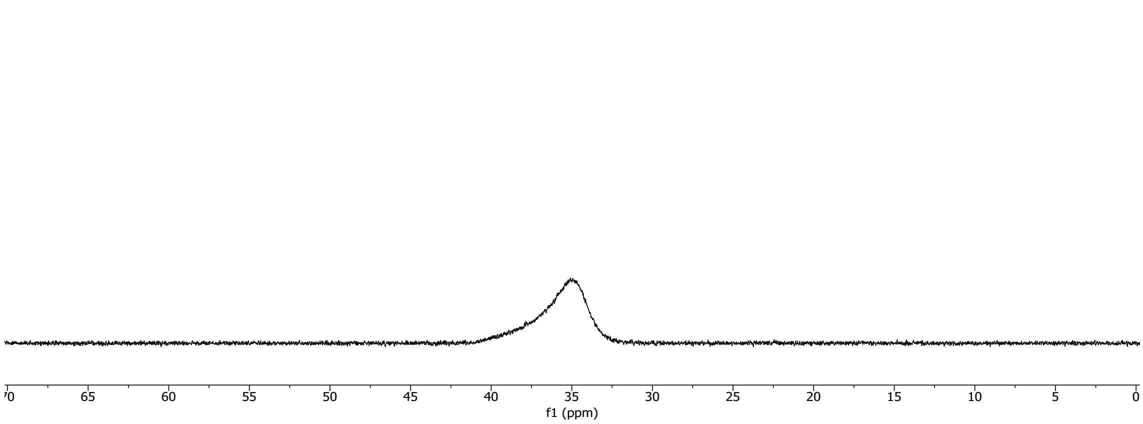

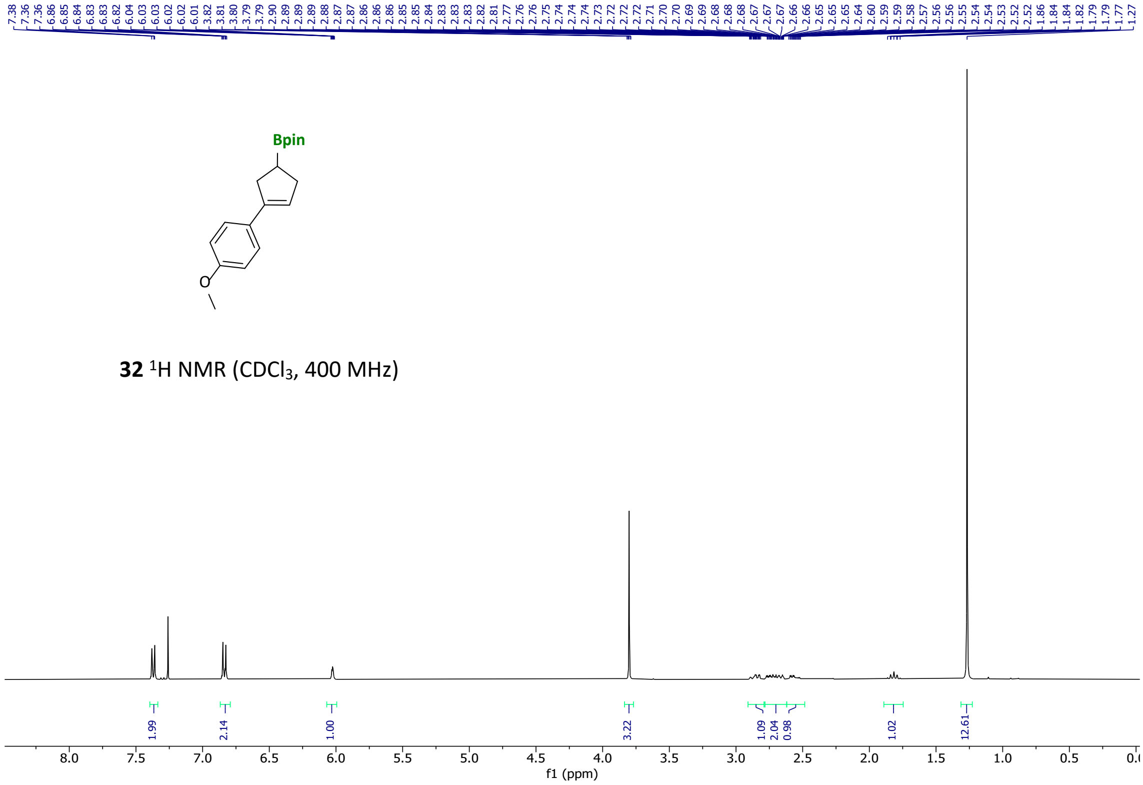
7.38 7.36 7.36 6.86 6.85 6.84 6.83 6.83 6.82 6.04 6.03 6.03 6.02 6.02 6.01 3.82 3.81 3.80 3.79 3.79 2.90 2.89 2.89 2.89 2.88 2.87 2.87 2.86 2.86 2.85 2.85 2.84 2.83 2.83 2.83 2.82 2.81 2.77 2.76 2.76 2.75 2.74 2.74 2.73 2.72 2.72 2.72 2.71 2.70 2.69 2.69 2.68 2.68 2.68 2.67 2.67 2.67 2.66 2.66 2.65 2.65 2.65 2.64 2.60 2.59 2.59 2.58 2.57 2.56 2.56 2.55 2.54 2.54 2.53 2.52 2.52 1.86 1.84 1.84 1.82 1.79 1.79 1.77 1.27
Bpin
O
32 1H NMR (CDCl3, 400 MHz)
1.99
2.14
1.00
3.22
1.09
2.04
0.98
1.02
12.61
f1 (ppm)

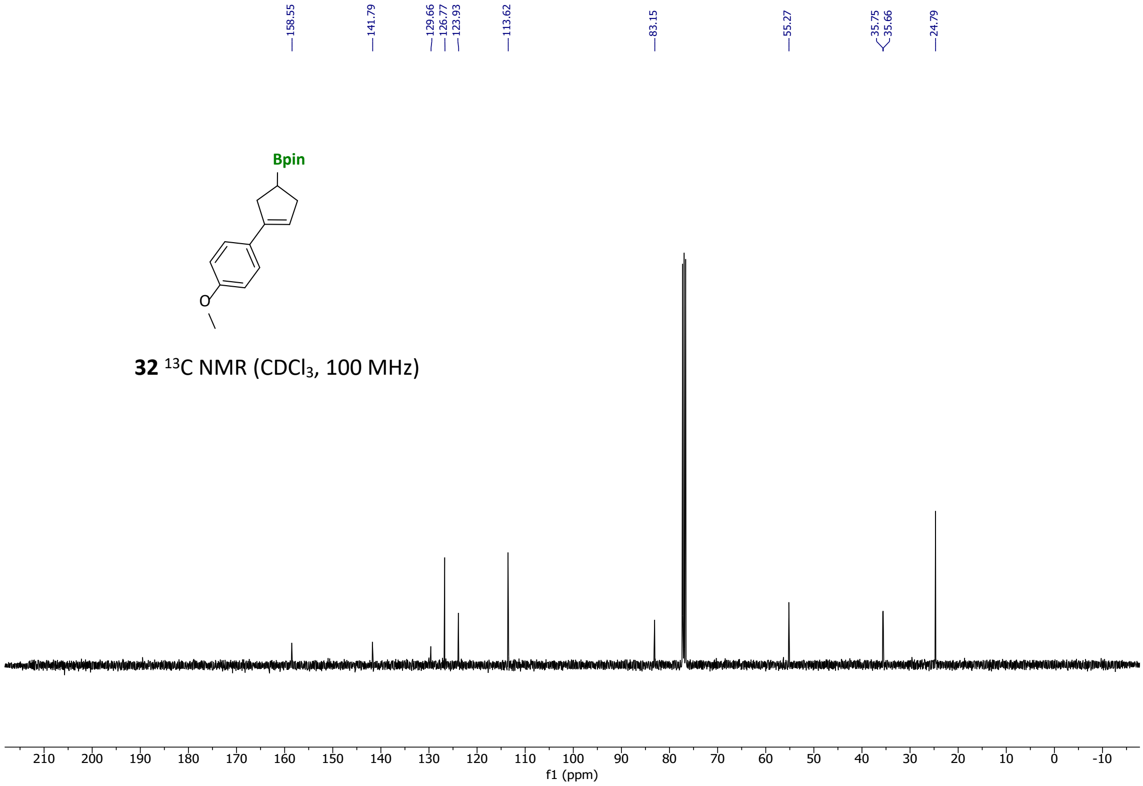
158.55
141.79
129.66
126.77
123.93
113.62
83.15
55.27
35.75
35.66
24.79
Bpin
O
32 13C NMR (CDCl3, 100 MHz)
f1 (ppm)

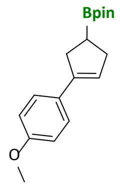


**32** $^{11}$B NMR ($CDCl_3$, 128.3 MHz)

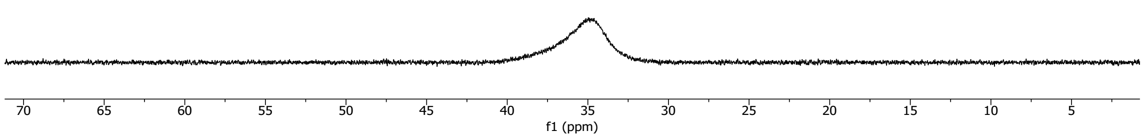


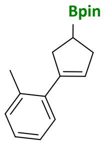


**33** $^{1}$H NMR ($CDCl_3$, 400 MHz)

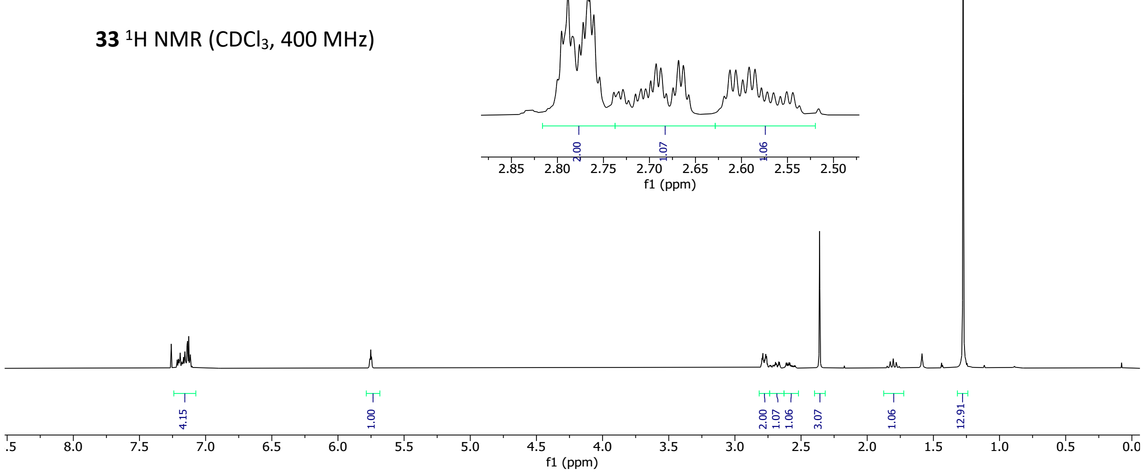

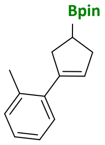


**33** $^{13}$C NMR ($CDCl_3$, 100 MHz)

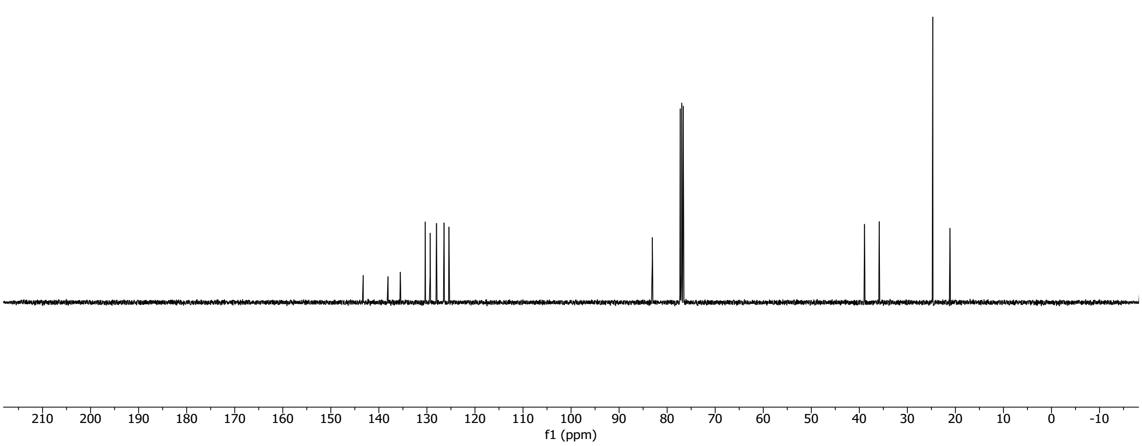


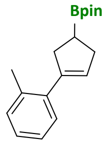


**33** $^{11}$B NMR ($CDCl_3$, 128.3 MHz)

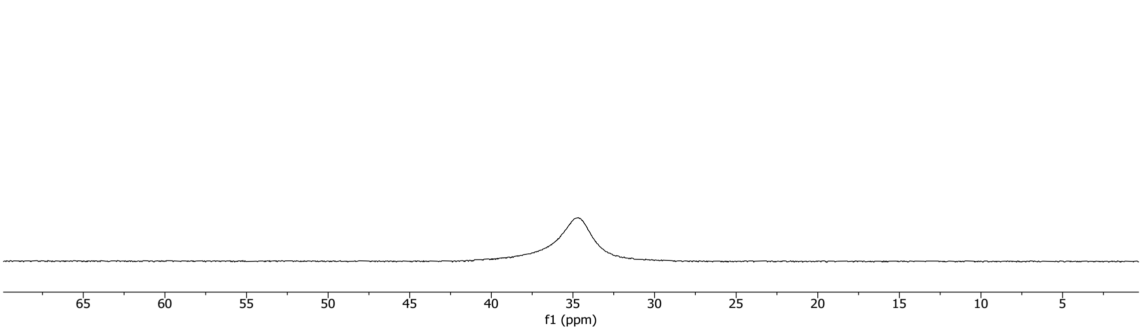

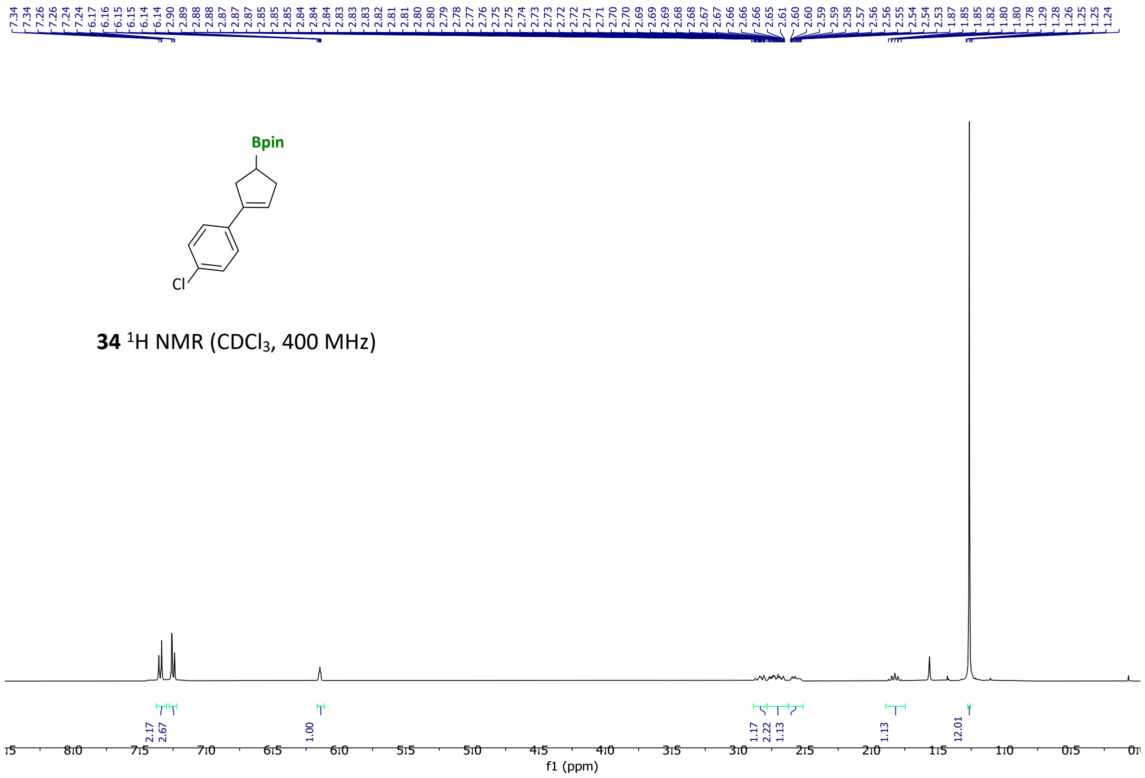


**34** $^{1}$H NMR ($CDCl_3$, 400 MHz)

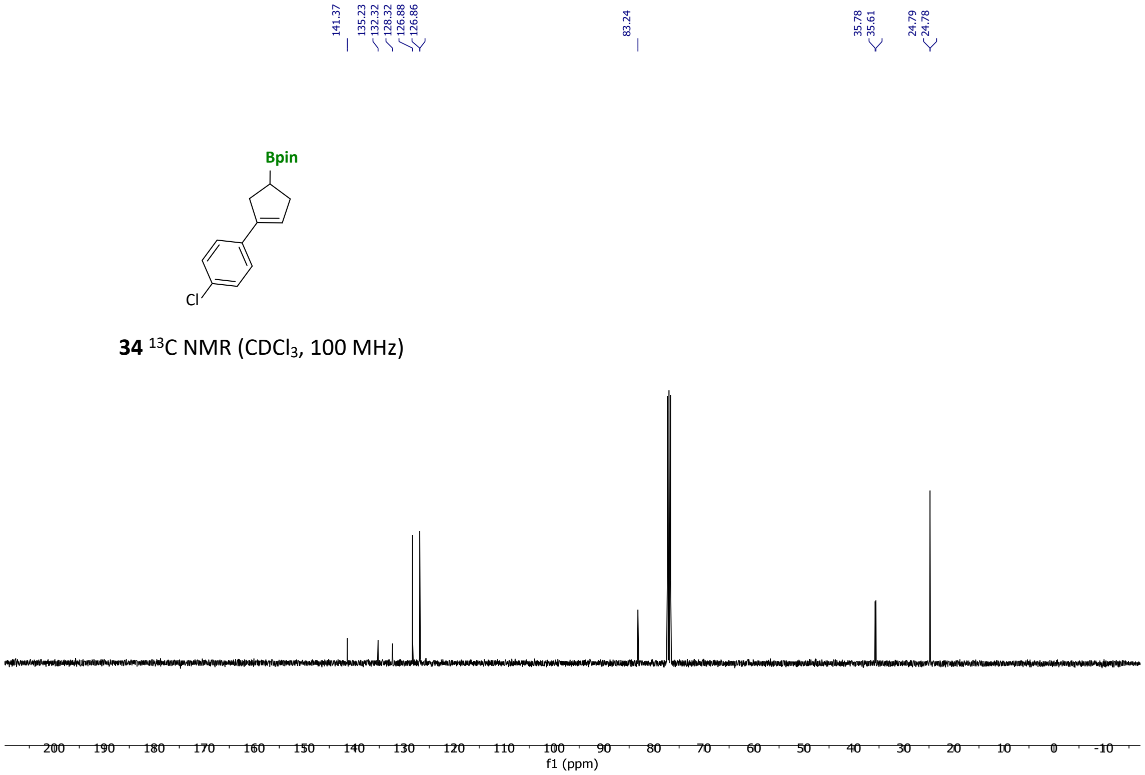


**34** $^{13}$C NMR ($CDCl_3$, 100 MHz)

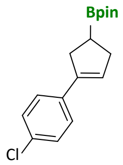


**34** $^{11}$B NMR ($CDCl_3$, 128.3 MHz)

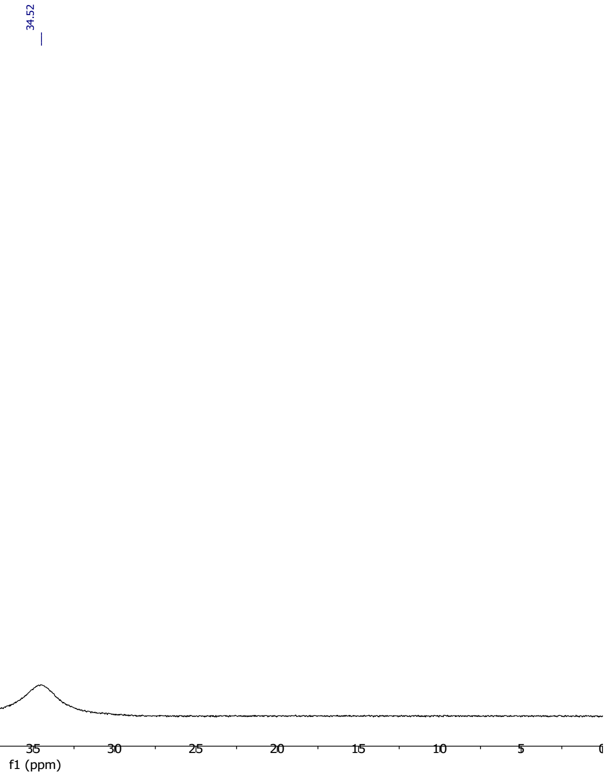


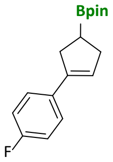


**35** $^{1}$H NMR ($CDCl_3$, 400 MHz)

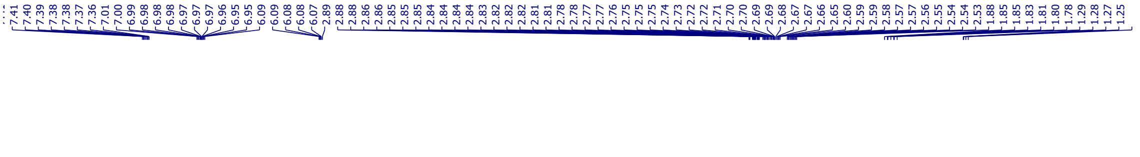

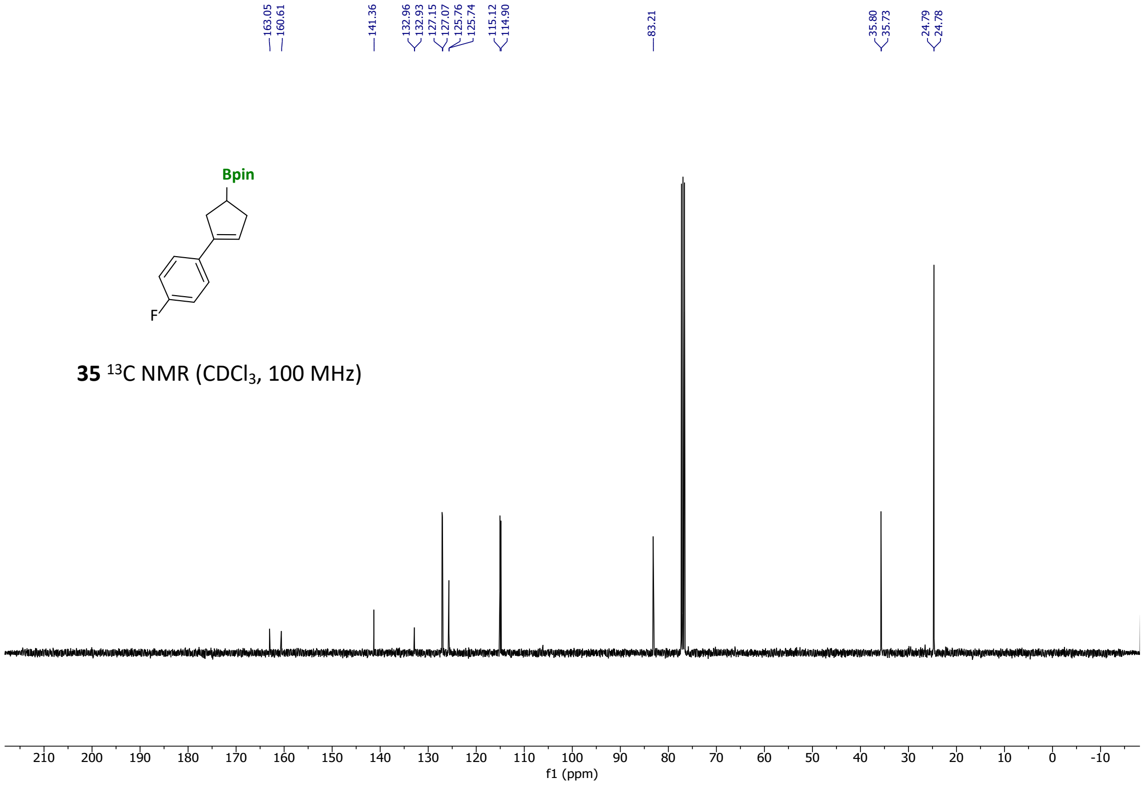


**35** $^{13}$C NMR ($CDCl_3$, 100 MHz)

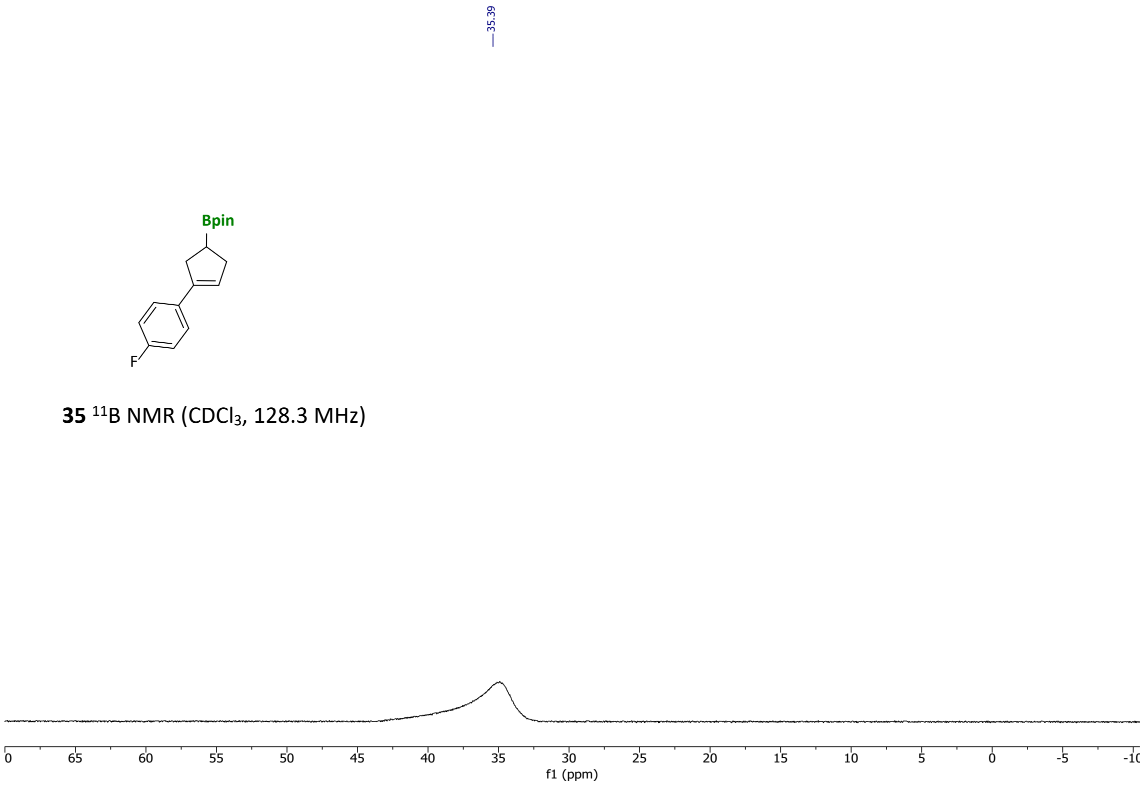


**35** $^{11}$B NMR ($CDCl_3$, 128.3 MHz)

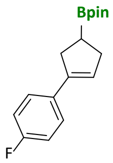


**35** $^{19}F$ NMR ($CDCl_3$, 377 MHz)

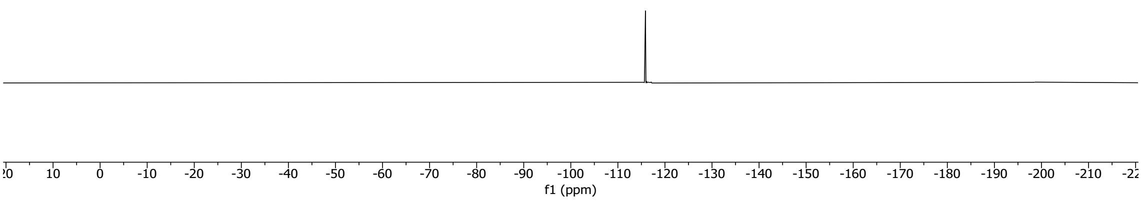


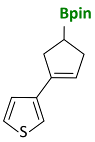


**36** $^{1}H$ NMR ($CDCl_3$, 400 MHz)

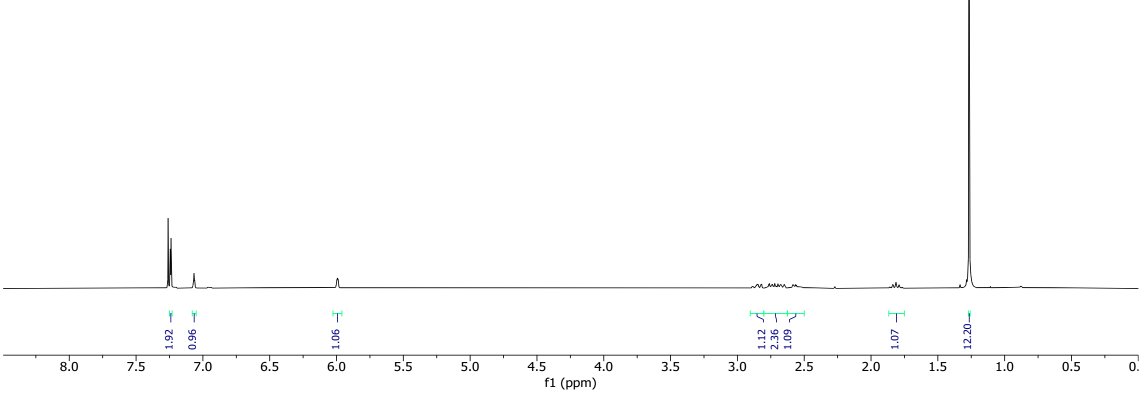

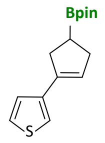


**36** $^{13}C$ NMR ($CDCl_3$, 100 MHz)

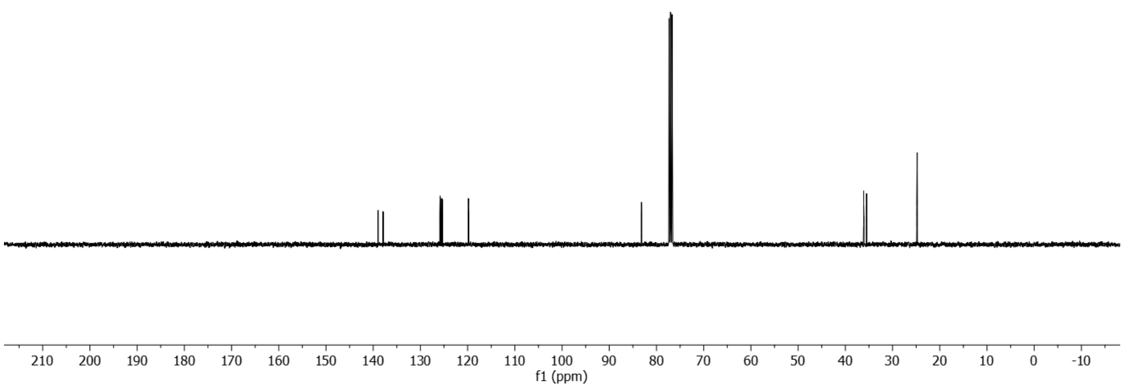


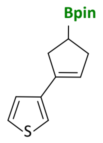


**36** $^{11}B$ NMR ($CDCl_3$, 128.3 MHz)

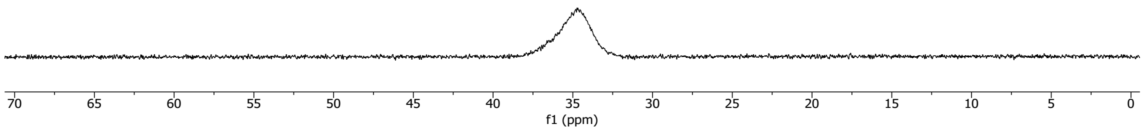

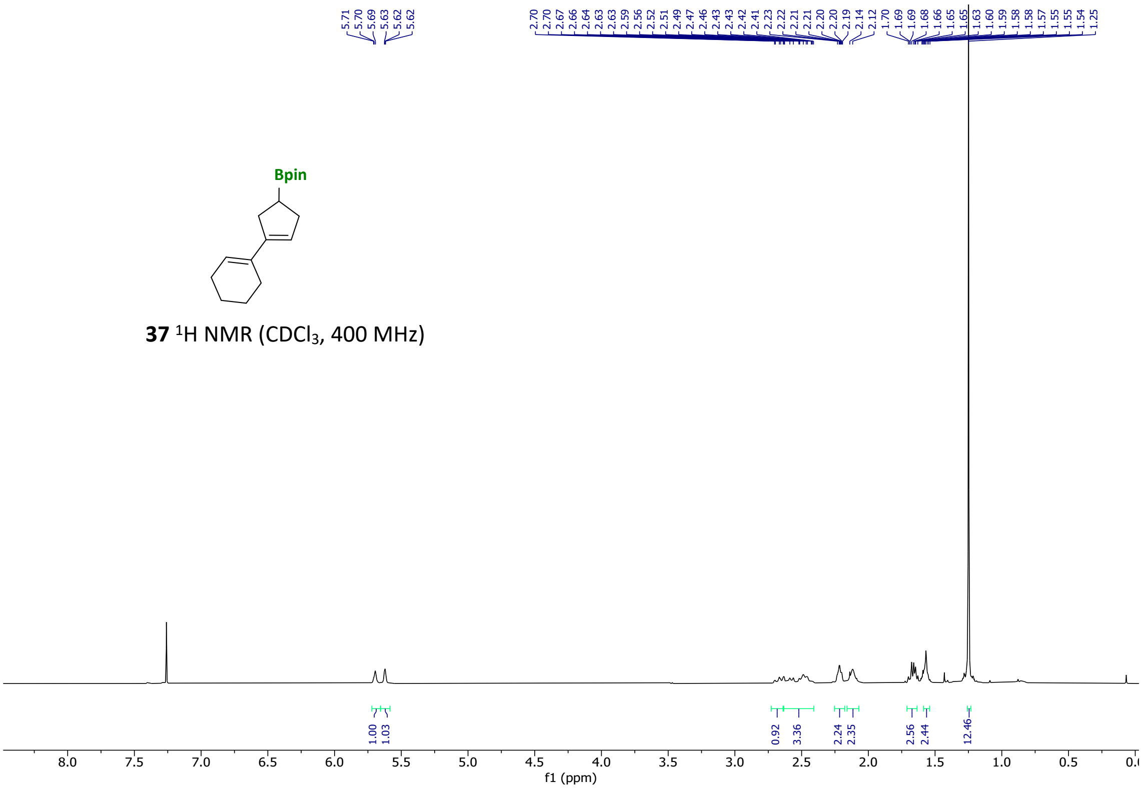


**37** $^{1}H$ NMR ($CDCl_3$, 400 MHz)

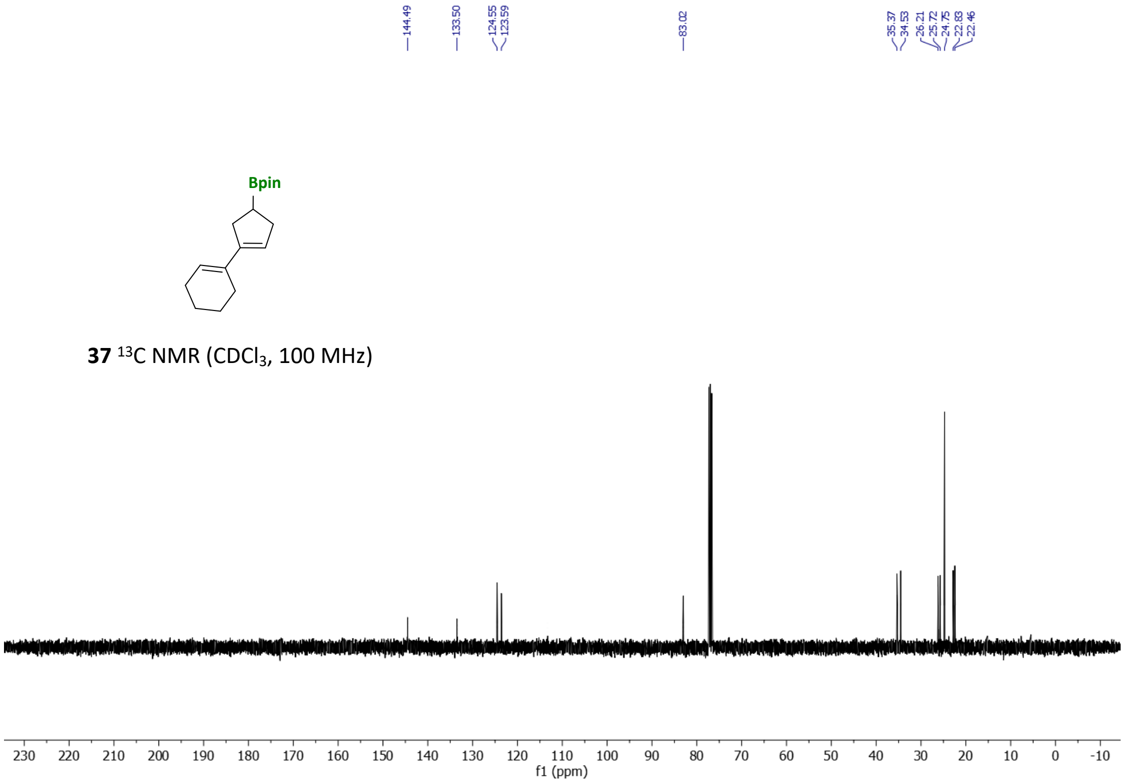


**37** $^{13}C$ NMR ($CDCl_3$, 100 MHz)

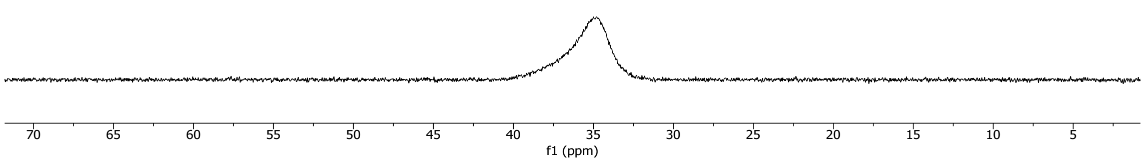


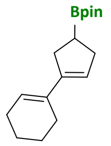


**37** $^{11}$B NMR ($CDCl_3$, 128.3 MHz)

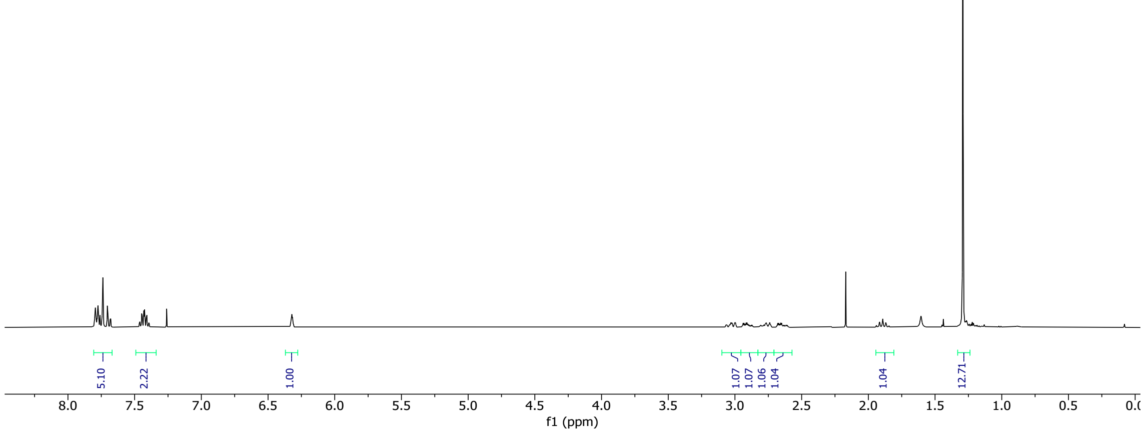


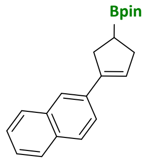


**38** $^{1}$H NMR ($CDCl_3$, 400 MHz)

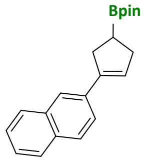

**38** $^{13}$C NMR (CDCl$_3$, 100 MHz)

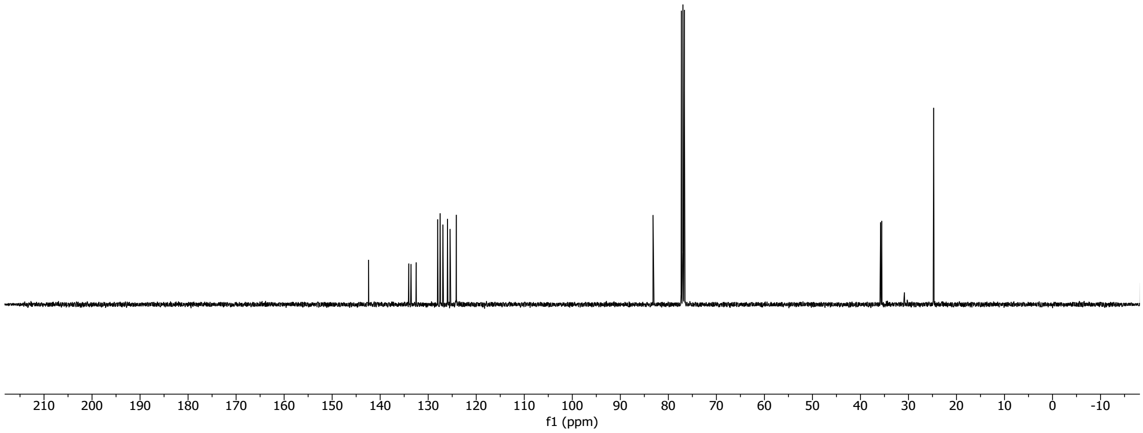


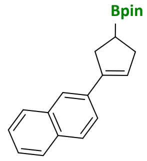

**38** $^{11}$B NMR (CDCl$_3$, 128.3 MHz)

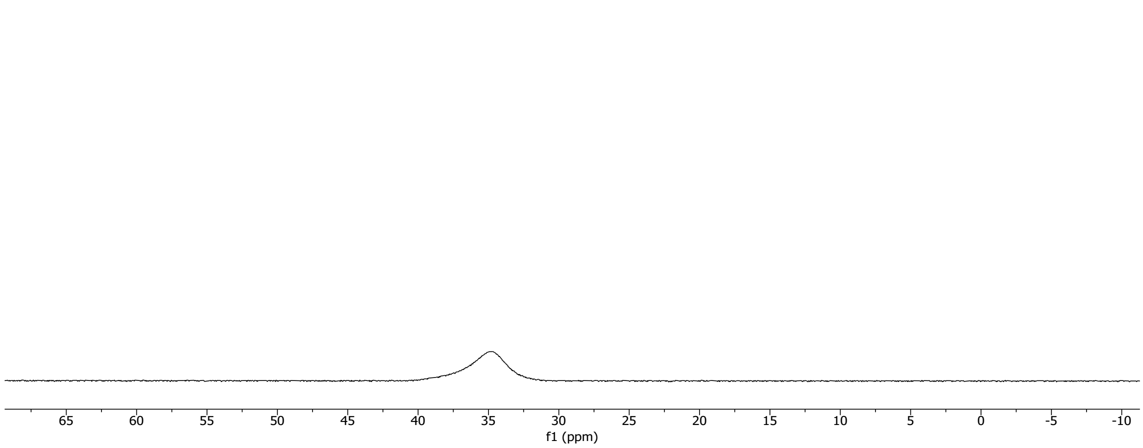

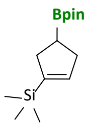


**39** $^{1}$H NMR ($CDCl_3$, 400 MHz)

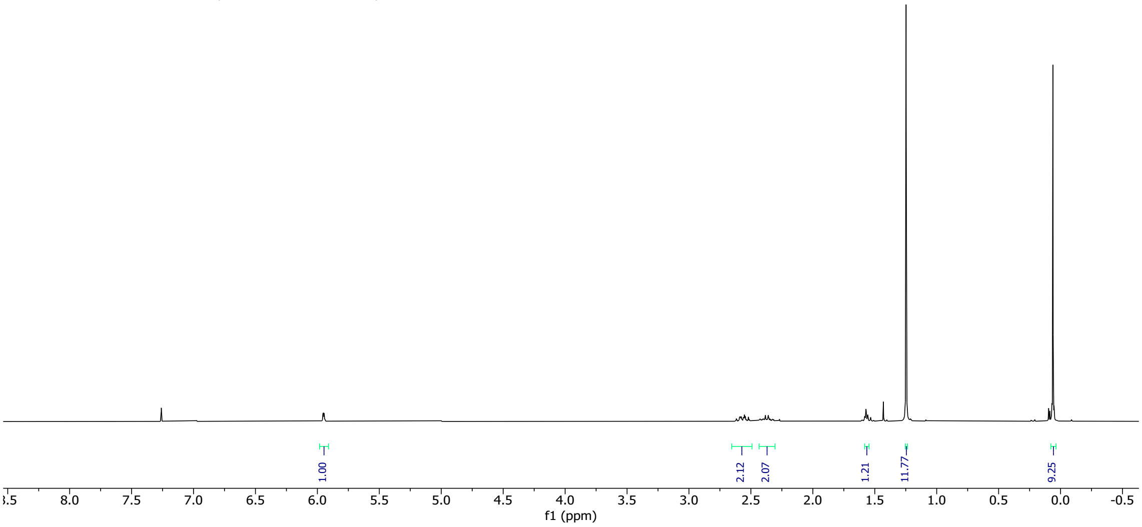


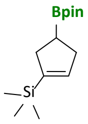


**39** $^{13}$C NMR ($CDCl_3$, 100 MHz)

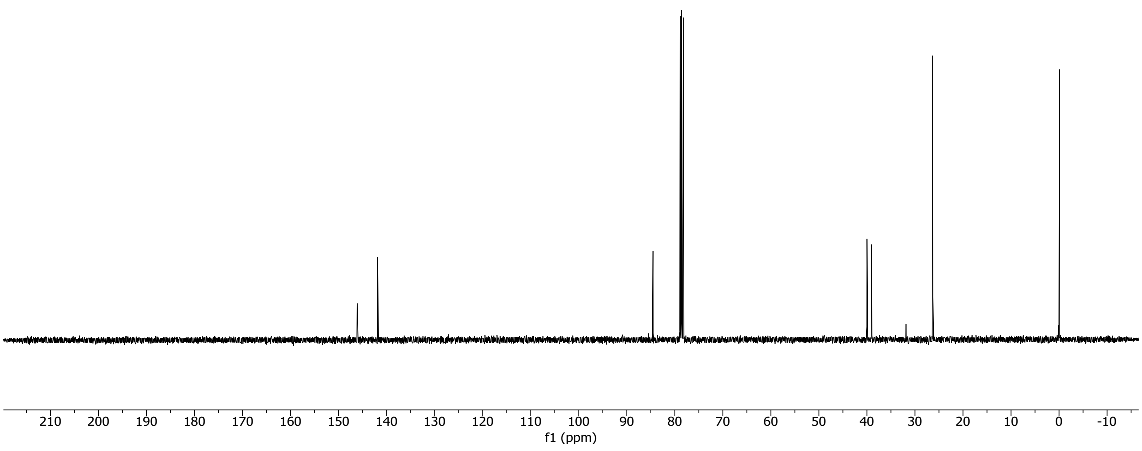

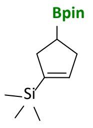


**39** $^{11}$B NMR ($CDCl_3$, 128.3 MHz)

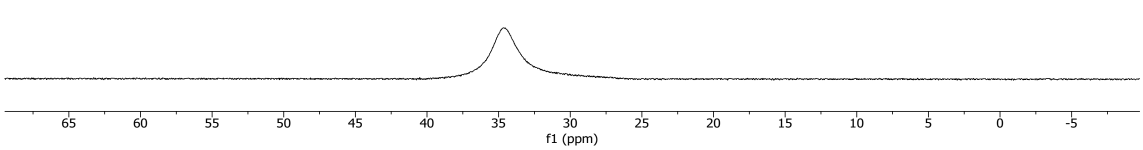


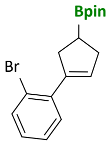


**50** $^{1}$H NMR ($CDCl_3$, 400 MHz)

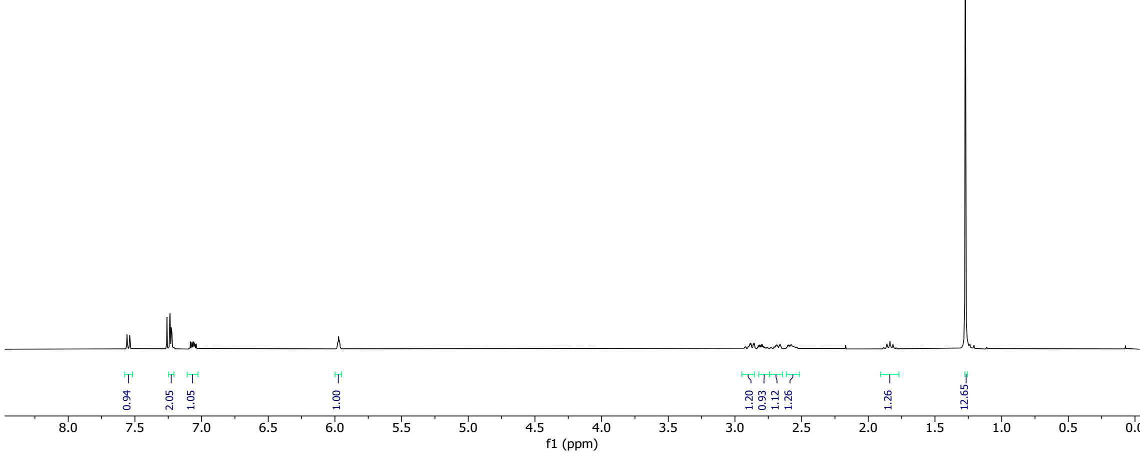

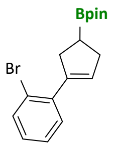


**50** $^{13}C$ NMR ($CDCl_3$, 100 MHz)

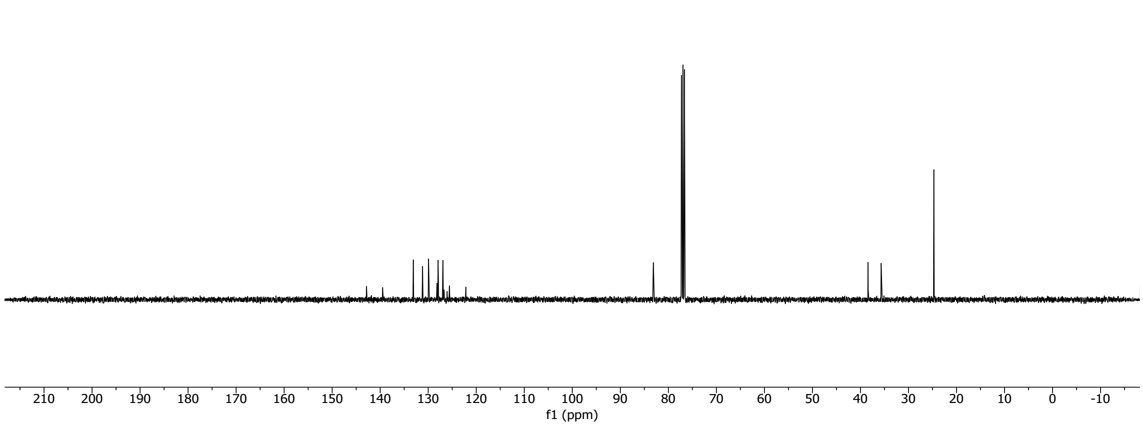


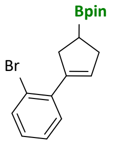


**50** $^{11}B$ NMR ($CDCl_3$, 128.3 MHz)

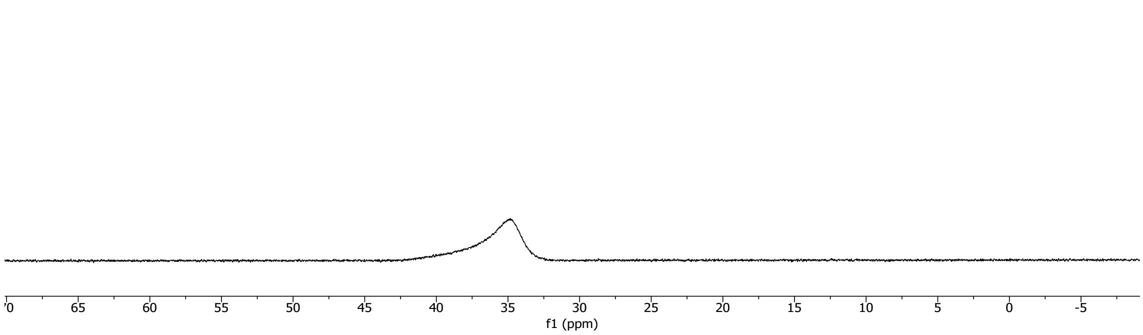

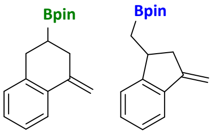

**52** / **53** $^{1}$H NMR ($CDCl_3$, 400 MHz)

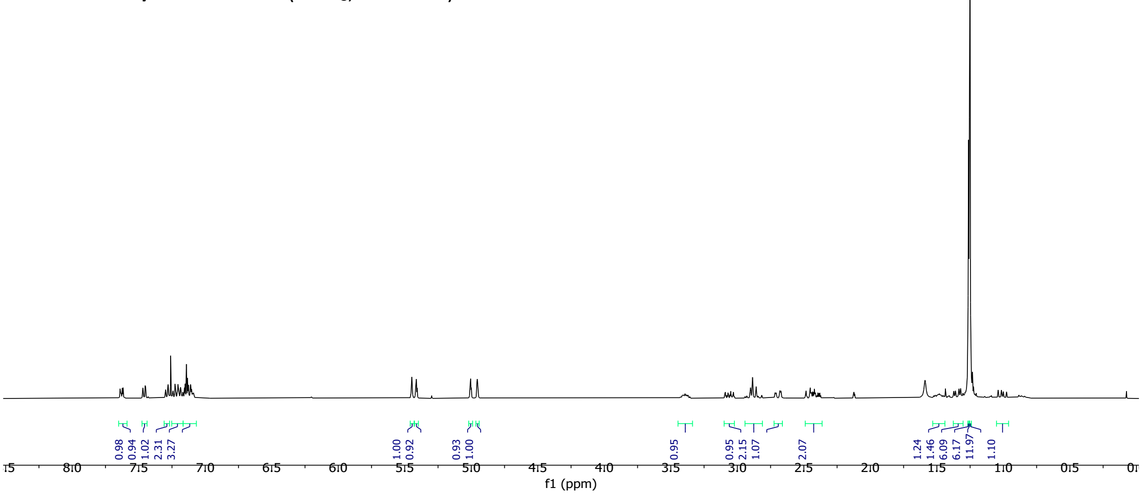


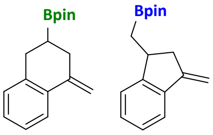

**52** / **53** $^{13}$C NMR ($CDCl_3$, 100 MHz)

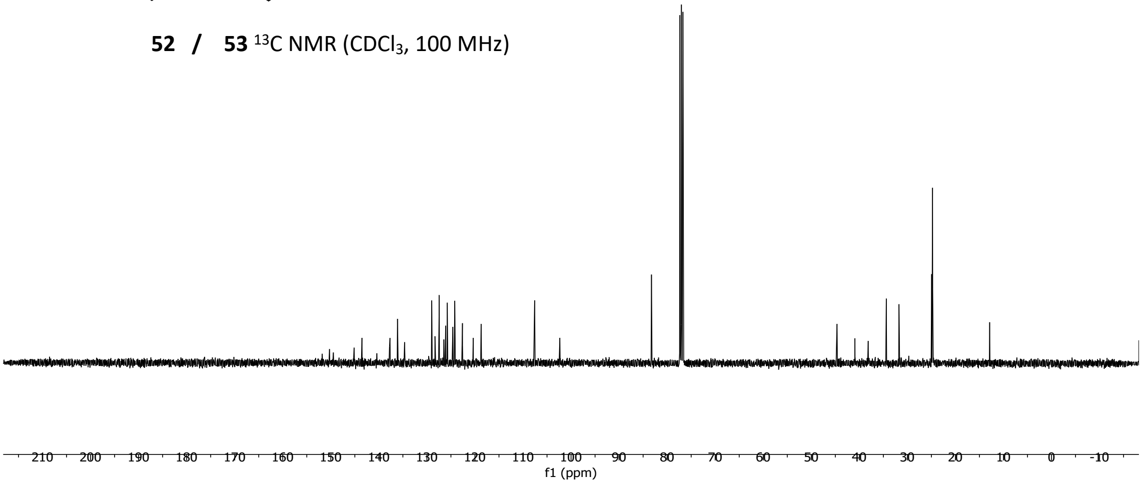

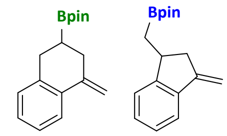


**52** / **53** $^{11}B$ NMR ($CDCl_3$, 128.3 MHz)

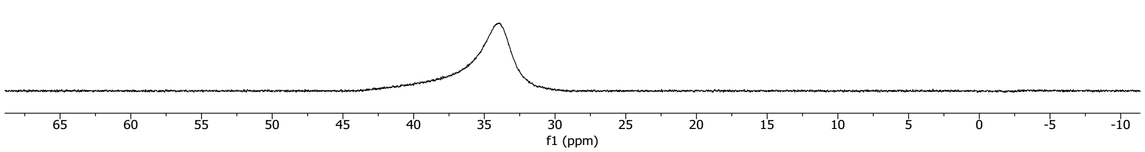

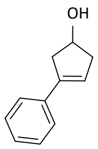


**40** $^{1}$H NMR ($CDCl_3$, 400 MHz)

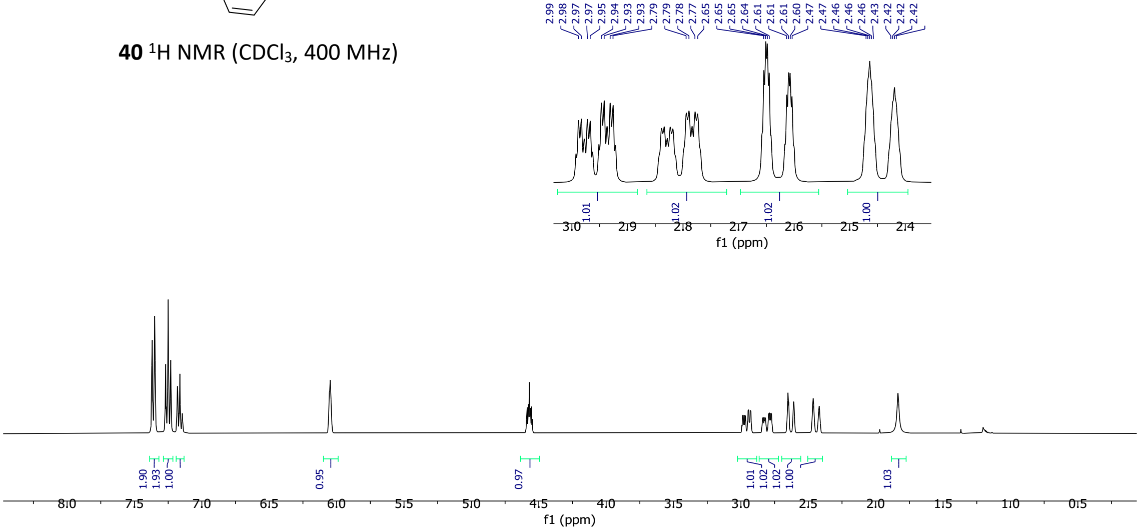


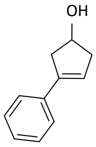


**40** $^{13}$C NMR ($CDCl_3$, 100 MHz)

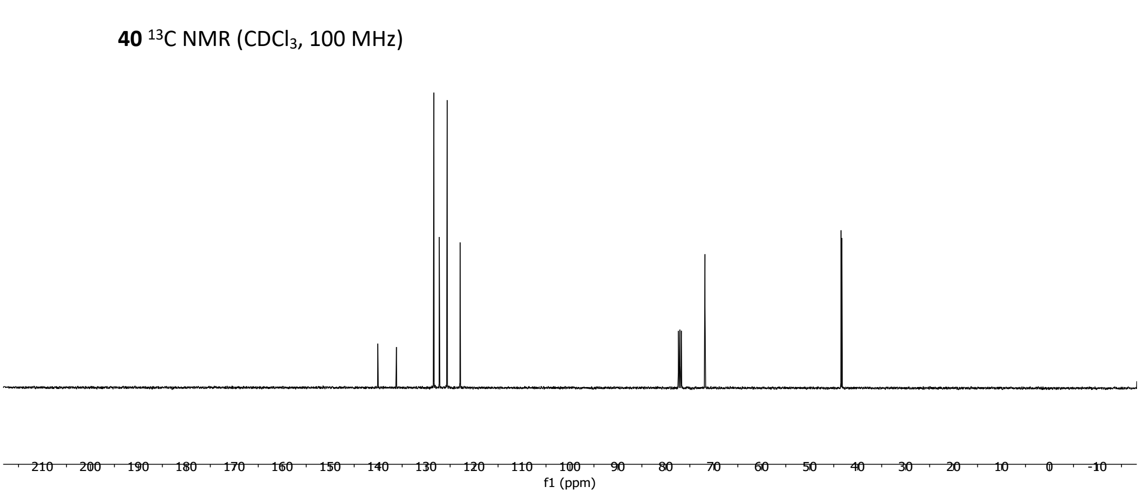

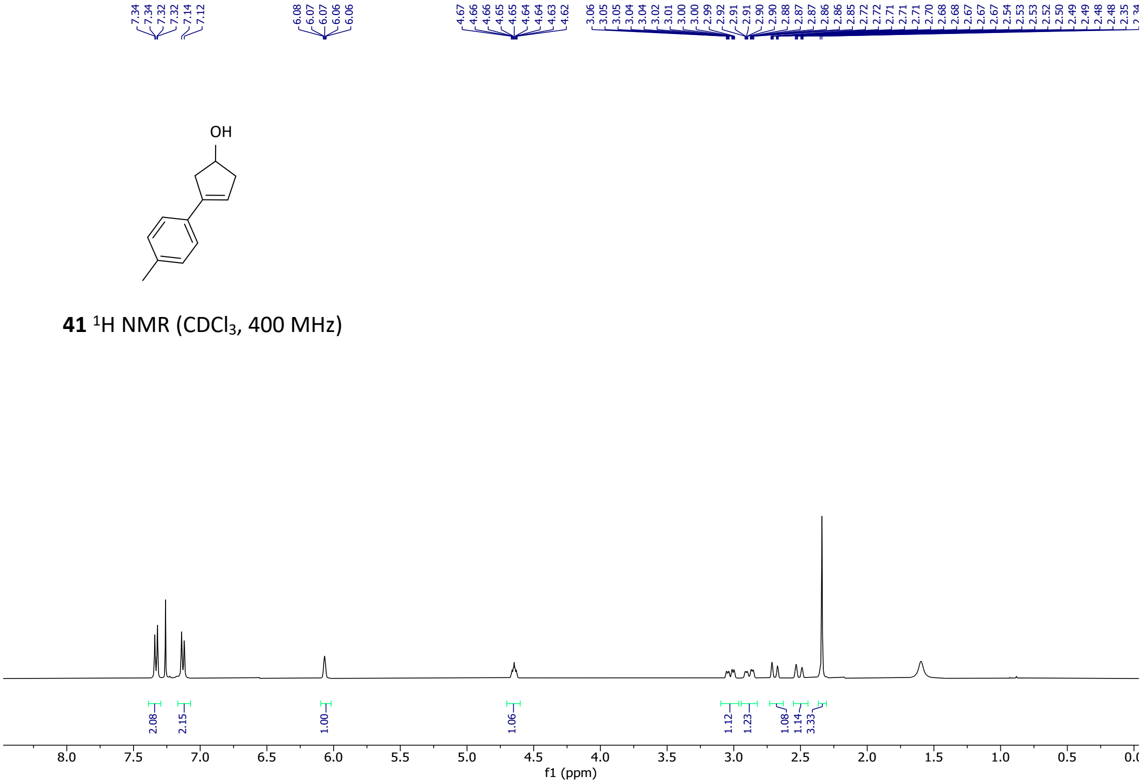


**41** $^{1}$H NMR ($CDCl_3$, 400 MHz)

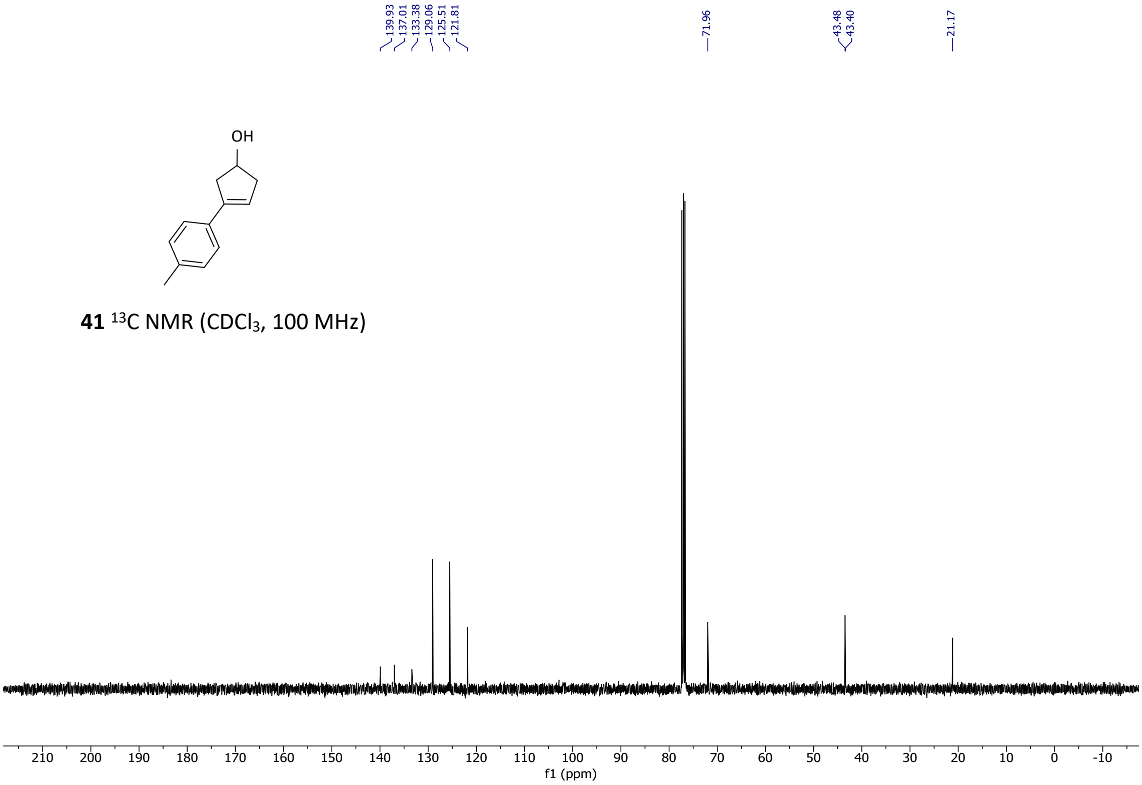


**41** $^{13}$C NMR ($CDCl_3$, 100 MHz)

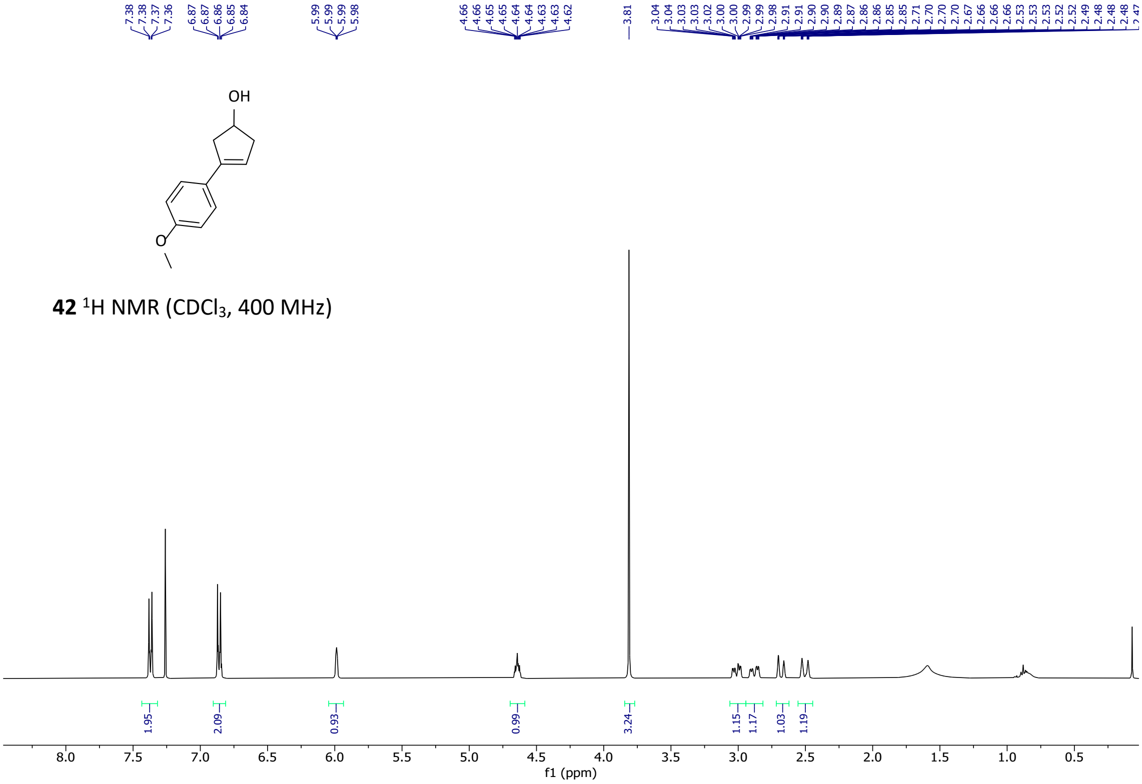


**42** $^{1}$H NMR ($CDCl_3$, 400 MHz)

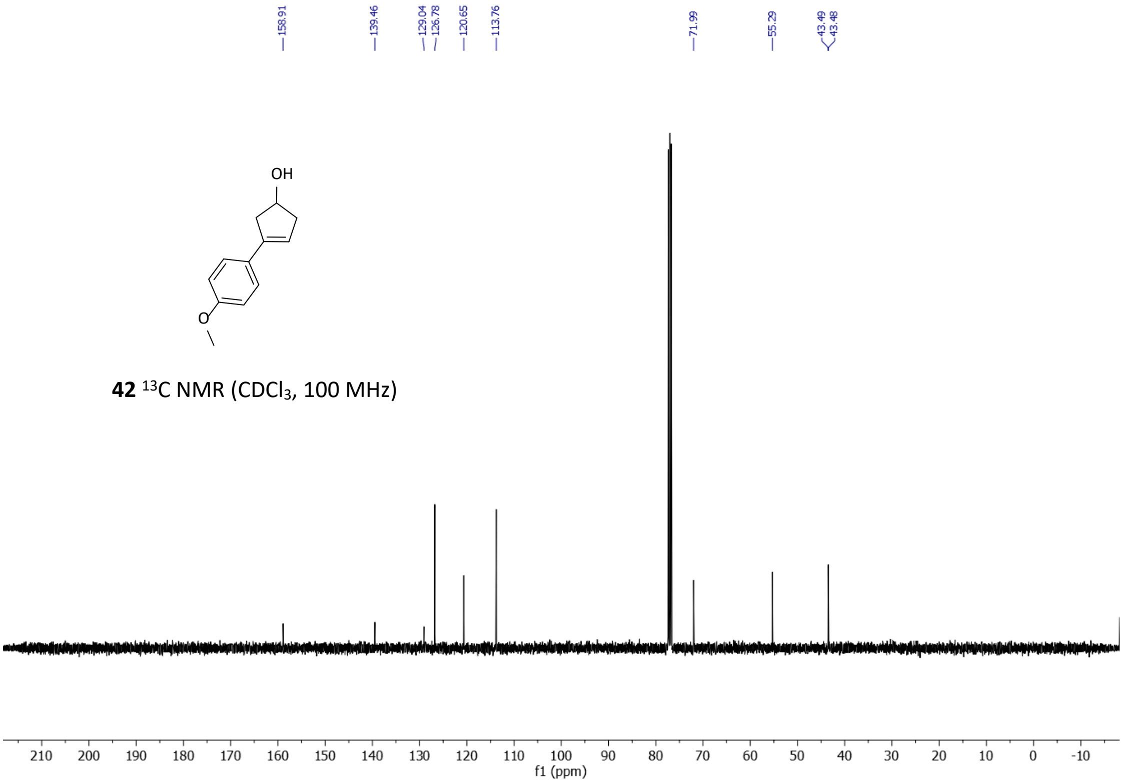


**42** $^{13}$C NMR ($CDCl_3$, 100 MHz)

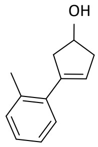

**43** $^{1}$H NMR ($CDCl_3$, 400 MHz)

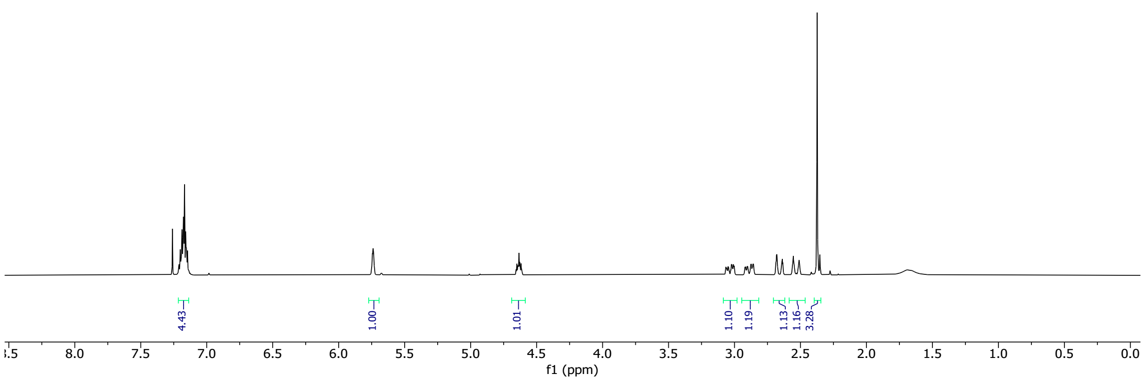


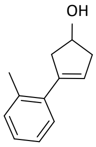

**43** $^{13}$C NMR ($CDCl_3$, 100 MHz)

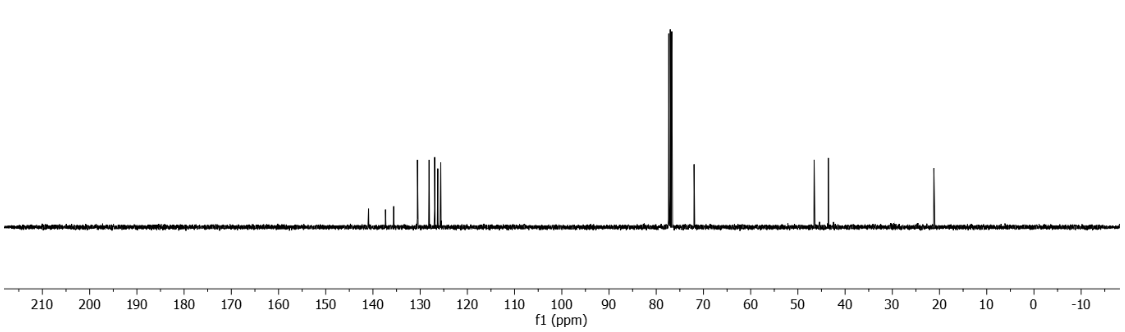

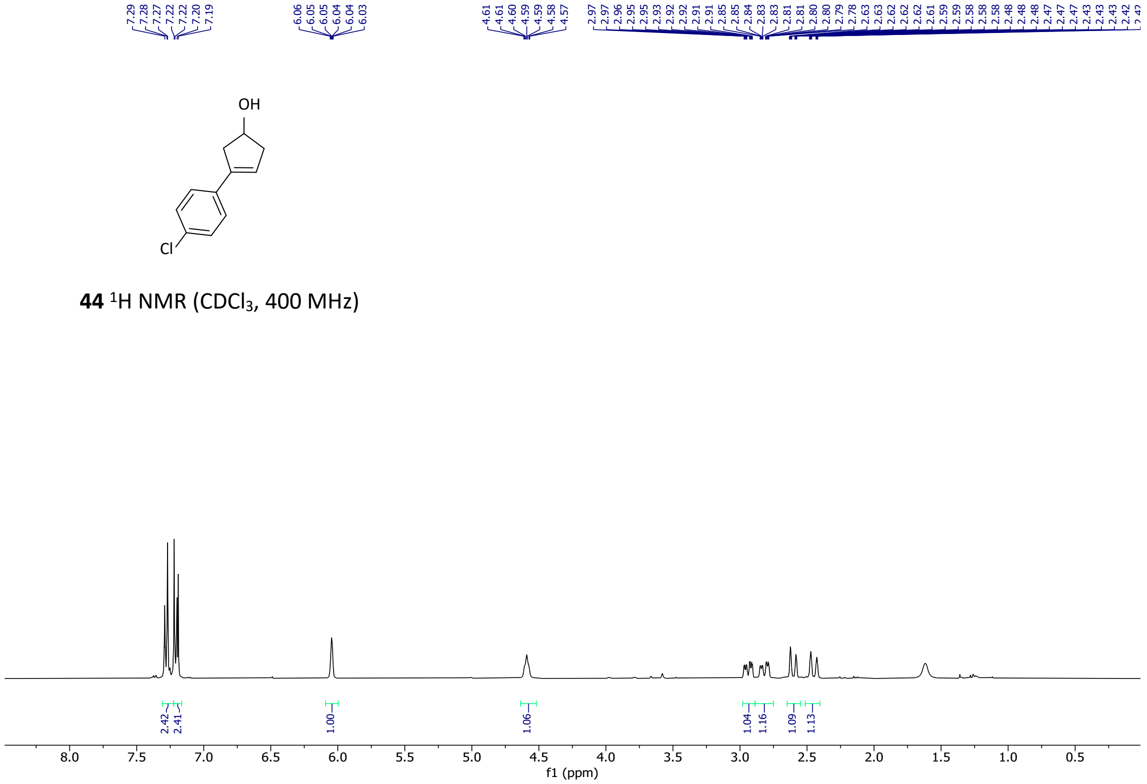


**44** $^{1}$H NMR ($CDCl_3$, 400 MHz)

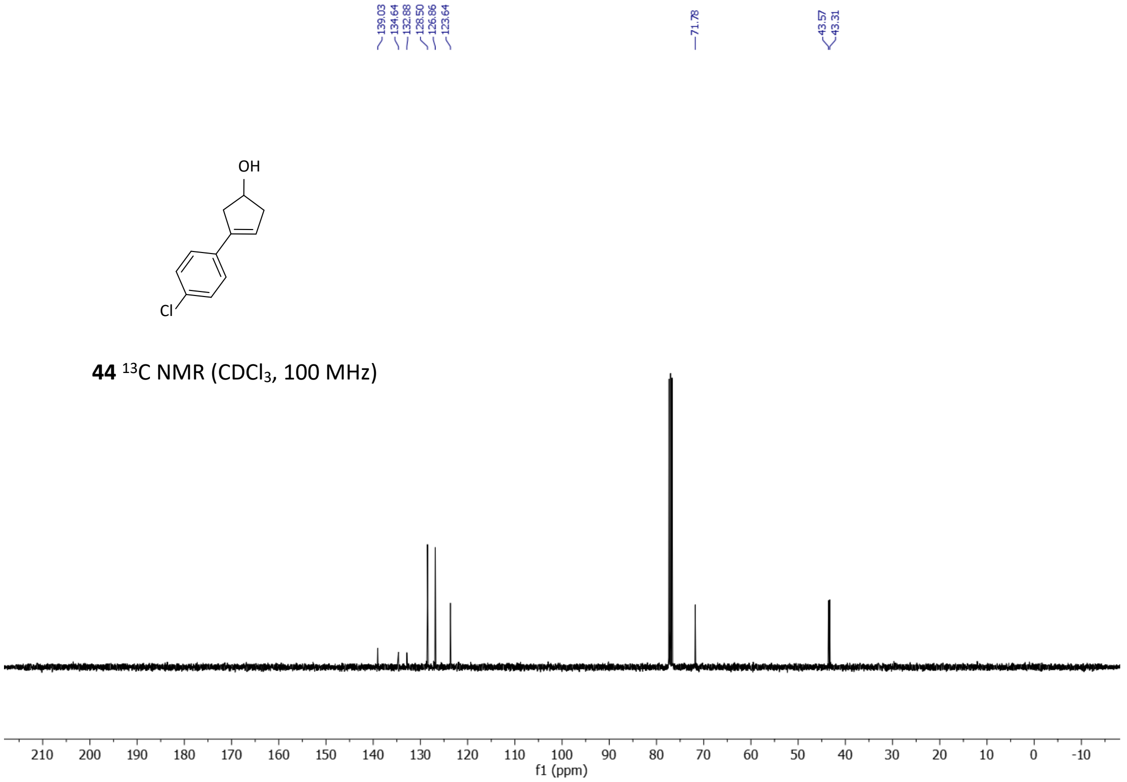


**44** $^{13}$C NMR ($CDCl_3$, 100 MHz)

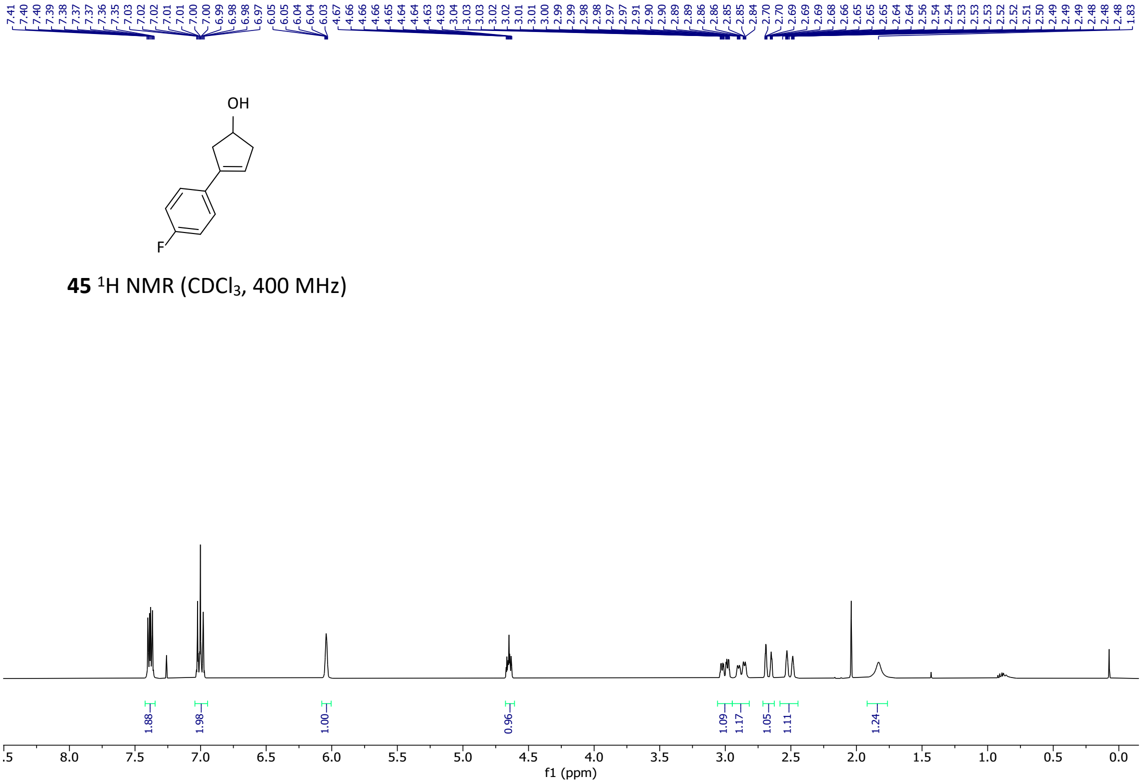


**45** $^{1}$H NMR ($CDCl_3$, 400 MHz)

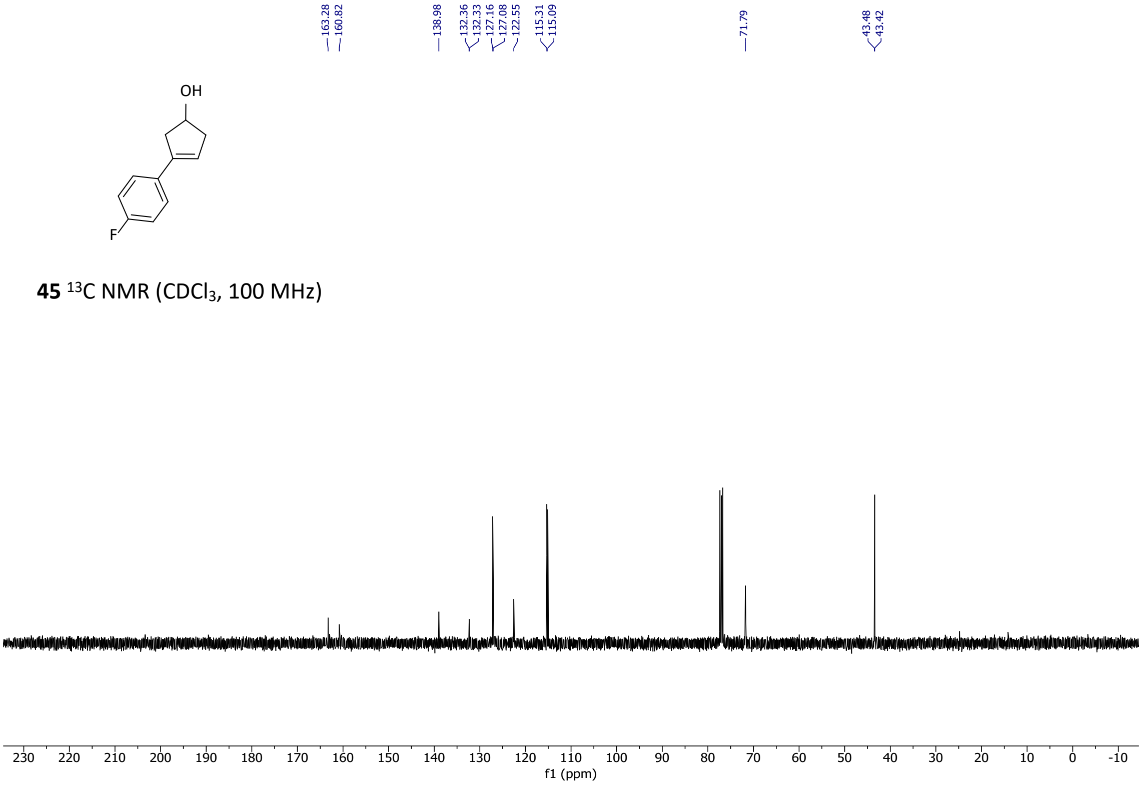


**45** $^{13}$C NMR ($CDCl_3$, 100 MHz)

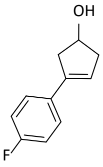


**45** $^{19}$F NMR ($CDCl_3$, 377 MHz)

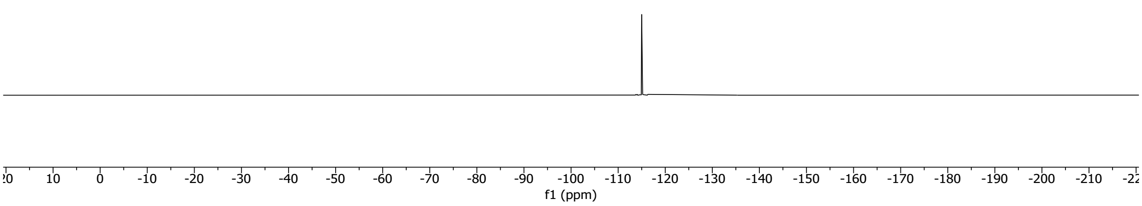


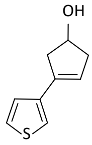


**46** $^{1}$H NMR ($CDCl_3$, 400 MHz)

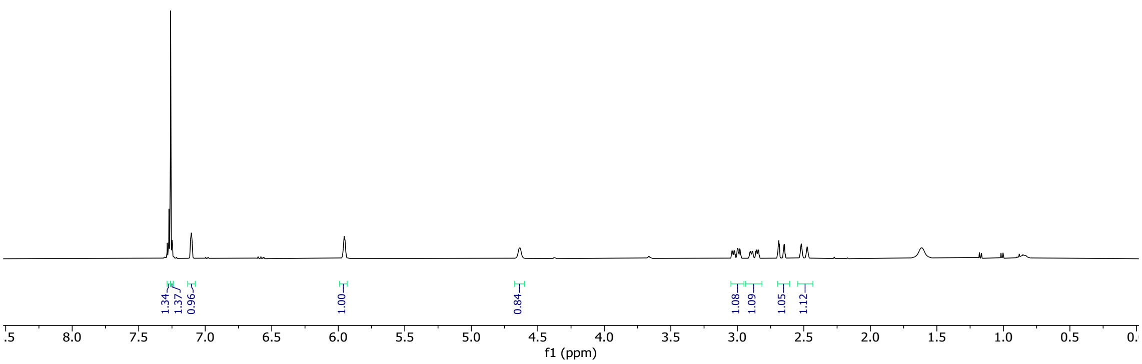

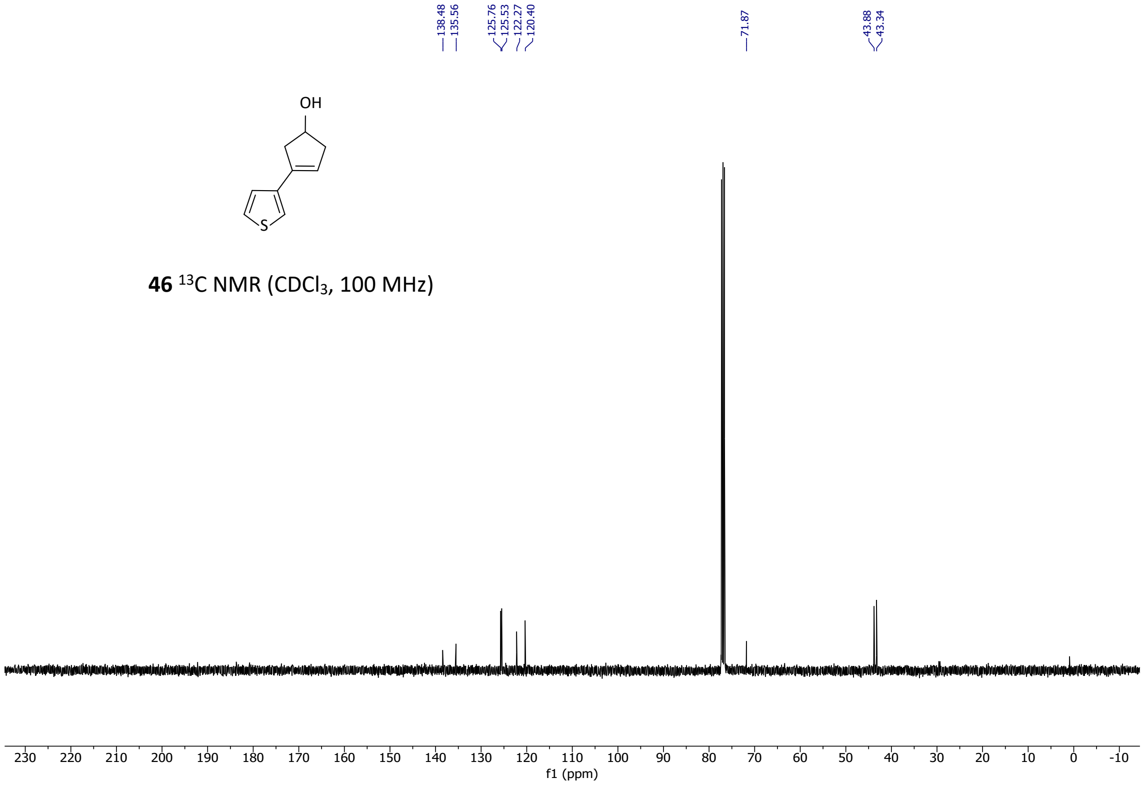


**46** $^{13}$C NMR ($CDCl_3$, 100 MHz)

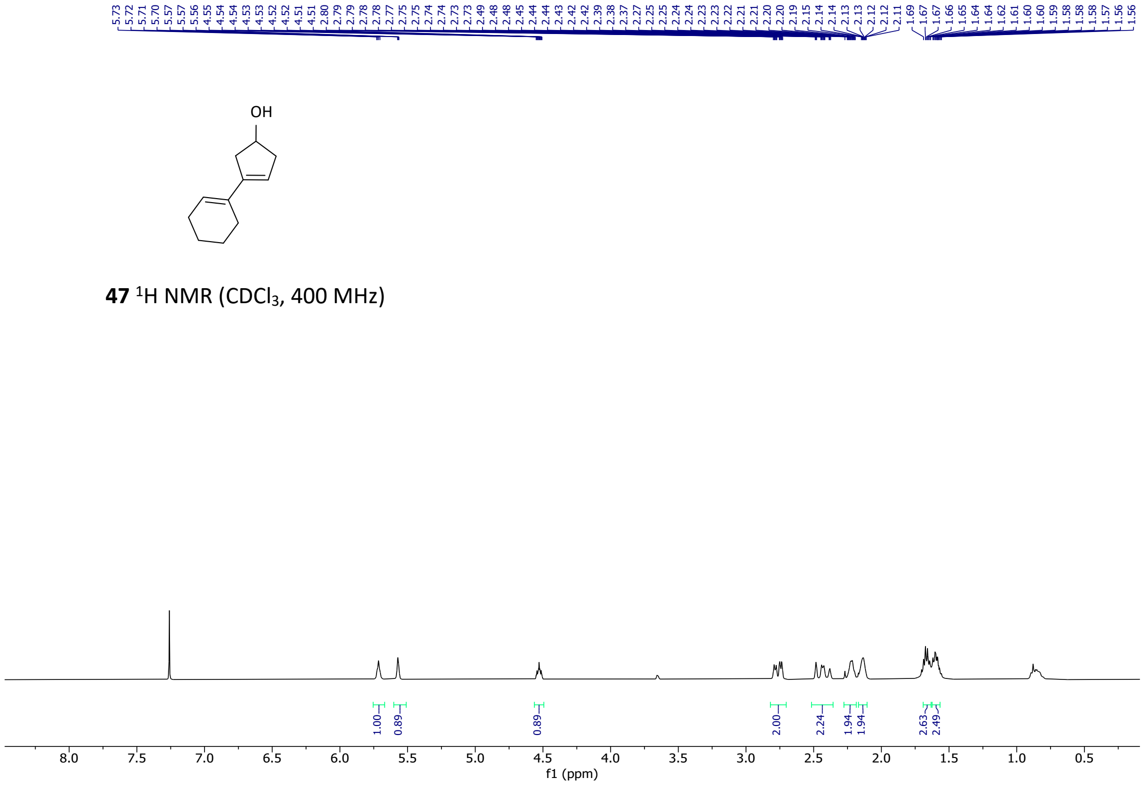


**47** $^{1}$H NMR ($CDCl_3$, 400 MHz)

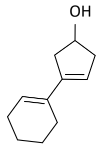

**47** $^{13}C$ NMR ($CDCl_3$, 100 MHz)

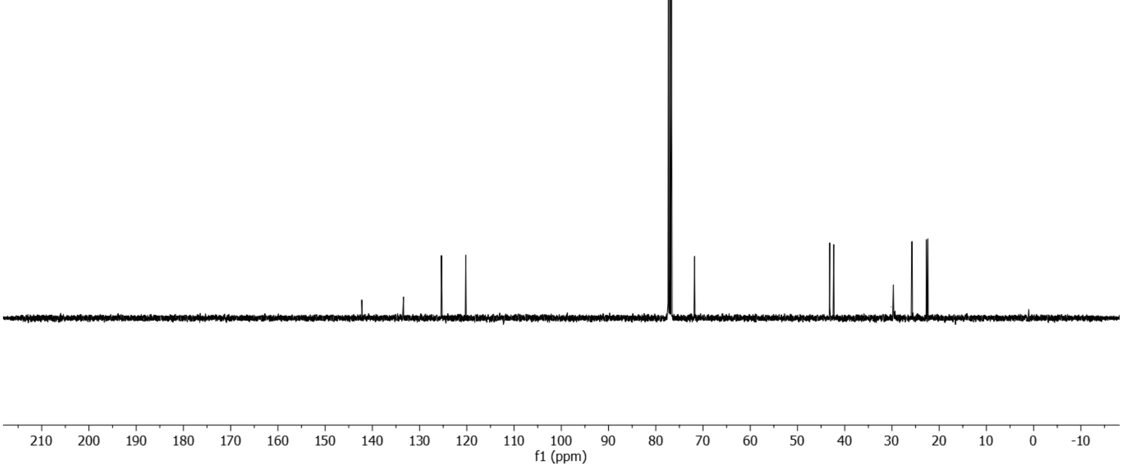


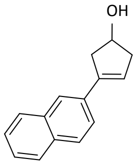

**48** $^{1}H$ NMR ($CDCl_3$, 400 MHz)

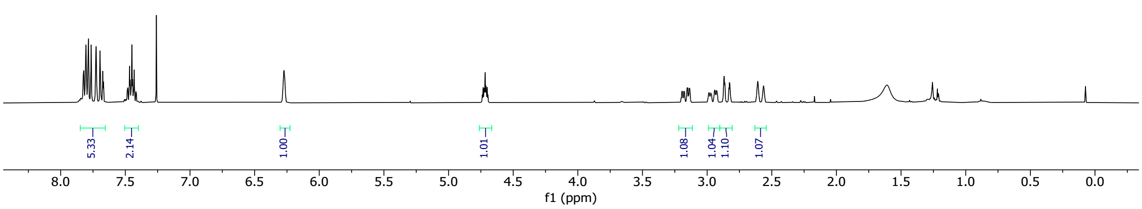

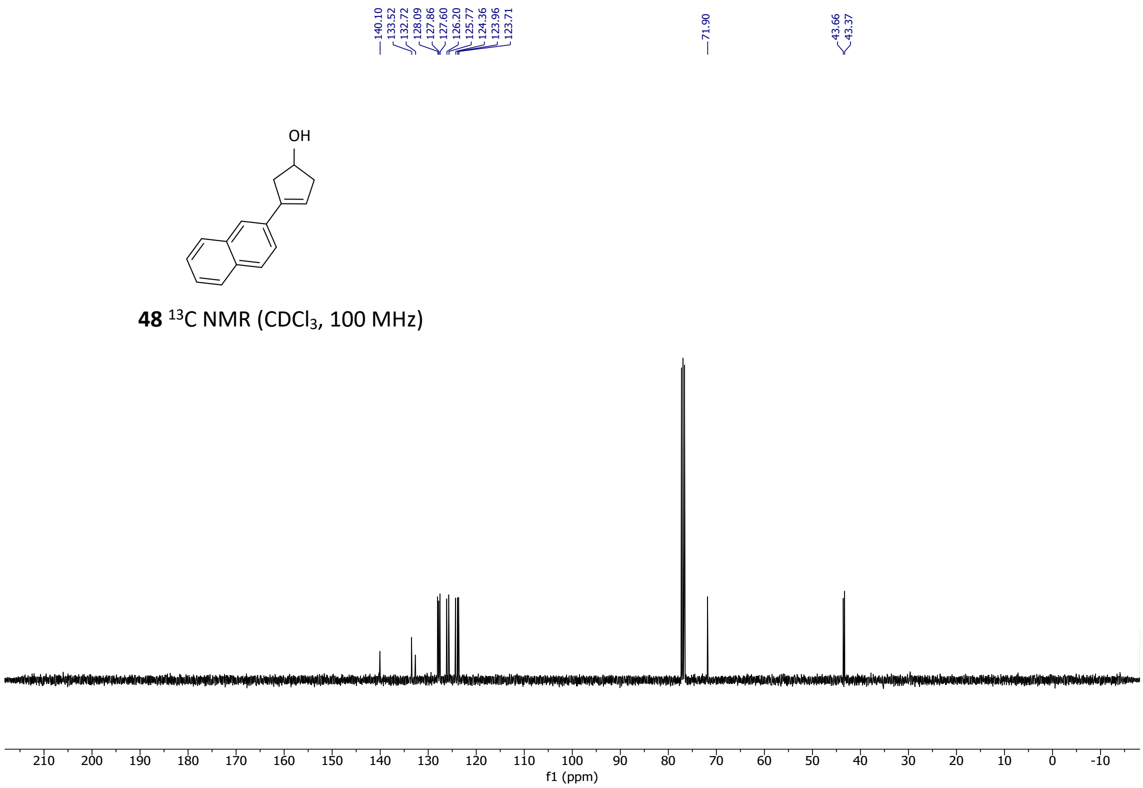


**48** $^{13}$C NMR ($CDCl_3$, 100 MHz)

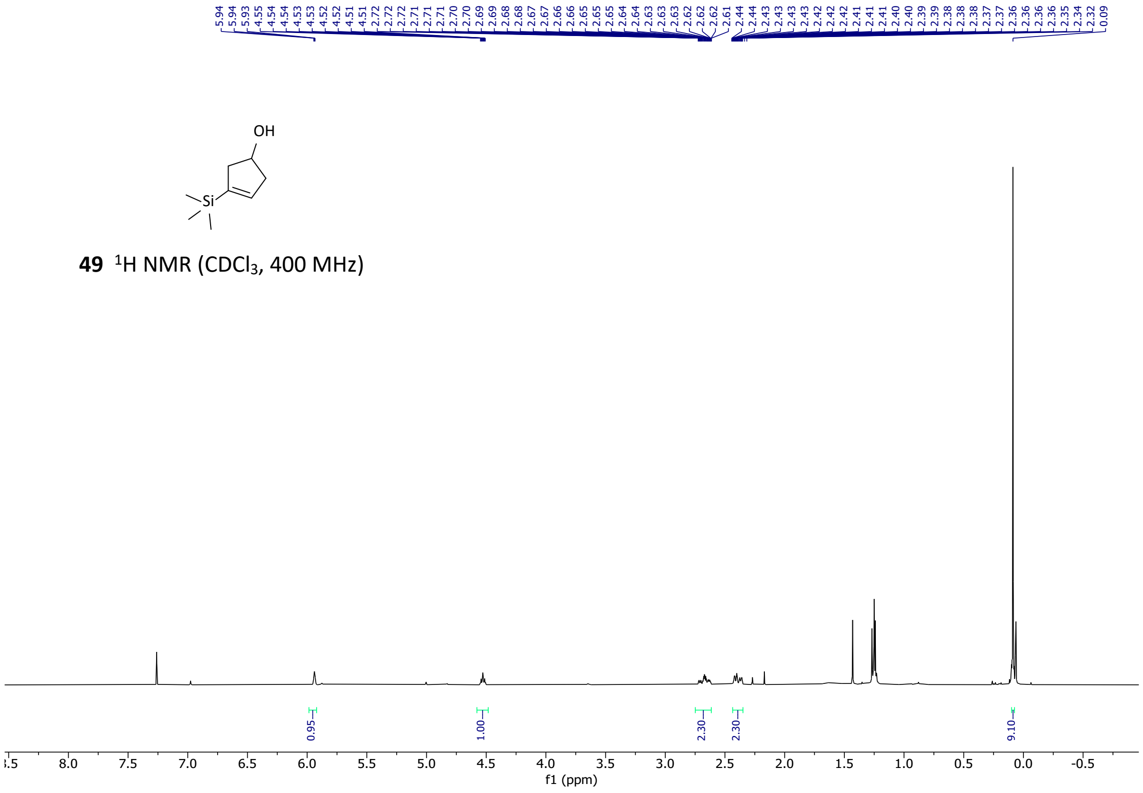


**49** $^{1}$H NMR ($CDCl_3$, 400 MHz)

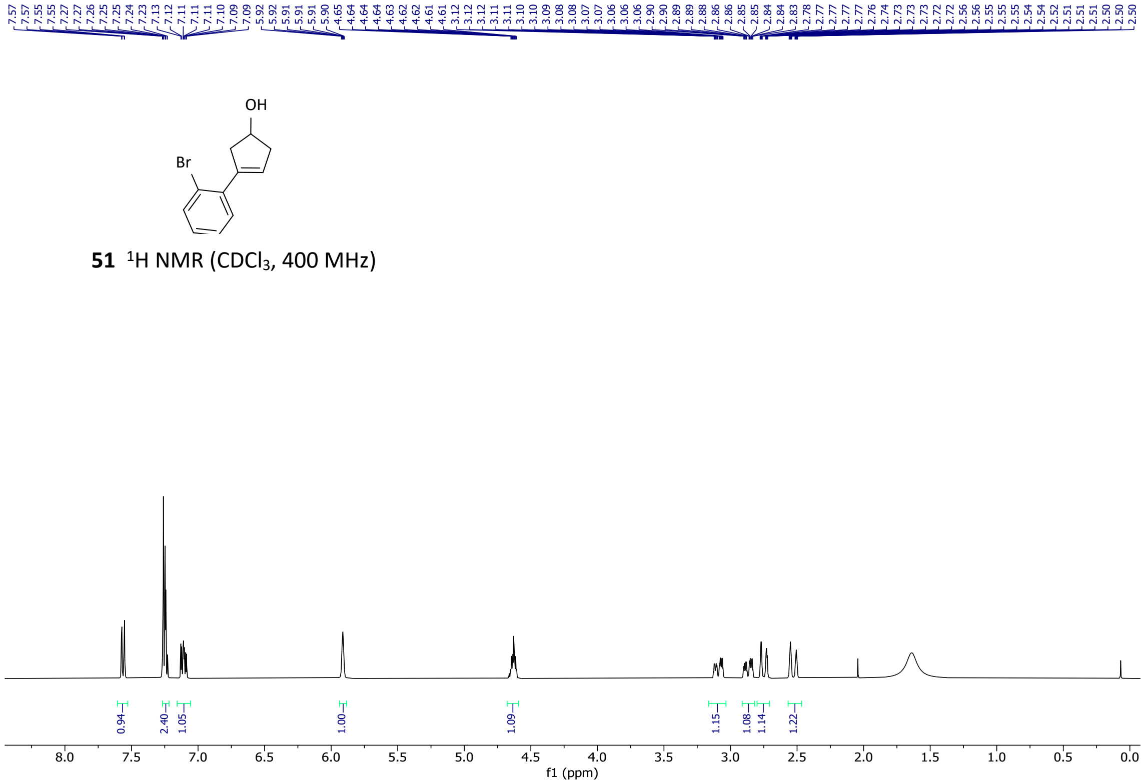
7.57 7.57 7.55 7.55 7.27 7.27 7.26 7.25 7.25 7.24 7.23 7.13 7.12 7.11 7.11 7.11 7.10 7.09 7.09 5.92 5.92 5.91 5.91 5.91 5.90 4.65 4.64 4.64 4.63 4.62 4.62 4.61 4.61 3.12 3.12 3.12 3.11 3.11 3.10 3.10 3.09 3.08 3.08 3.07 3.06 3.06 3.06 2.90 2.90 2.89 2.89 2.88 2.86 2.86 2.85 2.85 2.84 2.84 2.83 2.78 2.77 2.77 2.77 2.77 2.76 2.74 2.73 2.73 2.73 2.72 2.72 2.56 2.56 2.55 2.55 2.55 2.54 2.54 2.52 2.51 2.51 2.51 2.50 2.50 2.50
OH
Br
**51** $^{1}$H NMR ($CDCl_3$, 400 MHz)
0.94
2.40
1.05
1.00
1.09
1.15
1.08
1.14
1.22
8.0 7.5 7.0 6.5 6.0 5.5 5.0 4.5 4.0 3.5 3.0 2.5 2.0 1.5 1.0 0.5 0.0
f1 (ppm)

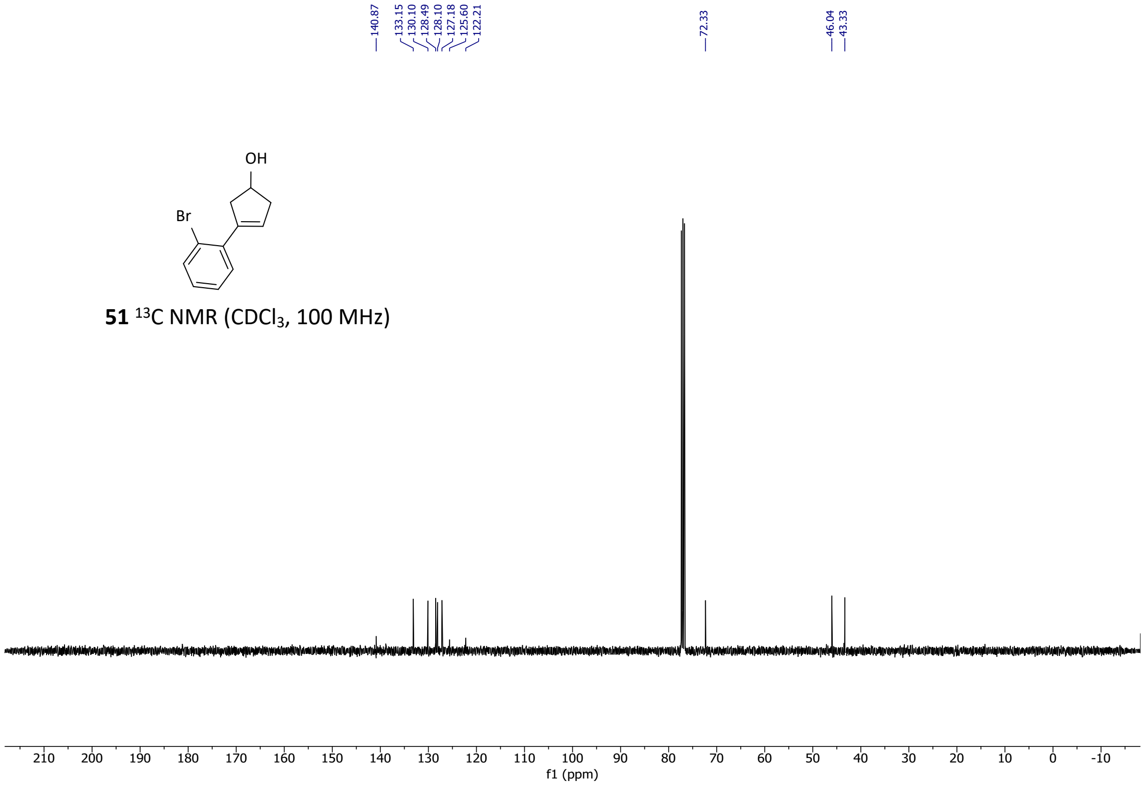
140.87
133.15
130.10
128.49
128.10
127.18
125.60
122.21
72.33
46.04
43.33
OH
Br
**51** $^{13}$C NMR ($CDCl_3$, 100 MHz)
210 200 190 180 170 160 150 140 130 120 110 100 90 80 70 60 50 40 30 20 10 0 -10
f1 (ppm)

## $^{1}$H, $^{13}$C Spectra for bicycle products

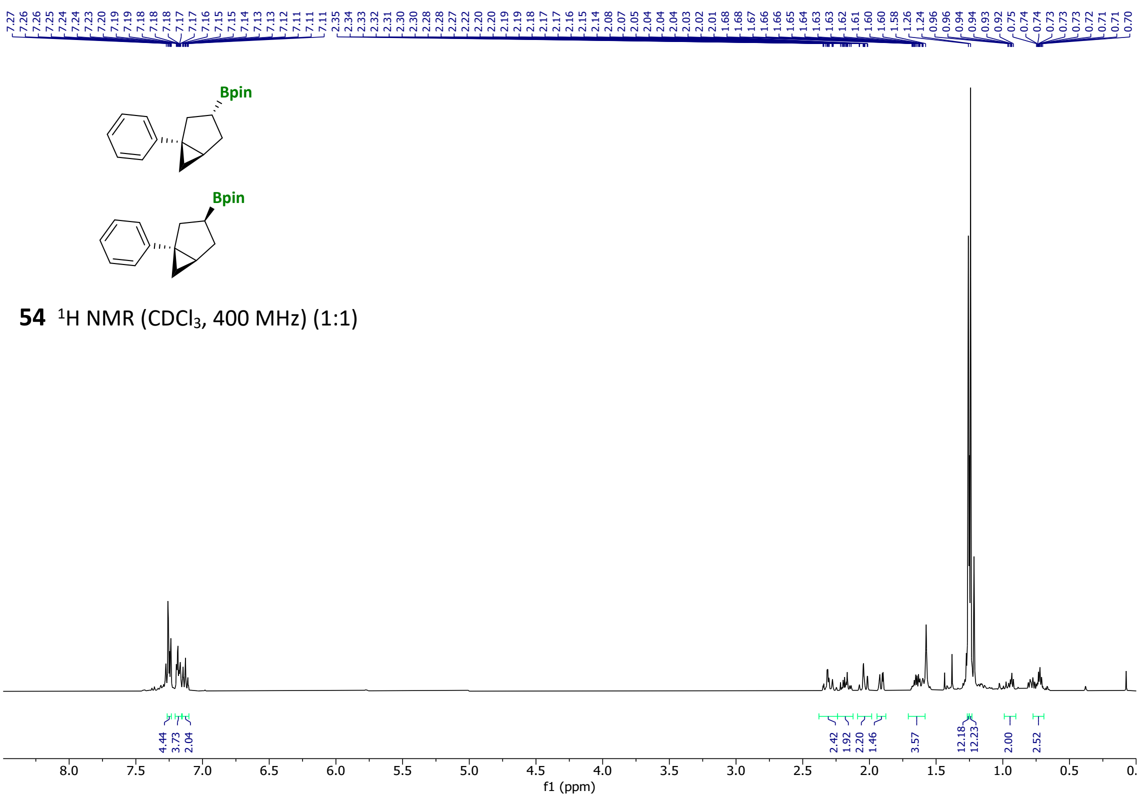


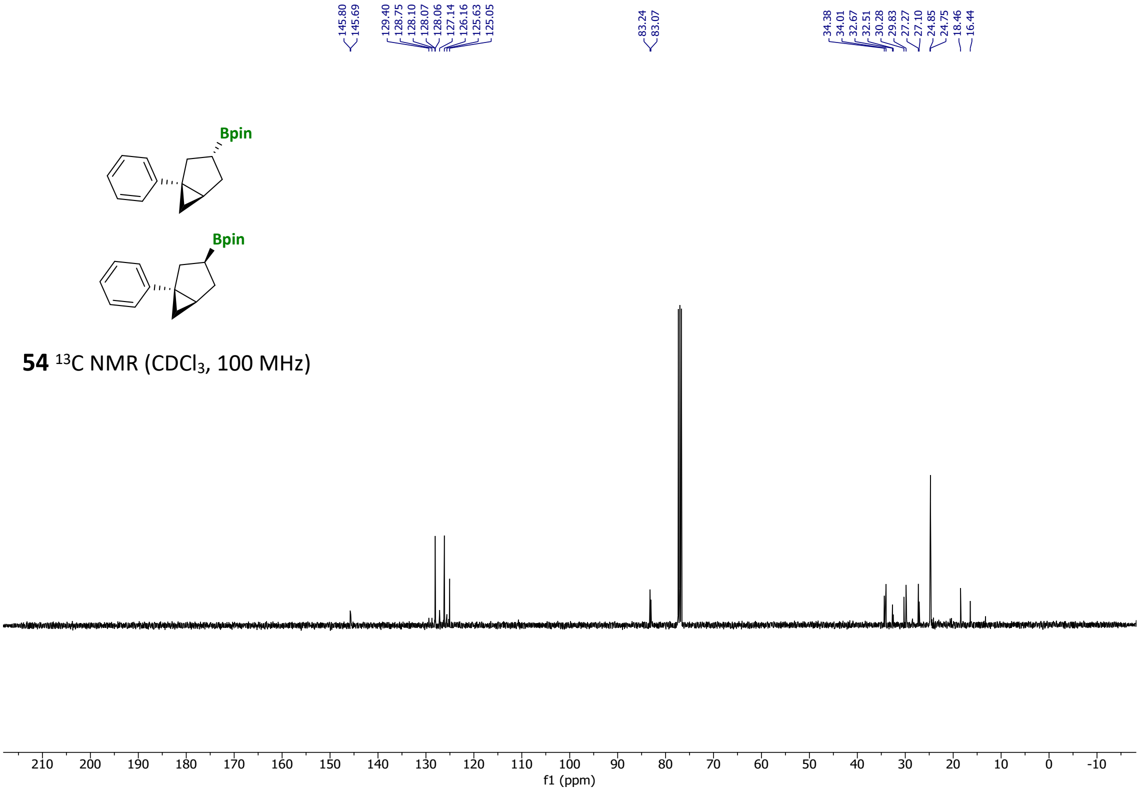

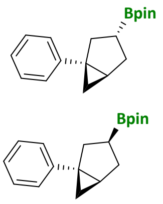

**54** $^{11}$B NMR (CDCl$_3$, 128.3 MHz)

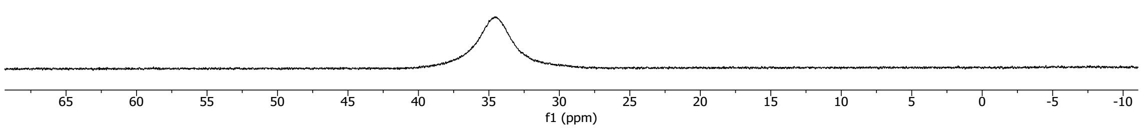


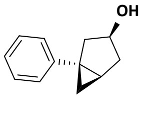

**55-*syn*** $^{1}$H NMR (CDCl$_3$, 400 MHz)

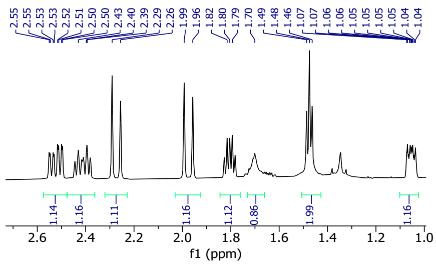


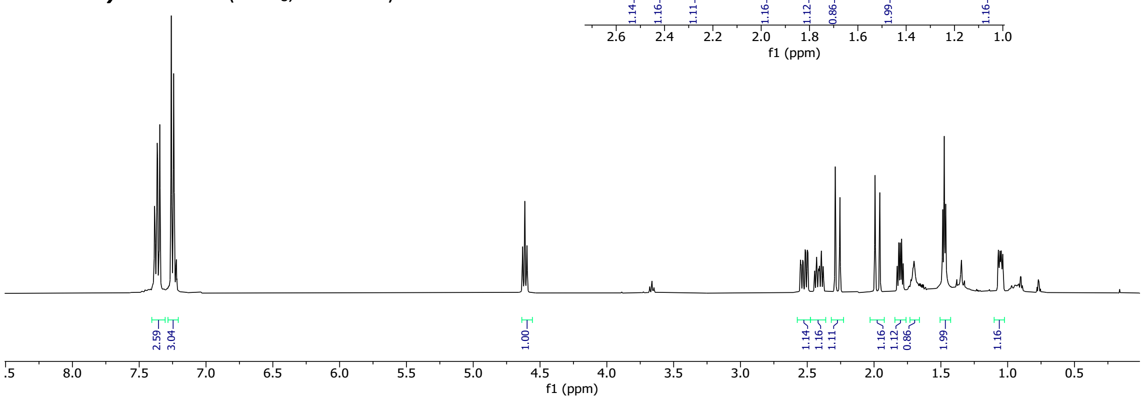

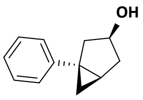


**55-*syn*-**$^{13}$C NMR (CDCl$_3$, 100

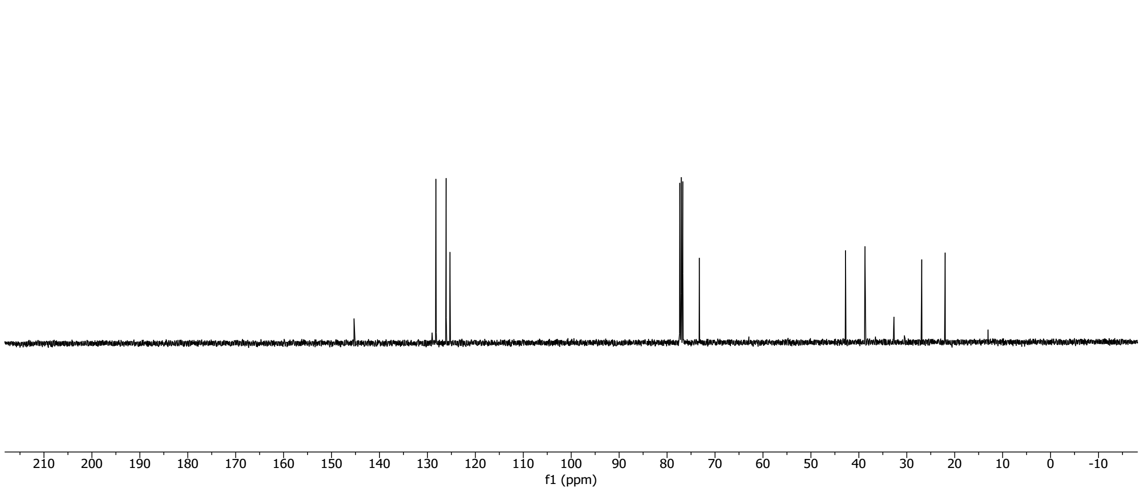


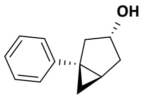


**55-*anti*** $^{1}$H NMR (CDCl$_3$, 400 MHz)

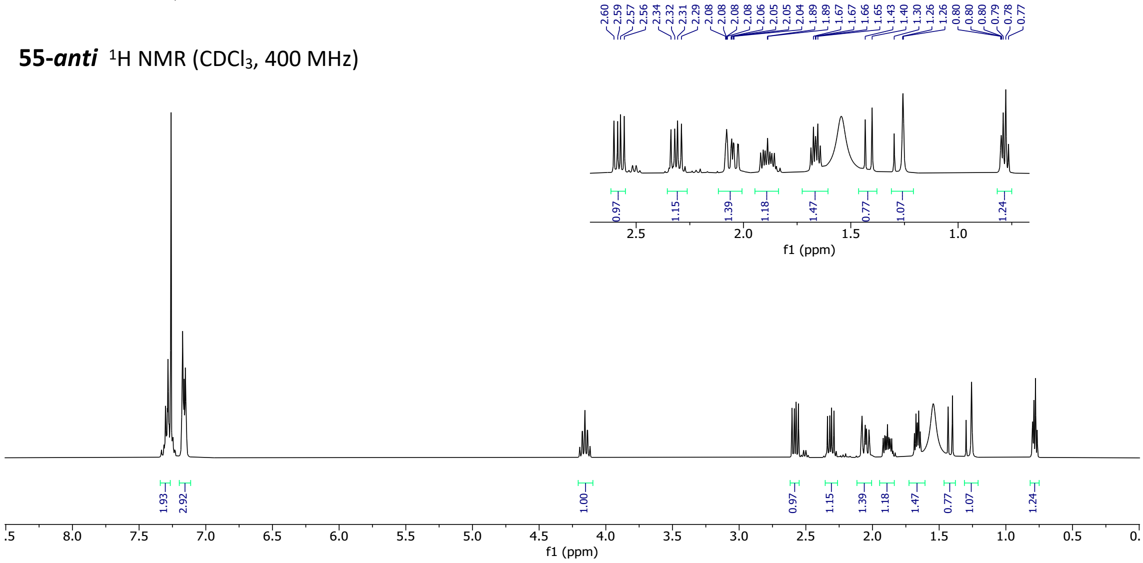

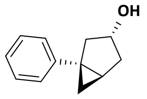


**55-*anti*-**$^{13}$C NMR ($CDCl_3$, 100 MHz)

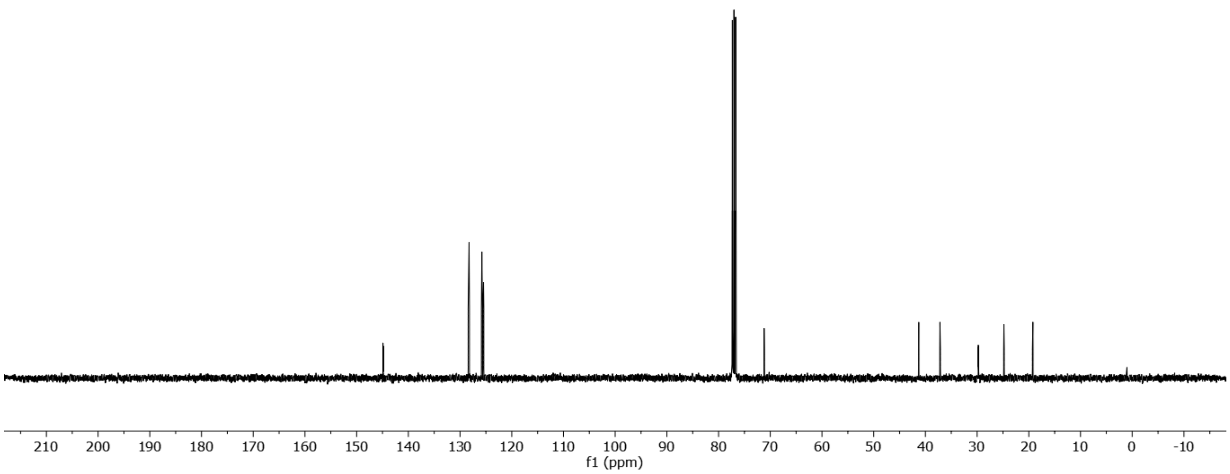


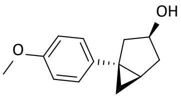


**56** $^{1}$H NMR ($CDCl_3$, 400 MHz)

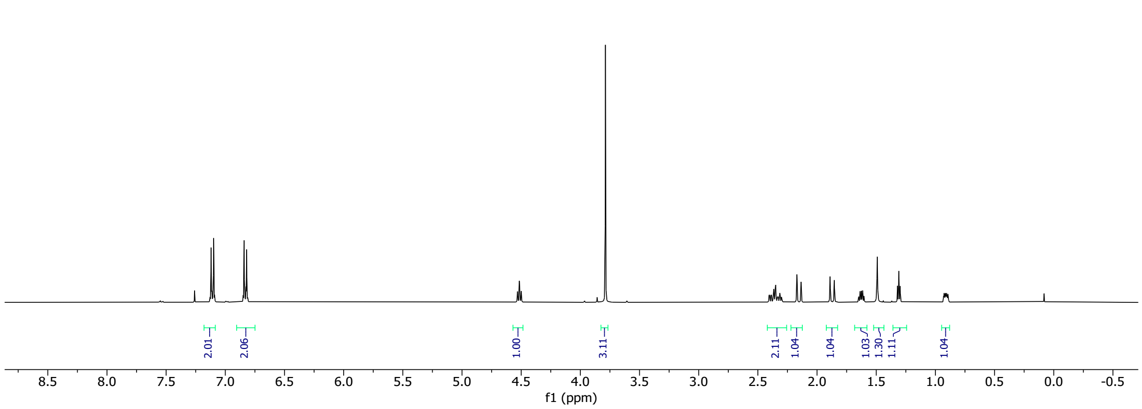

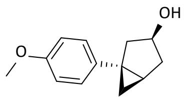

**56-**$^{13}$C NMR ($CDCl_3$, 100 MHz)

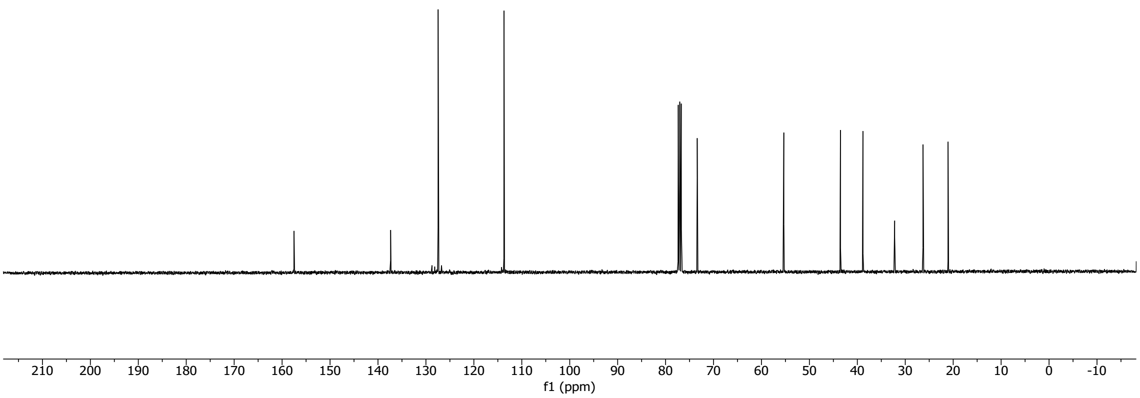


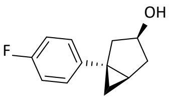

**57** $^{1}$H NMR ($CDCl_3$, 400 MHz)

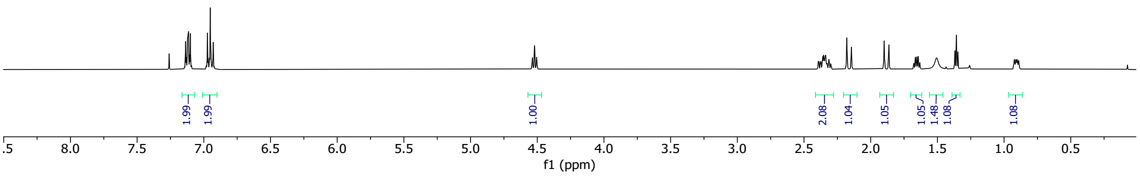

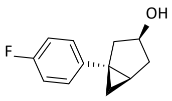

**57** $^{13}$C NMR ($CDCl_3$, 100 MHz)

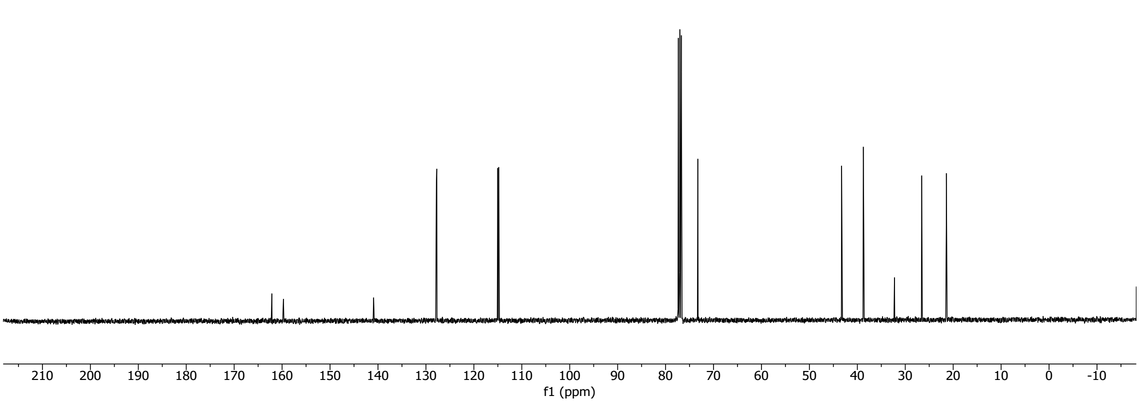


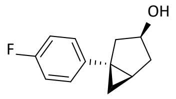

**57** $^{19}$F NMR ($CDCl_3$, 377 MHz)

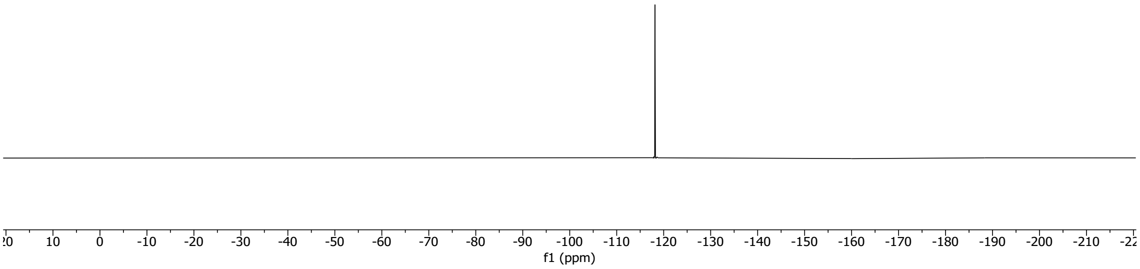

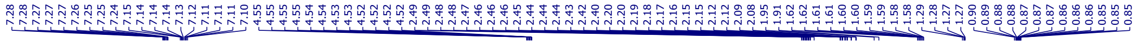


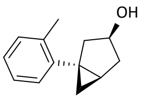


**58** ¹H NMR ($CDCl_3$, 400 MHz)

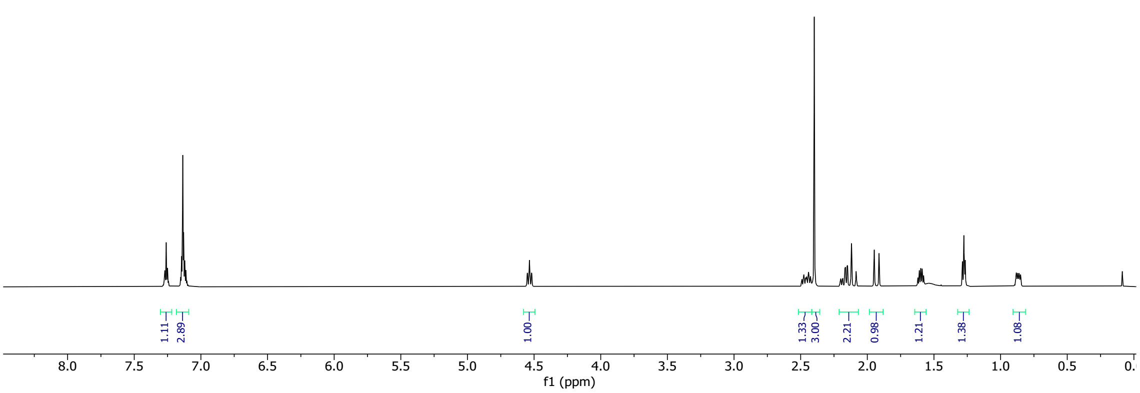


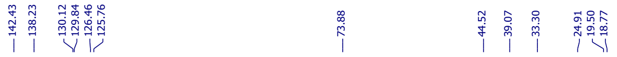


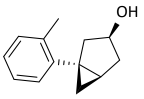


**58** ¹³C NMR ($CDCl_3$, 100 MHz)

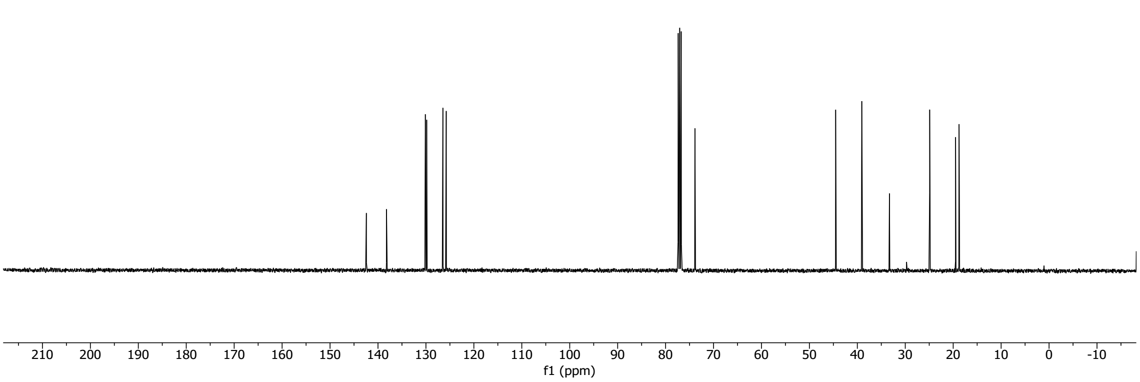

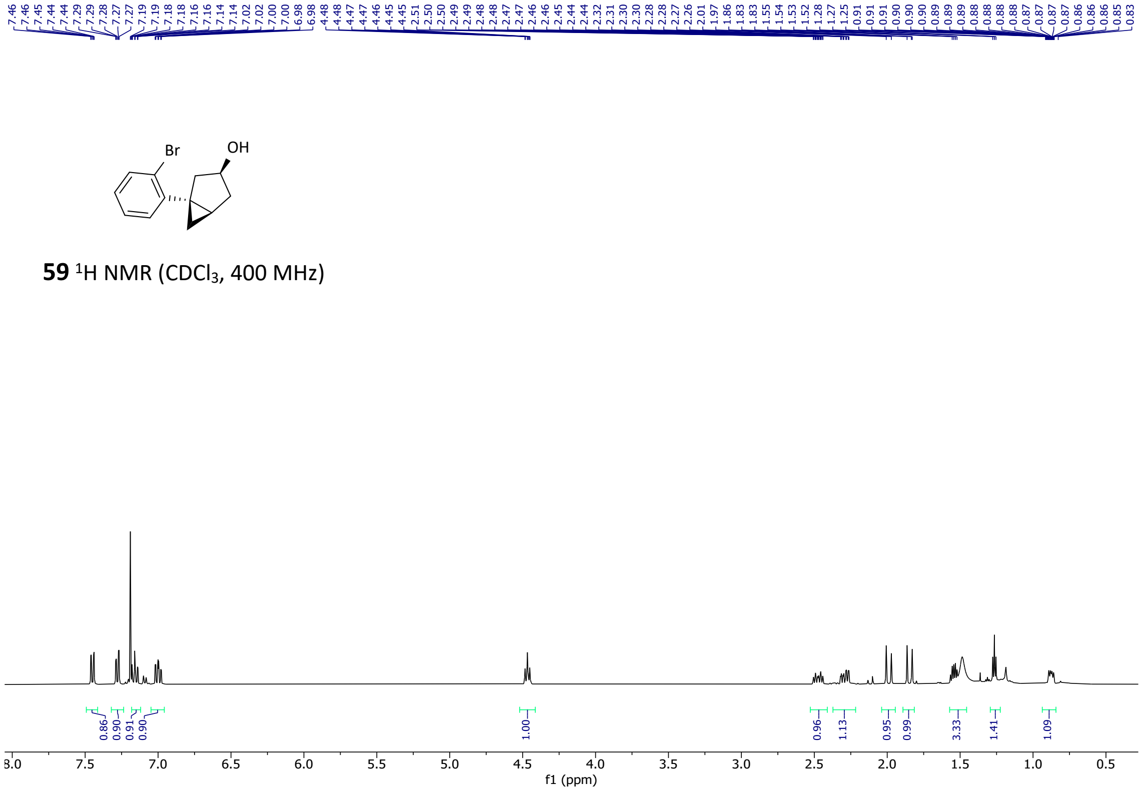


**59** $^{1}H$ NMR ($CDCl_3$, 400 MHz)

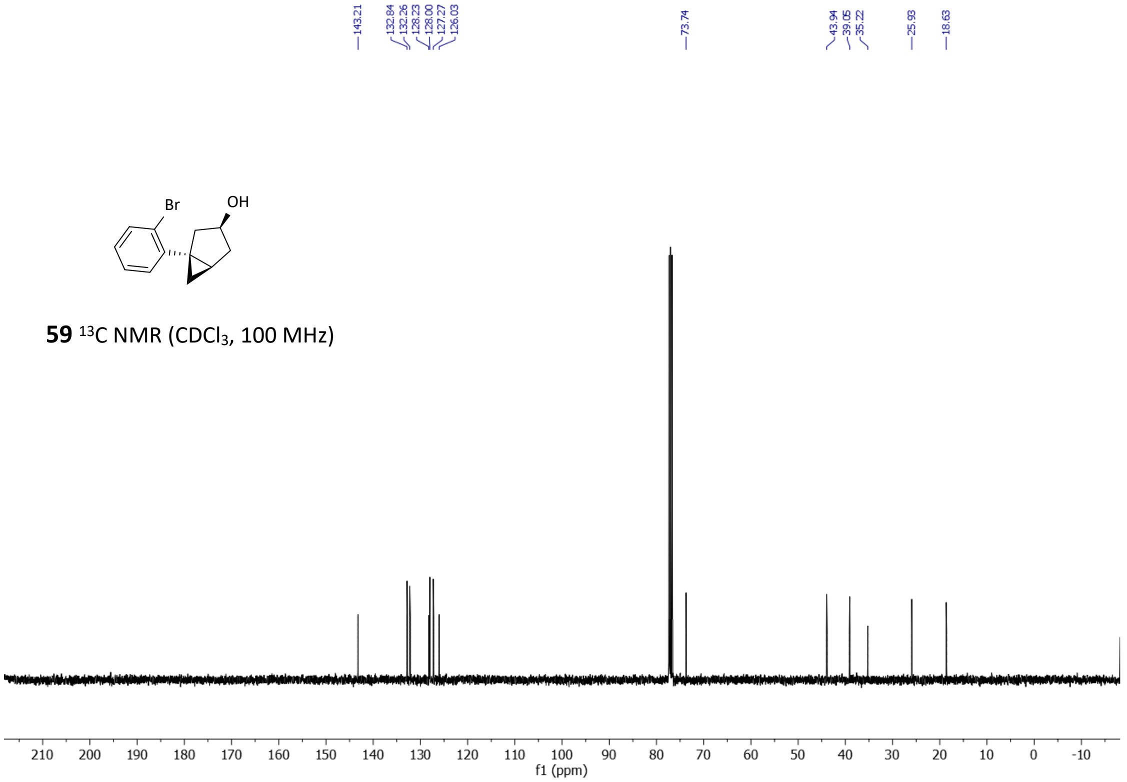


**59** $^{13}C$ NMR ($CDCl_3$, 100 MHz)

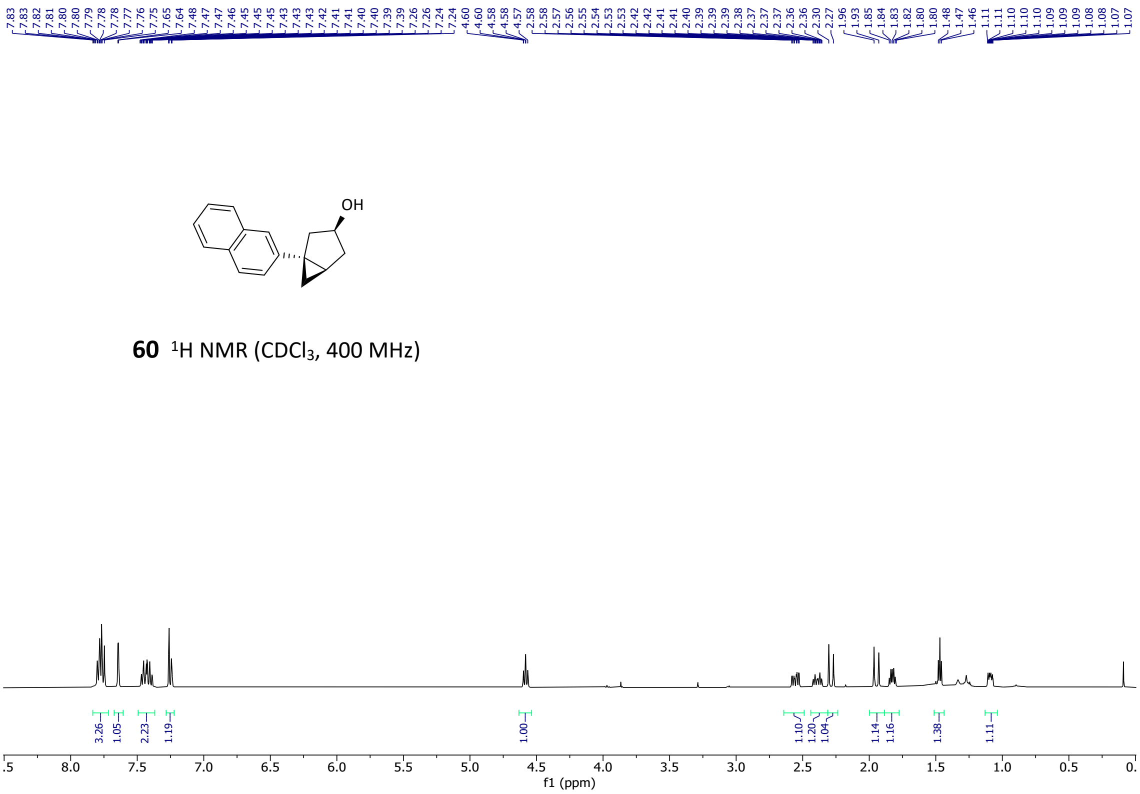


**60** ¹H NMR ($CDCl_3$, 400 MHz)

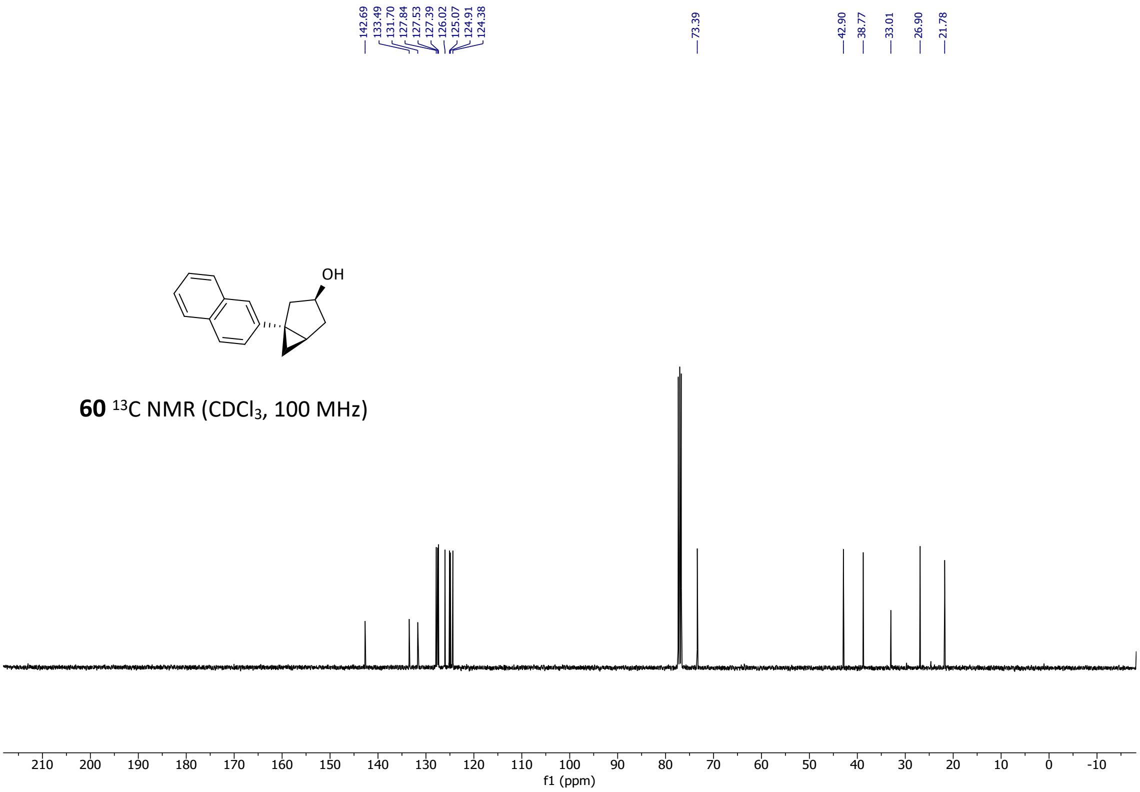


**60** ¹³C NMR ($CDCl_3$, 100 MHz)

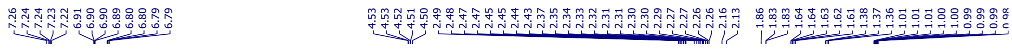


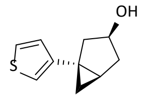


**61** $^{1}$H NMR ($CDCl_3$, 400 MHz)

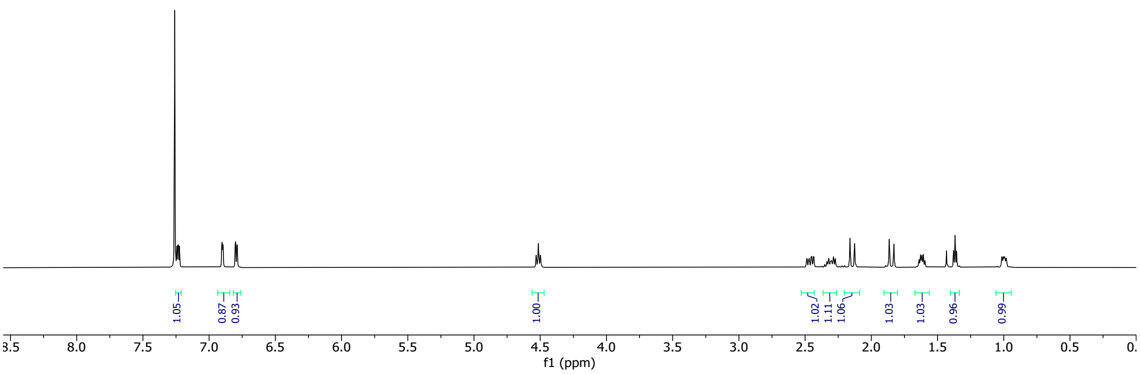


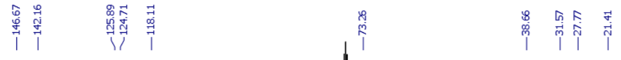


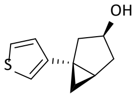


**61** $^{13}$C NMR ($CDCl_3$, 100 MHz)

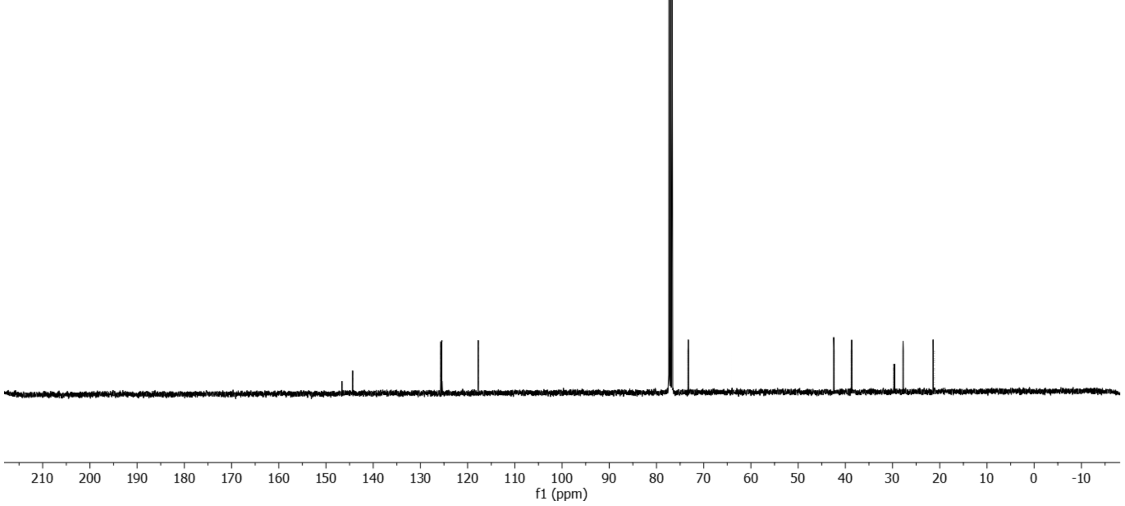

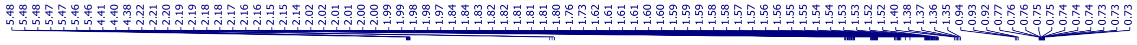


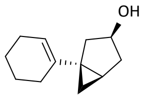


**62** $^{1}$H NMR ($CDCl_3$, 400 MHz)

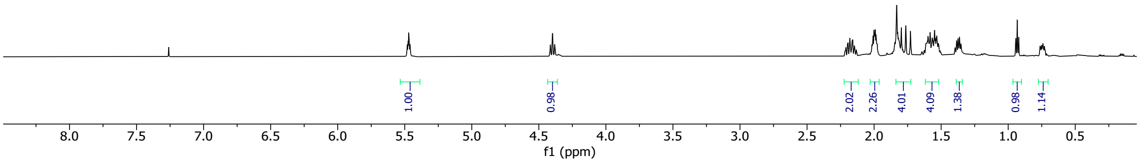


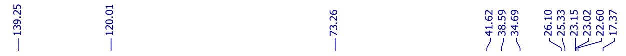


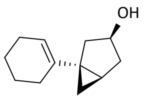


**62** $^{13}$C NMR ($CDCl_3$, 100 MHz)

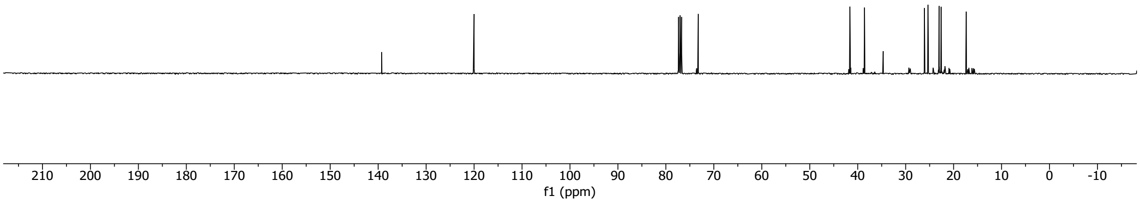

## - Computational Details

Geometry optimizations, transition state searches, and energy evaluations were performed with Gaussian 16 package.[4] The quantum mechanics calculations were performed within the framework of Density Functional Theory (DFT)[5] by using the ωB97X-D functional.[6] For Cu, P, K and I, we employed effective core potentials (ECPs) with double-ζ valence basis set (LANL2DZ),[7] supplemented with polarized shells with the following exponents: Cu (f = 3.525), P (d = 0.387), K (d = 1.000) and I (d = 0.289).[8] For all other atoms, the 6-31G(d) basis set was used.[9] Solvent effects of THF were included using the implicit solvation model SMD.[10] Free energies were computed at a concentration of 1 M and a temperature of 298.15 K.

To quantify the steric hindrance of substrate substituents, we used the distance-weighed volume parameter ($V_W$),[11] which measures the steric bulkiness of the molecular environment and its impact on the copper center. The descriptor quantifies the bulk produced by substrate substituents considering three parameters: 1) The number of atoms, excluding those forming the cyclic structures, 2) the size of the atom (*r* = van der Waals radii in Å), and 3) the distance (d) from the atom to the copper center (in Å). The factor $r^3$ is divided by d for each atom and the sum is extended to all the atoms in the given fragment, as given by the following equation:

$$V_W = \sum_{i=1}^{N} \frac{r^3}{d_i}$$

We have evaluated $V_W$ in transition states **TS2-A** and **TS2**, involving 4- and 5-membered ring structures, respectively. The Cu centre was placed at the origin. Then, we considered the number of atoms on the substituents of substrates **A** and **1a**, excluding those on the ring skeleton, and the distance from those atoms to the Cu centre. In both structures, **TS2-A** and **TS2**, we obtained the same value of $V_W$. This indicates that the ligand-substrate interactions have a minor effect on the free-energy barrier differences, and they can be attributed to ring strain with two opposite effects.

## Cartesian coordinates in Å, electronic energies and Gibbs free energies in a.u. for the most representative structures.

### I1

ΔG = -2021.854776 a.u.

| | | | |
|---|---|---|---|
| C | 2.261161 | 1.412400 | -0.818369 |
| C | 1.237468 | 2.355666 | -0.715953 |
| C | 1.325772 | 3.651911 | -1.217527 |
| C | 2.501154 | 3.993666 | -1.886526 |
| C | 3.539267 | 3.074119 | -2.020070 |
| C | 3.427912 | 1.794809 | -1.482567 |
| H | 2.618727 | 4.987313 | -2.305284 |
| H | 4.446033 | 3.360030 | -2.543211 |
| H | 4.245240 | 1.089466 | -1.591090 |

| | | | |
|---|---|---|---|
| C | 0.163719 | 4.601339 | -0.926424 |
| C | -1.122363 | 3.778611 | -1.009524 |
| C | -1.083770 | 2.464166 | -0.544061 |
| C | -2.348182 | 4.256275 | -1.469452 |
| C | -2.194137 | 1.617303 | -0.528646 |
| C | -3.482515 | 3.448365 | -1.446310 |
| H | -2.428417 | 5.270145 | -1.845966 |
| C | -3.409044 | 2.140305 | -0.980684 |
| H | -4.430194 | 3.839983 | -1.801202 |
| H | -4.297060 | 1.517643 | -0.988047 |
| O | 0.095563 | 1.947538 | -0.069230 |
| P | 1.909079 | -0.257303 | -0.137340 |
| P | -1.956361 | -0.152513 | -0.066202 |
| Cu | -0.107062 | -1.277978 | -0.877077 |
| C | 1.979640 | 0.006285 | 1.683906 |
| C | 1.585521 | -1.065675 | 2.492868 |
| C | 2.392380 | 1.197910 | 2.284092 |
| C | 1.622123 | -0.953651 | 3.878217 |
| H | 1.250619 | -1.993404 | 2.035274 |
| C | 2.413753 | 1.313498 | 3.672691 |
| H | 2.700635 | 2.040698 | 1.672360 |
| C | 2.033397 | 0.238647 | 4.471194 |
| H | 1.314710 | -1.792634 | 4.494459 |
| H | 2.733243 | 2.245609 | 4.129178 |
| H | 2.051256 | 0.330395 | 5.552962 |
| C | -1.893337 | -0.170891 | 1.772561 |
| C | -2.017536 | -1.414113 | 2.406980 |
| C | -1.662450 | 0.968657 | 2.544416 |
| C | -1.937523 | -1.508693 | 3.791623 |
| H | -2.192080 | -2.310929 | 1.817006 |
| C | -1.567287 | 0.868300 | 3.930925 |
| H | -1.562945 | 1.941675 | 2.074530 |
| C | -1.710928 | -0.366024 | 4.557109 |
| H | -2.046609 | -2.476138 | 4.272194 |
| H | -1.381892 | 1.760766 | 4.520569 |
| H | -1.639296 | -0.440008 | 5.637907 |
| C | -3.597897 | -0.885120 | -0.444347 |
| C | -3.743117 | -1.539514 | -1.671420 |
| C | -4.694384 | -0.796110 | 0.420637 |
| C | -4.968366 | -2.093621 | -2.033321 |
| H | -2.889583 | -1.627413 | -2.338492 |
| C | -5.915974 | -1.357194 | 0.060762 |
| H | -4.593804 | -0.288919 | 1.375655 |
| C | -6.054241 | -2.005072 | -1.166172 |
| H | -5.069235 | -2.603069 | -2.986550 |
| H | -6.761377 | -1.288069 | 0.738491 |
| H | -7.007907 | -2.444186 | -1.443116 |
| C | 3.432180 | -1.222201 | -0.476882 |
| C | 3.324166 | -2.291366 | -1.373652 |
| C | 4.661482 | -0.943161 | 0.132836 |
| C | 4.450152 | -3.060358 | -1.669085 |
| H | 2.346473 | -2.539519 | -1.797065 |
| C | 5.779177 | -1.714648 | -0.166918 |
| H | 4.742490 | -0.124228 | 0.842988 |
| C | 5.674055 | -2.771955 | -1.071723 |
| H | 4.365933 | -3.892350 | -2.362355 |
| H | 6.731377 | -1.495027 | 0.306628 |
| H | 6.547557 | -3.374669 | -1.302743 |
| O | 0.215572 | -2.898454 | -1.887418 |
| C | -0.066798 | -4.136569 | -1.336645 |

| | | | |
|---|---|---|---|
| C | 0.654280 | -5.219788 | -2.161748 |
| H | 0.458930 | -6.233626 | -1.790204 |
| H | 0.330112 | -5.168005 | -3.207172 |
| H | 1.736719 | -5.048376 | -2.135657 |
| C | -1.582125 | -4.419766 | -1.353644 |
| H | -2.108158 | -3.666487 | -0.755198 |
| H | -1.964810 | -4.357599 | -2.378882 |
| H | -1.831452 | -5.410725 | -0.952540 |
| C | 0.426292 | -4.224262 | 0.125018 |
| H | 0.262998 | -5.213015 | 0.572848 |
| H | 1.497671 | -3.997700 | 0.172058 |
| H | -0.104908 | -3.484011 | 0.739904 |
| C | 0.143992 | 5.797986 | -1.879658 |
| H | -0.678142 | 6.474400 | -1.631790 |
| H | 1.065123 | 6.378754 | -1.784241 |
| H | 0.035598 | 5.484190 | -2.922278 |
| C | 0.317019 | 5.116166 | 0.525042 |
| H | 1.246753 | 5.685373 | 0.625902 |
| H | -0.522582 | 5.769426 | 0.783678 |
| H | 0.342644 | 4.290363 | 1.242738 |

**I2**

ΔG = -2199.926537 a.u.

| | | | |
|---|---|---|---|
| C | -0.863089 | -2.380668 | 1.021249 |
| C | -1.680605 | -1.453601 | 1.668338 |
| C | -2.247688 | -1.664814 | 2.922832 |
| C | -1.955645 | -2.873182 | 3.555207 |
| C | -1.147846 | -3.825438 | 2.937117 |
| C | -0.609018 | -3.587098 | 1.676026 |
| H | -2.364627 | -3.083353 | 4.537596 |
| H | -0.937917 | -4.762047 | 3.443540 |
| H | 0.022546 | -4.334183 | 1.206215 |
| C | -3.189729 | -0.588881 | 3.462770 |
| C | -2.623567 | 0.765069 | 3.032311 |
| C | -2.028052 | 0.849373 | 1.774155 |
| C | -2.700125 | 1.939338 | 3.779128 |
| C | -1.518944 | 2.032263 | 1.233625 |
| C | -2.211044 | 3.140302 | 3.270475 |
| H | -3.147305 | 1.926104 | 4.767109 |
| C | -1.626306 | 3.189666 | 2.009412 |
| H | -2.281564 | 4.045151 | 3.865380 |
| H | -1.234754 | 4.129920 | 1.636915 |
| O | -1.920874 | -0.278974 | 0.998289 |
| P | -0.123776 | -1.868826 | -0.584175 |
| P | -0.603459 | 1.928062 | -0.365989 |
| Cu | 1.048288 | 0.202596 | -0.302933 |
| B | 3.047858 | 0.324321 | 0.153292 |
| O | 3.695243 | -0.433073 | 1.140251 |
| O | 4.001020 | 1.130855 | -0.481447 |
| C | 5.120290 | -0.294881 | 0.993375 |
| C | 5.243935 | 1.067301 | 0.242663 |
| C | -1.530768 | -1.987081 | -1.760919 |
| C | -1.357346 | -1.397748 | -3.018173 |
| C | -2.741348 | -2.617898 | -1.463081 |
| C | -2.370012 | -1.457018 | -3.969308 |
| H | -0.427165 | -0.884764 | -3.250719 |
| C | -3.761624 | -2.660921 | -2.410838 |
| H | -2.891513 | -3.077977 | -0.490754 |
| C | -3.576047 | -2.085782 | -3.665163 |
| H | -2.224655 | -0.995608 | -4.940847 |

| | | | |
|---|---|---|---|
| H | -4.700849 | -3.149125 | -2.168420 |
| H | -4.371981 | -2.120560 | -4.402984 |
| C | -1.916862 | 1.871159 | -1.660605 |
| C | -1.552634 | 2.209660 | -2.970815 |
| C | -3.219594 | 1.433304 | -1.412628 |
| C | -2.480747 | 2.136324 | -4.003885 |
| H | -0.540969 | 2.545844 | -3.183971 |
| C | -4.144447 | 1.349871 | -2.451235 |
| H | -3.528118 | 1.163663 | -0.408248 |
| C | -3.781171 | 1.706470 | -3.746352 |
| H | -2.186122 | 2.412913 | -5.011770 |
| H | -5.153133 | 1.006607 | -2.242546 |
| H | -4.505082 | 1.643739 | -4.553131 |
| C | 0.072878 | 3.626612 | -0.577165 |
| C | 1.449623 | 3.814520 | -0.422956 |
| C | -0.740881 | 4.726333 | -0.881045 |
| C | 2.002641 | 5.086980 | -0.560119 |
| H | 2.091241 | 2.962307 | -0.210626 |
| C | -0.185962 | 5.993969 | -1.017851 |
| H | -1.810639 | 4.588760 | -1.011475 |
| C | 1.187697 | 6.175618 | -0.855479 |
| H | 3.073308 | 5.223538 | -0.441852 |
| H | -0.824017 | 6.840485 | -1.252833 |
| H | 1.620377 | 7.165588 | -0.965244 |
| C | 0.932877 | -3.312122 | -1.017179 |
| C | 2.265680 | -3.286874 | -0.587397 |
| C | 0.464535 | -4.415742 | -1.736890 |
| C | 3.112736 | -4.354986 | -0.870283 |
| H | 2.639673 | -2.429266 | -0.031973 |
| C | 1.317647 | -5.478029 | -2.025391 |
| H | -0.566795 | -4.447125 | -2.075776 |
| C | 2.641687 | -5.449693 | -1.592081 |
| H | 4.144977 | -4.325891 | -0.533845 |
| H | 0.946685 | -6.328935 | -2.588858 |
| H | 3.305493 | -6.278451 | -1.819564 |
| C | -4.569117 | -0.774477 | 2.786255 |
| H | -4.990378 | -1.749314 | 3.052570 |
| H | -5.260055 | 0.007311 | 3.117890 |
| H | -4.490768 | -0.723310 | 1.695924 |
| C | -3.363234 | -0.684099 | 4.980011 |
| H | -2.411659 | -0.558295 | 5.505308 |
| H | -4.061637 | 0.077732 | 5.336335 |
| H | -3.789255 | -1.651913 | 5.257462 |
| C | 5.602428 | -1.487919 | 0.162865 |
| H | 6.690777 | -1.492243 | 0.046516 |
| H | 5.310534 | -2.411151 | 0.673066 |
| H | 5.142774 | -1.488554 | -0.830121 |
| C | 6.403383 | 1.152463 | -0.740821 |
| H | 6.411855 | 2.136192 | -1.220523 |
| H | 7.360551 | 1.020092 | -0.224786 |
| H | 6.322531 | 0.395954 | -1.524378 |
| C | 5.274145 | 2.272839 | 1.186049 |
| H | 6.217150 | 2.336278 | 1.738119 |
| H | 5.162463 | 3.187075 | 0.595205 |
| H | 4.449178 | 2.231122 | 1.903556 |
| C | 5.773459 | -0.328584 | 2.368562 |
| H | 5.633626 | -1.315074 | 2.821421 |
| H | 6.850116 | -0.141587 | 2.291658 |
| H | 5.337753 | 0.415075 | 3.039186 |

**I3**

ΔG = -3036.434165 a.u.

| | | | |
|---|---|---|---|
| Cu | -0.324518 | -0.110689 | -0.916480 |
| C | 1.845232 | -0.287268 | -1.965479 |
| H | 1.477291 | -0.325653 | -2.985568 |
| H | 2.216588 | -1.210443 | -1.527691 |
| C | 1.957471 | 0.884626 | -1.310703 |
| P | -0.206633 | -1.844843 | 0.812919 |
| P | -0.969918 | 1.976678 | -0.021359 |
| B | -1.893648 | -0.707257 | -2.134014 |
| O | -2.457280 | -0.150789 | -3.294927 |
| O | -2.746800 | -1.717351 | -1.680207 |
| C | -3.814792 | -0.610695 | -3.434166 |
| C | -3.798096 | -1.947993 | -2.627655 |
| C | 0.300492 | 3.859363 | 4.031051 |
| C | 0.203586 | 1.995931 | 2.504377 |
| C | -0.773487 | 4.110091 | 1.882573 |
| C | -0.383487 | 4.647889 | 3.104838 |
| C | 0.583303 | 2.529136 | 3.734773 |
| H | -1.316981 | 4.728185 | 1.173483 |
| H | -0.618679 | 5.681875 | 3.338693 |
| H | 0.603111 | 4.281540 | 4.984657 |
| C | 0.670415 | -1.673360 | 2.426135 |
| C | 2.112312 | -1.117741 | 4.767278 |
| C | 0.003655 | -1.457168 | 3.634447 |
| C | 2.071563 | -1.616810 | 2.406955 |
| C | 2.786671 | -1.342773 | 3.566822 |
| C | 0.721837 | -1.179214 | 4.797328 |
| H | 2.611744 | -1.785470 | 1.478267 |
| H | 3.871461 | -1.300479 | 3.529635 |
| H | 2.668862 | -0.898275 | 5.673389 |
| C | -1.920740 | -2.327066 | 1.307510 |
| C | -4.611088 | -2.939863 | 1.810502 |
| C | -2.343159 | -3.654153 | 1.403567 |
| C | -2.882046 | -1.330410 | 1.491636 |
| C | -4.225964 | -1.601650 | 1.751571 |
| C | -3.677810 | -3.958943 | 1.644919 |
| H | -1.630623 | -4.459081 | 1.259910 |
| H | -3.993423 | -4.995733 | 1.703057 |
| H | -5.647588 | -3.198594 | 1.996925 |
| C | -2.799055 | 1.965003 | 0.204202 |
| C | -5.556337 | 1.716572 | 0.619804 |
| C | -3.676127 | 2.937024 | -0.278194 |
| C | -3.340968 | 0.889548 | 0.913477 |
| C | -4.708535 | 0.739235 | 1.142219 |
| C | -5.046919 | 2.808507 | -0.078168 |
| H | -3.288320 | 3.786140 | -0.831779 |
| H | -5.723255 | 3.562022 | -0.468757 |
| H | -6.627935 | 1.637676 | 0.766436 |
| C | -0.688988 | 3.230610 | -1.335225 |
| C | -0.074058 | 4.863689 | -3.529106 |
| C | -1.276666 | 2.991170 | -2.587220 |
| C | 0.225878 | 4.279347 | -1.204764 |
| C | 0.532151 | 5.089468 | -2.297243 |
| C | -0.978615 | 3.811465 | -3.670140 |
| H | -1.942585 | 2.141825 | -2.721287 |
| H | 0.722293 | 4.463618 | -0.258513 |
| H | 1.251714 | 5.893730 | -2.176997 |
| H | -1.445402 | 3.618725 | -4.631701 |
| H | 0.163608 | 5.496433 | -4.379035 |

| | | | |
|---|---|---|---|
| C | 0.463935 | -3.437509 | 0.167809 |
| C | 1.377239 | -5.839367 | -0.945636 |
| C | 0.988619 | -4.445003 | 0.986795 |
| C | 0.403869 | -3.646883 | -1.214101 |
| C | 0.857835 | -4.841893 | -1.767301 |
| C | 1.441021 | -5.639409 | 0.432480 |
| H | 1.040423 | -4.298957 | 2.061638 |
| H | -0.011272 | -2.868811 | -1.849695 |
| H | 0.806217 | -4.991391 | -2.841600 |
| H | 1.844989 | -6.413629 | 1.077841 |
| H | 1.733247 | -6.770185 | -1.376889 |
| H | 0.430484 | 0.959711 | 2.274136 |
| H | -1.080638 | -1.499496 | 3.675559 |
| H | 1.101975 | 1.901894 | 4.453374 |
| H | 0.188967 | -1.010591 | 5.728393 |
| O | -2.454333 | -0.028011 | 1.418116 |
| C | -5.162397 | -0.424404 | 2.021052 |
| C | -0.463988 | 2.782774 | 1.562622 |
| C | -3.387577 | -3.159025 | -3.468887 |
| H | -3.197291 | -4.003765 | -2.799817 |
| H | -4.168210 | -3.450362 | -4.179084 |
| H | -2.466591 | -2.955453 | -4.023765 |
| C | -5.081257 | -2.254611 | -1.865306 |
| H | -5.931147 | -2.357404 | -2.549125 |
| H | -4.967065 | -3.194533 | -1.316400 |
| H | -5.305512 | -1.470187 | -1.138783 |
| C | -4.148581 | -0.754516 | -4.912518 |
| H | -5.151484 | -1.175039 | -5.044931 |
| H | -4.128529 | 0.228287 | -5.394116 |
| H | -3.431248 | -1.397231 | -5.427695 |
| C | -4.709757 | 0.453592 | -2.796385 |
| H | -4.522202 | 1.416702 | -3.281151 |
| H | -5.772989 | 0.216460 | -2.905999 |
| H | -4.482114 | 0.560349 | -1.733275 |
| C | -6.629407 | -0.788460 | 1.777381 |
| H | -7.279804 | 0.061374 | 2.000108 |
| H | -6.803185 | -1.099071 | 0.742325 |
| H | -6.942005 | -1.598704 | 2.441026 |
| C | -4.989322 | -0.002288 | 3.499769 |
| H | -5.622829 | 0.862298 | 3.723630 |
| H | -5.274096 | -0.826050 | 4.162622 |
| H | -3.951729 | 0.269301 | 3.716918 |
| H | 1.657124 | 1.803399 | -1.813783 |
| C | 2.609165 | 1.046275 | 0.040370 |
| H | 1.936186 | 1.591073 | 0.703456 |
| H | 2.790904 | 0.066972 | 0.476814 |
| C | 3.904004 | 1.825575 | -0.086971 |
| C | 5.094034 | 1.234224 | -0.302895 |
| C | 3.772405 | 3.308678 | -0.026873 |
| H | 5.959871 | 1.889777 | -0.405757 |
| B | 5.356832 | -0.288021 | -0.455858 |
| C | 3.092480 | 3.924089 | 1.033342 |
| C | 4.312871 | 4.114821 | -1.034907 |
| O | 6.566734 | -0.765580 | -0.892230 |
| O | 4.457340 | -1.295646 | -0.195066 |
| C | 2.969228 | 5.308739 | 1.088908 |
| H | 2.664411 | 3.320316 | 1.828560 |
| C | 4.175400 | 5.499927 | -0.987838 |
| H | 4.825082 | 3.646573 | -1.870449 |
| C | 6.395497 | -2.171321 | -1.186505 |

| | | | |
|---|---|---|---|
| C | 5.159336 | -2.555324 | -0.301095 |
| C | 3.503855 | 6.101528 | 0.074245 |
| H | 2.445744 | 5.766203 | 1.923093 |
| H | 4.589486 | 6.109071 | -1.785809 |
| C | 7.679286 | -2.909471 | -0.840900 |
| C | 6.112542 | -2.275445 | -2.685292 |
| C | 5.535044 | -2.976617 | 1.119121 |
| C | 4.218202 | -3.578143 | -0.916597 |
| H | 3.396578 | 7.181437 | 0.111166 |
| H | 7.548055 | -3.987477 | -0.977745 |
| H | 8.485089 | -2.579047 | -1.502740 |
| H | 7.986889 | -2.722324 | 0.189923 |
| H | 6.936619 | -1.812483 | -3.235189 |
| H | 6.028016 | -3.317980 | -3.004741 |
| H | 5.189398 | -1.752259 | -2.953467 |
| H | 4.623770 | -3.071320 | 1.716969 |
| H | 6.045857 | -3.943573 | 1.125109 |
| H | 6.183997 | -2.236320 | 1.596996 |
| H | 4.730593 | -4.534392 | -1.062625 |
| H | 3.374035 | -3.745870 | -0.242971 |
| H | 3.824883 | -3.242061 | -1.877630 |

**TS1**

ΔG = -3036.409482 a.u.

| | | | |
|---|---|---|---|
| Cu | -0.239057 | 0.190960 | -0.846420 |
| C | 0.227228 | 0.471551 | -2.844841 |
| H | -0.492221 | 0.890735 | -3.550545 |
| H | 0.779797 | -0.368950 | -3.268632 |
| C | 0.984574 | 1.403017 | -2.030384 |
| P | 0.284924 | -1.650721 | 0.499172 |
| P | -1.539835 | 1.717454 | 0.316682 |
| B | -1.491764 | -0.647537 | -2.282900 |
| O | -2.680896 | -0.045233 | -2.682569 |
| O | -1.475782 | -1.977609 | -2.670196 |
| C | -3.598562 | -1.085482 | -3.073663 |
| C | -2.626135 | -2.230912 | -3.504000 |
| C | 0.260272 | 2.874513 | 4.425602 |
| C | 0.499330 | 2.025132 | 2.180237 |
| C | -1.668144 | 2.718099 | 2.983169 |
| C | -1.106137 | 3.051903 | 4.211717 |
| C | 1.061420 | 2.363133 | 3.408803 |
| H | -2.733927 | 2.855520 | 2.822926 |
| H | -1.735148 | 3.448617 | 5.003061 |
| H | 0.697375 | 3.130271 | 5.386356 |
| C | 1.100818 | -1.339816 | 2.120711 |
| C | 2.479741 | -0.744066 | 4.482787 |
| C | 0.476384 | -1.496461 | 3.357883 |
| C | 2.419044 | -0.870995 | 2.077569 |
| C | 3.110989 | -0.588969 | 3.248277 |
| C | 1.162136 | -1.189004 | 4.532922 |
| H | 2.916709 | -0.729169 | 1.123255 |
| H | 4.137246 | -0.242619 | 3.180185 |
| H | 3.012279 | -0.514457 | 5.400952 |
| C | -1.196139 | -2.676966 | 0.933314 |
| C | -3.569651 | -4.099904 | 1.436910 |
| C | -1.269815 | -4.053262 | 0.698343 |
| C | -2.343039 | -2.055170 | 1.430678 |
| C | -3.532777 | -2.733499 | 1.705489 |
| C | -2.446345 | -4.754346 | 0.938781 |
| H | -0.410026 | -4.581813 | 0.303144 |

| | | | |
|---|---|---|---|
| H | -2.487039 | -5.820011 | 0.737670 |
| H | -4.476094 | -4.665907 | 1.621611 |
| C | -3.264144 | 1.237228 | 0.740558 |
| C | -5.799960 | 0.179501 | 1.294695 |
| C | -4.414379 | 1.963563 | 0.429034 |
| C | -3.428658 | -0.004862 | 1.350156 |
| C | -4.670792 | -0.564531 | 1.636495 |
| C | -5.672917 | 1.434556 | 0.703433 |
| H | -4.327295 | 2.936058 | -0.044776 |
| H | -6.562566 | 2.002882 | 0.451230 |
| H | -6.790743 | -0.216825 | 1.488469 |
| C | -1.778274 | 3.294990 | -0.593526 |
| C | -2.144891 | 5.602016 | -2.131550 |
| C | -2.154338 | 3.196198 | -1.940544 |
| C | -1.576823 | 4.555515 | -0.029652 |
| C | -1.761920 | 5.703680 | -0.797633 |
| C | -2.340767 | 4.344979 | -2.702216 |
| H | -2.311964 | 2.214970 | -2.383073 |
| H | -1.267409 | 4.647877 | 1.006588 |
| H | -1.599997 | 6.679508 | -0.350056 |
| H | -2.635997 | 4.258410 | -3.743792 |
| H | -2.285806 | 6.498939 | -2.727402 |
| C | 1.378543 | -2.935300 | -0.241373 |
| C | 2.837018 | -4.989539 | -1.458305 |
| C | 2.138398 | -3.819946 | 0.534280 |
| C | 1.364880 | -3.083953 | -1.630965 |
| C | 2.088163 | -4.110095 | -2.235200 |
| C | 2.865036 | -4.839952 | -0.072356 |
| H | 2.153395 | -3.720808 | 1.615380 |
| H | 0.754327 | -2.420857 | -2.234347 |
| H | 2.065102 | -4.220110 | -3.315232 |
| H | 3.449929 | -5.520887 | 0.538626 |
| H | 3.401652 | -5.788060 | -1.930226 |
| H | 1.120335 | 1.615759 | 1.387495 |
| H | -0.543202 | -1.863310 | 3.418899 |
| H | 2.124192 | 2.212917 | 3.570615 |
| H | 0.663161 | -1.308298 | 5.489965 |
| O | -2.279690 | -0.700657 | 1.651631 |
| C | -4.682804 | -1.931453 | 2.315831 |
| C | -0.867335 | 2.203365 | 1.957408 |
| C | -6.028720 | -2.640374 | 2.155121 |
| H | -6.018129 | -3.607450 | 2.664929 |
| H | -6.828416 | -2.053554 | 2.614962 |
| H | -6.276963 | -2.803180 | 1.101554 |
| C | -4.394452 | -1.733514 | 3.822770 |
| H | -5.187480 | -1.134390 | 4.281873 |
| H | -4.351854 | -2.703220 | 4.329267 |
| H | -3.442369 | -1.219337 | 3.984089 |
| C | -2.161192 | -2.109956 | -4.954977 |
| H | -1.340850 | -2.813438 | -5.125358 |
| H | -2.965799 | -2.344195 | -5.658423 |
| H | -1.793653 | -1.101499 | -5.169244 |
| C | -3.132399 | -3.637988 | -3.224523 |
| H | -2.390730 | -4.370783 | -3.556871 |
| H | -3.307496 | -3.795026 | -2.157880 |
| H | -4.064441 | -3.828369 | -3.766995 |
| C | -4.429350 | -1.439383 | -1.839225 |
| H | -3.798428 | -1.845629 | -1.043549 |
| H | -4.906954 | -0.530863 | -1.461430 |
| H | -5.209822 | -2.170442 | -2.072410 |

| | | | |
|---|---|---|---|
| C | -4.500480 | -0.559426 | -4.179765 |
| H | -3.921953 | -0.146192 | -5.008850 |
| H | -5.144429 | -1.356954 | -4.564869 |
| H | -5.143149 | 0.233524 | -3.785079 |
| C | 2.503540 | 1.399147 | -2.019275 |
| H | 2.893002 | 0.382008 | -1.914686 |
| H | 2.903313 | 1.772979 | -2.980818 |
| C | 3.085882 | 2.271115 | -0.920322 |
| C | 4.092568 | 1.875804 | -0.116829 |
| C | 2.529347 | 3.649714 | -0.773352 |
| H | 4.421757 | 2.579419 | 0.650124 |
| B | 4.923707 | 0.571123 | -0.223295 |
| C | 2.185497 | 4.405522 | -1.903435 |
| C | 2.388995 | 4.248731 | 0.484392 |
| O | 5.399542 | 0.046281 | -1.396858 |
| O | 5.335268 | -0.136942 | 0.879551 |
| C | 1.744714 | 5.718950 | -1.779908 |
| H | 2.276410 | 3.967146 | -2.892647 |
| C | 1.937814 | 5.558774 | 0.611701 |
| H | 2.622025 | 3.676834 | 1.376015 |
| C | 5.994884 | -1.236342 | -1.099995 |
| C | 6.281099 | -1.138535 | 0.444560 |
| C | 1.622693 | 6.303850 | -0.521396 |
| H | 1.491070 | 6.285656 | -2.670886 |
| H | 1.828705 | 5.993947 | 1.600915 |
| C | 7.233888 | -1.414494 | -1.965269 |
| C | 4.952840 | -2.290670 | -1.456970 |
| C | 7.672019 | -0.598715 | 0.773229 |
| C | 6.016394 | -2.421393 | 1.219875 |
| H | 1.271680 | 7.326940 | -0.425210 |
| H | 7.746216 | -2.348005 | -1.711805 |
| H | 6.941950 | -1.463358 | -3.018331 |
| H | 7.935824 | -0.586841 | -1.845380 |
| H | 4.678134 | -2.176900 | -2.509207 |
| H | 5.339428 | -3.302322 | -1.310126 |
| H | 4.045685 | -2.174485 | -0.857650 |
| H | 7.730110 | -0.396257 | 1.846482 |
| H | 8.452927 | -1.320254 | 0.516955 |
| H | 7.871789 | 0.336094 | 0.241026 |
| H | 6.666071 | -3.226797 | 0.862665 |
| H | 6.227533 | -2.261094 | 2.281288 |
| H | 4.976618 | -2.742309 | 1.124354 |
| H | 0.556818 | 2.403100 | -2.011943 |

**I4**

ΔG = -3036.47335 a.u.

| | | | |
|---|---|---|---|
| Cu | -0.052890 | -0.287693 | -0.676207 |
| C | -0.793585 | -0.290020 | -3.631597 |
| H | -0.934002 | -0.913416 | -4.535096 |
| H | -1.606206 | 0.450057 | -3.659585 |
| C | -0.887800 | -1.133839 | -2.331819 |
| H | -0.251347 | -2.013887 | -2.497081 |
| P | -0.396297 | 1.737097 | 0.496142 |
| P | 1.667017 | -1.538521 | 0.550981 |
| B | 0.570154 | 0.473342 | -3.672901 |
| O | 1.795111 | -0.129124 | -3.874429 |
| O | 0.693240 | 1.832645 | -3.474904 |
| C | 2.816736 | 0.841031 | -3.554463 |
| C | 2.061464 | 2.201191 | -3.742658 |
| C | 0.192383 | -2.703985 | 4.786483 |

| | | | |
|---|---|---|---|
| C | -0.231737 | -2.001997 | 2.519202 |
| C | 2.034529 | -2.347403 | 3.267993 |
| C | 1.562504 | -2.693773 | 4.530851 |
| C | -0.704233 | -2.357143 | 3.779492 |
| H | 3.103943 | -2.337689 | 3.077276 |
| H | 2.265386 | -2.954470 | 5.316412 |
| H | -0.174457 | -2.973814 | 5.772305 |
| C | -1.029480 | 1.416017 | 2.196128 |
| C | -2.159304 | 0.792757 | 4.680225 |
| C | -2.245361 | 0.726410 | 2.274104 |
| C | -0.380022 | 1.782915 | 3.374764 |
| C | -0.941382 | 1.463427 | 4.611189 |
| C | -2.815291 | 0.428010 | 3.504891 |
| H | 0.559093 | 2.326583 | 3.340937 |
| H | -0.425158 | 1.749514 | 5.522677 |
| H | -2.594772 | 0.553228 | 5.645679 |
| C | 0.984604 | 2.937997 | 0.723372 |
| C | 3.257649 | 4.576404 | 0.904575 |
| C | 0.924230 | 4.283924 | 0.350672 |
| C | 2.210778 | 2.455243 | 1.185109 |
| C | 3.354538 | 3.245158 | 1.305933 |
| C | 2.053155 | 5.091364 | 0.432866 |
| H | -0.000961 | 4.699306 | -0.031731 |
| H | 1.994052 | 6.130548 | 0.126117 |
| H | 4.123622 | 5.226325 | 0.965783 |
| C | 3.353499 | -0.843150 | 0.853989 |
| C | 5.820962 | 0.467146 | 1.130094 |
| C | 4.549671 | -1.514956 | 0.589246 |
| C | 3.444026 | 0.482063 | 1.279586 |
| C | 4.650635 | 1.165589 | 1.424214 |
| C | 5.771403 | -0.863624 | 0.724926 |
| H | 4.527999 | -2.545430 | 0.251932 |
| H | 6.691969 | -1.395664 | 0.507221 |
| H | 6.783763 | 0.957890 | 1.220766 |
| C | 2.118478 | -3.121543 | -0.273466 |
| C | 3.041733 | -5.354423 | -1.683749 |
| C | 2.206595 | -3.090804 | -1.670590 |
| C | 2.473704 | -4.289009 | 0.407692 |
| C | 2.936292 | -5.398712 | -0.295343 |
| C | 2.672193 | -4.200508 | -2.370784 |
| H | 1.927321 | -2.190682 | -2.212738 |
| H | 2.398468 | -4.332954 | 1.490015 |
| H | 3.212150 | -6.300160 | 0.243733 |
| H | 2.741297 | -4.162434 | -3.453809 |
| H | 3.404204 | -6.220641 | -2.229365 |
| C | -1.732499 | 2.808668 | -0.175875 |
| C | -3.717568 | 4.420082 | -1.303241 |
| C | -2.342774 | 3.814472 | 0.585018 |
| C | -2.133298 | 2.610729 | -1.497625 |
| C | -3.122793 | 3.415516 | -2.060988 |
| C | -3.326924 | 4.618830 | 0.021900 |
| H | -2.045860 | 3.967369 | 1.618987 |
| H | -1.664409 | 1.826379 | -2.082329 |
| H | -3.428962 | 3.251121 | -3.089621 |
| H | -3.794990 | 5.397064 | 0.616972 |
| H | -4.490250 | 5.045394 | -1.740522 |
| H | -0.932887 | -1.711432 | 1.741576 |
| H | -2.757364 | 0.421730 | 1.366743 |
| H | -1.771804 | -2.347740 | 3.975005 |
| H | -3.765772 | -0.096147 | 3.533309 |

| | | | |
|---|---|---|---|
| O | 2.258577 | 1.128897 | 1.536232 |
| C | 4.593868 | 2.605442 | 1.929460 |
| C | -2.589804 | -2.468228 | -0.939456 |
| C | 1.138682 | -2.007843 | 2.247938 |
| C | -3.512459 | -2.122510 | -0.013537 |
| C | 5.870550 | 3.383047 | 1.603363 |
| H | 5.810543 | 4.401802 | 1.995355 |
| H | 6.736699 | 2.916648 | 2.079971 |
| H | 6.050810 | 3.433746 | 0.525079 |
| C | 4.402401 | 2.576272 | 3.464636 |
| H | 5.261917 | 2.094274 | 3.941605 |
| H | 4.313487 | 3.596037 | 3.852983 |
| H | 3.501830 | 2.022075 | 3.745605 |
| C | 2.108467 | 2.723261 | -5.178867 |
| H | 1.409399 | 3.558845 | -5.276394 |
| H | 3.108183 | 3.079209 | -5.444561 |
| H | 1.812882 | 1.948671 | -5.893078 |
| C | 2.481632 | 3.295253 | -2.772379 |
| H | 1.887554 | 4.196431 | -2.950871 |
| H | 2.331897 | 2.990848 | -1.735570 |
| H | 3.537227 | 3.549595 | -2.913761 |
| C | 3.230067 | 0.594391 | -2.106689 |
| H | 2.380236 | 0.729331 | -1.429047 |
| H | 3.581489 | -0.435140 | -2.004270 |
| H | 4.036193 | 1.262977 | -1.791094 |
| C | 4.005275 | 0.633290 | -4.480263 |
| H | 3.708156 | 0.653741 | -5.530886 |
| H | 4.760324 | 1.408438 | -4.314128 |
| H | 4.466175 | -0.337743 | -4.275360 |
| B | -4.570535 | -0.990992 | -0.074119 |
| O | -5.102871 | -0.424528 | -1.208445 |
| O | -5.176756 | -0.527988 | 1.073777 |
| C | -6.311951 | 0.265393 | -0.818673 |
| C | -6.051469 | 0.560965 | 0.697215 |
| C | -6.481284 | 1.499072 | -1.691303 |
| H | -7.343956 | 2.086156 | -1.360081 |
| H | -5.593726 | 2.133760 | -1.665756 |
| H | -6.655052 | 1.196138 | -2.728109 |
| C | -7.470126 | -0.705550 | -1.050765 |
| H | -8.434360 | -0.233774 | -0.840536 |
| H | -7.463369 | -1.023138 | -2.097173 |
| H | -7.374800 | -1.598034 | -0.424755 |
| C | -7.289468 | 0.524026 | 1.579523 |
| H | -7.774832 | -0.453837 | 1.555494 |
| H | -7.010379 | 0.741241 | 2.614968 |
| H | -8.010987 | 1.281492 | 1.257104 |
| C | -5.281943 | 1.860543 | 0.915725 |
| H | -5.907437 | 2.732373 | 0.704384 |
| H | -4.951175 | 1.918042 | 1.956296 |
| H | -4.397377 | 1.905405 | 0.275624 |
| C | -2.336585 | -1.635800 | -2.176117 |
| H | -2.646548 | -2.225184 | -3.062255 |
| H | -3.011829 | -0.776675 | -2.150673 |
| H | -3.610734 | -2.763069 | 0.864656 |
| C | -1.792281 | -3.722490 | -0.779752 |
| C | -1.283581 | -4.391056 | -1.902733 |
| C | -1.570386 | -4.306671 | 0.476296 |
| C | -0.607178 | -5.600617 | -1.778008 |
| H | -1.428364 | -3.971804 | -2.892407 |
| C | -0.886878 | -5.509436 | 0.605380 |

| | | | |
|---|---|---|---|
| H | -1.924058 | -3.811515 | 1.373179 |
| C | -0.408482 | -6.168984 | -0.523966 |
| H | -0.229207 | -6.096203 | -2.667253 |
| H | -0.722194 | -5.927487 | 1.594247 |
| H | 0.129169 | -7.107068 | -0.425774 |

**TS2**

ΔG = -3036.443219 a.u.

| | | | |
|---|---|---|---|
| Cu | -0.238082 | -0.322468 | 0.748995 |
| C | 1.386902 | 2.355561 | 2.101664 |
| H | 0.810783 | 3.175763 | 2.543554 |
| H | 1.635919 | 1.688720 | 2.933866 |
| C | 0.519525 | 1.630403 | 1.027000 |
| H | 1.235746 | 1.237343 | 0.280676 |
| P | 1.526650 | -1.806262 | 0.058271 |
| P | -2.166737 | -0.897624 | -0.610421 |
| B | 2.676777 | 2.828716 | 1.364727 |
| O | 2.753240 | 3.857085 | 0.448319 |
| O | 3.860212 | 2.120731 | 1.434154 |
| C | 3.911015 | 3.602983 | -0.375636 |
| C | 4.825836 | 2.763994 | 0.580648 |
| C | -3.366278 | -5.278202 | 0.307982 |
| C | -2.696512 | -3.066824 | 0.994871 |
| C | -2.873887 | -3.592103 | -1.353391 |
| C | -3.243517 | -4.894901 | -1.025328 |
| C | -3.087016 | -4.361955 | 1.319758 |
| H | -2.785864 | -3.309242 | -2.397306 |
| H | -3.437413 | -5.611844 | -1.817412 |
| H | -3.663862 | -6.292300 | 0.557272 |
| C | 1.055271 | -3.471303 | -0.577524 |
| C | 0.210066 | -6.005481 | -1.424506 |
| C | 0.445326 | -4.357647 | 0.320573 |
| C | 1.219202 | -3.864881 | -1.908111 |
| C | 0.790384 | -5.122718 | -2.329167 |
| C | 0.041972 | -5.620740 | -0.095388 |
| H | 1.682194 | -3.196077 | -2.626404 |
| H | 0.919756 | -5.412059 | -3.367718 |
| H | -0.117949 | -6.987062 | -1.752789 |
| C | 2.379548 | -1.081729 | -1.410967 |
| C | 3.345064 | -0.274496 | -3.915999 |
| C | 3.716717 | -1.295137 | -1.751086 |
| C | 1.583556 | -0.403240 | -2.332369 |
| C | 2.007446 | -0.031793 | -3.605333 |
| C | 4.197471 | -0.872478 | -2.987680 |
| H | 4.374953 | -1.811271 | -1.059042 |
| H | 5.238727 | -1.037951 | -3.244623 |
| H | 3.735888 | 0.003653 | -4.889046 |
| C | -1.925345 | -0.708367 | -2.430433 |
| C | -1.407923 | -0.197823 | -5.142634 |
| C | -2.933052 | -0.869359 | -3.388532 |
| C | -0.677152 | -0.286616 | -2.885021 |
| C | -0.386533 | 0.007055 | -4.218689 |
| C | -2.668043 | -0.638305 | -4.733053 |
| H | -3.927778 | -1.171575 | -3.074992 |
| H | -3.452012 | -0.780828 | -5.469624 |
| H | -1.236419 | 0.002988 | -6.194875 |
| C | -3.784484 | -0.023755 | -0.396246 |
| C | -6.101694 | 1.490418 | 0.063854 |
| C | -3.995607 | 1.183704 | -1.071074 |
| C | -4.749734 | -0.461462 | 0.514778 |

| | | | |
|---|---|---|---|
| C | -5.899991 | 0.290986 | 0.741610 |
| C | -5.144926 | 1.933516 | -0.845372 |
| H | -3.251318 | 1.558357 | -1.767191 |
| H | -4.615482 | -1.393624 | 1.053691 |
| H | -6.640191 | -0.066087 | 1.451583 |
| H | -5.276790 | 2.877524 | -1.365799 |
| H | -6.995762 | 2.078719 | 0.246699 |
| C | 2.943808 | -2.124912 | 1.184818 |
| C | 5.088581 | -2.381388 | 2.962027 |
| C | 3.467924 | -3.385899 | 1.474978 |
| C | 3.504373 | -0.993161 | 1.788735 |
| C | 4.575941 | -1.119120 | 2.664716 |
| C | 4.533039 | -3.510937 | 2.366694 |
| H | 3.052291 | -4.274230 | 1.009716 |
| H | 3.122645 | -0.002599 | 1.562352 |
| H | 5.002026 | -0.227340 | 3.113906 |
| H | 4.932086 | -4.495992 | 2.589606 |
| H | 5.919449 | -2.484060 | 3.653657 |
| H | -2.460636 | -2.350956 | 1.778560 |
| H | 0.289196 | -4.060579 | 1.354223 |
| H | -3.164153 | -4.656699 | 2.362366 |
| H | -0.420584 | -6.298074 | 0.615169 |
| O | 0.299967 | -0.140147 | -1.942632 |
| C | 0.980480 | 0.628349 | -4.527273 |
| C | -1.719229 | 2.835908 | 0.876073 |
| C | -2.614778 | -2.662551 | -0.344640 |
| C | -2.125294 | 2.051416 | 1.885622 |
| C | 0.903440 | 2.132784 | -4.175293 |
| H | 1.870232 | 2.611716 | -4.360153 |
| H | 0.144573 | 2.625628 | -4.791639 |
| H | 0.643546 | 2.282492 | -3.122777 |
| C | 1.361017 | 0.477136 | -6.001652 |
| H | 0.624230 | 0.970036 | -6.641502 |
| H | 2.321686 | 0.959448 | -6.201044 |
| H | 1.430853 | -0.574518 | -6.296003 |
| C | 5.712242 | 3.627772 | 1.476984 |
| H | 6.163805 | 2.994676 | 2.246401 |
| H | 6.516587 | 4.103751 | 0.908490 |
| H | 5.129469 | 4.406920 | 1.977293 |
| C | 5.657326 | 1.692608 | -0.110031 |
| H | 6.257236 | 1.154830 | 0.630425 |
| H | 5.026221 | 0.965757 | -0.625818 |
| H | 6.340276 | 2.143353 | -0.837619 |
| C | 3.421792 | 2.802556 | -1.584499 |
| H | 3.005567 | 1.836375 | -1.281926 |
| H | 2.630170 | 3.370358 | -2.079996 |
| H | 4.224640 | 2.623632 | -2.305777 |
| C | 4.503828 | 4.928183 | -0.828355 |
| H | 4.703544 | 5.589537 | 0.017342 |
| H | 5.439116 | 4.764141 | -1.373360 |
| H | 3.805008 | 5.436274 | -1.499610 |
| B | -1.225572 | 1.013311 | 2.655457 |
| O | -0.636838 | 1.421610 | 3.861364 |
| O | -1.580154 | -0.367583 | 2.773818 |
| C | -0.801983 | 0.394960 | 4.838459 |
| C | -0.953192 | -0.889441 | 3.962878 |
| C | 0.404625 | 0.391050 | 5.768481 |
| H | 0.353630 | -0.447717 | 6.470900 |
| H | 1.339883 | 0.323532 | 5.208377 |
| H | 0.424347 | 1.318193 | 6.349413 |

| | | | |
|---|---|---|---|
| C | -2.070797 | 0.719387 | 5.633397 |
| H | -2.229231 | 0.015162 | 6.456008 |
| H | -1.975364 | 1.724263 | 6.054783 |
| H | -2.954096 | 0.705913 | 4.987408 |
| C | -1.848886 | -1.966117 | 4.561504 |
| H | -2.879968 | -1.620809 | 4.663699 |
| H | -1.846240 | -2.855981 | 3.923778 |
| H | -1.479957 | -2.264833 | 5.548391 |
| C | 0.395860 | -1.495660 | 3.581802 |
| H | 0.895908 | -1.942447 | 4.446459 |
| H | 0.235133 | -2.282789 | 2.838977 |
| H | 1.057246 | -0.742735 | 3.143954 |
| C | -0.358256 | 2.608751 | 0.251399 |
| H | -0.521610 | 2.209602 | -0.762433 |
| H | 0.139130 | 3.581811 | 0.103965 |
| H | -3.144299 | 2.177648 | 2.260561 |
| C | -2.585143 | 3.892315 | 0.285646 |
| C | -2.428528 | 4.291456 | -1.050136 |
| C | -3.598092 | 4.510635 | 1.033322 |
| C | -3.275837 | 5.234461 | -1.626500 |
| H | -1.647762 | 3.845966 | -1.658442 |
| C | -4.446078 | 5.452592 | 0.461490 |
| H | -3.717820 | 4.254151 | 2.081532 |
| C | -4.294517 | 5.816284 | -0.875714 |
| H | -3.138335 | 5.514273 | -2.667227 |
| H | -5.223590 | 5.911874 | 1.065416 |
| H | -4.954585 | 6.553712 | -1.322817 |

**I5**

ΔG = -3036.482411 a.u.

| | | | |
|---|---|---|---|
| Cu | 0.098156 | 0.818054 | 0.296544 |
| C | -1.340271 | 1.275596 | -2.822347 |
| H | -2.002620 | 1.814426 | -3.508801 |
| H | -0.554869 | 1.978445 | -2.516001 |
| C | -2.131849 | 0.833555 | -1.569048 |
| H | -1.525134 | 0.069172 | -1.041396 |
| P | 2.318023 | 1.209865 | -0.368167 |
| P | -0.373554 | -0.962964 | 1.759630 |
| B | -0.710783 | -0.004259 | -3.453655 |
| O | -1.261973 | -0.773017 | -4.450493 |
| O | 0.440197 | -0.572423 | -2.941336 |
| C | -0.602190 | -2.058243 | -4.420824 |
| C | 0.774545 | -1.718200 | -3.749721 |
| C | -0.375374 | 1.041821 | 5.926450 |
| C | -1.021100 | 0.986893 | 3.601995 |
| C | 0.268712 | -0.830340 | 4.541693 |
| C | 0.271950 | -0.182141 | 5.775367 |
| C | -1.024733 | 1.624207 | 4.838464 |
| H | 0.776088 | -1.784112 | 4.433314 |
| H | 0.780998 | -0.637062 | 6.619624 |
| H | -0.370516 | 1.543782 | 6.889254 |
| C | 3.185433 | 1.769747 | 1.158167 |
| C | 4.419778 | 2.513095 | 3.554887 |
| C | 2.422642 | 2.018935 | 2.301901 |
| C | 4.578205 | 1.888432 | 1.227576 |
| C | 5.190559 | 2.262778 | 2.419301 |
| C | 3.034631 | 2.389221 | 3.496802 |
| H | 5.184589 | 1.681404 | 0.349863 |
| H | 6.271479 | 2.353711 | 2.464721 |
| H | 4.902117 | 2.798117 | 4.484990 |

| | | | |
|---|---|---|---|
| C | 3.395999 | -0.197478 | -0.865234 |
| C | 5.024658 | -2.420832 | -1.368483 |
| C | 4.263692 | -0.203445 | -1.957704 |
| C | 3.376004 | -1.326786 | -0.046487 |
| C | 4.195107 | -2.435746 | -0.247300 |
| C | 5.055292 | -1.318518 | -2.218558 |
| H | 4.317443 | 0.659325 | -2.612640 |
| H | 5.714015 | -1.320216 | -3.080859 |
| H | 5.667550 | -3.268547 | -1.577967 |
| C | 0.666257 | -2.483055 | 1.886146 |
| C | 2.188468 | -4.834961 | 1.798275 |
| C | 0.143356 | -3.691020 | 2.363559 |
| C | 1.981384 | -2.489177 | 1.418290 |
| C | 2.757532 | -3.649421 | 1.340553 |
| C | 0.898718 | -4.854694 | 2.324520 |
| H | -0.872137 | -3.721622 | 2.744637 |
| H | 0.478527 | -5.785049 | 2.692089 |
| H | 2.755814 | -5.758196 | 1.756981 |
| C | -2.024013 | -1.743719 | 1.554627 |
| C | -4.408832 | -3.127721 | 1.107890 |
| C | -2.241179 | -2.410587 | 0.343715 |
| C | -3.016187 | -1.759923 | 2.533719 |
| C | -4.207135 | -2.448890 | 2.305203 |
| C | -3.423643 | -3.104299 | 0.122176 |
| H | -1.471875 | -2.392899 | -0.424635 |
| H | -2.862703 | -1.246619 | 3.478193 |
| H | -4.980112 | -2.452382 | 3.067593 |
| H | -3.582039 | -3.614038 | -0.823507 |
| H | -5.344275 | -3.648807 | 0.929268 |
| C | 2.692044 | 2.480066 | -1.642680 |
| C | 3.022670 | 4.411809 | -3.640551 |
| C | 3.321617 | 3.692625 | -1.353261 |
| C | 2.227085 | 2.243585 | -2.942949 |
| C | 2.398181 | 3.201421 | -3.936795 |
| C | 3.481679 | 4.654445 | -2.349022 |
| H | 3.677919 | 3.900384 | -0.349463 |
| H | 1.726899 | 1.306467 | -3.170418 |
| H | 2.033913 | 3.004600 | -4.940585 |
| H | 3.966681 | 5.596176 | -2.110623 |
| H | 3.147807 | 5.164142 | -4.413286 |
| H | -1.495728 | 1.454183 | 2.740888 |
| H | 1.342189 | 1.906805 | 2.266879 |
| H | -1.523566 | 2.582593 | 4.948244 |
| H | 2.426472 | 2.569884 | 4.377997 |
| O | 2.510985 | -1.291123 | 1.017611 |
| C | 4.189536 | -3.524634 | 0.822857 |
| C | -4.327893 | 0.334815 | -0.629404 |
| C | -0.377666 | -0.248651 | 3.449332 |
| C | -3.813126 | 1.252072 | 0.209982 |
| C | 4.728845 | -4.854996 | 0.291537 |
| H | 5.758515 | -4.739168 | -0.056823 |
| H | 4.752799 | -5.606896 | 1.084482 |
| H | 4.121386 | -5.237089 | -0.534659 |
| C | 5.087930 | -3.048146 | 1.989662 |
| H | 5.079277 | -3.787573 | 2.796986 |
| H | 6.118511 | -2.920355 | 1.643024 |
| H | 4.743487 | -2.092388 | 2.396036 |
| C | 1.837141 | -1.266302 | -4.751102 |
| H | 2.696894 | -0.874561 | -4.200132 |
| H | 2.180919 | -2.097857 | -5.373154 |

| | | | |
|---|---|---|---|
| H | 1.457657 | -0.474687 | -5.404748 |
| C | 1.336557 | -2.807733 | -2.847830 |
| H | 2.305747 | -2.493724 | -2.450831 |
| H | 0.673963 | -3.012756 | -2.003529 |
| H | 1.484958 | -3.735263 | -3.410347 |
| C | -1.472138 | -2.976785 | -3.562033 |
| H | -1.551208 | -2.594018 | -2.540584 |
| H | -2.479031 | -3.014243 | -3.987225 |
| H | -1.073181 | -3.994435 | -3.525024 |
| C | -0.503739 | -2.601227 | -5.837832 |
| H | -0.020839 | -1.888783 | -6.509825 |
| H | 0.065496 | -3.536257 | -5.850578 |
| H | -1.505737 | -2.808640 | -6.224682 |
| B | -2.515424 | 1.973571 | -0.456430 |
| O | -2.793716 | 3.294467 | -1.034431 |
| O | -1.372587 | 2.290459 | 0.527214 |
| C | -2.468348 | 4.279604 | -0.089958 |
| C | -1.174131 | 3.708703 | 0.598421 |
| C | -2.246431 | 5.602661 | -0.822831 |
| H | -1.867375 | 6.378880 | -0.148594 |
| H | -1.544328 | 5.481495 | -1.650941 |
| H | -3.197475 | 5.952313 | -1.238031 |
| C | -3.626242 | 4.471549 | 0.906915 |
| H | -3.469329 | 5.342371 | 1.552735 |
| H | -4.548619 | 4.626174 | 0.338594 |
| H | -3.768629 | 3.596062 | 1.543675 |
| C | -1.006207 | 4.130437 | 2.056984 |
| H | -1.844048 | 3.800118 | 2.674709 |
| H | -0.088608 | 3.701554 | 2.472972 |
| H | -0.929959 | 5.220847 | 2.135132 |
| C | 0.095789 | 4.077189 | -0.164200 |
| H | 0.293272 | 5.153282 | -0.137591 |
| H | 0.948018 | 3.581115 | 0.306785 |
| H | 0.039020 | 3.754507 | -1.207036 |
| C | -3.469322 | 0.129511 | -1.869418 |
| H | -3.353166 | -0.936005 | -2.114445 |
| H | -3.950650 | 0.586786 | -2.748016 |
| H | -4.335448 | 1.512543 | 1.132697 |
| C | -5.600021 | -0.408359 | -0.468974 |
| C | -6.076581 | -1.244625 | -1.489233 |
| C | -6.367582 | -0.320466 | 0.704250 |
| C | -7.251963 | -1.978019 | -1.338128 |
| H | -5.518939 | -1.332414 | -2.416337 |
| C | -7.540063 | -1.048662 | 0.858304 |
| H | -6.028733 | 0.311852 | 1.518876 |
| C | -7.989551 | -1.888911 | -0.161490 |
| H | -7.590937 | -2.620389 | -2.146312 |
| H | -8.106199 | -0.965003 | 1.781998 |
| H | -8.905094 | -2.460346 | -0.040166 |

**TS3**

ΔG = -3036.455949 a.u.

| | | | |
|---|---|---|---|
| Cu | 0.390182 | -0.010158 | -0.165713 |
| C | -3.733326 | -2.584995 | 0.028978 |
| H | -3.581331 | -3.255259 | -0.826993 |
| H | -3.969171 | -3.243414 | 0.880401 |
| C | -2.419686 | -1.817900 | 0.336249 |
| H | -1.653953 | -2.575017 | 0.511009 |
| P | 1.836777 | 1.941369 | -0.398371 |
| P | 1.637790 | -1.832286 | 0.686313 |

| | | | |
|---|---|---|---|
| B | -4.991012 | -1.678695 | -0.235276 |
| O | -5.317039 | -0.604056 | 0.555390 |
| O | -5.942985 | -1.922787 | -1.198762 |
| C | -6.653995 | -0.193080 | 0.224683 |
| C | -6.836863 | -0.787353 | -1.215078 |
| C | 2.689782 | -0.968260 | 5.114221 |
| C | 1.286949 | -0.655717 | 3.177343 |
| C | 3.225844 | -2.088333 | 3.044071 |
| C | 3.516836 | -1.814284 | 4.377048 |
| C | 1.573157 | -0.392707 | 4.514378 |
| H | 3.871521 | -2.753606 | 2.478160 |
| H | 4.389218 | -2.263988 | 4.841547 |
| H | 2.918710 | -0.755617 | 6.154177 |
| C | 2.148223 | 2.440909 | 1.350691 |
| C | 2.501593 | 3.129542 | 4.044149 |
| C | 1.042581 | 2.600013 | 2.197490 |
| C | 3.431070 | 2.625264 | 1.871879 |
| C | 3.605406 | 2.956334 | 3.214082 |
| C | 1.218226 | 2.958685 | 3.529515 |
| H | 4.303794 | 2.515964 | 1.235663 |
| H | 4.609193 | 3.086666 | 3.607105 |
| H | 2.640540 | 3.393814 | 5.088114 |
| C | 3.518916 | 1.968070 | -1.150923 |
| C | 6.036551 | 1.723321 | -2.358939 |
| C | 4.019193 | 2.992083 | -1.958873 |
| C | 4.331812 | 0.851876 | -0.945927 |
| C | 5.587274 | 0.694977 | -1.531614 |
| C | 5.265194 | 2.864909 | -2.563307 |
| H | 3.426451 | 3.884162 | -2.130362 |
| H | 5.640160 | 3.661963 | -3.197164 |
| H | 7.004680 | 1.645903 | -2.841376 |
| C | 3.233598 | -2.384539 | -0.061646 |
| C | 5.700287 | -3.034240 | -1.230821 |
| C | 3.570295 | -3.716276 | -0.317611 |
| C | 4.177189 | -1.408977 | -0.389597 |
| C | 5.417081 | -1.696071 | -0.960798 |
| C | 4.787889 | -4.035473 | -0.909569 |
| H | 2.873953 | -4.507625 | -0.062897 |
| H | 5.031567 | -5.073092 | -1.113522 |
| H | 6.647640 | -3.307133 | -1.682757 |
| C | 0.688211 | -3.405392 | 0.775896 |
| C | -1.028776 | -5.616317 | 0.784686 |
| C | 0.283123 | -3.996819 | -0.428448 |
| C | 0.233501 | -3.940373 | 1.982636 |
| C | -0.627677 | -5.037062 | 1.984583 |
| C | -0.562018 | -5.099454 | -0.423766 |
| H | 0.603404 | -3.568917 | -1.372474 |
| H | 0.531610 | -3.494101 | 2.926161 |
| H | -0.985217 | -5.435988 | 2.929009 |
| H | -0.873662 | -5.543174 | -1.364521 |
| H | -1.704006 | -6.466570 | 0.788725 |
| C | 1.054281 | 3.431188 | -1.154983 |
| C | -0.245012 | 5.560220 | -2.437000 |
| C | 0.690473 | 4.573904 | -0.438793 |
| C | 0.763950 | 3.372330 | -2.524627 |
| C | 0.126850 | 4.430059 | -3.163330 |
| C | 0.038677 | 5.628443 | -1.076487 |
| H | 0.910496 | 4.650429 | 0.620620 |
| H | 1.042516 | 2.492349 | -3.098707 |
| H | -0.087420 | 4.367266 | -4.225881 |

| | | | |
|---|---|---|---|
| H | -0.242411 | 6.507266 | -0.504096 |
| H | -0.752456 | 6.383204 | -2.930927 |
| H | 0.425696 | -0.191037 | 2.703402 |
| H | 0.038810 | 2.437706 | 1.816273 |
| H | 0.929512 | 0.272851 | 5.080848 |
| H | 0.349164 | 3.086499 | 4.167895 |
| O | 3.841408 | -0.110850 | -0.100651 |
| C | 6.400418 | -0.542753 | -1.157975 |
| C | 2.109280 | -1.507754 | 2.434121 |
| C | -1.378208 | 0.860454 | 0.362454 |
| C | 7.467554 | -0.867461 | -2.206396 |
| H | 8.171717 | -0.037749 | -2.309444 |
| H | 8.055478 | -1.737014 | -1.901399 |
| H | 7.024014 | -1.071830 | -3.185648 |
| C | 7.094194 | -0.269576 | 0.198313 |
| H | 7.662125 | -1.150446 | 0.514834 |
| H | 7.783320 | 0.576104 | 0.105224 |
| H | 6.365604 | -0.033764 | 0.980085 |
| C | -6.337551 | 0.154088 | -2.310342 |
| H | -6.330536 | -0.383758 | -3.262849 |
| H | -6.985695 | 1.029422 | -2.414237 |
| H | -5.316135 | 0.481301 | -2.099016 |
| C | -8.244564 | -1.268651 | -1.532769 |
| H | -8.278034 | -1.669720 | -2.550253 |
| H | -8.567401 | -2.055191 | -0.847233 |
| H | -8.956048 | -0.438594 | -1.472421 |
| C | -7.581332 | -0.826841 | 1.262898 |
| H | -7.539074 | -1.919552 | 1.219176 |
| H | -7.257275 | -0.514164 | 2.259858 |
| H | -8.619782 | -0.512073 | 1.123308 |
| C | -6.737214 | 1.323619 | 0.312247 |
| H | -5.979252 | 1.802510 | -0.309651 |
| H | -7.726360 | 1.674651 | -0.000573 |
| H | -6.570798 | 1.640434 | 1.346250 |
| B | -1.961461 | -1.011774 | -0.966157 |
| O | -2.904991 | -0.465172 | -1.834937 |
| O | -0.807530 | -1.377061 | -1.706150 |
| C | -2.271442 | -0.165626 | -3.084191 |
| C | -1.068765 | -1.173250 | -3.101357 |
| C | -3.271418 | -0.371311 | -4.214735 |
| H | -2.770687 | -0.308585 | -5.186789 |
| H | -3.769311 | -1.340175 | -4.135544 |
| H | -4.037679 | 0.408109 | -4.181458 |
| C | -1.827195 | 1.295438 | -3.045999 |
| H | -1.392698 | 1.609877 | -3.999989 |
| H | -2.696550 | 1.926703 | -2.838470 |
| H | -1.097248 | 1.461468 | -2.252172 |
| C | 0.197641 | -0.650365 | -3.764243 |
| H | 0.574927 | 0.234942 | -3.245640 |
| H | 0.977375 | -1.418050 | -3.728610 |
| H | 0.017743 | -0.395889 | -4.813869 |
| C | -1.443731 | -2.539001 | -3.680881 |
| H | -1.618453 | -2.490965 | -4.759893 |
| H | -0.623768 | -3.240226 | -3.499014 |
| H | -2.342177 | -2.934655 | -3.197627 |
| C | -2.323423 | 0.537405 | 1.268899 |
| C | -2.523588 | -0.934257 | 1.605049 |
| H | -1.763915 | -1.249958 | 2.334996 |
| H | -3.493087 | -1.088379 | 2.085473 |
| H | -1.346782 | 1.933035 | 0.124276 |

| | | | |
|---|---|---|---|
| C | -3.206048 | 1.537347 | 1.930225 |
| C | -3.533959 | 2.751687 | 1.304879 |
| C | -3.734801 | 1.308646 | 3.209698 |
| C | -4.344746 | 3.692864 | 1.928989 |
| H | -3.168178 | 2.946321 | 0.300866 |
| C | -4.545477 | 2.250232 | 3.839426 |
| H | -3.502101 | 0.383333 | 3.728643 |
| C | -4.857047 | 3.449384 | 3.203000 |
| H | -4.588873 | 4.616996 | 1.412058 |
| H | -4.933946 | 2.044707 | 4.833268 |
| H | -5.493233 | 4.182495 | 3.690145 |

**I6**

ΔG = -3036.474766 a.u.

| | | | |
|---|---|---|---|
| Cu | 0.392622 | 0.635534 | 0.717304 |
| C | -3.766573 | -1.509654 | 1.612419 |
| H | -3.295832 | -2.499930 | 1.583414 |
| H | -4.089199 | -1.344194 | 2.649625 |
| C | -2.705576 | -0.423095 | 1.243476 |
| H | -1.885025 | -0.520964 | 1.962680 |
| P | 1.899206 | 1.633045 | -0.949866 |
| P | 1.569011 | -1.122622 | 1.746576 |
| B | -5.028648 | -1.510569 | 0.674821 |
| O | -6.050564 | -0.598594 | 0.770353 |
| O | -5.224184 | -2.413705 | -0.343833 |
| C | -6.840919 | -0.720465 | -0.433547 |
| C | -6.559254 | -2.200077 | -0.859918 |
| C | 5.211520 | 0.703130 | 3.950706 |
| C | 2.955035 | 0.856352 | 3.114482 |
| C | 4.243320 | -1.151545 | 2.746950 |
| C | 5.320569 | -0.579424 | 3.418372 |
| C | 4.025353 | 1.418071 | 3.803496 |
| H | 4.331986 | -2.157519 | 2.347335 |
| H | 6.244183 | -1.139373 | 3.530233 |
| H | 6.051699 | 1.147299 | 4.476000 |
| C | 3.309691 | 2.393452 | -0.036614 |
| C | 5.322122 | 3.609641 | 1.484522 |
| C | 3.019262 | 3.497802 | 0.775820 |
| C | 4.613788 | 1.896476 | -0.064735 |
| C | 5.612764 | 2.500622 | 0.695981 |
| C | 4.020885 | 4.107748 | 1.522380 |
| H | 4.866037 | 1.043915 | -0.685913 |
| H | 6.622523 | 2.102700 | 0.665372 |
| H | 6.103983 | 4.081114 | 2.072127 |
| C | 2.679946 | 0.652260 | -2.300299 |
| C | 3.662056 | -1.115602 | -4.248736 |
| C | 2.628065 | 0.987867 | -3.656393 |
| C | 3.227561 | -0.588117 | -1.968336 |
| C | 3.741370 | -1.482614 | -2.906861 |
| C | 3.108246 | 0.107349 | -4.619852 |
| H | 2.190784 | 1.931092 | -3.964289 |
| H | 3.052702 | 0.377146 | -5.669514 |
| H | 4.035749 | -1.783462 | -5.017271 |
| C | 2.256101 | -2.538535 | 0.784765 |
| C | 3.254518 | -4.560986 | -0.886737 |
| C | 2.031275 | -3.884020 | 1.084674 |
| C | 3.009621 | -2.252943 | -0.354112 |
| C | 3.526923 | -3.230942 | -1.202177 |
| C | 2.517245 | -4.884779 | 0.249135 |
| H | 1.460779 | -4.154175 | 1.966209 |

| | | | |
|---|---|---|---|
| H | 2.324304 | -5.925520 | 0.488438 |
| H | 3.624726 | -5.356874 | -1.523636 |
| C | 0.664865 | -1.978297 | 3.106484 |
| C | -0.851228 | -3.228102 | 5.101911 |
| C | -0.525578 | -2.643953 | 2.783158 |
| C | 1.086525 | -1.946526 | 4.437734 |
| C | 0.327664 | -2.565078 | 5.430188 |
| C | -1.274091 | -3.270074 | 3.774251 |
| H | -0.861025 | -2.670444 | 1.749713 |
| H | 2.005784 | -1.437199 | 4.709035 |
| H | 0.663956 | -2.529253 | 6.462036 |
| H | -2.193353 | -3.783800 | 3.509234 |
| H | -1.439797 | -3.709495 | 5.877075 |
| C | 1.227544 | 3.098523 | -1.840651 |
| C | 0.130429 | 5.299708 | -3.175428 |
| C | 2.061019 | 4.051221 | -2.442760 |
| C | -0.157406 | 3.268306 | -1.904617 |
| C | -0.704064 | 4.364256 | -2.572081 |
| C | 1.515399 | 5.143118 | -3.108411 |
| H | 3.140170 | 3.937104 | -2.387894 |
| H | -0.810430 | 2.557619 | -1.407176 |
| H | -1.782095 | 4.488804 | -2.603861 |
| H | 2.169988 | 5.874955 | -3.571925 |
| H | -0.294664 | 6.155472 | -3.691317 |
| H | 2.035960 | 1.422909 | 2.983567 |
| H | 2.006668 | 3.891907 | 0.815204 |
| H | 3.937912 | 2.421275 | 4.207858 |
| H | 3.782759 | 4.969697 | 2.138350 |
| O | 3.227285 | -0.925466 | -0.635328 |
| C | 4.379365 | -2.766758 | -2.381243 |
| C | 3.052972 | -0.436551 | 2.588807 |
| C | -0.965176 | 2.011445 | 1.158914 |
| C | 4.495156 | -3.838366 | -3.466506 |
| H | 5.122320 | -3.484917 | -4.289156 |
| H | 4.976278 | -4.736386 | -3.070226 |
| H | 3.515698 | -4.115576 | -3.868582 |
| C | 5.794225 | -2.428170 | -1.854589 |
| H | 6.266594 | -3.326019 | -1.443167 |
| H | 6.418653 | -2.046439 | -2.668759 |
| H | 5.757386 | -1.670728 | -1.065714 |
| C | -6.566238 | -2.441887 | -2.360391 |
| H | -6.302054 | -3.483002 | -2.569250 |
| H | -7.564957 | -2.256645 | -2.768800 |
| H | -5.856490 | -1.796100 | -2.878999 |
| C | -7.469545 | -3.210626 | -0.161816 |
| H | -7.088377 | -4.219203 | -0.345516 |
| H | -7.489727 | -3.046471 | 0.919788 |
| H | -8.493773 | -3.155831 | -0.541534 |
| C | -8.294441 | -0.415319 | -0.108682 |
| H | -8.662440 | -1.031510 | 0.714338 |
| H | -8.395742 | 0.634896 | 0.180108 |
| H | -8.926235 | -0.588516 | -0.985774 |
| C | -6.288304 | 0.303232 | -1.426772 |
| H | -5.241021 | 0.093602 | -1.667723 |
| H | -6.872108 | 0.319599 | -2.351957 |
| H | -6.338948 | 1.297279 | -0.972827 |
| B | -2.149473 | -0.795291 | -0.177138 |
| O | -2.712109 | -0.404063 | -1.366097 |
| O | -1.155290 | -1.735751 | -0.357186 |
| C | -1.964336 | -1.019456 | -2.440694 |

| | | | |
|---|---|---|---|
| C | -1.250094 | -2.214424 | -1.717515 |
| C | -2.933302 | -1.434428 | -3.535411 |
| H | -2.402474 | -1.955011 | -4.338995 |
| H | -3.712277 | -2.091526 | -3.146782 |
| H | -3.410515 | -0.547328 | -3.962815 |
| C | -0.986411 | 0.026981 | -2.966461 |
| H | -0.411696 | -0.349503 | -3.817375 |
| H | -1.542090 | 0.912539 | -3.286507 |
| H | -0.288104 | 0.329111 | -2.182118 |
| C | 0.146564 | -2.523160 | -2.227751 |
| H | 0.119014 | -2.806332 | -3.284955 |
| H | 0.805828 | -1.661235 | -2.116541 |
| H | 0.572152 | -3.356274 | -1.661080 |
| C | -2.095687 | -3.486866 | -1.669460 |
| H | -2.166592 | -3.956211 | -2.655237 |
| H | -1.619552 | -4.197305 | -0.986805 |
| H | -3.106520 | -3.280987 | -1.303179 |
| C | -2.303966 | 2.145768 | 1.299022 |
| C | -3.284955 | 0.984654 | 1.368982 |
| H | -3.815674 | 1.031564 | 2.331024 |
| H | -4.069317 | 1.132460 | 0.614340 |
| H | -0.468485 | 2.994748 | 1.177813 |
| C | -2.960475 | 3.495689 | 1.382204 |
| C | -2.388731 | 4.618103 | 0.757834 |
| C | -4.180771 | 3.697337 | 2.048859 |
| C | -2.982285 | 5.873891 | 0.818973 |
| H | -1.467011 | 4.500345 | 0.197526 |
| C | -4.778083 | 4.954481 | 2.115619 |
| H | -4.676373 | 2.865815 | 2.538751 |
| C | -4.183205 | 6.053603 | 1.503198 |
| H | -2.508575 | 6.713699 | 0.317487 |
| H | -5.716278 | 5.071705 | 2.651281 |
| H | -4.651145 | 7.032532 | 1.550476 |

**I7**

ΔG = -1275.739208 a.u.

| | | | |
|---|---|---|---|
| C | -0.910487 | -1.275607 | -0.985572 |
| H | -0.720911 | -0.957730 | -2.018088 |
| H | -0.641421 | -2.337049 | -0.937148 |
| C | 0.020046 | -0.471441 | -0.041358 |
| H | -0.207818 | -0.812705 | 0.984253 |
| B | -2.408276 | -1.054663 | -0.596432 |
| O | -3.225428 | -0.110359 | -1.178925 |
| O | -3.033428 | -1.710628 | 0.442141 |
| C | -4.372936 | 0.055336 | -0.320273 |
| C | -4.422294 | -1.317320 | 0.433599 |
| C | 1.095658 | 1.743169 | -0.319717 |
| C | 2.113777 | 0.879949 | -0.494984 |
| C | -5.190196 | -2.393625 | -0.334750 |
| H | -5.013410 | -3.363252 | 0.139538 |
| H | -6.266723 | -2.199494 | -0.329181 |
| H | -4.852452 | -2.457338 | -1.373551 |
| C | -4.927536 | -1.239495 | 1.866742 |
| H | -4.931656 | -2.238575 | 2.312870 |
| H | -4.296786 | -0.595066 | 2.482848 |
| H | -5.951665 | -0.853327 | 1.891512 |
| C | -4.066672 | 1.233464 | 0.606708 |
| H | -3.205406 | 1.019097 | 1.247130 |
| H | -3.827197 | 2.110722 | -0.001344 |
| H | -4.922068 | 1.478129 | 1.243257 |

| | | | |
|---|---|---|---|
| C | -5.595388 | 0.362751 | -1.171501 |
| H | -5.741470 | -0.386822 | -1.952013 |
| H | -6.495743 | 0.401114 | -0.549802 |
| H | -5.474856 | 1.337191 | -1.654279 |
| B | 1.652622 | -0.679123 | -0.340257 |
| O | 1.939872 | -1.588555 | -1.455095 |
| O | 2.406975 | -1.300063 | 0.827428 |
| C | 3.003993 | -2.440133 | -1.103571 |
| C | 2.836723 | -2.591276 | 0.446933 |
| C | 2.873001 | -3.749180 | -1.878984 |
| H | 3.611893 | -4.487215 | -1.546116 |
| H | 1.874032 | -4.176415 | -1.762316 |
| H | 3.036269 | -3.567319 | -2.946569 |
| C | 4.338321 | -1.768671 | -1.468043 |
| H | 5.194927 | -2.437342 | -1.328257 |
| H | 4.304221 | -1.471669 | -2.521008 |
| H | 4.499012 | -0.868660 | -0.867249 |
| C | 4.123013 | -2.937999 | 1.193138 |
| H | 4.872721 | -2.151227 | 1.078808 |
| H | 3.917143 | -3.057864 | 2.263186 |
| H | 4.549874 | -3.879813 | 0.830073 |
| C | 1.748291 | -3.609092 | 0.815701 |
| H | 2.062581 | -4.641458 | 0.627517 |
| H | 1.519277 | -3.514382 | 1.882993 |
| H | 0.830652 | -3.416228 | 0.253732 |
| C | -0.248195 | 1.056700 | -0.129998 |
| H | -0.786826 | 1.464685 | 0.740375 |
| H | -0.905773 | 1.262787 | -0.985733 |
| H | 3.122051 | 1.250382 | -0.701690 |
| C | 1.174139 | 3.225702 | -0.313811 |
| C | 0.029074 | 4.009239 | -0.520047 |
| C | 2.386831 | 3.892625 | -0.075411 |
| C | 0.096023 | 5.400570 | -0.509101 |
| H | -0.926314 | 3.526919 | -0.703053 |
| C | 2.455846 | 5.281037 | -0.062799 |
| H | 3.286895 | 3.312608 | 0.109355 |
| C | 1.309093 | 6.044224 | -0.280869 |
| H | -0.805184 | 5.982440 | -0.680364 |
| H | 3.407564 | 5.770642 | 0.123704 |
| H | 1.361729 | 7.128767 | -0.268920 |
| K | 1.809285 | 0.411878 | 2.547495 |

## I8

ΔG = -1258.954918 a.u.

| | | | |
|---|---|---|---|
| C | 1.346328 | 1.302363 | -1.302198 |
| H | 1.005298 | 0.987747 | -2.297090 |
| H | 1.374852 | 2.401555 | -1.337252 |
| C | 0.354051 | 0.839283 | -0.209365 |
| H | 0.766708 | 1.152829 | 0.756136 |
| B | 2.816279 | 0.801642 | -1.051601 |
| O | 3.792555 | 0.741244 | -2.011932 |
| O | 3.259851 | 0.388122 | 0.181240 |
| C | 4.917165 | 0.029763 | -1.440847 |
| C | 4.693868 | 0.228484 | 0.099072 |
| C | -0.655017 | -1.232820 | 0.884655 |
| C | -1.689389 | -2.064256 | 0.714464 |
| C | 5.313863 | 1.517442 | 0.637415 |
| H | 4.950170 | 1.687811 | 1.654613 |
| H | 6.405213 | 1.453449 | 0.668034 |
| H | 5.032256 | 2.381086 | 0.027461 |

| | | | |
|---|---|---|---|
| C | 5.105412 | -0.953049 | 0.963865 |
| H | 4.913106 | -0.724042 | 2.016403 |
| H | 4.548180 | -1.855658 | 0.704435 |
| H | 6.174946 | -1.156986 | 0.851608 |
| C | 4.787113 | -1.427779 | -1.882683 |
| H | 3.865761 | -1.879765 | -1.502656 |
| H | 4.757598 | -1.466740 | -2.975121 |
| H | 5.635764 | -2.026477 | -1.539969 |
| C | 6.207713 | 0.627550 | -1.979089 |
| H | 6.252644 | 1.705905 | -1.814305 |
| H | 7.073477 | 0.162785 | -1.496763 |
| H | 6.279714 | 0.443754 | -3.055010 |
| B | -1.046622 | 1.533798 | -0.377167 |
| O | -2.039710 | 1.048808 | -1.188545 |
| O | -1.400792 | 2.693191 | 0.264807 |
| C | -3.240536 | 1.787458 | -0.872542 |
| C | -2.660214 | 3.126138 | -0.301523 |
| C | -4.078808 | 1.936547 | -2.131966 |
| H | -4.951822 | 2.567891 | -1.938140 |
| H | -3.503562 | 2.376008 | -2.949437 |
| H | -4.434708 | 0.953988 | -2.455583 |
| C | -3.998584 | 0.970712 | 0.174963 |
| H | -4.959517 | 1.430916 | 0.422629 |
| H | -4.186198 | -0.030141 | -0.223765 |
| H | -3.413868 | 0.865659 | 1.094169 |
| C | -3.498592 | 3.770335 | 0.791439 |
| H | -3.607841 | 3.111799 | 1.655485 |
| H | -3.021936 | 4.695967 | 1.127250 |
| H | -4.494636 | 4.019863 | 0.412028 |
| C | -2.331604 | 4.148141 | -1.389430 |
| H | -3.239653 | 4.575773 | -1.824105 |
| H | -1.745879 | 4.960133 | -0.949350 |
| H | -1.739491 | 3.697932 | -2.192140 |
| C | 0.223998 | -0.696614 | -0.217193 |
| H | 1.220056 | -1.147272 | -0.114570 |
| H | -0.167227 | -1.021576 | -1.184800 |
| H | -2.261999 | -2.462690 | 1.543318 |
| C | -0.330802 | -0.814656 | 2.281983 |
| C | -1.344566 | -0.539263 | 3.208332 |
| C | 1.003507 | -0.673815 | 2.689657 |
| C | -1.036000 | -0.165971 | 4.513284 |
| H | -2.383865 | -0.598603 | 2.898339 |
| C | 1.310059 | -0.300763 | 3.994003 |
| H | 1.809446 | -0.836741 | 1.980320 |
| C | 0.292780 | -0.050587 | 4.912989 |
| H | -1.837296 | 0.046729 | 5.214589 |
| H | 2.349790 | -0.200217 | 4.291244 |
| H | 0.534464 | 0.245565 | 5.929232 |
| I | -2.394225 | -2.818004 | -1.114220 |

**I4-A**

ΔG = -3036.470558 a.u.

| | | | |
|---|---|---|---|
| Cu | -0.028658 | 0.204135 | -0.105990 |
| C | 2.169734 | 1.340796 | -1.912471 |
| H | 2.126457 | 2.411278 | -1.670459 |
| H | 3.171460 | 1.165557 | -2.341358 |
| C | 1.900719 | 0.506995 | -0.630993 |
| H | 2.273662 | -0.512334 | -0.824743 |
| P | -0.883438 | -1.978928 | 0.077288 |
| P | -1.416514 | 1.718882 | 1.027534 |

| | | | |
|---|---|---|---|
| B | 1.005969 | 1.001681 | -2.896189 |
| O | -0.015053 | 1.874620 | -3.219566 |
| O | 0.824979 | -0.230844 | -3.490648 |
| C | -1.073120 | 1.120972 | -3.845509 |
| C | -0.321538 | -0.161138 | -4.360909 |
| C | -1.679279 | 0.812841 | 5.561917 |
| C | -0.434071 | 0.946521 | 3.500575 |
| C | -2.780885 | 1.508943 | 3.529402 |
| C | -2.828516 | 1.224651 | 4.892414 |
| C | -0.480518 | 0.678302 | 4.864414 |
| H | -3.680330 | 1.839392 | 3.018226 |
| H | -3.765420 | 1.330150 | 5.431150 |
| H | -1.717608 | 0.592760 | 6.624530 |
| C | -0.773840 | -2.349041 | 1.873292 |
| C | -0.409097 | -2.802536 | 4.607114 |
| C | -1.869703 | -2.624633 | 2.690539 |
| C | 0.509228 | -2.283484 | 2.436815 |
| C | 0.689155 | -2.522948 | 3.794110 |
| C | -1.686010 | -2.843858 | 4.055301 |
| H | 1.370617 | -2.046902 | 1.814065 |
| H | 1.687175 | -2.476265 | 4.220188 |
| H | -0.269631 | -2.978921 | 5.669400 |
| C | -2.595131 | -2.406982 | -0.455764 |
| C | -5.164263 | -2.702941 | -1.552195 |
| C | -2.903100 | -3.504758 | -1.265915 |
| C | -3.618367 | -1.486999 | -0.207345 |
| C | -4.905084 | -1.604475 | -0.734128 |
| C | -4.174846 | -3.648204 | -1.809785 |
| H | -2.137794 | -4.238163 | -1.495126 |
| H | -4.395773 | -4.500683 | -2.443900 |
| H | -6.147147 | -2.831246 | -1.992377 |
| C | -3.150478 | 1.935084 | 0.462349 |
| C | -5.737492 | 2.024778 | -0.609697 |
| C | -3.731404 | 3.158296 | 0.122988 |
| C | -3.905179 | 0.779104 | 0.257476 |
| C | -5.199229 | 0.786875 | -0.258795 |
| C | -5.012483 | 3.198952 | -0.419385 |
| H | -3.173574 | 4.078270 | 0.265500 |
| H | -5.452138 | 4.152962 | -0.691938 |
| H | -6.736148 | 2.082378 | -1.028510 |
| C | -0.738256 | 3.425342 | 0.967532 |
| C | 0.407596 | 5.962459 | 0.733684 |
| C | -0.072541 | 3.794684 | -0.207226 |
| C | -0.829549 | 4.337122 | 2.022695 |
| C | -0.255362 | 5.601185 | 1.904946 |
| C | 0.495557 | 5.059497 | -0.324182 |
| H | 0.004707 | 3.087257 | -1.029385 |
| H | -1.342747 | 4.060176 | 2.939221 |
| H | -0.324670 | 6.303250 | 2.730517 |
| H | 1.023670 | 5.330767 | -1.233074 |
| H | 0.861586 | 6.945241 | 0.647248 |
| C | 0.122686 | -3.336491 | -0.648492 |
| C | 1.643048 | -5.344822 | -1.860907 |
| C | 0.118298 | -4.637120 | -0.129106 |
| C | 0.905401 | -3.046432 | -1.769287 |
| C | 1.657720 | -4.051915 | -2.376478 |
| C | 0.874437 | -5.636245 | -0.733360 |
| H | -0.478668 | -4.868323 | 0.748837 |
| H | 0.924584 | -2.037260 | -2.172389 |
| H | 2.262310 | -3.817065 | -3.247408 |

| | | | |
|---|---|---|---|
| H | 0.865791 | -6.642139 | -0.324682 |
| H | 2.234226 | -6.125152 | -2.330931 |
| H | 0.499273 | 0.823518 | 2.956442 |
| H | -2.869123 | -2.677356 | 2.268888 |
| H | 0.416852 | 0.348754 | 5.378505 |
| H | -2.545174 | -3.053718 | 4.685208 |
| O | -3.307679 | -0.412476 | 0.589965 |
| C | -5.935748 | -0.549525 | -0.333060 |
| C | 2.675555 | 1.067907 | 0.585688 |
| H | 2.429390 | 2.128322 | 0.703105 |
| H | 2.349263 | 0.561038 | 1.503442 |
| C | -1.582676 | 1.371876 | 2.823998 |
| C | 5.054652 | 1.744625 | -0.014453 |
| C | 4.156994 | 0.830837 | 0.403399 |
| C | -7.122321 | -0.503043 | -1.297705 |
| H | -7.647038 | -1.462071 | -1.309104 |
| H | -7.850081 | 0.245936 | -0.974069 |
| H | -6.807439 | -0.265101 | -2.318411 |
| C | -6.454879 | -0.892643 | 1.083946 |
| H | -7.170192 | -0.133571 | 1.416640 |
| H | -6.956534 | -1.865702 | 1.075550 |
| H | -5.638116 | -0.934288 | 1.810927 |
| C | 0.215038 | -0.017622 | -5.784905 |
| H | 0.865680 | -0.868651 | -6.006020 |
| H | -0.593443 | -0.002640 | -6.521531 |
| H | 0.805715 | 0.896681 | -5.894949 |
| C | -1.120449 | -1.451653 | -4.232089 |
| H | -0.520772 | -2.295930 | -4.585417 |
| H | -1.406548 | -1.651226 | -3.196894 |
| H | -2.029707 | -1.402124 | -4.839912 |
| C | -2.116283 | 0.820955 | -2.768663 |
| H | -1.693090 | 0.210358 | -1.963809 |
| H | -2.460667 | 1.763404 | -2.334024 |
| H | -2.983006 | 0.294792 | -3.179615 |
| C | -1.702276 | 1.972932 | -4.939167 |
| H | -0.956797 | 2.335017 | -5.650083 |
| H | -2.458894 | 1.400475 | -5.485265 |
| H | -2.193882 | 2.841055 | -4.489991 |
| C | 5.582952 | -2.652168 | 0.107201 |
| C | 4.542186 | -2.821325 | 1.267446 |
| B | 4.583644 | -0.664751 | 0.536945 |
| O | 5.737821 | -1.215877 | 0.036117 |
| O | 3.797771 | -1.587556 | 1.187943 |
| C | 5.036179 | -3.101417 | -1.248601 |
| H | 5.726261 | -2.780777 | -2.034155 |
| H | 4.933394 | -4.189165 | -1.300463 |
| H | 4.059058 | -2.651404 | -1.448066 |
| C | 6.939127 | -3.287789 | 0.368287 |
| H | 6.832957 | -4.368023 | 0.510574 |
| H | 7.597155 | -3.121806 | -0.489705 |
| H | 7.419827 | -2.864215 | 1.252558 |
| C | 5.185489 | -2.867960 | 2.652596 |
| H | 4.401708 | -2.783210 | 3.410886 |
| H | 5.717976 | -3.809660 | 2.814520 |
| H | 5.887102 | -2.040557 | 2.795397 |
| C | 3.579123 | -3.984482 | 1.088355 |
| H | 4.125699 | -4.930088 | 1.011425 |
| H | 2.906839 | -4.050749 | 1.949006 |
| H | 2.970728 | -3.854947 | 0.192271 |
| H | 6.055446 | 1.403579 | -0.284262 |

| | | | |
|---|---|---|---|
| C | 4.816594 | 3.195409 | -0.199204 |
| C | 5.201858 | 3.817876 | -1.394188 |
| C | 4.224164 | 3.982650 | 0.797020 |
| C | 4.971854 | 5.174954 | -1.600955 |
| H | 5.671190 | 3.223226 | -2.173821 |
| C | 3.999919 | 5.341516 | 0.595273 |
| H | 3.947394 | 3.524878 | 1.741937 |
| C | 4.366348 | 5.942521 | -0.607130 |
| H | 5.266451 | 5.634680 | -2.539980 |
| H | 3.535888 | 5.930772 | 1.380614 |
| H | 4.188387 | 7.001799 | -0.766165 |

**TS2-A**

ΔG = -3036.439578 a.u.

| | | | |
|---|---|---|---|
| Cu | 0.158654 | 0.629706 | 0.285848 |
| C | 0.669352 | 1.659256 | 3.142341 |
| H | 1.328508 | 1.746340 | 4.023136 |
| H | 0.412750 | 2.688201 | 2.863648 |
| C | 1.402370 | 0.926048 | 2.000562 |
| H | 1.051028 | -0.116142 | 2.017016 |
| P | -1.929179 | 1.250279 | -0.679473 |
| P | 0.753505 | -1.681351 | -0.517005 |
| B | -0.586001 | 0.832094 | 3.581954 |
| O | -0.512265 | -0.513711 | 3.878393 |
| O | -1.875940 | 1.301246 | 3.654731 |
| C | -1.856136 | -1.036766 | 3.864270 |
| C | -2.710099 | 0.240780 | 4.177206 |
| C | 2.559661 | -1.405760 | -4.772622 |
| C | 2.288335 | -0.565598 | -2.528023 |
| C | 1.143365 | -2.580030 | -3.206329 |
| C | 1.697240 | -2.459131 | -4.479665 |
| C | 2.858122 | -0.460300 | -3.792231 |
| H | 0.482506 | -3.412685 | -2.984444 |
| H | 1.458192 | -3.195029 | -5.241444 |
| H | 2.993821 | -1.318315 | -5.764117 |
| C | -1.753080 | 1.237430 | -2.514795 |
| C | -1.379686 | 1.127866 | -5.284694 |
| C | -0.467033 | 1.312996 | -3.058286 |
| C | -2.851626 | 1.103753 | -3.372020 |
| C | -2.664869 | 1.052508 | -4.750063 |
| C | -0.283012 | 1.259849 | -4.437885 |
| H | -3.855953 | 1.030823 | -2.964642 |
| H | -3.523469 | 0.947231 | -5.406309 |
| H | -1.234586 | 1.078652 | -6.359696 |
| C | -3.438750 | 0.208747 | -0.474247 |
| C | -5.625155 | -1.527774 | -0.234304 |
| C | -4.681453 | 0.689860 | -0.057874 |
| C | -3.339203 | -1.148733 | -0.777265 |
| C | -4.407786 | -2.037832 | -0.685408 |
| C | -5.761970 | -0.177548 | 0.074617 |
| H | -4.803732 | 1.740350 | 0.182038 |
| H | -6.719058 | 0.204113 | 0.414711 |
| H | -6.483459 | -2.183273 | -0.136124 |
| C | -0.426151 | -3.103654 | -0.548597 |
| C | -2.353438 | -5.138521 | -0.411451 |
| C | -0.059438 | -4.412994 | -0.218634 |
| C | -1.770000 | -2.868279 | -0.838816 |
| C | -2.755641 | -3.855070 | -0.774295 |
| C | -1.015169 | -5.419147 | -0.147579 |
| H | 0.977045 | -4.642208 | 0.003390 |

| | | | |
|---|---|---|---|
| H | -0.717351 | -6.428066 | 0.118801 |
| H | -3.085084 | -5.936010 | -0.341559 |
| C | 2.109062 | -2.435539 | 0.478141 |
| C | 4.041493 | -3.639531 | 2.107173 |
| C | 1.900545 | -2.557274 | 1.858738 |
| C | 3.292285 | -2.927116 | -0.075469 |
| C | 4.251000 | -3.529752 | 0.736515 |
| C | 2.861305 | -3.153970 | 2.667622 |
| H | 0.985676 | -2.182075 | 2.311190 |
| H | 3.472439 | -2.843122 | -1.142551 |
| H | 5.171416 | -3.898356 | 0.295379 |
| H | 2.688801 | -3.233669 | 3.736705 |
| H | 4.797260 | -4.097758 | 2.737838 |
| C | -2.603603 | 2.905254 | -0.246838 |
| C | -3.537082 | 5.408091 | 0.572538 |
| C | -3.118712 | 3.803345 | -1.183605 |
| C | -2.567282 | 3.263700 | 1.104952 |
| C | -3.038594 | 4.508205 | 1.512475 |
| C | -3.577517 | 5.053123 | -0.773926 |
| H | -3.146219 | 3.541159 | -2.236744 |
| H | -2.172413 | 2.571025 | 1.843767 |
| H | -3.005015 | 4.776303 | 2.564134 |
| H | -3.964998 | 5.751091 | -1.509944 |
| H | -3.892037 | 6.384553 | 0.888108 |
| H | 2.497267 | 0.191198 | -1.777645 |
| H | 0.394087 | 1.423105 | -2.403906 |
| H | 3.521477 | 0.370507 | -4.014774 |
| H | 0.722028 | 1.306507 | -4.845471 |
| O | -2.109665 | -1.588402 | -1.194829 |
| C | -4.181347 | -3.469908 | -1.163964 |
| C | 2.938795 | 0.793510 | 2.143258 |
| H | 3.315338 | 1.510262 | 2.886762 |
| H | 3.254558 | -0.207123 | 2.466504 |
| C | 1.436614 | -1.634129 | -2.222032 |
| C | 4.425121 | 0.764710 | 0.009374 |
| C | 3.406413 | 1.189142 | 0.774501 |
| C | -5.217473 | -4.439613 | -0.591046 |
| H | -6.226139 | -4.147955 | -0.895178 |
| H | -5.056014 | -5.448993 | -0.978282 |
| H | -5.179294 | -4.474297 | 0.502146 |
| C | -4.282076 | -3.479510 | -2.708272 |
| H | -4.083458 | -4.485413 | -3.092061 |
| H | -5.286778 | -3.177617 | -3.021656 |
| H | -3.561631 | -2.791373 | -3.160807 |
| C | -2.876107 | 0.502739 | 5.672974 |
| H | -3.301711 | 1.500330 | 5.813755 |
| H | -3.549527 | -0.225964 | 6.133133 |
| H | -1.914161 | 0.466519 | 6.193009 |
| C | -4.056606 | 0.290358 | 3.475833 |
| H | -4.575143 | 1.219839 | 3.729812 |
| H | -3.940985 | 0.250396 | 2.392482 |
| H | -4.685815 | -0.548573 | 3.790113 |
| C | -2.095299 | -1.593298 | 2.459275 |
| H | -1.967612 | -0.812981 | 1.702491 |
| H | -1.366461 | -2.383578 | 2.256004 |
| H | -3.097175 | -2.018842 | 2.352480 |
| C | -1.972083 | -2.149907 | 4.893493 |
| H | -1.657448 | -1.818407 | 5.885081 |
| H | -3.004556 | -2.508659 | 4.954224 |
| H | -1.337979 | -2.991229 | 4.598186 |

| | | | |
|---|---|---|---|
| H | 4.463945 | 1.147390 | -1.012801 |
| C | 5.502294 | -0.181681 | 0.339803 |
| C | 5.920925 | -0.442072 | 1.654062 |
| C | 6.172097 | -0.843519 | -0.702761 |
| C | 6.944373 | -1.349602 | 1.913288 |
| H | 5.456151 | 0.085751 | 2.479622 |
| C | 7.199068 | -1.745133 | -0.446033 |
| H | 5.868859 | -0.648850 | -1.729011 |
| C | 7.587816 | -2.007892 | 0.867380 |
| H | 7.248347 | -1.534241 | 2.939889 |
| H | 7.697492 | -2.244973 | -1.271974 |
| B | 2.245786 | 2.202040 | 0.414754 |
| O | 2.218402 | 3.437485 | 1.128466 |
| O | 1.897141 | 2.466531 | -0.943339 |
| C | 2.433256 | 4.462934 | 0.163567 |
| C | 1.720935 | 3.884161 | -1.107527 |
| C | 1.838690 | 5.764545 | 0.688804 |
| H | 1.890066 | 6.553823 | -0.068950 |
| H | 0.797116 | 5.634917 | 0.990609 |
| H | 2.405101 | 6.099013 | 1.563684 |
| C | 3.944493 | 4.639271 | -0.034970 |
| H | 4.172027 | 5.470179 | -0.710441 |
| H | 4.404358 | 4.848920 | 0.935463 |
| H | 4.404954 | 3.730098 | -0.431818 |
| C | 2.346155 | 4.283141 | -2.438256 |
| H | 3.380711 | 3.940400 | -2.513909 |
| H | 1.778192 | 3.833235 | -3.259604 |
| H | 2.326000 | 5.370410 | -2.569611 |
| C | 0.234043 | 4.208876 | -1.115594 |
| H | 0.052538 | 5.274541 | -1.286426 |
| H | -0.259233 | 3.655538 | -1.916914 |
| H | -0.222650 | 3.926420 | -0.166146 |
| H | 8.390428 | -2.710266 | 1.072016 |

**I4-B**

ΔG = -3075.743631 a.u.

| | | | |
|---|---|---|---|
| Cu | 0.759351 | -0.678791 | -0.685633 |
| C | 1.368392 | -1.802053 | -3.377714 |
| H | 1.408981 | -1.651329 | -4.473085 |
| H | 2.349524 | -2.197148 | -3.081181 |
| C | 1.087508 | -0.463926 | -2.638567 |
| H | 0.064930 | -0.163459 | -2.932450 |
| P | 0.325266 | -1.304917 | 1.481074 |
| P | -1.527538 | 1.841904 | -0.683804 |
| B | 0.231946 | -2.810793 | -3.019256 |
| O | -1.015231 | -2.772231 | -3.601972 |
| O | 0.309080 | -3.773254 | -2.032936 |
| C | -1.906266 | -3.535128 | -2.763231 |
| C | -0.925321 | -4.524175 | -2.045386 |
| C | -2.442097 | 4.437045 | 3.065193 |
| C | -0.903644 | 3.236681 | 1.645186 |
| C | -3.252901 | 3.276580 | 1.110248 |
| C | -3.497449 | 4.054913 | 2.237089 |
| C | -1.146488 | 4.028373 | 2.766228 |
| H | -4.080576 | 2.989399 | 0.467861 |
| H | -4.512015 | 4.366709 | 2.467437 |
| H | -2.633176 | 5.047583 | 3.942858 |
| C | 0.617186 | 0.013385 | 2.721827 |
| C | 1.228769 | 2.027558 | 4.556263 |
| C | 1.813995 | 0.731218 | 2.606101 |

| | | | |
|---|---|---|---|
| C | -0.272528 | 0.314913 | 3.753775 |
| C | 0.031932 | 1.324735 | 4.664410 |
| C | 2.120502 | 1.726772 | 3.527458 |
| H | -1.202786 | -0.235821 | 3.854525 |
| H | -0.668978 | 1.560345 | 5.459228 |
| H | 1.465971 | 2.810363 | 5.270490 |
| C | -1.263568 | -2.100576 | 1.983763 |
| C | -3.681558 | -3.438449 | 2.445576 |
| C | -1.285898 | -3.328155 | 2.654224 |
| C | -2.490715 | -1.544461 | 1.605714 |
| C | -3.709377 | -2.195681 | 1.815962 |
| C | -2.483084 | -3.994002 | 2.879863 |
| H | -0.356986 | -3.784896 | 2.974427 |
| H | -2.481005 | -4.952471 | 3.388250 |
| H | -4.606992 | -3.976373 | 2.617910 |
| C | -3.129190 | 0.935248 | -0.874209 |
| C | -5.591998 | -0.406722 | -0.862913 |
| C | -4.073335 | 1.171671 | -1.874517 |
| C | -3.442027 | -0.023907 | 0.092125 |
| C | -4.682414 | -0.655294 | 0.165250 |
| C | -5.281646 | 0.480403 | -1.888098 |
| H | -3.865759 | 1.903759 | -2.647534 |
| H | -5.999222 | 0.662367 | -2.681773 |
| H | -6.558818 | -0.897669 | -0.861324 |
| C | -1.671365 | 3.055439 | -2.073936 |
| C | -1.740287 | 4.787703 | -4.282178 |
| C | -1.255088 | 2.620950 | -3.340441 |
| C | -2.122659 | 4.370469 | -1.932135 |
| C | -2.155166 | 5.230245 | -3.028845 |
| C | -1.293758 | 3.476675 | -4.437048 |
| H | -0.892601 | 1.604195 | -3.468860 |
| H | -2.439760 | 4.735808 | -0.960873 |
| H | -2.504495 | 6.250617 | -2.899771 |
| H | -0.966247 | 3.120556 | -5.409106 |
| H | -1.762919 | 5.460790 | -5.133973 |
| C | 1.528708 | -2.616409 | 1.944530 |
| C | 3.267548 | -4.723031 | 2.531054 |
| C | 2.175581 | -2.668906 | 3.181648 |
| C | 1.759850 | -3.623751 | 1.000591 |
| C | 2.617777 | -4.677896 | 1.298948 |
| C | 3.048928 | -3.716230 | 3.468959 |
| H | 2.001307 | -1.895417 | 3.923409 |
| H | 1.273709 | -3.582028 | 0.027903 |
| H | 2.789755 | -5.455995 | 0.561227 |
| H | 3.554417 | -3.747027 | 4.429396 |
| H | 3.946267 | -5.539363 | 2.759140 |
| H | 0.107666 | 2.903948 | 1.432577 |
| H | 2.517134 | 0.501385 | 1.808524 |
| H | -0.318708 | 4.313307 | 3.407320 |
| H | 3.058569 | 2.266621 | 3.441414 |
| O | -2.460256 | -0.307612 | 1.017145 |
| C | -4.992187 | -1.477929 | 1.411056 |
| C | 2.109396 | 1.917447 | -2.352378 |
| C | -1.950317 | 2.863815 | 0.799308 |
| C | 2.893403 | 2.765652 | -0.081013 |
| C | 2.915281 | 1.843233 | -1.071180 |
| C | -6.153845 | -2.449740 | 1.189705 |
| H | -6.383632 | -2.995785 | 2.108195 |
| H | -7.061775 | -1.904979 | 0.918925 |
| H | -5.933091 | -3.173662 | 0.399065 |

| | | | |
|---|---|---|---|
| C | -5.371175 | -0.492856 | 2.543593 |
| H | -6.272599 | 0.065598 | 2.270854 |
| H | -5.565822 | -1.040796 | 3.471461 |
| H | -4.568732 | 0.227332 | 2.731488 |
| C | -0.662920 | -5.800547 | -2.843234 |
| H | 0.168825 | -6.342311 | -2.383554 |
| H | -1.538305 | -6.456541 | -2.848402 |
| H | -0.390441 | -5.573554 | -3.878333 |
| C | -1.313457 | -4.863812 | -0.614786 |
| H | -0.578809 | -5.547193 | -0.177622 |
| H | -1.357133 | -3.969173 | 0.007173 |
| H | -2.291880 | -5.354472 | -0.588156 |
| C | -2.558572 | -2.544097 | -1.800906 |
| H | -1.804045 | -2.043667 | -1.184215 |
| H | -3.084651 | -1.776466 | -2.375462 |
| H | -3.278798 | -3.033497 | -1.139688 |
| C | -2.964586 | -4.201824 | -3.628007 |
| H | -2.517608 | -4.796504 | -4.427470 |
| H | -3.600640 | -4.853168 | -3.019861 |
| H | -3.601051 | -3.438732 | -4.085727 |
| H | 3.549421 | 2.565027 | 0.765885 |
| C | 2.163043 | 4.033300 | 0.091837 |
| C | 1.336842 | 4.648524 | -0.862992 |
| C | 2.320595 | 4.683954 | 1.329887 |
| C | 0.680775 | 5.842268 | -0.573032 |
| H | 1.198181 | 4.209576 | -1.841629 |
| C | 1.664879 | 5.873073 | 1.619010 |
| H | 2.967354 | 4.235272 | 2.079016 |
| C | 0.831470 | 6.456221 | 0.666372 |
| H | 0.043396 | 6.290770 | -1.328500 |
| H | 1.802077 | 6.344022 | 2.588049 |
| B | 4.016790 | 0.753239 | -0.853215 |
| O | 4.798103 | 0.216737 | -1.846640 |
| O | 4.408657 | 0.337276 | 0.399873 |
| C | 5.931862 | -0.418711 | -1.216211 |
| C | 5.392724 | -0.708253 | 0.225244 |
| C | 6.308404 | -1.656479 | -2.015597 |
| H | 7.107065 | -2.209871 | -1.511343 |
| H | 5.452728 | -2.320789 | -2.151937 |
| H | 6.671764 | -1.360848 | -3.004141 |
| C | 7.076142 | 0.594588 | -1.238407 |
| H | 7.996557 | 0.166520 | -0.831038 |
| H | 7.264987 | 0.895445 | -2.272649 |
| H | 6.824716 | 1.491740 | -0.664494 |
| C | 6.428667 | -0.596808 | 1.332893 |
| H | 6.866942 | 0.402266 | 1.379116 |
| H | 5.958116 | -0.808984 | 2.297637 |
| H | 7.231338 | -1.325197 | 1.179743 |
| C | 4.649876 | -2.035237 | 0.329791 |
| H | 5.330214 | -2.886201 | 0.234472 |
| H | 4.166053 | -2.091363 | 1.306959 |
| H | 3.873846 | -2.115900 | -0.437318 |
| C | 2.032322 | 0.623835 | -3.174880 |
| H | 1.729952 | 0.904541 | -4.200122 |
| H | 3.040260 | 0.208912 | -3.275447 |
| H | 2.532320 | 2.718643 | -2.978035 |
| H | 1.088723 | 2.231583 | -2.113115 |
| H | 0.310575 | 7.383184 | 0.886089 |

**TS2-B**

ΔG = -3075.718406 a.u.

| | | | |
|---|---|---|---|
| Cu | -0.190951 | 0.647953 | -0.432284 |
| C | 0.205384 | 1.561980 | -3.151650 |
| H | -0.113853 | 1.824892 | -4.177667 |
| H | 0.520722 | 2.513538 | -2.706037 |
| C | -0.963123 | 0.887341 | -2.385287 |
| H | -0.746663 | -0.192574 | -2.358613 |
| P | 1.813291 | 1.413586 | 0.757306 |
| P | -0.728263 | -1.655475 | 0.389267 |
| B | 1.412705 | 0.580013 | -3.338626 |
| O | 1.243691 | -0.727166 | -3.745469 |
| O | 2.739773 | 0.882679 | -3.132721 |
| C | 2.503242 | -1.407022 | -3.569610 |
| C | 3.528264 | -0.224751 | -3.628844 |
| C | -2.958761 | -1.560449 | 4.452745 |
| C | -2.319690 | -0.537311 | 2.364075 |
| C | -1.528057 | -2.748884 | 2.911391 |
| C | -2.212146 | -2.690122 | 4.122668 |
| C | -3.013438 | -0.484743 | 3.570086 |
| H | -0.955408 | -3.637038 | 2.661991 |
| H | -2.164172 | -3.530588 | 4.808471 |
| H | -3.492760 | -1.518636 | 5.397302 |
| C | 1.443851 | 1.482596 | 2.564924 |
| C | 0.786011 | 1.580352 | 5.283863 |
| C | 0.112452 | 1.613205 | 2.967166 |
| C | 2.445219 | 1.397837 | 3.539836 |
| C | 2.116969 | 1.446393 | 4.890959 |
| C | -0.214278 | 1.665489 | 4.320535 |
| H | 3.485150 | 1.291953 | 3.245426 |
| H | 2.901811 | 1.376873 | 5.638061 |
| H | 0.531230 | 1.613159 | 6.338854 |
| C | 3.377377 | 0.428814 | 0.784719 |
| C | 5.648216 | -1.206989 | 0.942805 |
| C | 4.650146 | 0.960156 | 0.566444 |
| C | 3.289495 | -0.926239 | 1.105166 |
| C | 4.396658 | -1.767706 | 1.197902 |
| C | 5.775275 | 0.144818 | 0.638168 |
| H | 4.766165 | 2.011230 | 0.328238 |
| H | 6.757839 | 0.567062 | 0.454782 |
| H | 6.538717 | -1.823297 | 0.997811 |
| C | 0.509905 | -3.017115 | 0.560606 |
| C | 2.525318 | -4.961428 | 0.713187 |
| C | 0.246716 | -4.345250 | 0.210625 |
| C | 1.794298 | -2.713768 | 1.011073 |
| C | 2.820997 | -3.655456 | 1.099210 |
| C | 1.247518 | -5.307012 | 0.281259 |
| H | -0.742345 | -4.624155 | -0.136326 |
| H | 1.032043 | -6.332046 | -0.002368 |
| H | 3.292943 | -5.726142 | 0.756376 |
| C | -1.930515 | -2.471702 | -0.743267 |
| C | -3.654983 | -3.714478 | -2.565361 |
| C | -1.541240 | -2.658953 | -2.077369 |
| C | -3.188978 | -2.912234 | -0.335151 |
| C | -4.049138 | -3.526135 | -1.244653 |
| C | -2.395085 | -3.282586 | -2.979757 |
| H | -0.569014 | -2.312308 | -2.418049 |
| H | -3.511956 | -2.773621 | 0.691205 |
| H | -5.031716 | -3.847954 | -0.913758 |
| H | -2.080241 | -3.420550 | -4.009738 |
| H | -4.327386 | -4.191353 | -3.272112 |

| | | | |
|---|---|---|---|
| C | 2.495969 | 3.084961 | 0.373258 |
| C | 3.475792 | 5.602657 | -0.360178 |
| C | 2.724107 | 4.063811 | 1.343030 |
| C | 2.776891 | 3.373311 | -0.967197 |
| C | 3.267861 | 4.624038 | -1.329989 |
| C | 3.206298 | 5.318301 | 0.975946 |
| H | 2.511464 | 3.859824 | 2.387598 |
| H | 2.632071 | 2.612868 | -1.728808 |
| H | 3.482377 | 4.833540 | -2.373642 |
| H | 3.369500 | 6.074129 | 1.738282 |
| H | 3.848526 | 6.582065 | -0.644605 |
| H | -2.357877 | 0.307886 | 1.683844 |
| H | -0.673195 | 1.680220 | 2.220325 |
| H | -3.589451 | 0.401737 | 3.819999 |
| H | -1.254219 | 1.758284 | 4.617890 |
| O | 2.031273 | -1.408678 | 1.360182 |
| C | 4.165806 | -3.203060 | 1.664700 |
| C | -3.471917 | 0.392517 | -2.499725 |
| C | -1.577585 | -1.670979 | 2.021498 |
| C | -4.376703 | 0.598139 | -0.129088 |
| C | -3.507605 | 0.943473 | -1.098354 |
| C | 5.307492 | -4.133075 | 1.246337 |
| H | 6.254588 | -3.799482 | 1.678085 |
| H | 5.136923 | -5.146117 | 1.619732 |
| H | 5.416321 | -4.175504 | 0.158093 |
| C | 4.063625 | -3.192602 | 3.208718 |
| H | 3.857967 | -4.201974 | 3.579562 |
| H | 5.005175 | -2.844230 | 3.645663 |
| H | 3.262219 | -2.531317 | 3.551970 |
| C | 3.951310 | 0.136057 | -5.052119 |
| H | 4.499163 | 1.082456 | -5.030958 |
| H | 4.604792 | -0.629178 | -5.480673 |
| H | 3.083371 | 0.260037 | -5.706836 |
| C | 4.748407 | -0.404242 | -2.742838 |
| H | 5.385812 | 0.483044 | -2.801540 |
| H | 4.466478 | -0.551987 | -1.700768 |
| H | 5.334706 | -1.268034 | -3.072794 |
| C | 2.452601 | -2.079521 | -2.198546 |
| H | 2.283980 | -1.337332 | -1.412538 |
| H | 1.625458 | -2.793880 | -2.173204 |
| H | 3.374427 | -2.622916 | -1.973555 |
| C | 2.666544 | -2.450351 | -4.663258 |
| H | 2.557651 | -2.013904 | -5.658205 |
| H | 3.650092 | -2.926027 | -4.594049 |
| H | 1.905299 | -3.227562 | -4.546786 |
| H | -4.246026 | 1.085667 | 0.837497 |
| C | -5.471286 | -0.392124 | -0.140944 |
| C | -6.181984 | -0.773641 | -1.288677 |
| C | -5.835415 | -0.990834 | 1.077166 |
| C | -7.197903 | -1.723832 | -1.222015 |
| H | -5.954191 | -0.317070 | -2.243594 |
| C | -6.837074 | -1.952326 | 1.143837 |
| H | -5.303884 | -0.706561 | 1.981600 |
| C | -7.526266 | -2.325682 | -0.009742 |
| H | -7.734871 | -1.995531 | -2.126485 |
| H | -7.084842 | -2.407127 | 2.098839 |
| B | -2.445697 | 2.089158 | -0.822594 |
| O | -2.508581 | 3.276124 | -1.588585 |
| O | -2.140297 | 2.426808 | 0.520103 |
| C | -2.732337 | 4.351276 | -0.678141 |

| | | | |
|---|---|---|---|
| C | -1.996813 | 3.857774 | 0.611642 |
| C | -2.170944 | 5.632859 | -1.281084 |
| H | -2.234426 | 6.461615 | -0.567939 |
| H | -1.129204 | 5.508552 | -1.583925 |
| H | -2.751424 | 5.904786 | -2.168084 |
| C | -4.245594 | 4.499159 | -0.479094 |
| H | -4.490260 | 5.356838 | 0.155313 |
| H | -4.715528 | 4.648339 | -1.455700 |
| H | -4.679168 | 3.598917 | -0.033981 |
| C | -2.620239 | 4.310885 | 1.925113 |
| H | -3.645689 | 3.949282 | 2.029531 |
| H | -2.034031 | 3.921938 | 2.764382 |
| H | -2.624893 | 5.403770 | 1.995384 |
| C | -0.517079 | 4.209481 | 0.592765 |
| H | -0.356041 | 5.288745 | 0.670309 |
| H | -0.025943 | 3.743634 | 1.447166 |
| H | -0.041333 | 3.848814 | -0.322141 |
| C | -2.244028 | 0.944080 | -3.227133 |
| H | -2.099101 | 0.387800 | -4.167891 |
| H | -2.442256 | 1.982995 | -3.513207 |
| H | -4.374117 | 0.673716 | -3.057404 |
| H | -3.457090 | -0.704851 | -2.474511 |
| H | -8.315041 | -3.070597 | 0.038419 |

## - References


1. H. Jing, X. Feng, M. Guo, S. Zhou, Y. Li, J. Zhang, W. Zhao, X. Tang, G. Wang, *Asian J. Org. Chem.* **2017**, *6*, 1375.
2. P. Dominguez-Molano, R. Weeks, R. J. Maza, J. J. Carbó, E. Fernández, *Angew. Chem.Int. Ed*. **2023**, *62*, e2023047.
3. H. Wu, Q. Wang, J. Zhu, *Angew. Chem. Int. Ed.* **2018**, *57*, 2721.
4. Gaussian 16, Revision C.01, M. J. Frisch, G. W. Trucks, H. B. Schlegel, G. E. Scuseria, M. A. Robb, J. R. Cheeseman, G. Scalmani, V. Barone, G. A. Petersson, H. Nakatsuji, X. Li, M. Caricato, A. V. Marenich, J. Bloino, B. G. Janesko, R. Gomperts, B. Mennucci, H. P. Hratchian, J. V. Ortiz, A. F. Izmaylov, J. L. Sonnenberg, D. Williams-Young, F. Ding, F. Lipparini, F. Egidi, J. Goings, B. Peng, A. Petrone, T. Henderson, D. Ranasinghe, V. G. Zakrzewski, J. Gao, N. Rega, G. Zheng, W. Liang, M. Hada, M. Ehara, K. Toyota, R. Fukuda, J. Hasegawa, M. Ishida, T. Nakajima, Y. Honda, O. Kitao, H. Nakai, T. Vreven, K. Throssell, J. A. Montgomery, Jr., J. E. Peralta, F. Ogliaro, M. J. Bearpark, J. J. Heyd, E. N. Brothers, K. N. Kudin, V. N. Staroverov, T. A. Keith, R. Kobayashi, J. Normand, K. Raghavachari, A. P. Rendell, J. C. Burant, S. S. Iyengar, J. Tomasi, M. Cossi, J. M. Millam, M. Klene, C. Adamo, R. Cammi, J. W. Ochterski, R. L. Martin, K. Morokuma, O. Farkas, J. B. Foresman, and D. J. Fox, Gaussian, Inc., Wallingford CT, **2016**.
5. R. G. Parr, W. Yang, Oxford University Press: Oxford, U.K., **1989**.
6. J. D. Chai, M. Head-Gordon, *Phys. Chem. Chem. Phys.,* **2008**, *10*, 6615.
7. a) P. J. Hay, W. R. Wadt, *J. Chem. Phys.*, **1985**, *82,* 270 (b) P. J. Hay, W. R. Wadt, *J. Chem. Phys.*, **1985**, *82*, 284, (c) P. J. Hay, W. R. Wadt, *J. Chem. Phys.*, **1985**, *82*, 299.
8. a) A. Höllwarth, M. Böhme, S. Dapprich, A. W. Ehlers, A. Gobbi, V. Jonas, K. F. Köler, R. Stegmann, A. Veldkamp, G. Frenking, *Chem. Phys. Lett.* **1993**, *208*, 237; b) A. Höllwarth, M. Böhme, S. Dapprich, A. W. Ehlers, A. Gobbi, V. Jonas, K. F. Köler, R. Stegmann, A. Veldkamp, G. Frenking, *Chem. Phys. Lett.* **1993**, 111.
9. a) M. S. Gordon, *Chem. Phys. Lett.*, **1980**, *76*, 163; b) R. C. J. Binning, L. A. Curtiss, *Comput. Chem.* **1990**, *11*, 1206; c) A. D. McLean, G. S. Chandler, *J. Phys. Chem.*, **1980**, *72*, 5639.
10. A. V. Marenich, C. J. Cramer, D. G. J. Truhlar, *Phys. Chem. B*, **2009**, *113*, 6378.
11. a) S. Aguado-Ullate, S. Saureu, L. Guasch, J. J. Carbó, *Chem. Eur. J.* **2012**, *18*, 995, b) S. Aguado-Ullate, M. Urbano-Cuadrado, I. Villaba, E. Pires, J. I. García, C. Bo, J. J. Carbó, *Chem. Eur. J.* **2012**, *18*, 14026; (c) MolQuO application (accessed Nov 2023): http://rodi.urv.es/~carbo/quadrants/index.html
